# Broad and Region-Specific Impacts of the Synthetic Cannabinoid CP 55,940 in Adolescent and Adult Female Mouse Brains

**DOI:** 10.3389/fnmol.2018.00436

**Published:** 2018-11-27

**Authors:** Emma Leishman, Michelle N. Murphy, Michelle I. Murphy, Ken Mackie, Heather B. Bradshaw

**Affiliations:** ^1^Program in Neuroscience, Indiana University, Bloomington, IN, United States; ^2^Department of Psychological and Brain Sciences, Indiana University, Bloomington, IN, United States; ^3^Gill Center for Biomolecular Science, Indiana University, Bloomington, IN, United States; ^4^Department of Counseling and Educational Psychology, Indiana University, Bloomington, IN, United States

**Keywords:** lipidomics, endogenous cannabinoid, CNS, lipoamine, synthetic cannabinoid, prostaglandin

## Abstract

Relative to Δ^9^-tetrahydrocannabinol (THC), the synthetic cannabinoid CP 55,940 (CP) is significantly more potent and efficacious at cannabinoid receptors, the primary targets for endogenous cannabinoids (eCBs). eCBs belong to a large, interconnected lipidome of bioactive signaling molecules with a myriad of effects in optimal and pathological function. Recreational use of highly potent and efficacious synthetic cannabinoids is common amongst adolescents, potentially impacting brain development. Knowledge of the molecular outcomes of synthetic cannabinoid use will be important to develop more targeted therapies for synthetic cannabinoid intoxication and to prevent long-term disruption to the CNS. Here, we test the hypothesis that CP has age and region-dependent effects on the brain lipidome. Adolescent [post-natal day (PND) 35 and PND 50] and young adult female mice were given either an acute dose of CP or vehicle and brains were collected 2 h later. Eight brain regions were dissected and levels of ∼80 lipids were screened from each region using HPLC/MS/MS. CP had widespread effects on the brain lipidome in all age groups. Interestingly, more changes were observed in the PND 35 mice and more were reductions in a lipid’s concentration, including region-dependent lowering of eCB levels. CP levels were highest in the cortex at PND 35, the hippocampus at PND 50, and in the cerebellum in the adult. These data provide novel insights into how high-potency, synthetic cannabinoids drive different, age-dependent, cellular signaling effects in the brain.

## Introduction

The cannabis plant’s primary psychoactive component, THC activates the cannabinoid receptors CB_1_ (Devane et al., 1988) and CB_2_ (Munro et al., 1993), which are also targets for eCBs. With enriched expression in the brain, CB_1_ mediates most of the behavioral effects and abuse liability of THC (Tseng and Craft, 2004; Huestis et al., 2007). In addition to endogenous and plant-derived cannabinoids, there are synthetic cannabinoids that are typically more potent and efficacious at cannabinoid receptors (Castaneto et al., 2014; Kemp et al., 2016). One of the first synthetic cannabinoids synthesized was CP 55,940 (CP) (Uchiyama et al., 2009a). CP is a high efficacy agonist for cannabinoid receptors, whereas THC is considered a low efficacy agonist (Pertwee, 2008; Castaneto et al., 2014; Thomas, 2017). Likewise, CP has ∼10-fold higher potency than THC (Showalter et al., 1996; Besnard et al., 2012). For example, CP stimulated G-protein binding at CB_1_ with an EC_50_ of 3.4 nM, whereas THC had an EC_50_ of 167.4 nM (Brents et al., 2011). In another assay measuring functional inhibition of cAMP accumulation, CP was a more effective agonist at CB_1_ and CB_2_ receptors than THC. CP inhibited adenylate cyclase with an IC_50_ of 1.83 nM at CB_1_ and an IC_50_ of 2.89 nM at CB_2_, whereas THC had an IC_50_ of 16.5 nM at CB_1_ and 41.8 nM at CB_2_ (Felder et al., 1995).

Derived from AA, eCBs like AEA (Devane et al., 1992) and 2-AG (Mechoulam et al., 1995; Sugiura et al., 1995) belong to larger families of bioactive lipid signaling molecules. For example, AEA is a lipoamine, a fatty acid conjugated to an amine (Huang et al., 2001) (Supplementary Figure 1). Demonstrating effects on signaling beyond CB_1_, many AA-derived lipoamines, like AEA (Zygmunt et al., 1999), A-Taur (Saghatelian et al., 2006), and A-GABA are agonists at the capsaicin-sensitive TRPV1 channel (Raboune et al., 2014). NAGly is an AEA metabolite (Bradshaw et al., 2009a) that activates GPR18 and GPR55 (McHugh et al., 2010, 2012; Console-Bram et al., 2014, 2017). Using targeted screening methods, we regularly measure AEA, its lipoamine structural analogs, 2-AG, its 2-acyl glycerol structural analogs, AA-derived PGs, and free fatty acids in samples extracted from a variety of tissues, including mouse brain, and show that they belong to a wider, interconnected lipidome in the brain and throughout the body, with important roles in health and disease (Rimmerman et al., 2008; Bradshaw et al., 2009b; Smoum et al., 2010; Bradshaw and Allard, 2011; Raboune et al., 2014; Leishman et al., 2016a,b).

Previously, we demonstrated that developmental context influences acute THC’s effects on the brain’s lipidome. Specifically, the adult brain showed more and different changes in lipid levels 2 h after a single THC injection than the adolescent brain; however, the adolescent brains did show increases in PGs, whereas, the adult showed decreases (Leishman et al., 2018). Although plant-derived cannabis is the most widely used illicit drug (Miech et al., 2016), the misuse of synthetic cannabinoids, sold under brand names such as “spice” and “K2,” is prevalent (Castaneto et al., 2014; Gilbert et al., 2015; Müller et al., 2015; Kemp et al., 2016; Miech et al., 2016). Some of the effects of these compounds are severe and detrimental to public health (Castaneto et al., 2014; Müller et al., 2015). There are reports of heart attacks, kidney failure, psychosis, and even deaths in people who consumed synthetic cannabinoids (Davidson et al., 2017; Moeller et al., 2017; Samra et al., 2017; Manix et al., 2018). Tragically, most of these people were young and apparently healthy (Underwood, 2015; Samra et al., 2017; Manix et al., 2018). Given unknown effects on the developing brain, the prevalence of synthetic cannabinoid use amongst teenagers and young adults is of particular concern. For example, over 6% of high school seniors in the United States admitted to using synthetic marijuana in the past year in a 2014 survey and 2.9% were current synthetic cannabinoid users (Miech et al., 2016). In the European Union, over 7% of 15- to 18-year olds reported using spice or a similar drug in their lifetime (Müller et al., 2015). In animal models, the use of CP during adolescence had detrimental effects on motivated behaviors and memory in adulthood (Biscaia et al., 2003, 2008; O’Shea et al., 2004, 2006; Higuera-Matas et al., 2008, 2010; Chadwick et al., 2011; Mateos et al., 2011; Renard et al., 2013), which were often more severe in females (Biscaia et al., 2003, 2008; Higuera-Matas et al., 2008; Mateos et al., 2011). Here, we will use CP as an archetype for the many available high-affinity, synthetic cannabinoids, as they all share the commonality of being significantly more potent than THC (Castaneto et al., 2014; Fantegrossi et al., 2014; Kemp et al., 2016; Davidson et al., 2017).

Effects of CP (or any synthetic cannabinoid) on the broader lipidome have not been studied. Here, we test the hypothesis that CP exposure will broadly alter the brain lipidome and that these effects will be age-dependent. Providing a novel perspective of acute CP’s effects on the brain, this data will add to growing evidence that drugs targeting the eCB system have widespread effects on bioactive lipid regulation. This knowledge may aid in our understanding of how to treat synthetic cannabinoid overdoses that have deleterious effects on brain function.

## Materials and Methods

### Mice, Drug Injections, and Tissue Collection

The Bloomington Institutional Animal Care and Use Committee of Indiana University reviewed and approved the animal procedures. Female mice from the CD1 strain were utilized to match our study examining THC’s effects on the brain lipidome (Leishman et al., 2018). Mice were given a single i.p. injection of either 3 mg/kg CP or 1:1:18 cremophor:ethanol:saline vehicle (please refer to the section “3 mg/kg CP Is Relevant for Synthetic Cannabinoid Abuse and Has Stronger Effects on the Female Mouse Brain Lipidome During Adolescence” for a justification of dose and use of female mice). Three different age groups were given injections: PND 35 and PND 50 adolescents, and adult mice (PND 113). At least eight mice in each age group received CP and at least eight received vehicle. Matching the THC study time course (Leishman et al., 2018), mice were sacrificed via rapid decapitation 2 h after injection. Brains were immediately removed and flash-frozen in liquid nitrogen, and then stored at -80°C until dissections were performed. Brains were dissected on an ice-cold dissection plate into the following regions: STR, HIPP, CER, THAL, CTX, HYP, MID, and STEM. These abbreviations for these brain areas will be used exclusively when discussing the results generated by these specific dissections. Each dissected area was immediately placed in liquid nitrogen and then stored at -80°C until used for lipid extraction.

### Lipid Extraction and High-Pressure Liquid Chromatography Coupled to Tandem Mass Spectrometry (HPLC/MS/MS)

Tissue extracts were performed as previously described (Bradshaw et al., 2006; Stuart et al., 2013; Raboune et al., 2014; Leishman et al., 2016a,b, 2017, 2018). First, samples were shock frozen in liquid nitrogen, weighed, and transferred to a centrifuge tube. The mass of the largest sample was multiplied by 50 to determine how many milliliters of HPLC-grade methanol (Fisher, Fair Lawn, NJ, United States) to add. Then, samples were spiked with 500 pmol deuterium-labeled NAGly (d_8_NAGly; Cayman Chemical, Ann Arbor, MI, United States). Samples were placed on ice in darkness for 2 h then individually homogenized and centrifuged at 19,000 × *g* for 20 min at 20°C. Supernatants were diluted with HPLC water (purified in house) to make a solution of 75% water, 25% supernatant. Lipids were partially purified on C18 solid phase extraction columns (Agilent, Palo Alto, CA, United States). A series of four elutions with 1.5 mL of 60%, 75%, 85%, and 100% methanol were collected (Stuart et al., 2013; Leishman et al., 2016a,b, 2018).

As previously described (Bradshaw et al., 2006; Tan et al., 2006; Smoum et al., 2010; Stuart et al., 2013; Tortoriello et al., 2013; Raboune et al., 2014; Leishman et al., 2016a,b, 2017, 2018), samples were analyzed using an Applied Biosystems API 3000 triple quadrupole mass spectrometer with electrospray ionization (Foster City, CA, United States). Using an Agilent XDB-C18 reversed phase analytical column and optimized mobile phase gradients, 20 μL from each elution were chromatographed. Two Shimadzu 10ADvp pumps (Columbia, MD, United States) provided the pressure for gradient elution. Mobile phase A: 20% methanol, 80% water (v/v) and 1 mM ammonium acetate (Sigma, St. Louis, MO, United States). Mobile phase B: 100% methanol, 1 mM ammonium acetate. Every method run began with 0% mobile phase B, reached 100% mobile phase B flowing at 0.2 mL/min, and gradually returned to 0% mobile phase B.

### Data Analysis and Statistical Procedures

The Bradshaw lab possesses a screening library for selected lipoamines, 2-acyl glycerols, PGs, and free fatty acids, and has multiple reactions monitoring HPLC/MS/MS methods tailored for groups of structurally similar compounds to detect the ∼80 lipids in the library (Supplementary Figure 2). This screening library will be referred to as the lipidome when discussing data from this study. HPLC/MS/MS data were analyzed using Analyst software (Applied Biosystems) (Bradshaw et al., 2006; Tan et al., 2006; Stuart et al., 2013; Tortoriello et al., 2013; Raboune et al., 2014; Leishman et al., 2016a,b, 2017, 2018). Chromatograms (Supplementary Figure 3) were generated by determining the retention time of analytes from the analytical column with a [M-1] or [M+1] parent peak and a fragmentation peak corresponding to the programmed values. Thus, unknown lipids are matched to known standards according to retention time and their mass fingerprint.

Extraction efficiency was calculated with the d_8_NAGly spiked recovery vial as previously described (Bradshaw et al., 2006; Tan et al., 2006; Stuart et al., 2013; Tortoriello et al., 2013; Raboune et al., 2014; Leishman et al., 2016a,b, 2017, 2018). For each individual lipid in each of the areas, concentrations in moles per gram adjusted for extraction efficiency from the drug treated animals were compared to vehicle concentrations. As previously described (Leishman et al., 2016a,b), lipids with a high-degree of structural homology were grouped together and analyzed as a unit (e.g., NAEs) and analyzed using a one-way ANOVA with *post hoc* Fishers LSD. All statistical tests were carried out using SPSS (IBM, Armonk, NY, United States). Statistical significance was defined as *p* < 0.05 and trending at *p* < 0.10. Because experiments on different age groups were conducted several months apart, mean lipid levels could not be directly compared between age groups; instead, analyzed data are represented in tabular format illustrating both the direction and magnitude of change in lipid levels between CP and vehicle within an age group (key and explanation of calculations found in Supplementary Figure 4).

## Results

### Overall Effects of Acute CP on the CD1 Female Mouse Brain Lipidome

Visual observation of the PND 35, PND 50 and the adult mice given CP revealed profoundly reduced locomotor activity but no mortality. Of the 73 lipoamines in our screening library (Supplementary Figure 2), over 50 were detected in most brain regions in both the vehicle and CP-treated mice at each developmental time point. Of the lipids in the library, most were detected in larger brain regions such as the CTX and CER, and the fewest were detected in smaller regions like the HYP and STR. Each of the NAE, free fatty acid, and 2-acyl glycerol species analyzed were detected in all brain regions. There were 656 total discrete measures in endogenous lipids that could have been detected in each group (82 lipids in eight brain regions). 529 were detected in the PND 35 brain, 450 were detected in the PND 50 brain, and 406 were detected in the adult brain. The percentage of those modified with acute CP differed as a function of age wherein 43.67% of the lipids detected changed in the PND 35 brains, 31.33% in the PND 50 brains, and 23.89% in the adult brains (Supplementary Figures 5–7). As an example calculation, levels of 8 lipids changed in the adult STR, 12 in the HIPP, 21 in the CER, 15 in the THAL, 8 in the CTX, 7 in the HYP, 13 in the MID, and 13 in the STEM (Supplementary Figure 7). Summing those gives 97. 97 divided by 406 (number of lipids detected) and multiplied by 100 gives 23.89%. Full lists of analyte levels in each of the brain regions and the statistical analyses are available in Supplementary Tables 1–48.

#### Effects on CNS Lipid Levels by CP in PND 35

**FIGURE 1:**
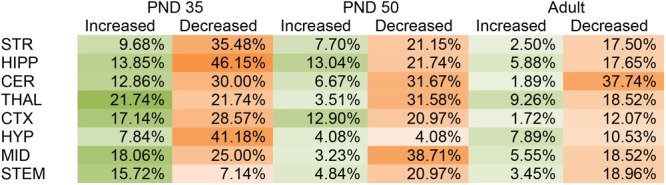
Percentage of significant changes in the CD1 female mouse brain lipidome in post-natal day (PND) 35, PND 50, and adult mice after 2-h exposure to systemic CP 55,940 (CP). The green portions of the figure represent the percentage of lipids detected in each brain area (row) that increased with acute 3 mg/kg CP relative to vehicle in each age group (column) and the orange parts represent the percentage of lipids detected in each brain area (row) that decreased with acute 3 mg/kg CP relative to vehicle in each age group (column). The areas with the most changes are shaded in darker colors. STR, striatum; HIPP, hippocampus; CER, cerebellum; THAL, thalamus; CTX, cortex; HYP, hypothalamus; MID, midbrain; STEM, brainstem. For example, in the adult MID, there were 54 lipids detected and 13 of them changed with acute CP. Of the 13 changes, 3 of them were increases. 3 was then divided by the number of lipids detected, which in this case was 54, and multiplied by 100 to yield the percentage increased (5.55%). For the adult MID, 10 lipids decreased with acute CP out of 54 detected, giving a percentage of 18.52% for the proportion of the lipidome that decreased.

In the PND 35 mice, most (152) of the 231 total changes in lipid levels across the eight regions were decreases; however, effects were region dependent. The HIPP had the most changes with 39 lipids changed out of 65 detected, whereas, the STEM had the fewest with 16 changes out of 70 detected. The STEM was the only brain area where most of changes were increases. The PND 35 mouse brain displayed changes common to all eight brain areas, including decreases in A-GABA and A-Taur and increases in *N*-palmitoyl leucine (Figure 1 and Supplementary Figure 5).

#### Effects on CNS Lipid Levels by CP in PND 50

Like in the PND 35 mice, most (110) of the 141 total changes in lipid levels in PND 50 mice were decreases. The MID was the most affected region of the PND 50 brain, with 26 total changes in lipid levels out of 62 detected. However, only two of these changes were decreases. In PND 50 mice, the HYP had the fewest changes, with only four lipids changing out of 49 detected (Figure 1 and Supplementary Figure 6).

#### Effects on CNS Lipid Levels by CP in the Adult

In the adult brain there were fewer changes in the lipidome compared to PND 35 and PND 50. Again, most (78) of the 97 changes in lipid levels were decreases. The region most affected in the adult brain was the CER, where levels of 21 different lipids were altered (20 decreases, 1 increase) out of 53 detected. In contrast, the CTX was the least affected brain region in the adult with only eight lipids changed out of 58 (Figure 1 and Supplementary Figure 7).

### Acute CP Modifies Lipid Levels Across the Brain in an Age-Dependent Fashion

#### Effects of Acute CP on Levels of Prostaglandins (PGs)

**FIGURE 2:**
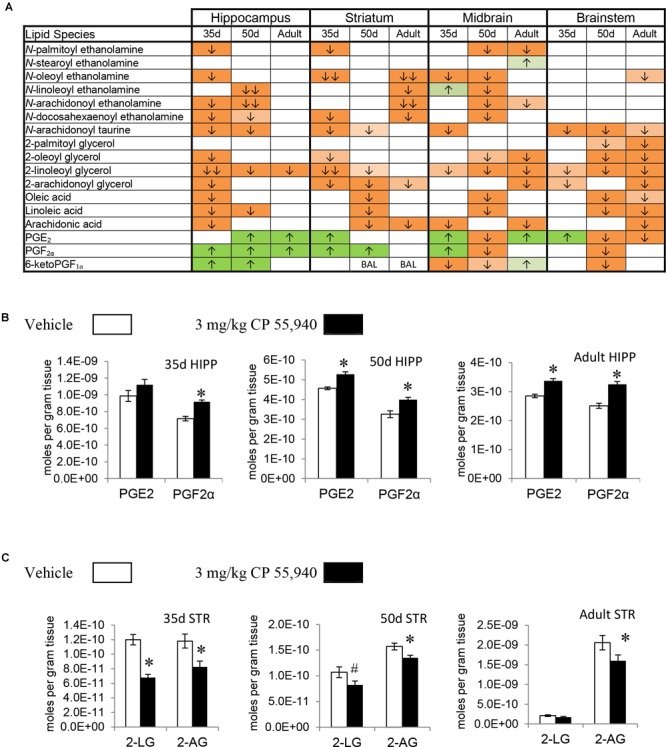
Effects of systemic 3 mg/kg CP 55,940 (CP) on levels of targeted lipids 2 h after injection in the post-natal day (PND) 35, PND 50, and adult CD1 female mouse hippocampus, striatum, midbrain, and brainstem. **(A)** Cells with shaded arrows indicate a change for that lipid in the CP-exposed brain area relative to the same vehicle-exposed area within each age group (35d = PND 35, 50d = PND 50). The arrow color indicates the direction of a significant result relative to control. Green colors represent increases, with darker green representing a significant increase of *p* < 0.05 and lighter green representing a trending increase of *p* < 0.10. Orange colors represent decreases in a lipid’s concentration, with darker orange indicating a significant decrease of *p* < 0.05 and light orange representing a trending decrease of *p* < 0.10. The number of arrows indicates the magnitude of the difference between CP and vehicle. One arrow indicates a magnitude difference of less than 1.5-fold and two arrows indicate a 1.5- to 1.99-fold change. BAL stands for “Below Analytical Limit,” whereas a blank cell indicates that there was no change in the lipid’s level due to CP. See Supplementary Figure 4 for a more detailed description of analysis. **(B)** Bar graphs showing mean levels of prostaglandin E_2_ (PGE_2_) and prostaglandin F_2α_ (PGF_2α_) in the post-natal day 35 hippocampus (35d HIPP), post-natal day 50 hippocampus (50d HIPP), and adult hippocampus (adult HIPP) 2 h after a systemic vehicle injection (open bars) or a systemic 3 mg/kg CP injection (black bars). The units on the *y*-axis are moles of lipid per gram of tissue. Error bars are ± standard error. An asterisk (^∗^) represents a difference of *p* < 0.05 between CP and vehicle groups. Levels of both these prostaglandins were higher in the 50d HIPP and adult HIPP (corresponding to green cells with one up arrow in panel **A**). In the 35d HIPP, levels of PGF_2α_ increased with CP treatment but levels of PGE_2_ did not change. **(C)** Bar graphs showing mean levels of 2-linoleoyl glycerol (2-LG) and 2-arachidonoyl glycerol (2-AG) in the post-natal day 35 striatum (35d STR), post-natal day 50 striatum (50d STR), and adult striatum (adult STR) 2 h after a systemic vehicle injection (open bars) or a systemic 3 mg/kg CP injection (black bars). The units on the *y*-axis are moles of lipid per gram of tissue. Error bars are ± standard error. Asterisk (^∗^) represents a difference of *p* < 0.05 between CP and vehicle groups and the pound sign (#) represents a trending difference of *p* < 0.10. In the 35d STR, 2-LG levels were lower in the CP-treated group (corresponding to a darker orange cell with two down arrows in panel **A**), whereas there was a smaller trending reduction in 2-LG in the 50d STR (corresponding to a lighter orange cell with one down arrow in panel **A**). No significant differences between groups in levels of 2-LG were found in the CP-treated adult STR. In all three age groups, levels of 2-AG were lower in the CP-exposed striatum (corresponding to darker orange cells with one down arrow in panel **A**).

**FIGURE 3:**
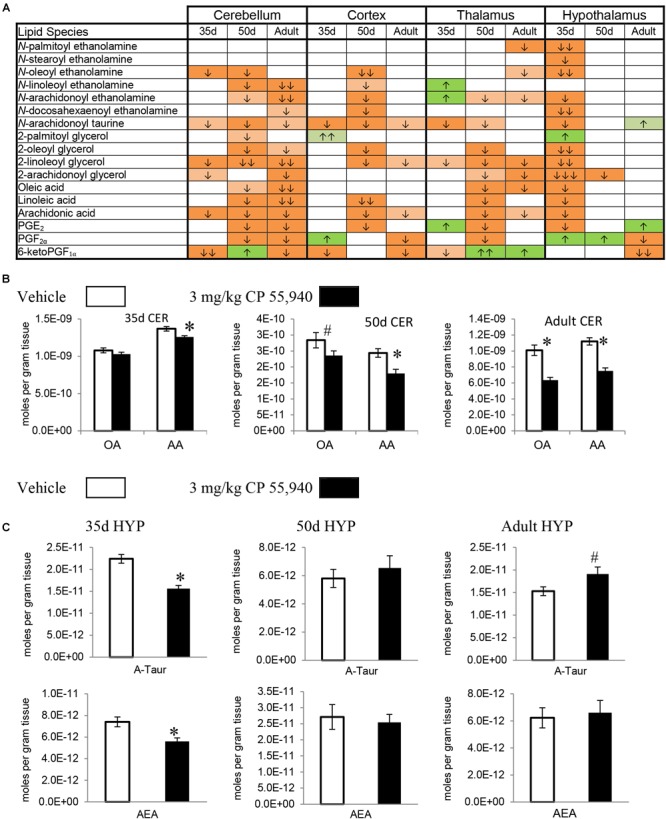
Effects of systemic 3 mg/kg CP 55,940 (CP) on levels of targeted lipids 2 h after injection in the post-natal day (PND) 35, PND 50, and adult CD1 female mouse cerebellum, cortex, thalamus, and hypothalamus. **(A)** Cells with shaded arrows indicate a change for that lipid in the CP-exposed brain area relative to the same vehicle-exposed area within each age group (35d = PND 35, 50d = PND 50). The arrow color indicates the direction of a significant result relative to control. Green colors represent increases, with darker green representing a significant increase of *p* < 0.05 and lighter green representing a trending increase of *p* < 0.10. Orange colors represent decreases in a lipid’s concentration, with darker orange indicating a significant decrease of *p* < 0.05 and light orange representing a trending decrease of *p* < 0.10. The number of arrows indicates the magnitude of the difference between CP and vehicle. One arrow indicates a magnitude difference of less than 1.5-fold, two arrows indicate a 1.5- to 1.99-fold change, and three arrows indicate a 2- to 2.99-fold change. A blank cell indicates that there was no change in the lipid’s level due to CP. See Supplementary Figure 4 for more detailed description of analysis. **(B)** Bar graphs showing mean levels of oleic acid (OA) and arachidonic acid (AA) in the post-natal day 35 cerebellum (35d CER), post-natal day 50 cerebellum (50d CER), and adult cerebellum (adult CER) 2 h after a systemic vehicle injection (open bars) or a systemic 3 mg/kg CP injection (black bars). The units on the *y*-axis are moles of lipid per gram of tissue. Error bars are ± standard error. Asterisk (^∗^) represents a difference of *p* < 0.05 between CP and vehicle groups and the pound sign (#) represents a trending difference of *p* < 0.10. In the 35d CER, there was no change in levels of OA. In the 50d CER, there was a trending decrease in OA (corresponding to a lighter orange cell with one down arrow in panel **A**), whereas there was an even larger decrease in OA in the adult CER (corresponding to an orange cell with two down arrows in panel **A**). In all three age groups, there was a reduction in AA levels in the CP-exposed cerebellum 2 h post-injection. **(C)** Bar graphs showing mean levels of *N*-arachidonoyl taurine (A-Taur) and *N*-arachidonoyl ethanolamine (AEA) in the post-natal day 35 hypothalamus (35d HYP), post-natal day 50 hypothalamus (50d HYP), and adult hypothalamus (adult HYP) 2 h after a systemic vehicle injection (open bars) or a systemic 3 mg/kg CP injection (black bars). The units on the *y*-axis are moles of lipid per gram of tissue. Error bars are ± standard error. Asterisk (^∗^) represents a difference of *p* < 0.05 between CP and vehicle groups and the pound sign (#) represents a trending difference of *p* < 0.10. In the 35d HYP, acute CP lowered levels of both A-Taur and AEA (corresponding to orange cells with one down arrow in panel **A**). In the 50d HYP, there are no significant differences in A-Taur or AEA levels between groups. Levels of A-Taur were trending higher in the CP-exposed adult HYP (corresponding to a lighter green cell with one up arrow in panel **A**), but AEA levels were unaffected in this age group.

Prostaglandins are a family of lipids with relatively well-understood receptors, biosynthesis, and metabolism (Funk, 2001). 15 changes (10 increases, 5 decreases) in PG levels were measured in the PND 35 brain, 5 for each PG screened. Apart from a decrease in PGE_2_ in the HYP, all changes in PGF_2α_ and PGE_2_ were increases. In contrast, changes in 6-keto PGF_1α_ were decreases except for an increase in the HIPP. The only brain area where all three PGs changed was the MID (Figures 2A,B, 3A and Supplementary Figure 5). With 7 increases and 11 decreases, most of the changes in PGs in the PND 50 brain were decreases, unlike the PND 35 brain. All 3 PGs differed in the HIPP, CER, THAL, MID, and STEM. In the HIPP, these changes were increases, whereas, in the MID and STEM, they were decreases (Figures 2A,B, 3A and Supplementary Figure 6). 14 changes (6 increases and 8 decreases) in PG levels were found in the adult brain. Levels of all three PGs changed in the adult CER and HYP (Figures 2A, 3A and Supplementary Figure 7). The only change in PGs common to all three age groups was an increase in PGF_2α_ in the HIPP (Figure 2B).

#### Effects of Acute CP on Levels of 2-Acyl Glycerols

2-AG, 2-PG, 2-oleoyl glycerol, and 2-LG are all 2-acyl glycerols (Jung et al., 2007). Concentrations of 2-acyl glycerols typically decreased in a region-specific manner, with the exception of increases in 2-PG in the PND 35 CTX and HYP. The other 15 changes in PND 35 mice were decreases. The HYP was the only region where all four 2-acyl glycerols changed. The 2-acyl glycerol most affected was 2-LG, which decreased in every area except the CTX. Five areas had decreases in levels of 2-AG: STR, HIPP, CER, HYP, and STEM (Figures 2A,C, 3A and Supplementary Figure 5). In PND 50 mice, there were 17 decreases in 2-acyl glycerol levels. 2-LG decreased in all brain areas except the HYP. There were three regions with decreases in 2-AG: the STR, HYP, and THAL (Figures 2A,C, 3A and Supplementary Figure 6). 15 reductions were uncovered in the adult brain. In the STEM, levels of all four 2-acyl glycerols decreased. 2-LG was also the most affected 2-acyl glycerol, decreasing in six brain areas. However, levels of 2-LG did not change in the STR or HYP. 2-AG decreased in the STR, CER, THAL, MID, and STEM (Figures 2A,C, 3A and Supplementary Figure 7). The decrease in 2-AG was common to all three age groups in the STR, and, in all three age groups, 2-LG was the most affected 2-acyl glycerol measured (Figure 2C).

#### Effects of Acute CP on Levels of Free Fatty Acids

All changes in free fatty acid levels were decreases, regardless of age group. Eight decreases were measured in the PND 35 brain. AA had more changes than linoleic or oleic acid. Levels of all three free fatty acids decreased in the HIPP and HYP, whereas no changes occurred in the STR, THAL, CTX, and STEM (Figures 2A, 3A,B and Supplementary Figure 5). With 16 decreases, the PND 50 brain had more changes in free fatty acids. Levels of all three fatty acids decreased in the STR, CER, and THAL. Linoleic acid was the most affected free fatty acid, decreasing in every region except the HYP (Figures 2A, 3A,B and Supplementary Figure 6). In the adult mice, there were 11 decreases. All three fatty acids decreased in the CER and STEM, which were the only areas where linoleic acid levels changed. The fatty acid most affected was AA, decreasing in the STR, CER, THAL, CTX, MID, and STEM (Figures 2A, 3A,B and Supplementary Figure 7). The decrease in AA in the CER was the only change common to all age groups (Figure 3B).

#### Effects of Acute CP on Levels of *N*-Acyl Ethanolamines

In the PND 35 brain, there were 17 changes in NAEs. The HYP had the most changes, with decreases in PEA, *N*-stearoyl ethanolamine, OEA, AEA, and DEA. The NAE most affected was OEA, decreasing in the STR, HIPP, CER, HYP, and MID. Levels of AEA decreased in the PND 35 HIPP and HYP but increased in the THAL and were unchanged in the STR, CER, CTX, MID, and STEM (Figures 2A, 3A,C and Supplementary Figure 5). In the PND 50 brain, all 16 changes in NAEs were decreases. With decreases in five NAEs (PEA, OEA, LEA, AEA, and DEA), the MID was the most affected brain area. AEA was the most altered NAE, decreasing in the HIPP, CER, THAL, CTX, and MID (Figures 2A, 3A,C and Supplementary Figure 6). For the adult brain, there were 15 alterations in NAEs, but none were found in the HIPP, CTX, and HYP. The area with the most changes in NAEs was the STR, with significant reductions in OEA, LEA, AEA, and DEA. AEA was the most affected NAE, decreasing in the STR, CER, THAL, and MID (Figures 2A, 3A,C and Supplementary Figure 7). None of the effects on NAE levels were measured in all three age groups, illustrating the importance of developmental context.

### Effect of Acute 3 mg/kg CP on Levels of Lipoamines Derived From Arachidonic Acid

**FIGURE 4:**
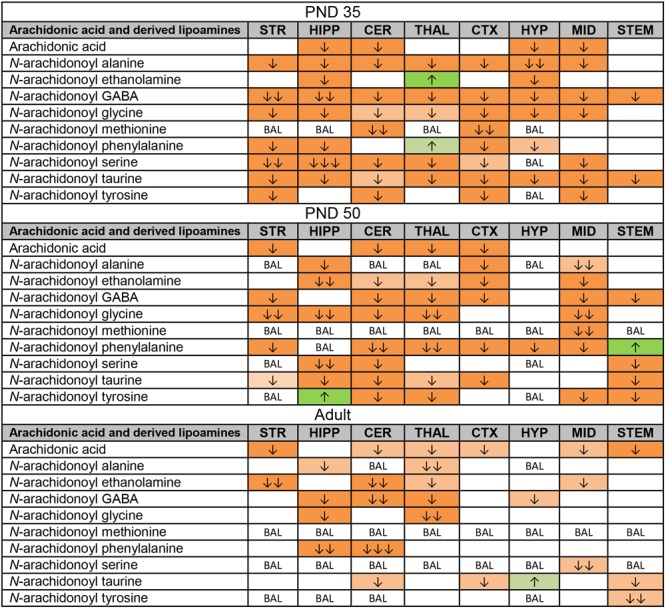
Effects of acute CP 55,940 (CP) on levels of arachidonic acid and arachidonic acid-derived lipoamines in eight regions of the post-natal day (PND) 35, PND 50, and adult female mouse brain. Effects in the PND 35 brain are shown in the top portion, the PND 50 effects are shown in the middle portion, and the effects in the adult brain are shown in the bottom portion of the figure. Cells with shaded arrows indicate a change for that lipid in the CP-exposed brain area relative to the same vehicle-exposed area within each age group. The arrow color indicates the direction of a significant result relative to control. Green colors represent increases, with darker green representing a significant increase of *p* < 0.05 and lighter green representing a trending increase of *p* < 0.10. Orange colors represent decreases in a lipid’s concentration, with darker orange indicating a significant decrease of *p* < 0.05 and light orange representing a trending decrease of *p* < 0.10. The number of arrows indicates the magnitude of the difference between CP and vehicle. One arrow indicates a magnitude difference of less than 1.5-fold, two arrows indicates a 1.5- to 1.99-fold change, and three arrows indicate a 2- to 2.99-fold change. BAL stands for “Below Analytical Limit,” whereas a blank cell indicates that there was no change in the lipid’s level due to CP. See Supplementary Figure 4 for more detailed description of analysis. Abbreviations for brain areas are: STR, striatum; HIPP, hippocampus; CER, cerebellum; THAL, thalamus; CTX, cortex; HYP, hypothalamus; MID, midbrain; STEM, brainstem.

Like the changes in AA itself, most changes in AA-derived lipoamines were decreases. Some of these decreases were common to all three age groups: *N*-arachidonoyl alanine in the HIPP, A-GABA in the CER and THAL, NAGly in the HIPP and THAL, and A-Taur in the CER, CTX, and STEM (Figure 4). With 50 differences, the PND 35 brain had the most changes in lipoamines derived from AA. Only two of these changes were increases. These were restricted to the THAL, where levels of AEA and A-Phe increased. A-GABA and A-Taur decreased across all eight brain areas (Figure 4). Compared to the PND 35 brain, fewer changes occurred in the PND 50 brain (41 changes). A-Phe increased in the STEM and *N*-arachidonoyl tyrosine increased in the HIPP, which were the only detected increases. A-Phe was the most affected AA-derived lipoamine, as levels decreased in seven of eight brain areas. The next most affected were A-GABA and A-Taur. A-GABA decreased in the STR, CER, THAL, CTX, MID, and STEM, and A-Taur decreased in the STR, HIPP, CER, THAL, CTX, and STEM (Figure 4). The adult mice had fewer changes in levels of AA-derived lipoamines (20 changes). The only increase was a modest increase in A-Taur in the HYP. With changes in four brain areas, the AA-derived lipoamines most affected were AEA, A-GABA, and A-Taur. A-GABA decreased in the HIPP, CER, THAL, and HYP. A-Taur levels fell in the CER, CTX, and STEM, but rose in the HYP (Figure 4). Overall, it appears that acute CP may mainly reduce signaling abilities of AA-derived lipoamines, and this effect is more profound in the adolescent brain.

### CP Levels Vary by Brain Region After Acute Administration

**FIGURE 5:**
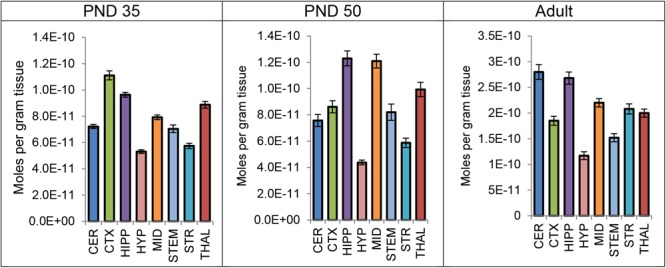
Average level of CP 55,940 in each of eight brain regions in post-natal day (PND) 35 adolescent, PND 50 adolescent, and adult female CD1 mice 2 h after a single systemic 3 mg/kg CP 55,940 injection. Lipid levels are in moles per gram of tissue and error bars represent the standard error of the mean. CER, cerebellum; CTX, cortex; HIPP, hippocampus; HYP, hypothalamus; MID, midbrain; STEM, brainstem; STR, striatum; THAL, thalamus.

Levels of CP were measured in each brain region 2 h after a systemic 3 mg/kg CP injection in PND 35, PND 50, and adult mice. A one-way ANOVA revealed a main effect of brain area on mean levels of CP in all three age groups, with levels varying twofold to threefold among the eight brain regions. Thus, the distribution of CP is not uniform throughout the brain 2 h after a single CP injection. In all three age groups, levels of CP were lowest in the HYP of the eight brain areas. However, the area with the highest levels of CP differed by age. In the PND 35 brain, levels of CP were highest in the CTX; in the PND 50 brain, levels of CP were highest in the HIPP; in the adult brain, levels of CP were highest in the CER (Figure 5). Please refer to the Supplementary Tables 49–54 for the full set descriptive statistics and ANOVA results regarding the distribution of CP amongst the eight brain areas.

## Discussion

CP 55,940 and other synthetic cannabinoids are highly potent and efficacious CB_1_ agonists associated with extreme psychological and physiological effects (Hudson and Ramsey, 2011; Castaneto et al., 2014; Fantegrossi et al., 2014; Davidson et al., 2017; Moeller et al., 2017; Samra et al., 2017; Manix et al., 2018). While there are similarities in some physiological effects between cannabis and synthetic cannabinoids, life-threatening reactions which have been attributed to synthetic cannabinoids are not associated with cannabis use alone (Underwood, 2015; Davidson et al., 2017; Manix et al., 2018). This is mostly attributed to higher potency and efficacy at CB_1_ (Pacher et al., 2018), although there are also “off-target” effects of synthetic cannabinoids and differences in metabolism that may be driving these adverse effects (Fantegrossi et al., 2014). The use of synthetic cannabinoids is prevalent in vulnerable populations such as adolescents (Gilbert et al., 2015; Müller et al., 2015). Here, we demonstrate that CP drives significant changes in the CNS lipidome that include but go well-beyond AEA and 2-AG, and that an acute dose of CP affects more lipids in the adolescent brain than the adult. Our data suggests that adolescents may be at particular risk for CNS insults with the use of synthetic cannabinoids, contributing to growing evidence that the use of CB_1_-acting drugs in adolescence is more harmful than during adulthood (O’Shea et al., 2004; Quinn et al., 2008; Abboussi et al., 2014; Moretti et al., 2014; Rubino and Parolaro, 2014, 2015).

### 3 mg/kg CP Is Relevant for Synthetic Cannabinoid Abuse and Has Stronger Effects on the Female Mouse Brain Lipidome During Adolescence

The dose of 3 mg/kg was chosen to match the dose used in our previous analysis of the effects of acute THC on the lipidome of adolescent and adult female mouse brain regions (Leishman et al., 2018). 3 mg/kg THC was estimated to be equivalent to smoking 1 joint for humans (Zamberletti et al., 2012). Given the differences in potency and efficacy between CP and THC, the degree of CB_1_ activation would be much greater after 3 mg/kg CP compared to 3 mg/kg THC. However, synthetic cannabinoid users do not typically consider the differences in potency. Many people use synthetic cannabinoids in the same manner as natural cannabinoids, meaning that if they normally smoke 1 joint of cannabis, then they will smoke 1 joint of spice, even if this causes a much stronger intoxication (Lapoint et al., 2011). Indeed, popular press articles sometime refer to synthetic cannabinoid users as “spice zombies” because of drug-induced catalepsy (Alexandrescu, 2018; BBC News, 2018). Providing more evidence of failure to titrate dose based on potency, patients seeking treatment for synthetic cannabinoid abuse consumed significantly more drug (4.05 g/day) than those for cannabis use disorder (2.31 g/day) (Cengel et al., 2018). CP has not been found in spice, likely due to the fact that it was a known CB_1_ agonist and easily measured by law enforcement. However, given that CP is a canonical synthetic cannabinoid (Lefever et al., 2017) and that CP analogs with similar potency at CB_1_ were identified in spice products (Uchiyama et al., 2009b; Hermanns-Clausen et al., 2013), CP is a suitable candidate to model synthetic cannabinoid abuse. At the 3 mg/kg dose, there were more changes in the female mouse brain lipidome as a consequence of acute CP exposure compared to acute THC exposure, although both drugs reduced eCB levels in some brain areas. This difference was most apparent in the PND 35 brain (Leishman et al., 2018). A follow-up study could use equipotent doses of CP and THC based on tetrad effects to help determine if the increased impact of CP on the lipidome relative to THC is due to increased potency. However, CP and THC are not specific to CB_1_ and CP can also activate CB_2_ and GPR55 (Ryberg et al., 2007). To examine the contribution of CB_1_ to the effects of CP and THC on the lipidome, a CB_1_ antagonist could be co-administered with CP and with THC in future studies.

In both the present study and the THC study, we chose to examine female mice. In the rodent literature, the effects of adolescent exposure to CP were often more severe in females (Biscaia et al., 2003, 2008; Higuera-Matas et al., 2008; Mateos et al., 2011). This is also the case with THC (Zamberletti et al., 2012). Although most of the people who experienced adverse reactions from synthetic cannabinoids were male, there are also documented cases of acute detrimental effects of synthetic cannabinoids in young women involving emergency room visits (Schneir et al., 2011). The higher prevalence of adverse effects in men might be due to higher rates of risky behavior in general. However, more recent surveys demonstrate that male and female adolescents are now equally likely to use illegal drugs (Becker et al., 2017). Making it important to study the effects of drugs of abuse in both sexes, there are sex differences at every stage of drug abuse and addiction (Becker et al., 2017). For example, women tend to increase the rate of alcohol, marijuana, opioids, and cocaine use more rapidly than men do (Lynch et al., 2002). These sex differences as well as the worrying increase in drug use amongst young women in recent years mean that females are worth examining. Follow up studies should test whether some of the effects of acute CP on the lipidome are sex dependent.

CP 55,940 may have developmental-dependent effects because the eCB system is dynamic throughout adolescence (Heng et al., 2011; Lee et al., 2013). We selected to use PND 35 (mid adolescent) and PND 50 (older adolescent) mice to determine whether changes in the lipidome in response to THC and CP are sensitive to the stage of adolescence. There is evidence that levels of AEA are higher in the PND 35 and young adult amygdala, HYP, prefrontal cortex, and HIPP compared to in these areas at PND 45. This was also the case with OEA and PEA and demonstrates that there is fluctuation in NAE production during development, with levels increasing, decreasing, and then increasing again (Lee et al., 2013). However, this study was performed in rats and there needs to be a more investigation whether this is the case in mice and whether lipoamines beyond NAEs follow a similar pattern. In male rats, levels of 2-AG were measured during adolescence in the pre-frontal cortex (Ellgren et al., 2008). 2-AG levels were highest at PND 29, then declined in mid adolescence (PND 38), before increasing again during late adolescence (PND 50) (Ellgren et al., 2008). In the same set of experiments, a different pattern was uncovered with AEA, wherein levels gradually increased during adolescence (Ellgren et al., 2008). In the female rat pre-frontal cortex, increases in AEA were found from PND 46–60, but decreases were measured between PND 60 and 70. However, no significant changes in 2-AG, MAGL, or FAAH were found in these rats (Rubino et al., 2015). Studies suggest CB_1_ expression is higher during early adolescence and declines with age (Ellgren et al., 2008; Heng et al., 2011; Lee and Gorzalka, 2012). Although there are discrepancies in the literature, which may be partially due to species and sex differences, the most consistent pattern is that eCB ligands fluctuate during adolescence, whereas CB_1_ expression peaks during early adolescence and declines toward adulthood (Lee et al., 2016). The relatively higher levels of CB_1_ in the PND 35 brain may potentially explain why a drug that strongly activates CB_1_ like CP had more effects at this time point.

### CP Causes Catalepsy and May Drive Neuroinflammation

Mice treated with CP in this study were most-likely cataleptic, as they had reduced locomotor activity. Showing that the dose given in this study was more than enough to produce catalepsy, the ED_50_ of CP is 0.4 mg/kg for catalepsy (Little et al., 1988; Compton et al., 1992). A single 0.3 mg/kg injection of CP produced catalepsy in C57 mice, but not in CB_1_ KO mice. This provides further evidence that the characteristic behavioral signs of CP consumption are mediated by CB_1_ (Kow et al., 2014). Catalepsy is linked to psychopathologies including schizophrenia (Fink et al., 2009). Recent translational studies have implicated abnormalities in the myelin protein CNP in the development of catalepsy. Reduced CNP levels generate neuroinflammation and reducing inflammation ameliorated catalepsy in mice (Pease-Raissi and Chan, 2018). The eCB system regulates inflammation, with AEA and 2-AG typically having anti-inflammatory effects (Donvito et al., 2017). The fact that these lipids decreased with CP means that the risk of neuroinflammation could potentially increase after CP exposure. Other AA-derived lipoamines are noted to have anti-inflammatory effects in the CNS, such as A-Ser and NAGly (Hanuš et al., 2014). A-Ser and NAGly decreased in some brain areas of CP-exposed mice, especially in PND 35 mice, suggesting a pro-inflammatory lipid environment in these regions that might contribute to catalepsy. CP’s effects on CB_2_ might affect the brain’s ability to respond to inflammatory stimuli, as CB_2_ is expressed on microglia (Merighi et al., 2012). Furthermore, there is now evidence that microglia have a role in healthy brain development, as they can support synapses (Lenz and McCarthy, 2015). CB_2_ can also be desensitized after 24 h exposure to CP (Shoemaker et al., 2005), so this receptor might also contribute to longer term effects of CP. CB_2_ KO mice show some schizophrenia-like behaviors (Ortega-Alvaro et al., 2011), and adolescent exposure to cannabinoid receptor agonists is linked to increased risk of developing schizophrenia (Murray et al., 2017). Therefore, the contribution of CB_2_ to detrimental effects of synthetic cannabinoids should also be examined, even if the actions at CB_2_ are not causing immediate behavioral effects.

### Motivation and Psychosis – Effects on Dopaminergic Circuitry

Acutely, CP can decrease female sexual motivation, a process reliant on dopamine transmission. However, doses of over 0.4 mg/kg also attenuated social motivation (Ferrari et al., 2000; Lopez et al., 2009; Gorzalka et al., 2010), suggesting a general effect on motivational circuitry. This circuitry is considered part of the brain’s reward system and is also critical for the neurobiology of addiction (Koob and Volkow, 2010). In preclinical models, the use of CP during adolescence has been linked to increased administration of drugs of abuse like cocaine or morphine during adulthood (Biscaia et al., 2008; Higuera-Matas et al., 2010). Higuera-Matas et al. (2010) gave rats a daily 0.4 mg/kg CP injection from PND 28 until PND 38. At PND 75, cocaine self-administration was measured. Female rats exposed to CP during adolescence administered significantly more cocaine than rats exposed to vehicle. The use of CP and the increase in cocaine self-administration were correlated with changes in expression of genes encoding proteins of the striatal and hippocampal dopaminergic circuitry (Higuera-Matas et al., 2010). Specifically, CP administration during adolescence upregulated the DAT in the caudate-putamen and decreased dopamine D2 receptor levels in the HIPP (Higuera-Matas et al., 2010). Therefore, the non-voluntary exposure of rats to CP during adolescence has short and long-term impacts on motivated behaviors, potentially by down-regulating dopamine receptor expression and upregulating levels of DAT.

Excessive dopamine signaling is involved in psychosis and dopamine D2 receptors are often a target for anti-psychotic drugs (Howes and Kapur, 2009). Effects of synthetic cannabinoids on the dopaminergic system might contribute to the psychosis seen in users. CP is aversive to rodents (McGregor et al., 1996) and aversive stimuli can actually increase dopamine transmission (Brischoux et al., 2009), which could drive psychosis-like symptoms. Additionally, there is evidence that CB_1_ activation drives dopamine release (French et al., 1997), making it likely that acute doses of CP increase dopamine signaling even without evidence of rewarding effects. 2-AG signaling may act to dampen dopamine release (Oleson and Cheer, 2012), meaning that reductions in 2-AG found in the CP-exposed brain areas may trigger dopamine release. Administration of THC and the synthetic analog, Nabilone, can produce schizophrenia-like symptoms in otherwise healthy adults (Murray et al., 2017). These symptoms can be ameliorated by the plant cannabinoid CBD, which has generated the hypothesis that CB_1_-activating cannabinoids are psychotomimetic, whereas those that negatively modulate CB_1_ can be anti-psychotic (Murray et al., 2017). Interestingly, there may be opposing effects of CBD and CP on levels of AEA. For example, CBD increased levels of AEA in BV-2 microglial cells (Rimmerman et al., 2012) and in the CSF of patients with schizophrenia, correlating with improved symptoms (Leweke et al., 2012). In contrast, levels of AEA instead tended to decrease in brains exposed to CP and in adult brains exposed to THC (Leishman et al., 2018). Therefore, alterations in AEA may be indicative of whether a drug has pro-psychotic or anti-psychotic effects.

What mechanisms could lead to a reduction in AEA? There are multiple pathways to yield AEA from its precursor *N*-acyl phosphatidylethanolamine (NAPE), but the most direct is NAPE’s hydrolysis by a NAPE-specific phospholipase D (NAPE-PLD) (Di Marzo et al., 1994). In the less direct, alternative pathways: intermediates lyso-NAPE (Simon and Cravatt, 2006) or phospho-AEA (Liu et al., 2006) are synthesized from NAPE and are further processed to produce AEA. Reductions in brain levels of AEA and NAEs could be driven by inhibition of NAPE-PLD. Our previous study of NAPE-PLD KO mice demonstrated that NAPE-PLD deletion downregulates NAEs. PGs are also upregulated in NAPE-PLD KO brain areas, but there were very few changes in 2-acyl glycerols (Leishman et al., 2016a). This makes NAPE-PLD a more likely candidate to drive the decreases in AEA in areas like the PND 50 HIPP, where all 3 PGs increased and AEA decreased, but wherein there were no effects on AA or 2-AG. However, there may be other pathways contributing to the effects of CP outside of NAPE-PLD inhibition. NAPE-PLD activity can be measured using recombinant enzymes and inhibitors of NAPE-PLD are now available (Castellani et al., 2017) to more directly test this hypothesis. AEA is primarily hydrolyzed by fatty acid amide hydrolase (FAAH) (Cravatt et al., 1996). Therefore, an alternative hypothesis is that CP upregulates FAAH activity to decrease AEA levels. Previous work by our group in FAAH KO mice has demonstrated that FAAH deletion increases AEA at the expense of AA-derived lipoamines such as NAGly and has no effect on PGs or free AA (Leishman et al., 2016a). The fact that these lipids changed with CP calls into question whether it is acting on FAAH. However, the consequences of FAAH upregulation on the lipidome have not yet been examined. To test whether FAAH is required for CP to decrease AEA, our acute CP experiments can be repeated in FAAH KO mice.

### CP Distribution

CP 55,940 is a member of the cyclohexylphenol category of synthetic cannabinoids, whose structures are different from classical cannabinoids like THC and are instead bicyclic (Hudson and Ramsey, 2011). However, CP is still lipophilic in nature and can be detected using similar methods to the detection of THC: lipid extraction and analysis with liquid (or gas) chromatography and mass spectrometry (Uchiyama et al., 2009a; Hudson and Ramsey, 2011). Previously, we showed that THC levels were highest in the HIPP after acute exposure across all age groups (Leishman et al., 2018). Similarly, here, hippocampal CP levels were among the two highest brain regions across all age groups. Correlated with enriched CB_1_ expression in the HIPP (Herkenham et al., 1990), the enriched levels of CP in the HIPP may contribute to deficits in learning and memory (Han et al., 2012) and emotional processing (Loureiro et al., 2016). In contrast to THC, levels of CP were lowest in the HYP across all age groups. One potential explanation is localized metabolism of CP. However, standards for CP metabolites are not readily available for analysis (Jin et al., 2013). It is possible that like THC’s primary metabolite, 11-OH-THC (Lemberger et al., 1972), some of the CP metabolites may have behavioral effects.

### Potential Mechanisms of CP on CNS Lipidome Regulation

One of the signatures of acute CP on the lipidome was a widespread reduction in lipid levels, especially in levels of lipids derived from AA. High levels of CB_1_ activation may partially underlie these effects. Activating CB_1_ on a presynaptic neuron can decrease neurotransmitter release (Wilson and Nicoll, 2001). However, neurotransmitter release can trigger eCB production in the post-synaptic neuron (Lu and Mackie, 2016). As there is now less neurotransmitter release this could in turn decrease the release of eCBs. The production of eCBs depends on the availability of precursors and enzymes. Although CP affected more lipids than eCBs, this implicates eCB system enzymes as potential mediators of CP’s effects because these enzymes regulate lipid levels beyond eCB substrates, including those of the AA family (Leishman et al., 2016a,b; Bradshaw and Leishman, 2017). DAGLs synthesize 2-acyl glycerols (Bisogno et al., 2003). The DAGLα isoform appears more important for biosynthesis of 2-AG in the brain, whereas the DAGLβ isoform appears more important in the periphery and immune cells (Reisenberg et al., 2012). Along with 2-AG levels, AEA levels were downregulated in the CER, STR, and HIPP of a line of DAGLα KO mice (Tanimura et al., 2010). Another line of DAGLα KO had an 80% reduction in whole brain 2-AG and AA levels and a 40% reduction in AEA (Gao et al., 2010). A third line of DAGLα KO had reduced levels of 2-AG and AA in the forebrain, but did not detect a significant reduction in AEA in this area, hinting at some regional specificity for the contribution of DAGLα activity to AEA levels (Shonesy et al., 2014). Reductions in whole-brain 2-AG, AA, and AEA also occur when DAGLα is pharmacologically inhibited (Ogasawara et al., 2016). The reductions in 2-AG, AA, and AEA levels were temporary, being measured at 2 h and 4 h post-injection of the DAGLα inhibitor DH376 and returning to baseline by 8 h (Ogasawara et al., 2016). It is thus possible that DAGL is inhibited in the brains of mice exposed to CP, as DAGL inhibition could cause a concurrent decrease in 2-AG, AA, and AEA that could be detected at 2 h.

It is not yet known how levels of AA-derived lipoamines change in DAGLα KO or with DAGLα inhibitors, as a full lipidomics screen of multiple brain areas has not yet been applied to DAGLα KO or to mice exposed to DAGLα-blocking drugs. However, if levels of AA-derived lipoamines are reduced when DAGLα is blocked, then that would provide a stronger suggestion of that enzyme’s role in CP’s effects on the lipidome. Follow-up studies can determine if the effects of acute CP are abolished when DAGLα is inhibited or deleted. If CP’s effects on the lipidome require DAGLα, then more direct measurements of DAGLα activity can be performed in the presence of various concentrations of CP. Recently, a fluorescent probe was developed to measure DAGLα activity (van der Wel et al., 2015). This probe can be used in ABPP assays on cells overexpressing DAGL (Hsu et al., 2012) or in mouse brain proteasomes (Baggelaar et al., 2013). By performing ABPP in the presence and absence of CP, then CP inhibition of DAGLα can be quantified. Another advantage of ABPP is the ability to detect off-target serine hydrolase activity by incubating with an FP-biotin probe (Liu et al., 1999), which may reveal an even more widespread impact of acute CP on enzymes important for maintaining levels of bioactive lipids.

One of the roles of 2-AG is in appetite stimulation and CP might potentially reduce appetite by acting on 2-AG. An acute injection of 0.1 mg/kg CP in rats produced anorexic effects lasting up to 24 h (McGregor et al., 1996). Two additional studies demonstrated that food intake was temporarily reduced when rats were given chronic injections of CP in adolescence (Biscaia et al., 2003; Mateos et al., 2011). Data here shows that PND 35 and PND 50 adolescent mice had lower levels of 2-AG in the HYP after acute CP. The HYP is an important region for appetite regulation and feeding behavior and 2-AG stimulates eating (Kirkham et al., 2002). Thus, reductions in 2-AG in the HYP could contribute to anorexic effects of CP. People who are overweight or obese tend to have higher circulating levels of eCBs (Di Marzo, 2008). However, giving them CP would not be an ideal, due to the effects on locomotion and cognition. Instead, if we can elucidate some of the pathways used by CP to reduce lipid levels, we can develop a more targeted drug to ameliorate the disruptions in lipid metabolism. For example, a DAGL inhibitor could be used instead of CP. Indeed, there are already groups working on developing DAGL inhibitors for metabolic disorders, after it was noted that DAGL KO animals are leaner and consume less food than WT littermates (Janssen and van der Stelt, 2016). DAGL inhibitors are also being investigated for inflammatory pain (Wilkerson et al., 2017), which could replicate some of the analgesic effects of CP without the abuse liability.

As revealed by proteomics, a structurally similar drug to CP with similar pharmacology, CP 47,497-C8, alters expression of multiple enzymes involved in lipid metabolism. Specifically, when human peripheral blood mononuclear cells were stimulated with 10 μM CP 47,497-C8 for 3 h, expression of 5-LOX was upregulated 26-fold compared to vehicle, expression of fatty acid synthase increased over twofold, and expression of group XV phospholipase A2, long-chain-fatty-acid-CoA ligase 1, ABHD12, phospholipase D1, and thromboxane-A synthase significantly increased (Bileck et al., 2016). These enzymes are all involved in inflammation and levels of pro-inflammatory cytokines increased in the cells exposed to CP 47,497-C8 (Bileck et al., 2016). There was also evidence of DNA damage in these cells caused by inhibition of repair mechanisms (Bileck et al., 2016). Inflammation and DNA damage may contribute to the adverse health outcomes in synthetic cannabinoid users (Bileck et al., 2016). Because CP is so structurally similar, it is possible that CP has similar effects on enzyme levels, but this has not yet been tested. The increase in ABHD12 is of particular interest because it is involved in 2-acyl glycerol hydrolysis (Savinainen et al., 2012). An increase in ABHD12 activity may contribute to reductions in 2-acyl glycerols. Decreases in AA and its derivatives may also be driven by an increase in 5-LOX activity (Rouzer and Marnett, 2011). The widespread effect of CP on lipid levels may be due to its ability to affect multiple enzymes involved in lipid metabolism.

## Conclusion

The potent and efficacious synthetic cannabinoid, CP, affects the lipidome in a brain-region and development-dependent manner and appears to have far-reaching effects on multiple lipid signaling pathways. These effects were region and age-dependent providing additional clues to how the consequences of this specific class of drugs of abuse must be considered differentially between populations. Overall, acute CP altered the levels of more lipids in younger brains, suggesting that dysregulation of the lipidome is more vulnerable to CP during adolescence. CP caused region-dependent reductions in eCB levels, possibly due to CB_1_ activation. Future studies will compare the effects of THC and CP using CB_1_ KO mice and CB_1_ antagonists to better determine the contribution of CB_1_ activity. However, the effects of CP are not only more widespread in the CNS; they have dramatically more impact in the adolescent. Understanding how synthetic cannabinoids differentially affect the brain and its development will allow us to better treat those who are using these drugs but will also allow us to better educate about their abuse liabilities.

## Author Contributions

EL performed all lipid extractions, collected and analyzed HPLC/MS/MS data, assisted with tissue collection, and prepared the manuscript. MNM and MIM performed drug and vehicle injections. KM provided the mice and assisted with manuscript preparation. HB designed the experiments, collected tissue, performed dissections, and prepared the manuscript.

## Conflict of Interest Statement

The author, HB, of this manuscript is on the Advisory Board for Phytecs and consults on how endogenous cannabinoids function in the central nervous system. Phytecs had no financial contribution to the current work. The remaining authors declare that the research was conducted in the absence of any commercial or financial relationships that could be construed as a potential conflict of interest.

## Abbreviations

2-AG: 2-arachidonoyl glycerol
2-LG: 2-linoleoyl glycerol
2-PG: 2-palmitoyl glycerol
5-LOX: 5-lipoxygense
AA: arachidonic acid
ABHD12: α/β-hydrolase domain 12
ABPP: activity based protein profiling
AEA: *N*-arachidonoyl ethanolamine (anandamide)
A-GABA: *N*-arachidonoyl GABA
A-Phe: *N*-arachidonoyl phenylalanine
A-Ser: *N*-arachidonoyl serine
A-Taur: *N*-arachidonoyl taurine
CBD: cannabidiol
CER: cerebellum (specific to this study)
CNP: 2′3′-cyclic nucleotide 3′-phosphodiesterase
CP: CP 55,940
CTX: cortex (specific to this study)
d_8_NAGly: deuterium labeled *N*-arachidonoyl glycine
DAGL: diacylglycerol lipase
DAT: dopamine transporter
DEA: *N*-docosahexaenoyl ethanolamine
eCB: endogenous cannabinoid
HIPP: hippocampus (specific to this study)
HPLC/MS/MS: high pressure liquid chromatography coupled with tandem mass spectrometry
HYP: hypothalamus (specific to this study)
KO: knockout
LEA: *N*-linoleoyl ethanolamine
MID: midbrain (specific to this study)
NAE: *N*-acyl ethanolamine
NAGly: *N*-arachidonoyl glycine
OEA: *N*-oleoyl ethanolamine
PEA: *N*-palmitoyl ethanolamine
PG: prostaglandin
PND: post-natal day
STEM: brainstem (specific to this study)
STR: striatum (specific to this study)
THAL: thalamus (specific to this study)
THC: Δ^9^-tetrahydrocannabinol
