# Supplementary material for: Broad and Region-Specific Impacts of the Synthetic Cannabinoid CP 55,940 in Adolescent and Adult Female Mouse Brains
Source: Front Mol Neurosci. 2018 Nov 27;11:436. doi: 10.3389/fnmol.2018.00436 (PMC6277767; doi:10.3389/fnmol.2018.00436)
Supplement: Supplementary file 1 [file Table_1.DOCX]

Supplementary Material

Broad and Region-Specific Impacts of the Synthetic Cannabinoid CP 55,940 in Adolescent and Adult Female Mouse Brains

Emma Leishman, Michelle N Murphy, Michelle I Murphy, Ken Mackie, Heather B Bradshaw*

*** Correspondence:** Corresponding Author: hbbradsh@indiana.edu

# Supplementary Figures and Tables

7 Supplementary Figures and 54 Supplementary Tables.

## Supplementary Figures

**
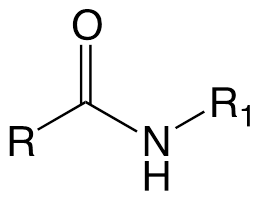
**

R = fatty acid R_1_ = amine

**Supplemental Figure 1.** Generic structure of a lipoamine, a fatty acid conjugated to an amine via an amide bond

| *N*-acyl alanine | [M–H]^-^ | Fragment |  | *N*-acyl proline | [M–H]^-^ | Fragment |
| --- | --- | --- | --- | --- | --- | --- |
| *N*-palmitoyl alanine | 326.5 | 88.09 |  | *N*-palmitoyl proline | 352.53 | 114.12 |
| *N*-stearoyl alanine | 354.55 | 88.09 |  | *N*-stearoyl proline | 380.59 | 114.12 |
| *N*-oleoyl alanine | 352.53 | 88.09 |  | *N*-oleoyl proline | 378.31 | 114.12 |
| *N*-linoleoyl alanine | 350.52 | 88.09 |  | *N*-linoleoyl proline | 376.56 | 114.12 |
| *N*-arachidonoyl alanine | 374.5 | 88.09 |  | *N*-arachidonoyl proline | 400.58 | 114.12 |
| *N*-docosahexaenoyl alanine | 398.56 | 88.09 |  | *N*-docosahexaenoyl proline | 424.6 | 114.12 |
| *N*-acyl dopamine | [M–H]^-^ | Fragment |  | *N*-acyl serine | [M–H]^-^ | Fragment |
| *N*-oleoyl dopamine | 416.3 | 123.2 |  | *N*-palmitoyl serine | 342.3 | 74 |
| *N*-arachidonoyl dopamine | 438.4 | 123.2 |  | *N*-stearoyl serine | 370.3 | 74 |
| *N*-acyl ethanolamine | [M+H]^+^ | Fragment |  | *N*-oleoyl serine | 368.3 | 74 |
| *N*-palmitoyl ethanolamine | 300.29 | 62.1 |  | *N*-linoleoyl serine | 366.27 | 74 |
| *N*-stearoyl ethanolamine | 328.3 | 62.1 |  | *N*-arachidonoyl serine | 390.3 | 74 |
| *N*-oleoyl ethanolamine | 326.3 | 62.1 |  | *N*-docosahexaenoyl serine | 414.3 | 74 |
| *N*-linoleoyl ethanolamine | 324.3 | 62.1 |  | *N*-acyl taurine | [M–H]^-^ | Fragment |
| *N*-arachidonoyl ethanolamine | 348.29 | 62.1 |  | *N*-arachidonoyl taurine | 410.6 | 124 |
| *N*-docosahexaenoyl ethanolamine | 372.6 | 62.1 |  | *N*-acyl tryptophan | [M–H]^-^ | Fragment |
| *N*-acyl GABA | [M–H]^-^ | Fragment |  | *N*-palmitoyl tryptophan | 441.63 | 203.1 |
| *N*-palmitoyl GABA | 340.54 | 102.1 |  | *N*-stearoyl tryptophan | 469.68 | 203.1 |
| *N*-stearoyl GABA | 368.58 | 102.1 |  | *N*-oleoyl tryptophan | 467.67 | 203.1 |
| *N*-oleoyl GABA | 366.57 | 102.1 |  | *N*-linoleoyl tryptophan | 465.65 | 203.1 |
| *N*-linoleoyl GABA | 364.54 | 102.1 |  | *N*-arachidonoyl tryptophan | 489.67 | 203.1 |
| *N*-arachidonoyl GABA | 388.57 | 102.1 |  | *N*-docosahexaenoyl tryptophan | 513.69 | 203.1 |
| *N*-docosahexaenoyl GABA | 412.59 | 102.1 |  | *N*-acyl tyrosine | [M–H]^-^ | Fragment |
| *N*-acyl glycine | [M–H]^-^ | Fragment |  | *N*-palmitoyl tyrosine | 418.59 | 180.18 |
| *N*-palmitoyl glycine | 312.26 | 74.2 |  | *N*-stearoyl tyrosine | 446.65 | 180.18 |
| *N*-stearoyl glycine | 340.3 | 74.2 |  | *N*-oleoyl tyrosine | 444.63 | 180.18 |
| *N*-oleoyl glycine | 338.3 | 74.2 |  | *N*-linoleoyl tyrosine | 442.61 | 180.18 |
| *N*-linoleoyl glycine | 336.3 | 74.2 |  | *N*-arachidonoyl tyrosine | 466 | 180.18 |
| *N*-arachidonoyl glycine | 360.3 | 74.2 |  | *N*-docosahexaenoyl tyrosine | 490.66 | 180.18 |
| *N*-docosahexaenoyl glycine | 384.3 | 74.2 |  | *N*-acyl valine | [M–H]^-^ | Fragment |
| *N*-acyl leucine | [M–H]^-^ | Fragment |  | *N*-palmitoyl valine | 354.31 | 116.31 |
| *N*-palmitoyl leucine | 368.58 | 130.1 |  | *N*-stearoyl valine | 382.6 | 116.14 |
| *N*-stearoyl leucine | 396.63 | 130.1 |  | *N*-oleoyl valine | 380.59 | 116.14 |
| *N*-oleoyl leucine | 394.61 | 130.1 |  | *N*-linoleoyl valine | 378.58 | 116.14 |
| *N*-linoleoyl leucine | 392.6 | 130.1 |  | *N*-docosahexaenoyl valine | 426.62 | 116.14 |
| *N*-docosahexaenoyl leucine | 440.64 | 130.1 |  | Free Fatty Acids | [M–H]^-^ | Fragment |
| *N*-acyl methionine | [M–H]^-^ | Fragment |  | Oleic acid | 281.5 | 263 |
| *N*-palmitoyl methionine | 386.62 | 148.2 |  | Linoleic acid | 279.5 | 261 |
| *N*-stearoyl methionine | 414.64 | 148.2 |  | Arachidonic acid | 303.5 | 285 |
| *N*-oleoyl methionine | 412.65 | 148.2 |  | PhosphoLEA | [M–H]^-^ | Fragment |
| *N*-linoleoyl methionine | 410.64 | 148.2 |  | phosphoLEA | 403.5 | 58.5 |
| *N*-arachidonoyl methionine | 434.66 | 148.2 |  | 2-acyl glycerol | [M+H]^+^ | Fragment |
| *N*-docosahexaenoyl methionine | 458.68 | 148.2 |  | 2-arachidonoyl glycerol | 379.3 | 287.5 |
| *N*-acyl phenylalanine | [M–H]^-^ | Fragment |  | 2-linoleoyl glycerol | 355.5 | 245 |
| *N*-palmitoyl phenylalanine | 402.59 | 164.1 |  | 2-oleoyl glycerol | 357.5 | 265.2 |
| *N*-stearoyl phenylalanine | 430.65 | 164.1 |  | 2-palmitoyl glycerol | 331.5 | 239.5 |
| *N*-oleoyl phenylalanine | 428.63 | 164.1 |  | Prostaglandins | [M–H]^-^ | Fragment |
| *N*-linoleoyl phenylalanine | 426.61 | 164.1 |  | PGE_2_ | 351.2 | 315 |
| *N*-arachidonoyl phenylalanine | 450.64 | 164.1 |  | PGF_2α_ | 353.3 | 309.2 |
| *N*-docosahexaenoyl phenylalanine | 474.66 | 164.1 |  | 6-ketoPGF_1α_ | 369.3 | 206.9 |
| CP 55,940 | [M+H]^+^ | Fragment |  |  |  |  |
| CP 55,940 | 377.6 | 121.3 |  |  |  |  |

**Supplemental Figure 2.** Lipids in the HPLC/MS/MS screening library with parent and fragment ion masses. Lipids are grouped by amide family. Negative ionization mode, resulting in a [M – H]^-^ parent ion, is used for all methods except the *N*-acyl ethanolamine, 2-acyl glycerol and CP 55,940 methods, which uses positive ionization and generates a [M + H]^+^ parent ion.

**Supplemental Figure 3.** Example overlaid chromatogram comparing CP 55,940 peak from an adult CD1 female hippocampus sample to a 30 fmol CP 55,940 Standard Peak

| Significant decrease relative to vehicle (p≤.05) | ↓ |
| --- | --- |
| Trending decrease relative to vehicle (.05<p≤.10) | ↓ |
| Trending increase relative to vehicle (.05<p≤.10) | ↑ |
| Significant increase relative to vehicle (p≤.05) | ↑ |

| ↑↑↑↑↑ | 10 or more times higher than vehicle |
| --- | --- |
| ↑↑↑↑ | 3-9.99 times higher than vehicle |
| ↑↑↑ | 2-2.99 times higher than vehicle |
| ↑↑ | 1.50-1.99 times higher than vehicle |
| ↑ | 1-1.49 times higher than vehicle |
| ↓ | 1-1.49 times lower than vehicle |
| ↓↓ | 1.50-1.99 times lower than vehicle |
| ↓↓↓ | 2-2.99 times lower than vehicle |
| ↓↓↓↓ | 3-9.99 times lower than vehicle |
| ↓↓↓↓↓ | 10 or more times lower than vehicle |

**Supplemental Figure 4.** Key for interpreting figures showing effects of CP 55,940 on a lipid’s concentration within a brain region at a specific developmental time point. The top part shows the color coding, which is determined by whether there was a significant or trending increase or decrease in a lipid’s concentration i.e. the direction of change. The bottom part shows the key for interpreting the magnitude of those changes. To determine the magnitude change, the mean level of a lipid in a specific region of the CP mice was divided by that same lipid’s mean level in the corresponding vehicle brain area. For example, the mean level of PGE_2_ in the HIPP of adult mice was 3.37x10^-10^ moles per gram in the CP group and 2.85x10^-10^ moles per gram in the vehicle group. 3.37x10^-10^ divided by 2.85x10^-10^ equals 1.18, assigning it 1 up arrow because the magnitude of change was between 1 and 1.5 times higher than vehicle. For decreases the process was very similar: the mean level in the CP group was divided by the mean level in the vehicle; however, the reciprocal of the decimal was taken to express a fold decrease (if the level in the CP mouse is ½ of the vehicle level then that is a 2-fold decrease).

| **Lipid Species** | **STR** | **HIPP** | **CER** | **THAL** | **CTX** | **HYP** | **MID** | **STEM** |
| --- | --- | --- | --- | --- | --- | --- | --- | --- |
| ***N*-acyl alanine** |  |  |  |  |  |  |  |  |
| *N*-palmitoyl alanine |  | ↓ |  |  |  |  |  |  |
| *N*-stearoyl alanine | ↓ | ↓ |  |  |  |  |  |  |
| *N*-oleoyl alanine | ↓ | ↓ |  | ↓ | ↓ | ↓ |  |  |
| *N*-linoleoyl alanine |  | ↓ |  |  |  | ↓ |  |  |
| *N*-arachidonoyl alanine | ↓ | ↓ | ↓ | ↓ | ↓ | ↓↓ | ↓ |  |
| *N*-docosahexaenoyl alanine | ↓ | ↓ | ↓ | ↓ |  | BAL | ↓ |  |
| ***N*-acyl ethanolamine** |  |  |  |  |  |  |  |  |
| *N*-palmitoyl ethanolamine | ↓ | ↓ |  |  |  | ↓↓ |  |  |
| *N*-stearoyl ethanolamine |  |  |  |  |  | ↓ |  |  |
| *N*-oleoyl ethanolamine | ↓↓ | ↓ | ↓ |  |  | ↓↓ | ↓ |  |
| *N*-linoleoyl ethanolamine |  |  |  | ↑ |  |  | ↑ |  |
| *N*-arachidonoyl ethanolamine |  | ↓ |  | ↑ |  | ↓ |  |  |
| *N*-docosahexaenoyl ethanolamine | ↓ | ↓ |  |  |  | ↓↓ |  |  |
| ***N*-acyl GABA** |  |  |  |  |  |  |  |  |
| *N*-palmitoyl GABA |  |  |  | ↑ |  |  |  |  |
| *N*-stearoyl GABA |  | ↓ |  |  |  |  |  |  |
| *N*-oleoyl GABA | ↓ | ↓↓ | ↓ | ↓ | ↓ | ↓ | ↓ |  |
| *N*-linoleoyl GABA | BAL | ↓↓ |  |  | ↓ | BAL |  |  |
| *N*-arachidonoyl GABA | ↓↓ | ↓↓ | ↓ | ↓ | ↓ | ↓ | ↓ | ↓ |
| *N*-docosahexaenoyl GABA | ↓ | ↓↓ | ↓ | ↓ | ↓ | ↓ | ↓ |  |
| ***N*-acyl glycine** |  |  |  |  |  |  |  |  |
| *N*-stearoyl glycine |  | ↓ |  |  |  |  |  |  |
| *N*-oleoyl glycine | ↓ | ↓ |  | ↓ | ↓ |  |  |  |
| *N*-linoleoyl glycine |  |  | ↓ |  | ↓ |  |  |  |
| *N*-arachidonoyl glycine | ↓ | ↓ | ↓ | ↓ | ↓ | ↓ | ↓ |  |
| *N*-docosahexaenoyl glycine |  | ↓ | ↓ | ↓ | ↓ |  | ↓ |  |
| ***N*-acyl leucine** |  |  |  |  |  |  |  |  |
| *N*-palmitoyl leucine | ↑ | ↑ | ↑↑ | ↑ | ↑↑ | ↑ | ↑ | ↑ |
| *N*-stearoyl leucine | ↓ |  |  |  |  |  |  |  |
| *N*-oleoyl leucine |  |  | ↑ | ↑ | ↑ |  |  | ↑ |
| *N*-linoleoyl leucine | BAL |  | ↑ |  |  | BAL |  |  |
| *N*-docosahexaenoyl leucine | ↑ | ↑ | ↑ | ↑↑ | ↑↑ | BAL | ↑ | ↑ |
| ***N*-acyl methionine** |  |  |  |  |  |  |  |  |
| *N*-stearoyl methionine |  |  |  |  |  | BAL | ↑ |  |
| *N*-oleoyl methionine |  | ↓ |  |  | ↓ | BAL |  |  |
| *N*-arachidonoyl methionine | BAL | BAL | ↓↓ | BAL | ↓↓ | BAL |  |  |
| *N*-docosahexaenoyl methionine | BAL | BAL |  | BAL | BAL | BAL | ↓ | BAL |
| ***N*-acyl phenylalanine** |  |  |  |  |  |  |  |  |
| *N*-palmitoyl phenylalanine |  | ↑ | ↑ | ↑ |  |  | ↑ | ↑ |
| *N*-oleoyl phenylalanine |  |  | ↑ |  |  |  | ↑ |  |
| *N*-linoleoyl phenylalanine | BAL |  | ↑ | BAL |  | BAL |  | BAL |
| *N*-arachidonoyl phenylalanine | ↓ | ↓ |  | ↑ | ↓ | ↓ |  |  |
| *N*-docosahexaenoyl phenylalanine |  |  |  | ↑ |  | BAL |  |  |
| ***N*-acyl proline** |  |  |  |  |  |  |  |  |
| *N*-palmitoyl proline |  |  | ↓ |  |  | BAL |  | ↑ |
| *N-*oleoyl proline | BAL | BAL |  | BAL | BAL | BAL | BAL | ↑ |
| ***N*-acyl serine** |  |  |  |  |  |  |  |  |
| *N*-palmitoyl serine |  |  | ↓ |  | ↓ |  |  |  |
| *N*-stearoyl serine | ↓ |  |  | ↓ | ↓ |  |  |  |
| *N*-arachidonoyl serine | ↓↓ | ↓↓↓ | ↓ | ↓ | ↓ | BAL | ↓ |  |
| *N*-docosahexaenoyl serine | ↓ | ↓ | ↓ |  | ↓↓ | BAL | ↓ |  |
| ***N*-acyl taurine** |  |  |  |  |  |  |  |  |
| *N*-arachidonoyl taurine | ↓ | ↓ | ↓ | ↓ | ↓ | ↓ | ↓ | ↓ |
| ***N*-acyl tryptophan** |  |  |  |  |  |  |  |  |
| *N*-palmitoyl tryptophan |  |  |  |  |  | BAL |  | ↓ |
| ***N*-acyl tyrosine** |  |  |  |  |  |  |  |  |
| *N*-palmitoyl tyrosine |  | ↑ | ↑ | ↑ | ↑ |  |  |  |
| *N*-stearoyl tyrosine | BAL |  |  | ↓ | ↑ | BAL | ↓ |  |
| *N*-arachidonoyl tyrosine | ↓ |  | ↓ |  | ↓ | BAL | ↓ |  |
| *N*-docosahexaenoyl tyrosine | BAL | BAL | ↓ |  |  | BAL | ↓ |  |
| ***N*-acyl valine** |  |  |  |  |  |  |  |  |
| *N*-palmitoyl valine | ↑↑ | ↑ | ↑↑ | ↑↑ | ↑↑ |  | ↑↑ | ↑ |
| *N*- stearoyl valine | ↑ | ↑ | BAL | ↑ | ↑ |  | ↑ | ↑ |
| *N*-oleoyl valine |  | ↑ | BAL | ↑ | ↑ |  | ↑ | ↑ |
| *N*-docosahexaenoyl valine | BAL | BAL | BAL |  | ↑ | BAL | ↑↑ | ↑ |
| **2-acyl glycerol** |  |  |  |  |  |  |  |  |
| 2-palmitoyl glycerol |  |  |  |  | ↑↑ | ↑ |  |  |
| 2-oleoyl glycerol | ↓ | ↓ |  |  |  | ↓↓ |  |  |
| 2-linoleoyl glycerol | ↓↓ | ↓↓ | ↓ | ↓ |  | ↓↓ | ↓ | ↓ |
| 2-arachidonoyl glycerol | ↓ | ↓ | ↓ |  |  | ↓↓↓ |  | ↓ |
| **Free Fatty Acids** |  |  |  |  |  |  |  |  |
| Oleic acid |  | ↓ |  |  |  | ↓ |  |  |
| Linoleic acid |  | ↓ |  |  |  | ↓ |  |  |
| Arachidonic acid |  | ↓ | ↓ |  |  | ↓ | ↓ |  |
| **phosphoLEA** |  |  |  |  |  |  |  |  |
| phosphoLEA |  |  |  | ↑ | ↑↑ | ↑ | ↑ |  |
| **Prostaglandins** |  |  |  |  |  |  |  |  |
| PGE_2_ | ↑ |  |  | ↑ |  | ↓ | ↑ | ↑ |
| PGF_2α_ | ↑ | ↑ |  |  | ↑ | ↑ | ↑ |  |
| 6-ketoPGF_1α_ |  | ↑ | ↓↓ | ↓ | ↓ |  | ↓ |  |

**Supplemental Figure 5.** Lipids affected by acute 3 mg/kg CP 55,940 in at least one area of the PND 35 CD1 female mouse brain. See Supplemental Figure 4 for a key on interpreting this figure.

| **Lipid Species** | **STR** | **HIPP** | **CER** | **THAL** | **CTX** | **HYP** | **MID** | **STEM** |
| --- | --- | --- | --- | --- | --- | --- | --- | --- |
| ***N*-acyl alanine** |  |  |  |  |  |  |  |  |
| *N*-palmitoyl alanine |  |  |  |  |  |  | ↓ |  |
| *N*-arachidonoyl alanine | BAL | ↓ | BAL | BAL | ↓ | BAL | ↓↓ |  |
| ***N*-acyl ethanolamine** |  |  |  |  |  |  |  |  |
| *N*-palmitoyl ethanolamine |  |  |  |  |  |  | ↓ |  |
| *N*-oleoyl ethanolamine |  |  | ↓ |  | ↓↓ |  | ↓ |  |
| *N*-linoleoyl ethanolamine |  | ↓↓ | ↓ |  | ↓ |  | ↓ |  |
| *N*-arachidonoyl ethanolamine |  | ↓↓ | ↓ | ↓ | ↓ |  | ↓ |  |
| *N*-docosahexaenoyl ethanolamine |  | ↓ |  |  | ↓ |  | ↓ |  |
| ***N*-acyl GABA** |  |  |  |  |  |  |  |  |
| *N*-oleoyl GABA |  |  | ↓ |  |  |  |  |  |
| *N*-linoleoyl GABA | BAL | BAL |  | ↓↓ |  | BAL |  |  |
| *N*-arachidonoyl GABA | ↓ |  | ↓ | ↓ | ↓ |  | ↓ | ↓ |
| *N*-docosahexaenoyl GABA | ↓ | BAL |  | ↓ |  | BAL | ↓ |  |
| ***N*-acyl glycine** |  |  |  |  |  |  |  |  |
| *N*-arachidonoyl glycine | ↓↓ | ↓↓ | ↓ | ↓↓ |  |  | ↓↓ |  |
| *N*-docosahexaenoyl glycine | ↓↓ |  |  |  |  |  |  |  |
| ***N*-acyl leucine** |  |  |  |  |  |  |  |  |
| *N*-palmitoyl leucine | ↑ |  | ↑↑ | ↑ | ↑ | ↑ | ↑ | ↑ |
| *N*-oleoyl leucine |  | ↓ |  |  |  |  |  |  |
| *N*-docosahexaenoyl leucine |  | BAL |  |  |  | BAL | ↑ |  |
| ***N*-acyl methionine** |  |  |  |  |  |  |  |  |
| *N*-palmitoyl methionine |  |  |  |  | ↑ |  |  |  |
| *N*-stearoyl methionine |  | BAL |  |  |  |  | ↓ |  |
| *N*-oleoyl methionine |  | BAL |  |  |  | BAL | ↓↓ | ↓ |
| *N*-arachidonoyl methionine | BAL | BAL | BAL | BAL | BAL | BAL | ↓↓ | BAL |
| ***N*-acyl phenylalanine** |  |  |  |  |  |  |  |  |
| *N*-palmitoyl phenylalanine |  |  |  |  | ↑ |  |  |  |
| *N*-stearoyl phenylalanine |  |  |  |  | ↑ |  | ↓ |  |
| *N*-oleoyl phenylalanine |  |  | ↑ |  |  |  |  |  |
| *N*-arachidonoyl phenylalanine | ↓ | BAL | ↓↓ | ↓↓ | ↓ | ↓ | ↓ | ↑ |
| *N*-docosahexaenoyl phenylalanine |  | BAL | ↓ | BAL |  | BAL |  |  |
| ***N*-acyl serine** |  |  |  |  |  |  |  |  |
| *N*-palmitoyl serine |  |  |  | ↓ |  |  | ↓ |  |
| *N*-stearoyl serine |  |  |  | ↓ |  |  |  |  |
| *N*-oleoyl serine |  | ↑ |  |  | ↑ |  |  |  |
| *N*-linoleoyl serine |  | ↑ |  |  |  |  |  |  |
| *N*-arachidonoyl serine | BAL | ↓↓ | ↓ |  |  | BAL |  | ↓ |
| ***N*-acyl taurine** |  |  |  |  |  |  |  |  |
| *N*-arachidonoyl taurine | ↓ | ↓ | ↓ | ↓ | ↓ |  |  | ↓ |
| ***N*-acyl tyrosine** |  |  |  |  |  |  |  |  |
| *N*-palmitoyl tyrosine | ↑ |  |  |  | ↑ |  |  | ↑ |
| *N*-arachidonoyl tyrosine | BAL | ↑ | ↓ | ↓ |  | BAL | ↓ | ↓ |
| ***N*-acyl valine** |  |  |  |  |  |  |  |  |
| *N*-palmitoyl valine | ↑↑ | BAL | ↑ |  | ↑ |  |  |  |
| *N*- stearoyl valine | BAL | BAL |  |  | ↑ | BAL |  |  |
| **2-acyl -glycerol** |  |  |  |  |  |  |  |  |
| 2-palmitoyl glycerol |  |  | ↓ |  |  |  |  | ↓ |
| 2-oleoyl glycerol |  |  | ↓ | ↓ | ↓ |  | ↓ | ↓ |
| 2-linoleoyl glycerol | ↓ | ↓ | ↓↓ | ↓ | ↓ |  | ↓ | ↓ |
| 2-arachidonoyl glycerol | ↓ |  |  | ↓ |  | ↓ |  |  |
| **Free Fatty Acids** |  |  |  |  |  |  |  |  |
| Oleic Acid | ↓ |  | ↓ | ↓ |  |  | ↓ | ↓ |
| Linoleic acid | ↓ | ↓ | ↓ | ↓ | ↓↓ |  | ↓ | ↓ |
| Arachidonic acid | ↓ |  | ↓ | ↓ | ↓ |  |  |  |
| **Prostaglandins** |  |  |  |  |  |  |  |  |
| PGE_2_ |  | ↑ | ↓ | ↓ | ↓ |  | ↓ | ↓ |
| PGF_2α_ | ↑ | ↑ | ↓ | ↓ |  | ↑ | ↓ | ↓ |
| 6-ketoPGF_1α_ | BAL | ↑ | ↑ | ↑↑ |  |  | ↓ | ↓ |

**Supplemental Figure 6.** Lipids with concentrations affected by acute 3 mg/kg CP 55,940 in at least one area of the PND 50 CD1 female mouse brain. See Supplemental Figure 4 for a key on interpreting this figure.

| **Lipid Species** | **STR** | **HIPP** | **CER** | **THAL** | **CTX** | **HYP** | **MID** | **STEM** |
| --- | --- | --- | --- | --- | --- | --- | --- | --- |
| ***N*-acyl alanine** |  |  |  |  |  |  |  |  |
| *N*-arachidonoyl alanine |  | ↓ | BAL | ↓↓ |  | BAL |  |  |
| ***N*-acyl ethanolamine** |  |  |  |  |  |  |  |  |
| *N*-palmitoyl ethanolamine |  |  |  | ↓ |  |  | ↓ |  |
| *N*-stearoyl ethanolamine |  |  |  |  |  |  | ↑ |  |
| *N*-oleoyl ethanolamine | ↓↓ |  |  | ↓ |  |  |  | ↓ |
| *N*-linoleoyl ethanolamine | ↓ |  | ↓↓ |  |  |  |  |  |
| *N*-arachidonoyl ethanolamine | ↓↓ |  | ↓↓ | ↓ |  |  | ↓ |  |
| *N*-docosahexaenoyl ethanolamine | ↓ |  | ↓ |  |  |  |  |  |
| ***N*-acyl GABA** |  |  |  |  |  |  |  |  |
| *N*-palmitoyl GABA |  |  |  | ↑ |  |  |  |  |
| *N*-oleoyl GABA |  | ↓ |  |  |  | BAL |  |  |
| *N*-linoleoyl GABA | BAL | BAL | ↓ |  | ↓ | BAL | BAL |  |
| *N*-arachidonoyl GABA |  | ↓ | ↓↓ | ↓ |  | ↓ |  |  |
| *N*-docosahexaenoyl GABA | BAL | ↓ | ↓ |  |  | BAL |  |  |
| ***N*-acyl glycine** |  |  |  |  |  |  |  |  |
| *N*-linoleoyl glycine | BAL |  |  |  |  | BAL | ↓↓ |  |
| *N*-arachidonoyl glycine |  | ↓ |  | ↓↓ |  |  |  |  |
| ***N*-acyl leucine** |  |  |  |  |  |  |  |  |
| *N*-palmitoyl leucine |  |  |  | ↑ |  |  |  | ↑ |
| *N*-oleoyl leucine | ↑ |  |  |  |  | BAL |  |  |
| ***N*-acyl methionine** |  |  |  |  |  |  |  |  |
| *N*-palmitoyl methionine | BAL |  |  | ↑ |  | BAL |  |  |
| ***N*-acyl phenylalanine** |  |  |  |  |  |  |  |  |
| *N*-palmitoyl phenylalanine |  |  |  |  |  | ↑↑ |  |  |
| *N*-stearoyl phenylalanine |  |  |  |  | ↓↓ |  |  |  |
| *N*-oleoyl phenylalanine |  | ↓ |  |  |  |  |  |  |
| *N*-arachidonoyl phenylalanine |  | ↓↓ | ↓↓↓ |  |  |  |  |  |
| ***N*-acyl serine** |  |  |  |  |  |  |  |  |
| *N*-palmitoyl serine | ↓ |  |  |  |  | BAL | BAL |  |
| *N*-stearoyl serine | BAL | ↓ |  |  |  |  |  |  |
| *N*-oleoyl serine |  |  | ↓ |  |  |  |  |  |
| *N*-linoleoyl serine |  |  |  |  |  |  | ↓ |  |
| *N*-arachidonoyl serine | BAL | BAL | BAL | BAL | BAL | BAL | ↓↓ | BAL |
| ***N*-acyl taurine** |  |  |  |  |  |  |  |  |
| *N*-arachidonoyl taurine |  |  | ↓ |  | ↓ | ↑ |  | ↓ |
| ***N*-acyl tyrosine** |  |  |  |  |  |  |  |  |
| *N*-palmitoyl tyrosine |  |  | ↓ |  |  |  |  |  |
| *N*-arachidonoyl tyrosine | BAL | BAL | BAL |  |  | BAL |  | ↓↓ |
| ***N*-acyl valine** |  |  |  |  |  |  |  |  |
| *N*-palmitoyl valine | BAL |  |  | ↑↑ | ↑ |  |  | ↑ |
| *N*-oleoyl valine | BAL | BAL | ↑↑↑ | BAL |  | BAL |  |  |
| **2-acyl glycerol** |  |  |  |  |  |  |  |  |
| 2-palmitoyl glycerol |  |  |  |  |  |  |  | ↓ |
| 2-oleoyl glycerol |  |  | ↓ |  |  |  | ↓ | ↓ |
| 2-linoleoyl glycerol |  | ↓ | ↓↓ | ↓ | ↓ |  | ↓ | ↓ |
| 2-arachidonoyl glycerol | ↓ |  | ↓ | ↓ |  |  | ↓ | ↓ |
| **Free Fatty Acids** |  |  |  |  |  |  |  |  |
| Oleic Acid |  |  | ↓↓ | ↓ |  |  |  | ↓ |
| Linoleic acid |  |  | ↓↓ |  |  |  |  | ↓ |
| Arachidonic acid | ↓ |  | ↓ | ↓ | ↓ |  | ↓ | ↓ |
| **phosphoLEA** |  |  |  |  |  |  |  |  |
| phosphoLEA |  | ↑ | ↓ |  |  | ↓ | ↓ |  |
| **Prostaglandins** |  |  |  |  |  |  |  |  |
| PGE_2_ |  | ↑ | ↓ |  |  | ↑ | ↑ | ↓ |
| PGF_2α_ |  | ↑ | ↓ |  | ↓ | ↓ |  |  |
| 6-ketoPGF_1α_ | BAL |  | ↓ | ↑ | ↓ | ↓↓ | ↑ |  |

**Supplemental Figure 7.** Lipids with concentrations affected by acute 3 mg/kg CP 55,940 in at least one area of the adult CD1 female mouse brain. See Supplemental Figure 4 for a key.

**1.2 Supplemental Tables**

**Supplemental Table 1.** Lipid levels in the striatum of WT female PND 35 mice treated with Vehicle or 3 mg/kg CP 55,940. For lipids that were detected, the mean level of that lipid is shown in that particular brain region for Vehicle or CP 55,940-treated mice, along with the standard deviation (SD) and standard error (SE). Lipids that were Present in Some Samples at Random are denoted by the acronym “PISSR” and lipids whose levels did not reach detection are denoted “BDL” (below detection limit).

|  | Striatum | | | | | | |
| --- | --- | --- | --- | --- | --- | --- | --- |
|  | Vehicle | | | 3mg/kg CP 55,940 | | | |
| ***N*-acyl alanine** | Mean | Std Dev | Std Error | | Mean | Std Dev | Std Error |
| *N*-palmitoyl alanine | 1.51E-11 | 2.73E-12 | 9.11E-13 | | 1.47E-11 | 2.78E-12 | 9.27E-13 |
| *N*-stearoyl alanine | 1.26E-11 | 1.25E-12 | 4.15E-13 | | 1.12E-11 | 1.55E-12 | 5.16E-13 |
| *N*-oleoyl alanine | 7.52E-12 | 9.34E-13 | 3.11E-13 | | 6.67E-12 | 9.58E-13 | 3.19E-13 |
| *N*-linoleoyl alanine | 1.8E-12 | 2.78E-13 | 9.27E-14 | | 1.57E-12 | 4.79E-13 | 1.6E-13 |
| *N*-arachidonoyl alanine | 1.03E-11 | 1.37E-12 | 4.56E-13 | | 7.95E-12 | 1.26E-12 | 4.2E-13 |
| *N*-docosahexaenoyl alanine | 4.19E-12 | 7.72E-13 | 2.57E-13 | | 3E-12 | 6.68E-13 | 2.23E-13 |
| ***N*-acyl dopamine** |  |  |  | |  |  |  |
| *N*-oleoyl dopamine | PISSR |  |  | | PISSR |  |  |
| *N*-arachidonoyl dopamine | PISSR |  |  | | PISSR |  |  |
| ***N*-acyl ethanolamine** |  |  |  | |  |  |  |
| *N*-palmitoyl ethanolamine | 4.42E-11 | 1.13E-11 | 3.99E-12 | | 3.18E-11 | 6.39E-12 | 2.13E-12 |
| *N*-stearoyl ethanolamine | 1.14E-11 | 2.17E-12 | 7.67E-13 | | 9E-12 | 3.54E-12 | 1.18E-12 |
| *N*-oleoyl ethanolamine | 1.01E-10 | 2.45E-11 | 8.66E-12 | | 6.1E-11 | 8.96E-12 | 2.99E-12 |
| *N*-linoleoyl ethanolamine | 2.9E-11 | 7.94E-12 | 2.65E-12 | | 3.02E-11 | 3.78E-12 | 1.26E-12 |
| *N*-arachidonoyl ethanolamine | 1.29E-11 | 2.99E-12 | 9.97E-13 | | 1.25E-11 | 2.21E-12 | 7.36E-13 |
| *N*-docosahexaenoyl ethanolamine | 2.16E-11 | 3.53E-12 | 1.18E-12 | | 1.6E-11 | 1.48E-12 | 4.94E-13 |
| ***N*-acyl GABA** |  |  |  | |  |  |  |
| *N*-palmitoyl GABA | 3.72E-12 | 4.63E-13 | 1.54E-13 | | 3.96E-12 | 8.16E-13 | 2.72E-13 |
| *N*-stearoyl GABA | 3.7E-12 | 3.35E-13 | 1.12E-13 | | 3.5E-12 | 5.42E-13 | 1.81E-13 |
| *N*-oleoyl GABA | 1.91E-12 | 1.7E-13 | 5.68E-14 | | 1.45E-12 | 2.25E-13 | 7.49E-14 |
| *N*-linoleoyl GABA | PISSR |  |  | | PISSR |  |  |
| *N*-arachidonoyl GABA | 1.11E-11 | 9.68E-13 | 3.23E-13 | | 7.34E-12 | 4.89E-13 | 1.63E-13 |
| *N*-docosahexaenoyl GABA | 1.12E-12 | 7.99E-14 | 2.66E-14 | | 7.6E-13 | 8.78E-14 | 2.93E-14 |
| ***N*-acyl glycine** |  |  |  | |  |  |  |
| *N*-palmitoyl glycine | 2.99E-11 | 3.02E-12 | 1.01E-12 | | 2.93E-11 | 4.44E-12 | 1.48E-12 |
| *N*-stearoyl glycine | 1.73E-11 | 1.21E-12 | 4.05E-13 | | 1.64E-11 | 1.94E-12 | 6.47E-13 |
| *N*-oleoyl glycine | 1.25E-11 | 1.34E-12 | 4.46E-13 | | 1.1E-11 | 1.36E-12 | 4.53E-13 |
| *N*-linoleoyl glycine | 2.31E-12 | 4.51E-13 | 1.5E-13 | | 1.9E-12 | 7.39E-13 | 2.46E-13 |
| *N*-arachidonoyl glycine | 2.87E-11 | 2.86E-12 | 9.53E-13 | | 2.49E-11 | 2.41E-12 | 8.04E-13 |
| *N*-docosahexaenoyl glycine | 1.16E-11 | 2.24E-12 | 7.47E-13 | | 1.04E-11 | 1.38E-12 | 4.6E-13 |
| ***N*-acyl leucine** |  |  |  | |  |  |  |
| *N*-palmitoyl leucine | 2.17E-12 | 3.23E-13 | 1.08E-13 | | 2.54E-12 | 4.33E-13 | 1.44E-13 |
| *N*-stearoyl leucine | 1.24E-12 | 2.1E-13 | 7.01E-14 | | 9.98E-13 | 2.31E-13 | 7.71E-14 |
| *N*-oleoyl leucine | 1.17E-12 | 1.16E-13 | 3.87E-14 | | 1.08E-12 | 1.63E-13 | 5.45E-14 |
| *N*-linoleoyl leucine | PISSR |  |  | | PISSR |  |  |
| *N*-docosahexaenoyl leucine | 3.66E-13 | 5.59E-14 | 1.86E-14 | | 5.34E-13 | 6.53E-14 | 2.18E-14 |
| ***N*-acyl methionine** |  |  |  | |  |  |  |
| *N*-palmitoyl methionine | 1.67E-12 | 5.2E-13 | 1.73E-13 | | 1.86E-12 | 3.5E-13 | 1.17E-13 |
| *N*-stearoyl methionine | 1.34E-12 | 3.04E-13 | 1.01E-13 | | 1.31E-12 | 4.44E-13 | 1.48E-13 |
| *N*-oleoyl methionine | 8.41E-13 | 3.18E-13 | 1.06E-13 | | 9.72E-13 | 2.19E-13 | 7.3E-14 |
| *N*-linoleoyl methionine | PISSR |  |  | | PISSR |  |  |
| *N*-arachidonoyl methionine | PISSR |  |  | | PISSR |  |  |
| *N*-docosahexaenoyl methionine | PISSR |  |  | | PISSR |  |  |
| ***N*-acyl phenylalanine** |  |  |  | |  |  |  |
| *N*-palmitoyl phenylalanine | 1.33E-12 | 1.31E-13 | 4.35E-14 | | 1.48E-12 | 2.67E-13 | 8.91E-14 |
| *N*-stearoyl phenylalanine | 1.44E-12 | 2.39E-13 | 7.96E-14 | | 1.28E-12 | 2.26E-13 | 7.55E-14 |
| *N*-oleoyl phenylalanine | 7.39E-13 | 1.17E-13 | 3.89E-14 | | 6.72E-13 | 1.34E-13 | 4.47E-14 |
| *N*-linoleoyl phenylalanine | PISSR |  |  | | PISSR |  |  |
| *N*-arachidonoyl phenylalanine | 6.1E-13 | 1.01E-13 | 3.35E-14 | | 5.08E-13 | 7.94E-14 | 2.65E-14 |
| *N*-docosahexaenoyl phenylalanine | 6.2E-13 | 1.34E-13 | 4.45E-14 | | 5.57E-13 | 1.75E-13 | 5.84E-14 |

**Supplemental Table 1: Continued**

|  | Striatum | | | | | |
| --- | --- | --- | --- | --- | --- | --- |
|  | Vehicle | | | 3mg/kg CP 55,940 | | |
| ***N*-acyl proline** | Mean | Std Dev | Std Error | Mean | Std Dev | Std Error |
| *N*-palmitoyl proline | 1.12E-12 | 3.29E-13 | 1.1E-13 | 1.03E-12 | 2.81E-13 | 9.36E-14 |
| *N*-stearoyl proline | PISSR |  |  | PISSR |  |  |
| *N-*oleoyl proline | PISSR |  |  | PISSR |  |  |
| *N*-linoleoyl proline | PISSR |  |  | PISSR |  |  |
| *N*-arachidonoyl proline | PISSR |  |  | PISSR |  |  |
| *N*-docosahexaenoyl proline | BDL |  |  | BDL |  |  |
| ***N*-acyl serine** |  |  |  |  |  |  |
| *N*-palmitoyl serine | 3.64E-11 | 3.97E-12 | 1.32E-12 | 3.33E-11 | 7.18E-12 | 2.39E-12 |
| *N*-stearoyl serine | 2.19E-11 | 1.86E-12 | 6.2E-13 | 1.89E-11 | 2.33E-12 | 7.76E-13 |
| *N*-oleoyl serine | 2.49E-10 | 2.67E-11 | 8.89E-12 | 2.35E-10 | 2.89E-11 | 9.63E-12 |
| *N*-linoleoyl serine | 4.84E-11 | 5.37E-12 | 1.79E-12 | 4.58E-11 | 5.21E-12 | 1.74E-12 |
| *N*-arachidonoyl serine | 3.37E-12 | 7.92E-13 | 2.64E-13 | 2.14E-12 | 5.13E-13 | 1.71E-13 |
| *N*-docosahexaenoyl serine | 1.48E-11 | 2.98E-12 | 9.94E-13 | 9.97E-12 | 1.91E-12 | 6.38E-13 |
| ***N*-acyl taurine** |  |  |  |  |  |  |
| *N*-arachidonoyl taurine | 5.87E-11 | 4.79E-12 | 1.6E-12 | 4.32E-11 | 5.93E-12 | 1.98E-12 |
| ***N*-acyl tryptophan** |  |  |  |  |  |  |
| *N*-palmitoyl tryptophan | 2.28E-13 | 4.78E-14 | 1.59E-14 | 2.09E-13 | 1.05E-13 | 3.51E-14 |
| *N*-stearoyl tryptophan | 6.21E-13 | 1.37E-13 | 4.58E-14 | 5.77E-13 | 2E-13 | 6.66E-14 |
| *N*-oleoyl tryptophan | PISSR |  |  | PISSR |  |  |
| *N*-linoleoyl tryptophan | BDL |  |  | BDL |  |  |
| *N*-arachidonoyl tryptophan | BDL |  |  | BDL |  |  |
| *N*-docosahexaenoyl tryptophan | BDL |  |  | BDL |  |  |
| ***N*-acyl tyrosine** |  |  |  |  |  |  |
| *N*-palmitoyl tyrosine | 4.06E-13 | 1.15E-13 | 3.82E-14 | 3.84E-13 | 9.14E-14 | 3.05E-14 |
| *N*-stearoyl tyrosine | PISSR |  |  | PISSR |  |  |
| *N-*oleoyl tyrosine | 1.97E-13 | 4.65E-14 | 1.55E-14 | 1.91E-13 | 7.44E-14 | 2.48E-14 |
| *N*-linoleoyl tyrosine | BDL |  |  | BDL |  |  |
| *N*-arachidonoyl tyrosine | 4.15E-13 | 9.85E-14 | 3.28E-14 | 3.18E-13 | 8.74E-14 | 2.91E-14 |
| *N*-docosahexaenoyl tyrosine | PISSR |  |  | PISSR |  |  |
| ***N*-acyl valine** |  |  |  |  |  |  |
| *N*-palmitoyl valine | 5.81E-13 | 8.55E-14 | 2.85E-14 | 9.76E-13 | 3.33E-13 | 1.11E-13 |
| *N*- stearoyl valine | 3.17E-13 | 8.55E-14 | 2.85E-14 | 4.54E-13 | 8.77E-14 | 2.92E-14 |
| *N*-oleoyl valine | 3.15E-13 | 8.98E-14 | 2.99E-14 | 3.53E-13 | 1.44E-13 | 4.8E-14 |
| *N*-nervonoyl valine | BDL |  |  | BDL |  |  |
| *N*-linoleoyl valine | PISSR |  |  | PISSR |  |  |
| *N*-docosahexaenoyl valine | PISSR |  |  | PISSR |  |  |
| **2-acyl glycerols** |  |  |  |  |  |  |
| 2-palmitoyl-*sn*-glycerol | 4.08E-10 | 7.97E-11 | 2.66E-11 | 3.66E-10 | 5.53E-11 | 1.84E-11 |
| 2-oleoyl-*sn*-glycerol | 7.6E-10 | 1.34E-10 | 4.45E-11 | 6.52E-10 | 1.04E-10 | 3.46E-11 |
| 2-linoleoyl-*sn*-glycerol | 1.2E-10 | 2.13E-11 | 7.11E-12 | 6.79E-11 | 1.27E-11 | 4.24E-12 |
| 2-arachidonoyl-*sn*-glycerol | 1.18E-10 | 2.87E-11 | 9.58E-12 | 8.26E-11 | 2.41E-11 | 8.03E-12 |
| **Free Fatty Acids** |  |  |  |  |  |  |
| Oleic acid | 1.24E-09 | 1.22E-10 | 4.05E-11 | 1.18E-09 | 1.44E-10 | 4.82E-11 |
| Linoleic acid | 5.22E-10 | 5.8E-11 | 1.93E-11 | 4.78E-10 | 5.57E-11 | 1.86E-11 |
| Arachidonic acid | 1.96E-09 | 2.02E-10 | 6.74E-11 | 1.87E-09 | 1.73E-10 | 5.77E-11 |
| **PhosphoLEA** |  |  |  |  |  |  |
| PhosphoLEA | 1.92E-11 | 5.61E-12 | 1.87E-12 | 1.91E-11 | 4.54E-12 | 1.51E-12 |
| **Prostaglandins** |  |  |  |  |  |  |
| PGE_2_ | 2.99E-10 | 1.58E-11 | 5.25E-12 | 3.74E-10 | 3.56E-11 | 1.19E-11 |
| PGF_2α_ | 2.16E-10 | 2.36E-11 | 7.86E-12 | 2.8E-10 | 1.89E-11 | 6.29E-12 |
| 6-ketoPGF_1α_ | 5.36E-12 | 1.58E-12 | 5.27E-13 | 5.31E-12 | 1.22E-12 | 4.08E-13 |
| **CBD/CP/THC** |  |  |  |  |  |  |
| Cannabidiol | BDL |  |  | BDL |  |  |
| CP 55,940 | BDL |  |  | 5.74E-11 | 6.13E-12 | 2.04E-12 |
| THC | BDL |  |  | BDL |  |  |
| **THC Metabolites** |  |  |  |  |  |  |
| 11-nor-9-carboxyTHC | BDL |  |  | BDL |  |  |
| 11-OH-THC | BDL |  |  | BDL |  |  |

**Supplemental Table 2.** List of lipids in the striatum significantly affected by 3mg/kg CP 55,940: PND 35

| PND 35 Striatum Significant Differences in One-Way ANOVA | | | | |
| --- | --- | --- | --- | --- |
| Lipid | F | p | Direction (relative to Veh) | Magnitude (x Veh level) |
| *N-*stearoyl alanine | 4.32 | .054 | ↓ | 0.90 |
| *N-*oleoyl alanine | 3.63 | .075 | ↓ | 0.89 |
| *N*-arachidonoyl alanine | 14.43 | .002 | ↓ | 0.77 |
| *N*-docosahexaenoyl alanine | 12.21 | .003 | ↓ | 0.72 |
| *N-*palmitoyl ethanolamine | 7.94 | .013 | ↓ | 0.72 |
| *N*-oleoyl ethanolamine | 21.06 | .000 | ↓ | 0.60 |
| *N*-docosahexaenoyl ethanolamine | 19.18 | .000 | ↓ | 0.74 |
| *N*-oleoyl GABA | 23.86 | .000 | ↓ | 0.76 |
| *N*-arachidonoyl GABA | 109.75 | .000 | ↓ | 0.66 |
| *N*-docosahexaenoyl GABA | 81.01 | .000 | ↓ | 0.68 |
| *N*-oleoyl glycine | 5.58 | .031 | ↓ | 0.88 |
| *N*-arachidonoyl glycine | 9.59 | .007 | ↓ | 0.87 |
| *N*-palmitoyl leucine | 4.20 | .057 | ↑ | 1.17 |
| *N*-stearoyl leucine | 5.55 | .032 | ↓ | 0.81 |
| *N*-docosahexaenoyl leucine | 34.49 | .000 | ↑ | 1.46 |
| *N*-arachidonoyl phenylalanine | 5.72 | .029 | ↓ | 0.83 |
| *N*-stearoyl serine | 9.08 | .008 | ↓ | 0.86 |
| *N*-arachidonoyl serine | 15.24 | .001 | ↓ | 0.64 |
| *N*-docosahexaenoyl serine | 16.37 | .001 | ↓ | 0.68 |
| *N*-arachidonoyl taurine | 37.20 | .000 | ↓ | 0.91 |
| *N*-arachidonoyl tyrosine | 4.95 | .041 | ↓ | 0.77 |
| *N*-palmitoyl valine | 11.86 | .003 | ↑ | 1.68 |
| *N*-stearoyl valine | 11.15 | .004 | ↑ | 1.43 |
| 2-oleoyl glycerol | 3.64 | .074 | ↓ | 0.86 |
| 2-linoleoyl glycerol | 40.34 | .000 | ↓ | 0.57 |
| 2-arachidonoyl glycerol | 8.04 | .012 | ↓ | 0.70 |
| PGE_2_ | 33.75 | .000 | ↑ | 1.25 |
| PGF_2α_ | 40.40 | .000 | ↑ | 1.30 |
| CP 55,940 | 790.01 | .000 | ↑ | infinite |

**Supplemental Table 3.** Lipid levels in the hippocampus of PND 35 WT adolescent female mice treated with Vehicle or 3 mg/kg CP 55,940

|  | Hippocampus | | | | | | |
| --- | --- | --- | --- | --- | --- | --- | --- |
|  | Vehicle | | | 3mg/kg CP 55,940 | | | |
| ***N*-acyl alanine** | Mean | Std Dev | Std Error | | Mean | Std Dev | Std Error |
| *N*-palmitoyl alanine | 2.3E-11 | 3.13E-12 | 1.04E-12 | | 1.96E-11 | 3.94E-12 | 1.31E-12 |
| *N*-stearoyl alanine | 2.21E-11 | 2.3E-12 | 7.66E-13 | | 1.75E-11 | 2E-12 | 6.66E-13 |
| *N*-oleoyl alanine | 1.34E-11 | 1.33E-12 | 4.44E-13 | | 1.01E-11 | 1.14E-12 | 3.81E-13 |
| *N*-linoleoyl alanine | 3.16E-12 | 6.52E-13 | 2.17E-13 | | 2.58E-12 | 6.51E-13 | 2.17E-13 |
| *N*-arachidonoyl alanine | 2.37E-11 | 1.96E-12 | 6.53E-13 | | 1.79E-11 | 2.97E-12 | 9.88E-13 |
| *N*-docosahexaenoyl alanine | 6.75E-12 | 1.13E-12 | 3.77E-13 | | 5.16E-12 | 9.54E-13 | 3.18E-13 |
| ***N*-acyl dopamine** |  |  |  | |  |  |  |
| *N*-oleoyl dopamine | BDL |  |  | | BDL |  |  |
| *N*-arachidonoyl dopamine | BDL |  |  | | BDL |  |  |
| ***N*-acyl ethanolamine** |  |  |  | |  |  |  |
| *N*-palmitoyl ethanolamine | 7.68E-11 | 1.53E-11 | 5.09E-12 | | 6.04E-11 | 1.08E-11 | 3.61E-12 |
| *N*-stearoyl ethanolamine | 1.97E-11 | 5.29E-12 | 1.76E-12 | | 1.72E-11 | 2.47E-12 | 8.24E-13 |
| *N*-oleoyl ethanolamine | 1.92E-10 | 3.79E-11 | 1.26E-11 | | 1.48E-10 | 2.36E-11 | 7.86E-12 |
| *N*-linoleoyl ethanolamine | 7.45E-11 | 1.54E-11 | 5.14E-12 | | 6.76E-11 | 7.3E-12 | 2.43E-12 |
| *N*-arachidonoyl ethanolamine | 5.77E-11 | 1.16E-11 | 3.86E-12 | | 4.75E-11 | 2.67E-12 | 8.91E-13 |
| *N*-docosahexaenoyl ethanolamine | 4.46E-11 | 7.71E-12 | 2.57E-12 | | 3.15E-11 | 4.9E-12 | 1.63E-12 |
| ***N*-acyl GABA** |  |  |  | |  |  |  |
| *N*-palmitoyl GABA | 6.04E-12 | 7.21E-13 | 2.4E-13 | | 5.66E-12 | 9.07E-13 | 3.02E-13 |
| *N*-stearoyl GABA | 7.27E-12 | 1.42E-12 | 4.73E-13 | | 5.59E-12 | 8.8E-13 | 2.93E-13 |
| *N*-oleoyl GABA | 4.87E-12 | 8.84E-13 | 2.95E-13 | | 2.89E-12 | 4.73E-13 | 1.58E-13 |
| *N*-linoleoyl GABA | 9.02E-13 | 1.4E-13 | 4.67E-14 | | 4.99E-13 | 1.09E-13 | 3.64E-14 |
| *N*-arachidonoyl GABA | 3.27E-11 | 4.54E-12 | 1.51E-12 | | 2.17E-11 | 3.32E-12 | 1.11E-12 |
| *N*-docosahexaenoyl GABA | 1.77E-12 | 3.13E-13 | 1.04E-13 | | 9.2E-13 | 9.78E-14 | 3.26E-14 |
| ***N*-acyl glycine** |  |  |  | |  |  |  |
| *N*-palmitoyl glycine | 4.83E-11 | 2.45E-12 | 8.15E-13 | | 4.65E-11 | 5.35E-12 | 1.78E-12 |
| *N*-stearoyl glycine | 2.88E-11 | 2.71E-12 | 9.03E-13 | | 2.5E-11 | 3.69E-12 | 1.23E-12 |
| *N*-oleoyl glycine | 2.42E-11 | 1.64E-12 | 5.46E-13 | | 2.1E-11 | 1.89E-12 | 6.32E-13 |
| *N*-linoleoyl glycine | 6E-12 | 1.01E-12 | 3.35E-13 | | 5.29E-12 | 8.79E-13 | 2.93E-13 |
| *N*-arachidonoyl glycine | 7.77E-11 | 8.85E-12 | 2.95E-12 | | 6.69E-11 | 7.77E-12 | 2.59E-12 |
| *N*-docosahexaenoyl glycine | 2.27E-11 | 3E-12 | 9.99E-13 | | 1.89E-11 | 2.18E-12 | 7.27E-13 |
| ***N*-acyl leucine** |  |  |  | |  |  |  |
| *N*-palmitoyl leucine | 3.2E-12 | 1.99E-13 | 6.64E-14 | | 4.19E-12 | 3.51E-13 | 1.17E-13 |
| *N*-stearoyl leucine | 1.87E-12 | 2.58E-13 | 8.61E-14 | | 1.77E-12 | 2.86E-13 | 9.53E-14 |
| *N*-oleoyl leucine | 1.59E-12 | 1.73E-13 | 5.76E-14 | | 1.71E-12 | 1.88E-13 | 6.26E-14 |
| *N*-linoleoyl leucine | 4.17E-13 | 8.67E-14 | 2.89E-14 | | 3.89E-13 | 7.86E-14 | 2.62E-14 |
| *N*-docosahexaenoyl leucine | 5.43E-13 | 1.49E-13 | 4.97E-14 | | 7.64E-13 | 1.51E-13 | 5.05E-14 |
| ***N*-acyl methionine** |  |  |  | |  |  |  |
| *N*-palmitoyl methionine | PISSR |  |  | | PISSR |  |  |
| *N*-stearoyl methionine | 1.96E-12 | 5.03E-13 | 1.68E-13 | | 1.74E-12 | 5.49E-13 | 1.83E-13 |
| *N*-oleoyl methionine | 1.23E-12 | 4.26E-13 | 1.42E-13 | | 8.45E-13 | 3.85E-13 | 1.28E-13 |
| *N*-linoleoyl methionine | BDL |  |  | | BDL |  |  |
| *N*-arachidonoyl methionine | PISSR |  |  | | PISSR |  |  |
| *N*-docosahexaenoyl methionine | BDL |  |  | | BDL |  |  |
| ***N*-acyl phenylalanine** |  |  |  | |  |  |  |
| *N*-palmitoyl phenylalanine | 2.27E-12 | 2.98E-13 | 9.94E-14 | | 2.85E-12 | 4.12E-13 | 1.37E-13 |
| *N*-stearoyl phenylalanine | 2.66E-12 | 5.07E-13 | 1.69E-13 | | 2.56E-12 | 2.69E-13 | 8.95E-14 |
| *N*-oleoyl phenylalanine | 1.28E-12 | 1.6E-13 | 5.35E-14 | | 1.23E-12 | 1.22E-13 | 4.07E-14 |
| *N*-linoleoyl phenylalanine | 2.49E-13 | 8.79E-14 | 2.93E-14 | | 2.51E-13 | 7.96E-14 | 2.65E-14 |
| *N*-arachidonoyl phenylalanine | 1.59E-12 | 2.6E-13 | 8.66E-14 | | 1.21E-12 | 1.18E-13 | 3.93E-14 |
| *N*-docosahexaenoyl phenylalanine | 1.17E-12 | 1.35E-13 | 4.51E-14 | | 1.25E-12 | 2.39E-13 | 7.97E-14 |

**Supplemental Table 3: Continued**

|  | Hippocampus | | | | | |
| --- | --- | --- | --- | --- | --- | --- |
|  | Vehicle | | | 3mg/kg CP 55,940 | | |
| ***N*-acyl proline** | Mean | Std Dev | Std Error | Mean | Std Dev | Std Error |
| *N*-palmitoyl proline | 1.92E-12 | 2.89E-13 | 9.62E-14 | 1.75E-12 | 3.81E-13 | 1.27E-13 |
| *N*-stearoyl proline | PISSR |  |  | PISSR |  |  |
| *N-*oleoyl proline | PISSR |  |  | PISSR |  |  |
| *N*-linoleoyl proline | BDL |  |  | BDL |  |  |
| *N*-arachidonoyl proline | BDL |  |  | BDL |  |  |
| *N*-docosahexaenoyl proline | BDL |  |  | BDL |  |  |
| ***N*-acyl serine** |  |  |  |  |  |  |
| *N*-palmitoyl serine | 7.19E-11 | 1.22E-11 | 4.08E-12 | 7.46E-11 | 1.56E-11 | 5.2E-12 |
| *N*-stearoyl serine | 3.26E-11 | 2.95E-12 | 9.84E-13 | 3.13E-11 | 3.47E-12 | 1.16E-12 |
| *N*-oleoyl serine | 3.92E-10 | 7.05E-11 | 2.35E-11 | 3.91E-10 | 5.87E-11 | 1.96E-11 |
| *N*-linoleoyl serine | 7.83E-11 | 1.35E-11 | 4.51E-12 | 8.04E-11 | 1.2E-11 | 4E-12 |
| *N*-arachidonoyl serine | 1.77E-11 | 2.58E-12 | 8.6E-13 | 8.58E-12 | 1.3E-12 | 4.34E-13 |
| *N*-docosahexaenoyl serine | 3.27E-11 | 4.8E-12 | 1.6E-12 | 2.42E-11 | 4.78E-12 | 1.59E-12 |
| ***N*-acyl taurine** |  |  |  |  |  |  |
| *N*-arachidonoyl taurine | 2.68E-10 | 2.7E-11 | 9E-12 | 2.18E-10 | 2.99E-11 | 9.96E-12 |
| ***N*-acyl tryptophan** |  |  |  |  |  |  |
| *N*-palmitoyl tryptophan | 4.04E-13 | 8.98E-14 | 2.99E-14 | 4.02E-13 | 4.69E-14 | 1.56E-14 |
| *N*-stearoyl tryptophan | 1.24E-12 | 3.75E-13 | 1.25E-13 | 1.25E-12 | 2.87E-13 | 9.56E-14 |
| *N*-oleoyl tryptophan | PISSR |  |  | PISSR |  |  |
| *N*-linoleoyl tryptophan | BDL |  |  | BDL |  |  |
| *N*-arachidonoyl tryptophan | BDL |  |  | BDL |  |  |
| *N*-docosahexaenoyl tryptophan | BDL |  |  | BDL |  |  |
| ***N*-acyl tyrosine** |  |  |  |  |  |  |
| *N*-palmitoyl tyrosine | 8.64E-13 | 1.89E-13 | 6.3E-14 | 1.11E-12 | 1.46E-13 | 4.86E-14 |
| *N*-stearoyl tyrosine | 1.4E-13 | 3.56E-14 | 1.19E-14 | 1.27E-13 | 2.42E-14 | 8.08E-15 |
| *N-*oleoyl tyrosine | 4.04E-13 | 1.27E-13 | 4.23E-14 | 3.7E-13 | 1.41E-13 | 4.7E-14 |
| *N*-linoleoyl tyrosine | PISSR |  |  | PISSR |  |  |
| *N*-arachidonoyl tyrosine | 9.96E-13 | 4.31E-13 | 1.44E-13 | 7.88E-13 | 2.81E-13 | 9.37E-14 |
| *N*-docosahexaenoyl tyrosine | PISSR |  |  | PISSR |  |  |
| ***N*-acyl valine** |  |  |  |  |  |  |
| *N*-palmitoyl valine | 1.27E-12 | 2.58E-13 | 8.6E-14 | 1.58E-12 | 3.67E-13 | 1.22E-13 |
| *N*- stearoyl valine | 6.12E-13 | 1.26E-13 | 4.19E-14 | 8.48E-13 | 1.28E-13 | 4.28E-14 |
| *N*-oleoyl valine | 5.65E-13 | 1.13E-13 | 3.78E-14 | 7.17E-13 | 1.73E-13 | 5.76E-14 |
| *N*-nervonoyl valine | BDL |  |  | BDL |  |  |
| *N*-linoleoyl valine | PISSR |  |  | PISSR |  |  |
| *N*-docosahexaenoyl valine | PISSR |  |  | PISSR |  |  |
| **2-acyl glycerols** |  |  |  |  |  |  |
| 2-palmitoyl glycerol | 7.79E-10 | 2.45E-10 | 8.15E-11 | 7.71E-10 | 2.45E-10 | 8.17E-11 |
| 2-oleoyl glycerol | 1.14E-09 | 2.02E-10 | 6.72E-11 | 9.43E-10 | 1.36E-10 | 4.52E-11 |
| 2-linoleoyl glycerol | 1.89E-10 | 4.09E-11 | 1.36E-11 | 1.2E-10 | 2.59E-11 | 8.62E-12 |
| 2-arachidonoyl glycerol | 3.13E-10 | 6.83E-11 | 2.28E-11 | 2.47E-10 | 4.74E-11 | 1.58E-11 |
| **Free Fatty Acids** |  |  |  |  |  |  |
| Oleic acid | 3.29E-09 | 3.27E-10 | 1.09E-10 | 2.69E-09 | 3.22E-10 | 1.07E-10 |
| Linoleic acid | 1.26E-09 | 1.19E-10 | 3.98E-11 | 9.72E-10 | 1.04E-10 | 3.47E-11 |
| Arachidonic acid | 5.04E-09 | 3.66E-10 | 1.22E-10 | 4.25E-09 | 3.45E-10 | 1.15E-10 |
| **PhosphoLEA** |  |  |  |  |  |  |
| PhosphoLEA | 2.68E-11 | 6.16E-12 | 2.05E-12 | 2.75E-11 | 5.82E-12 | 1.94E-12 |
| **Prostaglandins** |  |  |  |  |  |  |
| PGE_2_ | 9.87E-10 | 1.94E-10 | 6.48E-11 | 1.12E-09 | 1.93E-10 | 6.44E-11 |
| PGF_2α_ | 7.16E-10 | 8.26E-11 | 2.75E-11 | 9.18E-10 | 6.36E-11 | 2.12E-11 |
| 6-ketoPGF_1α_ | 1.46E-11 | 2.12E-12 | 7.06E-13 | 1.92E-11 | 3.19E-12 | 1.06E-12 |
| **THC/CP/CBD** |  |  |  |  |  |  |
| Cannabidiol | BDL |  |  | BDL |  |  |
| CP 55,940 | BDL |  |  | 9.63E-11 | 5.56E-12 | 1.85E-12 |
| THC | BDL |  |  | BDL |  |  |
| **THC Metabolites** |  |  |  |  |  |  |
| 11-nor-9-carboxyTHC | BDL |  |  | BDL |  |  |
| 11-OH-THC | BDL |  |  | BDL |  |  |

**Supplemental Table 4.** List of lipids in the hippocampus significantly affected by 3 mg/kg CP 55,940: PND 35

| Hippocampus Significant Differences in One-Way ANOVA | | | | |
| --- | --- | --- | --- | --- |
| Lipid | F | p | Direction | Magnitude |
| *N-*palmitoyl alanine | 4.16 | .058 | ↓ | 0.85 |
| *N-*stearoyl alanine | 20.67 | .000 | ↓ | 0.79 |
| *N-*oleoyl alanine | 32.79 | .000 | ↓ | 0.75 |
| *N-*linoleoyl alanine | 3.62 | .075 | ↓ | 0.82 |
| *N*-arachidonoyl alanine | 24.26 | .000 | ↓ | 0.76 |
| *N*-docosahexaenoyl alanine | 10.42 | .005 | ↓ | 0.76 |
| *N-*palmitoyl ethanolamine | 6.85 | .019 | ↓ | 0.79 |
| *N*-oleoyl ethanolamine | 8.61 | .010 | ↓ | 0.77 |
| *N*-arachidonoyl ethanolamine | 6.60 | .021 | ↓ | 0.82 |
| *N*-docosahexaenoyl ethanolamine | 18.42 | .001 | ↓ | 0.71 |
| *N*-stearoyl GABA | 9.07 | .008 | ↓ | 0.77 |
| *N*-oleoyl GABA | 31.16 | .000 | ↓ | 0.59 |
| *N*-linoleoyl GABA | 46.15 | .000 | ↓ | 0.55 |
| *N*-arachidonoyl GABA | 34.83 | .000 | ↓ | 0.66 |
| *N*-docosahexaenoyl GABA | 60.22 | .000 | ↓ | 0.52 |
| *N*-stearoyl glycine | 6.20 | .024 | ↓ | 0.87 |
| *N*-oleoyl glycine | 14.46 | .002 | ↓ | 0.87 |
| *N*-arachidonoyl glycine | 7.49 | .015 | ↓ | 0.86 |
| *N*-docosahexaenoyl glycine | 9.83 | .006 | ↓ | 0.83 |
| *N*-palmitoyl leucine | 53.80 | .000 | ↑ | 1.31 |
| *N*-docosahexaenoyl leucine | 9.71 | .007 | ↑ | 1.41 |
| *N*-oleoyl methionine | 4.11 | .060 | ↓ | 0.68 |
| *N*-palmitoyl phenylalanine | 12.01 | .003 | ↑ | 1.26 |
| *N*-arachidonoyl phenylalanine | 16.50 | .001 | ↓ | 0.76 |
| *N*-arachidonoyl serine | 88.74 | .000 | ↓ | 0.49 |
| *N*-docosahexaenoyl serine | 14.42 | .002 | ↓ | 0.74 |
| *N*-arachidonoyl taurine | 13.68 | .002 | ↓ | 0.81 |
| *N*-palmitoyl tyrosine | 9.78 | .006 | ↑ | 1.29 |
| *N*-palmitoyl valine | 4.07 | .061 | ↑ | 1.24 |
| *N*-stearoyl valine | 15.57 | .002 | ↑ | 1.39 |
| *N*-oleoyl valine | 4.89 | .042 | ↑ | 1.27 |
| 2-oleoyl glycerol | 6.00 | .026 | ↓ | 0.82 |
| 2-linoleoyl glycerol | 18.28 | .001 | ↓ | 0.63 |
| 2-arachidonoyl glycerol | 5.77 | .029 | ↓ | 0.79 |
| Oleic acid | 15.50 | .001 | ↓ | 0.82 |
| Linoleic acid | 29.88 | .000 | ↓ | 0.77 |
| Arachidonic acid | 22.25 | .000 | ↓ | 0.84 |
| PGF_2α_ | 33.82 | .000 | ↑ | 1.28 |
| 6-ketoPGF_1α_ | 12.85 | .002 | ↑ | 1.32 |
| CP 55,940 | 2701.91 | .000 | ↑ | infinite |

**Supplemental Table 5.** Lipid levels in the cerebellum of PND35 WT adolescent female mice treated with Vehicle or 3 mg/kg CP 55,940

|  | Cerebellum | | | | | | |
| --- | --- | --- | --- | --- | --- | --- | --- |
|  | Vehicle | | | 3mg/kg CP 55,940 | | | |
| ***N*-acyl alanine** | Mean | Std Dev | Std Error | | Mean | Std Dev | Std Error |
| *N*-palmitoyl alanine | 9.36E-12 | 2.13E-12 | 7.1E-13 | | 7.87E-12 | 1.9E-12 | 6.32E-13 |
| *N*-stearoyl alanine | 1.4E-11 | 2.14E-12 | 7.15E-13 | | 1.31E-11 | 4.28E-12 | 1.43E-12 |
| *N*-oleoyl alanine | 7.03E-12 | 1.12E-12 | 3.74E-13 | | 6.55E-12 | 1.56E-12 | 5.19E-13 |
| *N*-linoleoyl alanine | 1.34E-12 | 2.23E-13 | 7.43E-14 | | 1.26E-12 | 4.08E-13 | 1.36E-13 |
| *N*-arachidonoyl alanine | 7.46E-12 | 1.61E-12 | 5.36E-13 | | 5.29E-12 | 8.89E-13 | 2.96E-13 |
| *N*-docosahexaenoyl alanine | 4.33E-12 | 8E-13 | 2.67E-13 | | 3.11E-12 | 6.89E-13 | 2.3E-13 |
| ***N*-acyl dopamine** |  |  |  | |  |  |  |
| *N*-oleoyl dopamine | BDL |  |  | | BDL |  |  |
| *N*-arachidonoyl dopamine | BDL |  |  | | BDL |  |  |
| ***N*-acyl ethanolamine** |  |  |  | |  |  |  |
| *N*-palmitoyl ethanolamine | 3.79E-11 | 6.61E-12 | 2.2E-12 | | 3.88E-11 | 8.78E-12 | 2.93E-12 |
| *N*-stearoyl ethanolamine | 1.09E-11 | 3E-12 | 9.99E-13 | | 1.11E-11 | 4.26E-12 | 1.42E-12 |
| *N*-oleoyl ethanolamine | 9.88E-11 | 1.65E-11 | 5.49E-12 | | 7.98E-11 | 1.18E-11 | 3.94E-12 |
| *N*-linoleoyl ethanolamine | 2.84E-11 | 8.31E-12 | 2.77E-12 | | 2.95E-11 | 5.94E-12 | 1.98E-12 |
| *N*-arachidonoyl ethanolamine | 1.18E-11 | 2.81E-12 | 9.38E-13 | | 1.17E-11 | 2.18E-12 | 7.25E-13 |
| *N*-docosahexaenoyl ethanolamine | 2.28E-11 | 4.72E-12 | 1.57E-12 | | 2.05E-11 | 3.63E-12 | 1.21E-12 |
| ***N*-acyl GABA** |  |  |  | |  |  |  |
| *N*-palmitoyl GABA | 6.54E-12 | 1.44E-12 | 4.8E-13 | | 7.17E-12 | 2.12E-12 | 7.07E-13 |
| *N*-stearoyl GABA | 6.32E-12 | 1.05E-12 | 3.5E-13 | | 5.88E-12 | 1.19E-12 | 3.98E-13 |
| *N*-oleoyl GABA | 4.6E-12 | 8.78E-13 | 2.93E-13 | | 3.82E-12 | 8.01E-13 | 2.67E-13 |
| *N*-linoleoyl GABA | 8.52E-13 | 2.31E-13 | 7.71E-14 | | 7.16E-13 | 2.21E-13 | 7.38E-14 |
| *N*-arachidonoyl GABA | 1.25E-11 | 2.05E-12 | 6.82E-13 | | 9.02E-12 | 1.76E-12 | 5.88E-13 |
| *N*-docosahexaenoyl GABA | 2.79E-12 | 5.68E-13 | 1.89E-13 | | 2.27E-12 | 4.84E-13 | 1.61E-13 |
| ***N*-acyl glycine** |  |  |  | |  |  |  |
| *N*-palmitoyl glycine | 2.71E-11 | 2.21E-12 | 7.38E-13 | | 2.65E-11 | 2.31E-12 | 7.71E-13 |
| *N*-stearoyl glycine | 1.42E-11 | 2.07E-12 | 6.88E-13 | | 1.37E-11 | 1.68E-12 | 5.59E-13 |
| *N*-oleoyl glycine | 1.6E-11 | 1.24E-12 | 4.13E-13 | | 1.52E-11 | 1.42E-12 | 4.75E-13 |
| *N*-linoleoyl glycine | 3.66E-12 | 4.64E-13 | 1.55E-13 | | 3.27E-12 | 4.21E-13 | 1.4E-13 |
| *N*-arachidonoyl glycine | 2.86E-11 | 3.82E-12 | 1.27E-12 | | 2.55E-11 | 2.27E-12 | 7.57E-13 |
| *N*-docosahexaenoyl glycine | 1.46E-11 | 1.56E-12 | 5.21E-13 | | 1.34E-11 | 1.16E-12 | 3.87E-13 |
| ***N*-acyl leucine** |  |  |  | |  |  |  |
| *N*-palmitoyl leucine | 1.81E-12 | 1.46E-13 | 4.86E-14 | | 2.75E-12 | 2.58E-13 | 8.6E-14 |
| *N*-stearoyl leucine | 9.73E-13 | 1.32E-13 | 4.39E-14 | | 9.56E-13 | 9.15E-14 | 3.05E-14 |
| *N*-oleoyl leucine | 1.18E-12 | 1.03E-13 | 3.42E-14 | | 1.49E-12 | 1.1E-13 | 3.65E-14 |
| *N*-linoleoyl leucine | 2.84E-13 | 5.1E-14 | 1.7E-14 | | 3.65E-13 | 5.92E-14 | 1.97E-14 |
| *N*-docosahexaenoyl leucine | 5.15E-13 | 7.31E-14 | 2.44E-14 | | 7.43E-13 | 1.11E-13 | 3.7E-14 |
| ***N*-acyl methionine** |  |  |  | |  |  |  |
| *N*-palmitoyl methionine | 1.55E-12 | 3.91E-13 | 1.3E-13 | | 1.86E-12 | 4.49E-13 | 1.5E-13 |
| *N*-stearoyl methionine | 1.56E-12 | 4.05E-13 | 1.35E-13 | | 1.59E-12 | 2.33E-13 | 7.75E-14 |
| *N*-oleoyl methionine | 1.27E-12 | 2.01E-13 | 6.68E-14 | | 1.2E-12 | 2.67E-13 | 8.91E-14 |
| *N*-linoleoyl methionine | PISSR |  |  | | PISSR |  |  |
| *N*-arachidonoyl methionine | 1.35E-12 | 2.97E-13 | 9.9E-14 | | 8.56E-13 | 2.83E-13 | 9.44E-14 |
| *N*-docosahexaenoyl methionine | 6.24E-13 | 2.81E-13 | 9.36E-14 | | 5.43E-13 | 1.6E-13 | 5.35E-14 |
| ***N*-acyl phenylalanine** |  |  |  | |  |  |  |
| *N*-palmitoyl phenylalanine | 1.96E-12 | 1.68E-13 | 5.6E-14 | | 2.82E-12 | 2.03E-13 | 6.77E-14 |
| *N*-stearoyl phenylalanine | 1.87E-12 | 1.21E-13 | 4.02E-14 | | 1.92E-12 | 1.6E-13 | 5.34E-14 |
| *N*-oleoyl phenylalanine | 1.25E-12 | 1.31E-13 | 4.36E-14 | | 1.43E-12 | 1.55E-13 | 5.17E-14 |
| *N*-linoleoyl phenylalanine | 2.02E-13 | 6.32E-14 | 2.11E-14 | | 2.56E-13 | 5.18E-14 | 1.73E-14 |
| *N*-arachidonoyl phenylalanine | 1.17E-12 | 1.14E-13 | 3.79E-14 | | 1.22E-12 | 1.81E-13 | 6.04E-14 |
| *N*-docosahexaenoyl phenylalanine | 1.05E-12 | 1.66E-13 | 5.52E-14 | | 1.05E-12 | 2.05E-13 | 6.85E-14 |

**Supplemental Table 5: Continued**

|  | Cerebellum | | | | | |
| --- | --- | --- | --- | --- | --- | --- |
|  | Vehicle | | | 3mg/kg CP 55,940 | | |
| ***N*-acyl proline** | Mean | Std Dev | Std Error | Mean | Std Dev | Std Error |
| *N*-palmitoyl proline | 8.08E-13 | 1.2E-13 | 3.99E-14 | 7E-13 | 1.38E-13 | 4.6E-14 |
| *N*-stearoyl proline | 4.33E-13 | 4.56E-14 | 1.52E-14 | 3.97E-13 | 5.52E-14 | 1.84E-14 |
| *N-*oleoyl proline | 4E-13 | 1.21E-13 | 4.04E-14 | 3.42E-13 | 9E-14 | 3E-14 |
| *N*-linoleoyl proline | BDL |  |  | BDL |  |  |
| *N*-arachidonoyl proline | BDL |  |  | BDL |  |  |
| *N*-docosahexaenoyl proline | BDL |  |  | BDL |  |  |
| ***N*-acyl serine** |  |  |  |  |  |  |
| *N*-palmitoyl serine | 6.44E-11 | 6.53E-12 | 2.18E-12 | 5.23E-11 | 7.29E-12 | 2.43E-12 |
| *N*-stearoyl serine | 2.95E-11 | 1.7E-12 | 5.68E-13 | 2.93E-11 | 5.92E-12 | 1.97E-12 |
| *N*-oleoyl serine | 2.58E-10 | 2.19E-11 | 7.31E-12 | 2.53E-10 | 2.18E-11 | 7.27E-12 |
| *N*-linoleoyl serine | 4.63E-11 | 5.11E-12 | 1.7E-12 | 4.55E-11 | 4.23E-12 | 1.41E-12 |
| *N*-arachidonoyl serine | 7.24E-12 | 1.96E-12 | 6.52E-13 | 5.34E-12 | 1.63E-12 | 5.43E-13 |
| *N*-docosahexaenoyl serine | 4.02E-11 | 7.5E-12 | 2.5E-12 | 2.79E-11 | 6.19E-12 | 2.06E-12 |
| ***N*-acyl taurine** |  |  |  |  |  |  |
| *N*-arachidonoyl taurine | 8.23E-11 | 9.8E-12 | 3.27E-12 | 7.17E-11 | 1.3E-11 | 4.34E-12 |
| ***N*-acyl tryptophan** |  |  |  |  |  |  |
| *N*-palmitoyl tryptophan | 3.16E-13 | 5.13E-14 | 1.71E-14 | 3.12E-13 | 9.86E-14 | 3.29E-14 |
| *N*-stearoyl tryptophan | 7.01E-13 | 1.35E-13 | 4.51E-14 | 6.79E-13 | 1.13E-13 | 3.77E-14 |
| *N*-oleoyl tryptophan | 1.41E-13 | 3.81E-14 | 1.27E-14 | 1.48E-13 | 5.53E-14 | 1.84E-14 |
| *N*-linoleoyl tryptophan | PISSR |  |  | PISSR |  |  |
| *N*-arachidonoyl tryptophan | PISSR |  |  | PISSR |  |  |
| *N*-docosahexaenoyl tryptophan | PISSR |  |  | PISSR |  |  |
| ***N*-acyl tyrosine** |  |  |  |  |  |  |
| *N*-palmitoyl tyrosine | 1.33E-12 | 2.23E-13 | 7.44E-14 | 1.58E-12 | 1.63E-13 | 5.42E-14 |
| *N*-stearoyl tyrosine | 9.55E-14 | 2.36E-14 | 7.87E-15 | 8.44E-14 | 2.18E-14 | 7.27E-15 |
| *N-*oleoyl tyrosine | 6.95E-13 | 1.03E-13 | 3.44E-14 | 7.24E-13 | 1.34E-13 | 4.46E-14 |
| *N*-linoleoyl tyrosine | PISSR |  |  | PISSR |  |  |
| *N*-arachidonoyl tyrosine | 1.18E-12 | 2.27E-13 | 7.56E-14 | 8.32E-13 | 1.91E-13 | 6.36E-14 |
| *N*-docosahexaenoyl tyrosine | 1.87E-12 | 2.89E-13 | 9.64E-14 | 1.57E-12 | 3.8E-13 | 1.27E-13 |
| ***N*-acyl valine** |  |  |  |  |  |  |
| *N*-palmitoyl valine | 1.1E-13 | 3.67E-14 | 1.22E-14 | 1.73E-13 | 6.77E-14 | 2.26E-14 |
| *N*- stearoyl valine | PISSR |  |  | PISSR |  |  |
| *N*-oleoyl valine | PISSR |  |  | PISSR |  |  |
| *N*-nervonoyl valine | BDL |  |  | BDL |  |  |
| *N*-linoleoyl valine | BDL |  |  | BDL |  |  |
| *N*-docosahexaenoyl valine | BDL |  |  | BDL |  |  |
| **2-acyl glycerols** |  |  |  |  |  |  |
| 2-palmitoyl glycerol | 3.14E-10 | 7.83E-11 | 2.61E-11 | 3.01E-10 | 9.29E-11 | 3.1E-11 |
| 2-oleoyl glycerol | 8.9E-10 | 1.66E-10 | 5.52E-11 | 7.55E-10 | 1.95E-10 | 6.5E-11 |
| 2-linoleoyl glycerol | 1.33E-10 | 3.36E-11 | 1.12E-11 | 1.01E-10 | 2.21E-11 | 7.38E-12 |
| 2-arachidonoyl glycerol | 3.35E-10 | 6.18E-11 | 2.06E-11 | 2.68E-10 | 7.91E-11 | 2.64E-11 |
| **Free Fatty Acids** |  |  |  |  |  |  |
| Oleic acid | 1.08E-09 | 1.01E-10 | 3.37E-11 | 1.03E-09 | 7.95E-11 | 2.65E-11 |
| Linoleic acid | 4.52E-10 | 4.56E-11 | 1.52E-11 | 4.27E-10 | 2.99E-11 | 9.98E-12 |
| Arachidonic acid | 1.37E-09 | 9.15E-11 | 3.05E-11 | 1.26E-09 | 5.09E-11 | 1.7E-11 |
| **PhosphoLEA** |  |  |  |  |  |  |
| PhosphoLEA | 1E-11 | 1.98E-12 | 6.61E-13 | 1.04E-11 | 2.4E-12 | 7.99E-13 |
| **Prostaglandins** |  |  |  |  |  |  |
| PGE_2_ | 4.33E-10 | 6.03E-11 | 2.01E-11 | 4.01E-10 | 7.37E-11 | 2.46E-11 |
| PGF_2α_ | 3.02E-10 | 3.28E-11 | 1.09E-11 | 3.05E-10 | 4.39E-11 | 1.46E-11 |
| 6-ketoPGF_1α_ | 1.42E-11 | 3.84E-12 | 1.28E-12 | 8.74E-12 | 1.66E-12 | 5.54E-13 |
| **THC/CP/CBD** |  |  |  |  |  |  |
| Cannabidiol | BDL |  |  | BDL |  |  |
| CP 55,940 | BDL |  |  | 7.22E-11 | 4.78E-12 | 1.59E-12 |
| THC | BDL |  |  | BDL |  |  |
| **THC Metabolites** |  |  |  |  |  |  |
| 11-nor-9-carboxyTHC | BDL |  |  | BDL |  |  |
| 11-OH-THC | BDL |  |  | BDL |  |  |

**Supplemental Table 6.** List of lipids in the cerebellum significantly affected by 3 mg/kg CP 55,940: PND 35

| Cerebellum Significant Differences in One-Way ANOVA | | | | |
| --- | --- | --- | --- | --- |
| Lipid | F | p | Direction (relative to Veh) | Magnitude (x Veh level) |
| *N*-arachidonoyl alanine | 12.54 | .003 | ↓ | 0.71 |
| *N*-docosahexaenoyl alanine | 12.07 | .003 | ↓ | 0.72 |
| *N*-oleoyl ethanolamine | 7.64 | .014 | ↓ | 0.81 |
| *N*-oleoyl GABA | 3.85 | .068 | ↓ | 0.83 |
| *N*-arachidonoyl GABA | 14.70 | .001 | ↓ | 0.72 |
| *N*-docosahexaenoyl GABA | 4.40 | .052 | ↓ | 0.81 |
| *N*-linoleoyl glycine | 3.47 | .081 | ↓ | 0.89 |
| *N*-arachidonoyl glycine | 4.27 | .055 | ↓ | 0.89 |
| *N*-docosahexaenoyl glycine | 3.43 | .083 | ↓ | 0.92 |
| *N-*palmitoyl leucine | 92.29 | .000 | ↑ | 1.52 |
| *N*-oleoyl leucine | 36.64 | .000 | ↑ | 1.25 |
| *N*-linoleoyl leucine | 9.78 | .007 | ↑ | 1.29 |
| *N*-docosahexaenoyl leucine | 26.45 | .000 | ↑ | 1.44 |
| *N*-arachidonoyl methionine | 13.20 | .002 | ↓ | 0.63 |
| *N*-palmitoyl phenylalanine | 96.60 | .000 | ↑ | 1.43 |
| *N*-oleoyl phenylalanine | 6.98 | .018 | ↑ | 1.14 |
| *N*-linoleoyl phenylalanine | 4.06 | .061 | ↑ | 1.28 |
| *N*-palmitoyl proline | 3.16 | .095 | ↓ | 0.87 |
| *N*-palmitoyl serine | 13.80 | .002 | ↓ | 0.81 |
| *N*-arachidonoyl serine | 5.04 | .039 | ↓ | 0.74 |
| *N*-docosahexaenoyl serine | 14.44 | .002 | ↓ | 0.72 |
| *N*-arachidonoyl taurine | 3.77 | .070 | ↓ | 0.87 |
| *N-*palmitoyl tyrosine | 7.11 | .017 | ↑ | 1.19 |
| *N*-arachidonoyl tyrosine | 12.71 | .003 | ↓ | 0.70 |
| *N*-docosahexaenoyl tyrosine | 3.56 | .078 | ↓ | 0.84 |
| *N*-palmitoyl valine | 5.96 | .027 | ↑ | 1.70 |
| 2-linoleoyl glycerol | 5.87 | .028 | ↓ | 0.75 |
| 2-arachidonoyl glycerol | 4.00 | .063 | ↓ | 0.80 |
| Arachidonic acid | 9.80 | .006 | ↓ | 0.92 |
| 6-ketoPGF_1α_ | 15.16 | .001 | ↑ | 0.61 |
| CP 55,940 | 2052.69 | .000 | ↑ | infinite |

**Supplemental Table 7.** Lipid levels in the thalamus of PND 35 WT adolescent female mice treated with Vehicle or 3 mg/kg CP 55,940

|  | Thalamus | | | | | | |
| --- | --- | --- | --- | --- | --- | --- | --- |
|  | Vehicle | | | 3mg/kg CP 55,940 | | | |
| ***N*-acyl alanine** | Mean | Std Dev | Std Error | | Mean | Std Dev | Std Error |
| *N*-palmitoyl alanine | 1.34E-11 | 3.5E-12 | 1.17E-12 | | 1.21E-11 | 3.06E-12 | 1.02E-12 |
| *N*-stearoyl alanine | 1.41E-11 | 2.25E-12 | 7.5E-13 | | 1.24E-11 | 2.08E-12 | 6.93E-13 |
| *N*-oleoyl alanine | 9.24E-12 | 1.61E-12 | 5.38E-13 | | 7.79E-12 | 1.11E-12 | 3.69E-13 |
| *N*-linoleoyl alanine | 1.69E-12 | 6.41E-13 | 2.14E-13 | | 1.66E-12 | 3.34E-13 | 1.11E-13 |
| *N*-arachidonoyl alanine | 1.25E-11 | 2.72E-12 | 9.06E-13 | | 1.02E-11 | 1.7E-12 | 5.67E-13 |
| *N*-docosahexaenoyl alanine | 5.94E-12 | 1.27E-12 | 4.24E-13 | | 4.07E-12 | 1.41E-12 | 4.69E-13 |
| ***N*-acyl dopamine** |  |  |  | |  |  |  |
| *N*-oleoyl dopamine | BDL |  |  | | BDL |  |  |
| *N*-arachidonoyl dopamine | BDL |  |  | | BDL |  |  |
| ***N*-acyl ethanolamine** |  |  |  | |  |  |  |
| *N*-palmitoyl ethanolamine | 9.56E-11 | 8.71E-12 | 3.08E-12 | | 8.69E-11 | 1.44E-11 | 4.8E-12 |
| *N*-stearoyl ethanolamine | 1.98E-11 | 6.25E-12 | 2.21E-12 | | 1.97E-11 | 7.5E-12 | 2.5E-12 |
| *N*-oleoyl ethanolamine | 1.83E-10 | 2.41E-11 | 8.53E-12 | | 1.71E-10 | 2.76E-11 | 9.22E-12 |
| *N*-linoleoyl ethanolamine | 3.93E-11 | 7.84E-12 | 2.61E-12 | | 5.07E-11 | 9.4E-12 | 3.13E-12 |
| *N*-arachidonoyl ethanolamine | 2.31E-11 | 3.58E-12 | 1.19E-12 | | 2.8E-11 | 5.1E-12 | 1.7E-12 |
| *N*-docosahexaenoyl ethanolamine | 3.79E-11 | 8.39E-12 | 2.8E-12 | | 4.03E-11 | 9.22E-12 | 3.07E-12 |
| ***N*-acyl GABA** |  |  |  | |  |  |  |
| *N*-palmitoyl GABA | 1.17E-11 | 1.32E-12 | 4.41E-13 | | 1.41E-11 | 2.47E-12 | 8.23E-13 |
| *N*-stearoyl GABA | 1E-11 | 6.39E-13 | 2.13E-13 | | 1.05E-11 | 1.32E-12 | 4.39E-13 |
| *N*-oleoyl GABA | 6.89E-12 | 4.88E-13 | 1.63E-13 | | 5.58E-12 | 6.86E-13 | 2.29E-13 |
| *N*-linoleoyl GABA | 1.1E-12 | 2.13E-13 | 7.11E-14 | | 9.8E-13 | 2.25E-13 | 7.51E-14 |
| *N*-arachidonoyl GABA | 3.63E-11 | 2.7E-12 | 9.01E-13 | | 2.62E-11 | 3E-12 | 9.99E-13 |
| *N*-docosahexaenoyl GABA | 4.11E-12 | 4.17E-13 | 1.39E-13 | | 2.9E-12 | 4.94E-13 | 1.65E-13 |
| ***N*-acyl glycine** |  |  |  | |  |  |  |
| *N*-palmitoyl glycine | 5.28E-11 | 6.63E-12 | 2.21E-12 | | 4.99E-11 | 5.87E-12 | 1.96E-12 |
| *N*-stearoyl glycine | 2.9E-11 | 3.62E-12 | 1.21E-12 | | 2.74E-11 | 3.84E-12 | 1.28E-12 |
| *N*-oleoyl glycine | 2.6E-11 | 2.48E-12 | 8.25E-13 | | 2.36E-11 | 2.42E-12 | 8.07E-13 |
| *N*-linoleoyl glycine | 4.86E-12 | 1.26E-12 | 4.21E-13 | | 4.36E-12 | 8.16E-13 | 2.72E-13 |
| *N*-arachidonoyl glycine | 5.36E-11 | 8.12E-12 | 2.71E-12 | | 4.62E-11 | 6.61E-12 | 2.2E-12 |
| *N*-docosahexaenoyl glycine | 2.07E-11 | 3.05E-12 | 1.02E-12 | | 1.76E-11 | 3.21E-12 | 1.07E-12 |
| ***N*-acyl leucine** |  |  |  | |  |  |  |
| *N*-palmitoyl leucine | 2.88E-12 | 3.52E-13 | 1.17E-13 | | 4.1E-12 | 7.06E-13 | 2.35E-13 |
| *N*-stearoyl leucine | 1.63E-12 | 3.9E-13 | 1.3E-13 | | 1.66E-12 | 1.47E-13 | 4.91E-14 |
| *N*-oleoyl leucine | 1.65E-12 | 2.64E-13 | 8.81E-14 | | 2.09E-12 | 2.83E-13 | 9.44E-14 |
| *N*-linoleoyl leucine | 3.77E-13 | 8.42E-14 | 2.81E-14 | | 3.45E-13 | 9.22E-14 | 3.07E-14 |
| *N*-docosahexaenoyl leucine | 4.59E-13 | 6.03E-14 | 2.01E-14 | | 8.15E-13 | 1.13E-13 | 3.77E-14 |
| ***N*-acyl methionine** |  |  |  | |  |  |  |
| *N*-palmitoyl methionine | 1.45E-12 | 5.32E-13 | 1.77E-13 | | 1.84E-12 | 5.53E-13 | 1.84E-13 |
| *N*-stearoyl methionine | 2.01E-12 | 4.02E-13 | 1.34E-13 | | 1.9E-12 | 4.16E-13 | 1.39E-13 |
| *N*-oleoyl methionine | 1.15E-12 | 3.17E-13 | 1.06E-13 | | 1.17E-12 | 2.94E-13 | 9.79E-14 |
| *N*-linoleoyl methionine | BDL |  |  | | BDL |  |  |
| *N*-arachidonoyl methionine | PISSR |  |  | | PISSR |  |  |
| *N*-docosahexaenoyl methionine | PISSR |  |  | | PISSR |  |  |
| ***N*-acyl phenylalanine** |  |  |  | |  |  |  |
| *N*-palmitoyl phenylalanine | 2.04E-12 | 2.69E-13 | 8.98E-14 | | 2.75E-12 | 2.29E-13 | 7.63E-14 |
| *N*-stearoyl phenylalanine | 2.18E-12 | 1.4E-13 | 4.66E-14 | | 2.22E-12 | 2.1E-13 | 7E-14 |
| *N*-oleoyl phenylalanine | 1.13E-12 | 7.99E-14 | 2.66E-14 | | 1.2E-12 | 1.25E-13 | 4.17E-14 |
| *N*-linoleoyl phenylalanine | PISSR |  |  | | PISSR |  |  |
| *N*-arachidonoyl phenylalanine | 1.09E-12 | 2.59E-13 | 8.63E-14 | | 1.31E-12 | 1.94E-13 | 6.48E-14 |
| *N*-docosahexaenoyl phenylalanine | 9.13E-13 | 1.42E-13 | 4.73E-14 | | 1.14E-12 | 1.29E-13 | 4.3E-14 |

**Supplemental Table 7: Continued**

|  | Thalamus | | | | | |
| --- | --- | --- | --- | --- | --- | --- |
|  | Vehicle | | | 3mg/kg CP 55,940 | | |
| ***N*-acyl proline** | Mean | Std Dev | Std Error | Mean | Std Dev | Std Error |
| *N*-palmitoyl proline | 1.26E-12 | 4.57E-13 | 1.52E-13 | 1.05E-12 | 2.24E-13 | 7.47E-14 |
| *N*-stearoyl proline | 5.18E-13 | 1.67E-13 | 5.57E-14 | 5.08E-13 | 1.31E-13 | 4.35E-14 |
| *N-*oleoyl proline | PISSR |  |  | PISSR |  |  |
| *N*-linoleoyl proline | BDL |  |  | BDL |  |  |
| *N*-arachidonoyl proline | PISSR |  |  | PISSR |  |  |
| *N*-docosahexaenoyl proline | BDL |  |  | BDL |  |  |
| ***N*-acyl serine** |  |  |  |  |  |  |
| *N*-palmitoyl serine | 1.13E-10 | 1.47E-11 | 4.92E-12 | 1.05E-10 | 1.9E-11 | 6.34E-12 |
| *N*-stearoyl serine | 3.86E-11 | 5.05E-12 | 1.68E-12 | 3.19E-11 | 5.07E-12 | 1.69E-12 |
| *N*-oleoyl serine | 3.25E-10 | 4.17E-11 | 1.39E-11 | 3.24E-10 | 5.08E-11 | 1.69E-11 |
| *N*-linoleoyl serine | 5.95E-11 | 8.12E-12 | 2.71E-12 | 5.67E-11 | 1.21E-11 | 4.02E-12 |
| *N*-arachidonoyl serine | 7.75E-12 | 1.51E-12 | 5.02E-13 | 6.28E-12 | 1.22E-12 | 4.08E-13 |
| *N*-docosahexaenoyl serine | 2.92E-11 | 7.13E-12 | 2.38E-12 | 2.59E-11 | 4.93E-12 | 1.64E-12 |
| ***N*-acyl taurine** |  |  |  |  |  |  |
| *N*-arachidonoyl taurine | 6.85E-11 | 8.14E-12 | 2.71E-12 | 5.99E-11 | 8.6E-12 | 2.87E-12 |
| ***N*-acyl tryptophan** |  |  |  |  |  |  |
| *N*-palmitoyl tryptophan | 4.28E-13 | 1.18E-13 | 3.93E-14 | 4.31E-13 | 1.1E-13 | 3.67E-14 |
| *N*-stearoyl tryptophan | 1.31E-12 | 3.26E-13 | 1.09E-13 | 1.26E-12 | 2.16E-13 | 7.21E-14 |
| *N*-oleoyl tryptophan | 1.56E-13 | 6.69E-14 | 2.23E-14 | 1.87E-13 | 1.01E-13 | 3.36E-14 |
| *N*-linoleoyl tryptophan | BDL |  |  | BDL |  |  |
| *N*-arachidonoyl tryptophan | BDL |  |  | BDL |  |  |
| *N*-docosahexaenoyl tryptophan | PISSR |  |  | PISSR |  |  |
| ***N*-acyl tyrosine** |  |  |  |  |  |  |
| *N*-palmitoyl tyrosine | 9.82E-13 | 1.78E-13 | 5.93E-14 | 1.31E-12 | 1.61E-13 | 5.35E-14 |
| *N*-stearoyl tyrosine | 1.42E-13 | 3.69E-14 | 1.23E-14 | 1.15E-13 | 2.83E-14 | 9.44E-15 |
| *N-*oleoyl tyrosine | 4.75E-13 | 1.46E-13 | 4.86E-14 | 4.87E-13 | 1.45E-13 | 4.84E-14 |
| *N*-linoleoyl tyrosine | BDL |  |  | BDL |  |  |
| *N*-arachidonoyl tyrosine | 8.68E-13 | 2.27E-13 | 7.56E-14 | 7.9E-13 | 1.35E-13 | 4.51E-14 |
| *N*-docosahexaenoyl tyrosine | 7.09E-13 | 1.64E-13 | 5.48E-14 | 7.13E-13 | 2.51E-13 | 8.38E-14 |
| ***N*-acyl valine** |  |  |  |  |  |  |
| *N*-palmitoyl valine | 1.48E-12 | 2.51E-13 | 8.37E-14 | 2.65E-12 | 3.64E-13 | 1.21E-13 |
| *N*- stearoyl valine | 8.53E-13 | 2.01E-13 | 6.71E-14 | 1.07E-12 | 1.19E-13 | 3.98E-14 |
| *N*-oleoyl valine | 6.24E-13 | 6.87E-14 | 2.29E-14 | 9.01E-13 | 1.19E-13 | 3.95E-14 |
| *N*-nervonoyl valine | BDL |  |  | BDL |  |  |
| *N*-linoleoyl valine | PISSR |  |  | PISSR |  |  |
| *N*-docosahexaenoyl valine | 1.74E-13 | 7.49E-14 | 2.5E-14 | 2E-13 | 3.81E-14 | 1.27E-14 |
| **2-acyl glycerols** |  |  |  |  |  |  |
| 2-palmitoyl glycerol | 5.12E-10 | 1.27E-10 | 4.22E-11 | 5.34E-10 | 1.43E-10 | 4.75E-11 |
| 2-oleoyl glycerol | 1.73E-09 | 2.98E-10 | 9.94E-11 | 1.68E-09 | 4.04E-10 | 1.35E-10 |
| 2-linoleoyl glycerol | 1.93E-10 | 6.2E-11 | 2.07E-11 | 1.52E-10 | 2.8E-11 | 9.33E-12 |
| 2-arachidonoyl glycerol | 3.75E-10 | 9.68E-11 | 3.23E-11 | 3.59E-10 | 1.2E-10 | 4.02E-11 |
| **Free Fatty Acids** |  |  |  |  |  |  |
| Oleic acid | 1.74E-09 | 3.05E-10 | 1.02E-10 | 1.67E-09 | 1.77E-10 | 5.89E-11 |
| Linoleic acid | 6.15E-10 | 1.5E-10 | 5.01E-11 | 5.7E-10 | 6.24E-11 | 2.08E-11 |
| Arachidonic acid | 2.31E-09 | 3.74E-10 | 1.25E-10 | 2.14E-09 | 2.1E-10 | 6.99E-11 |
| **PhosphoLEA** |  |  |  |  |  |  |
| PhosphoLEA | 2.37E-11 | 5.21E-12 | 1.74E-12 | 2.93E-11 | 6.78E-12 | 2.26E-12 |
| **Prostaglandins** |  |  |  |  |  |  |
| PGE_2_ | 5.45E-10 | 8.14E-11 | 2.71E-11 | 6.31E-10 | 5.86E-11 | 1.95E-11 |
| PGF_2α_ | 4.91E-10 | 7.26E-11 | 2.42E-11 | 5.11E-10 | 5.87E-11 | 1.96E-11 |
| 6-ketoPGF_1α_ | 1.85E-11 | 5.71E-12 | 1.9E-12 | 1.42E-11 | 2.87E-12 | 9.56E-13 |
| **THC/CP/CBD** |  |  |  |  |  |  |
| Cannabidiol | BDL |  |  | BDL |  |  |
| CP 55,940 | BDL |  |  | 8.87E-11 | 7.48E-12 | 2.49E-12 |
| THC | BDL |  |  | BDL |  |  |
| **THC Metabolites** |  |  |  |  |  |  |
| 11-nor-9-carboxyTHC | BDL |  |  | BDL |  |  |
| 11-OH-THC | BDL |  |  | BDL |  |  |

**Supplemental Table 8.** List of lipids in the thalamus significantly affected by 3 mg/kg CP 55,940: PND 35

| Thalamus Significant Differences in One-Way ANOVA | | | | |
| --- | --- | --- | --- | --- |
| Lipid | F | p | Direction (relative to Veh) | Magnitude (x Veh level) |
| *N*-oleoyl alanine | 4.96 | .041 | ↓ | 0.84 |
| *N*-arachidonoyl alanine | 4.55 | .049 | ↓ | 0.82 |
| *N*-docosahexaenoyl alanine | 8.70 | .009 | ↓ | 0.69 |
| *N*-linoleoyl ethanolamine | 7.79 | .013 | ↑ | 1.29 |
| *N*-arachidonoyl ethanolamine | 5.56 | .031 | ↑ | 1.21 |
| *N*-palmitoyl GABA | 6.55 | .021 | ↑ | 1.21 |
| *N*-oleoyl GABA | 21.57 | .000 | ↓ | 0.81 |
| *N*-arachidonoyl GABA | 55.94 | .000 | ↓ | 0.72 |
| *N*-docosahexaenoyl GABA | 31.16 | .000 | ↓ | 0.71 |
| *N*-oleoyl glycine | 4.15 | .059 | ↓ | 0.91 |
| *N*-arachidonoyl glycine | 4.45 | .051 | ↓ | 0.86 |
| *N*-docosahexaenoyl glycine | 4.46 | .051 | ↓ | 0.85 |
| *N*-palmitoyl leucine | 21.40 | .000 | ↑ | 1.42 |
| *N*-oleoyl leucine | 11.15 | .004 | ↑ | 1.27 |
| *N*-docosahexaenoyl leucine | 69.28 | .000 | ↑ | 1.78 |
| *N*-palmitoyl phenylalanine | 35.83 | .000 | ↑ | 1.35 |
| *N*-arachidonoyl phenylalanine | 4.15 | .059 | ↑ | 1.20 |
| *N*-docosahexaenoyl phenylalanine | 12.28 | .003 | ↑ | 1.25 |
| *N*-stearoyl serine | 7.87 | .013 | ↓ | 0.83 |
| *N*-arachidonoyl serine | 5.12 | .038 | ↓ | 0.81 |
| *N*-arachidonoyl taurine | 4.75 | .045 | ↓ | 0.87 |
| *N*-palmitoyl tyrosine | 16.55 | .001 | ↑ | 1.34 |
| *N*-stearoyl tyrosine | 3.10 | .098 | ↓ | 0.81 |
| *N*-palmitoyl valine | 63.17 | .000 | ↑ | 1.79 |
| *N*-stearoyl valine | 7.58 | .014 | ↑ | 1.26 |
| *N*-oleoyl valine | 36.70 | .000 | ↑ | 1.44 |
| 2-linoleoyl glycerol | 3.29 | .088 | ↓ | 0.79 |
| phosphoLEA | 3.94 | .065 | ↑ | 1.24 |
| PGE_2_ | 6.60 | .021 | ↑ | 1.16 |
| 6-ketoPGF_1α_ | 4.19 | .057 | ↓ | 0.77 |
| CP 55,940 | 1264.94 | .000 | ↑ | infinite |

**Supplemental Table 9.** Lipid levels in the cortex of WT PND 35 adolescent female mice treated with Vehicle or 3 mg/kg CP 55,940

|  | Cortex | | | | | | |
| --- | --- | --- | --- | --- | --- | --- | --- |
|  | Vehicle | | | 3mg/kg CP 55,940 | | | |
| ***N*-acyl alanine** | Mean | Std Dev | Std Error | | Mean | Std Dev | Std Error |
| *N*-palmitoyl alanine | 5.75E-12 | 1.43E-12 | 4.78E-13 | | 4.96E-12 | 1.39E-12 | 4.65E-13 |
| *N*-stearoyl alanine | 1.18E-11 | 3.86E-12 | 1.29E-12 | | 1.04E-11 | 2.08E-12 | 6.94E-13 |
| *N*-oleoyl alanine | 3.72E-12 | 7.44E-13 | 2.48E-13 | | 2.8E-12 | 5.4E-13 | 1.8E-13 |
| *N*-linoleoyl alanine | 7.46E-13 | 1.77E-13 | 5.89E-14 | | 6.3E-13 | 2.14E-13 | 7.12E-14 |
| *N*-arachidonoyl alanine | 3.64E-12 | 9.11E-13 | 3.04E-13 | | 2.81E-12 | 6.12E-13 | 2.04E-13 |
| *N*-docosahexaenoyl alanine | 7.77E-13 | 1.3E-13 | 4.32E-14 | | 7.37E-13 | 1.72E-13 | 5.73E-14 |
| ***N*-acyl dopamine** |  |  |  | |  |  |  |
| *N*-oleoyl dopamine | BDL |  |  | | BDL |  |  |
| *N*-arachidonoyl dopamine | BDL |  |  | | BDL |  |  |
| ***N*-acyl ethanolamine** |  |  |  | |  |  |  |
| *N*-palmitoyl ethanolamine | 1.76E-10 | 5.98E-11 | 1.99E-11 | | 1.43E-10 | 3.07E-11 | 1.02E-11 |
| *N*-stearoyl ethanolamine | 6.11E-11 | 2.17E-11 | 7.23E-12 | | 5.63E-11 | 2.06E-11 | 6.87E-12 |
| *N*-oleoyl ethanolamine | 5.02E-10 | 1.59E-10 | 5.31E-11 | | 4.68E-10 | 8.46E-11 | 2.82E-11 |
| *N*-linoleoyl ethanolamine | 1.99E-10 | 6.7E-11 | 2.23E-11 | | 2.31E-10 | 6.33E-11 | 2.11E-11 |
| *N*-arachidonoyl ethanolamine | 1.03E-10 | 2.29E-11 | 7.63E-12 | | 1.13E-10 | 2.67E-11 | 8.91E-12 |
| *N*-docosahexaenoyl ethanolamine | 1.21E-10 | 4.62E-11 | 1.54E-11 | | 1.21E-10 | 4.7E-11 | 1.57E-11 |
| ***N*-acyl GABA** |  |  |  | |  |  |  |
| *N*-palmitoyl GABA | 1.26E-11 | 2.14E-12 | 7.13E-13 | | 1.28E-11 | 2.1E-12 | 6.99E-13 |
| *N*-stearoyl GABA | 1.51E-11 | 1.92E-12 | 6.42E-13 | | 1.48E-11 | 1.81E-12 | 6.04E-13 |
| *N*-oleoyl GABA | 1.12E-11 | 1.34E-12 | 4.47E-13 | | 9.1E-12 | 1.13E-12 | 3.77E-13 |
| *N*-linoleoyl GABA | 4.3E-12 | 6.22E-13 | 2.07E-13 | | 3.78E-12 | 5.84E-13 | 1.95E-13 |
| *N*-arachidonoyl GABA | 2.72E-11 | 3.37E-12 | 1.12E-12 | | 2.26E-11 | 2.67E-12 | 8.89E-13 |
| *N*-docosahexaenoyl GABA | 5.47E-12 | 6.87E-13 | 2.29E-13 | | 4.81E-12 | 8.63E-13 | 2.88E-13 |
| ***N*-acyl glycine** |  |  |  | |  |  |  |
| *N*-palmitoyl glycine | 1.36E-11 | 1.65E-12 | 5.5E-13 | | 1.28E-11 | 2.8E-12 | 9.34E-13 |
| *N*-stearoyl glycine | 7.76E-12 | 1.45E-12 | 4.82E-13 | | 7.24E-12 | 1.25E-12 | 4.16E-13 |
| *N*-oleoyl glycine | 6.18E-12 | 6.47E-13 | 2.16E-13 | | 5.53E-12 | 6.48E-13 | 2.16E-13 |
| *N*-linoleoyl glycine | 5.22E-13 | 1.03E-13 | 3.44E-14 | | 3.61E-13 | 1.47E-13 | 4.91E-14 |
| *N*-arachidonoyl glycine | 1.8E-11 | 3.57E-12 | 1.19E-12 | | 1.35E-11 | 3.68E-12 | 1.23E-12 |
| *N*-docosahexaenoyl glycine | 1.52E-12 | 2.06E-13 | 6.85E-14 | | 1.18E-12 | 2.97E-13 | 9.91E-14 |
| ***N*-acyl leucine** |  |  |  | |  |  |  |
| *N*-palmitoyl leucine | 1.16E-12 | 9.19E-14 | 3.06E-14 | | 1.76E-12 | 2.48E-13 | 8.27E-14 |
| *N*-stearoyl leucine | 1.6E-12 | 1.08E-13 | 3.61E-14 | | 1.55E-12 | 1.23E-13 | 4.09E-14 |
| *N*-oleoyl leucine | 3.57E-13 | 3.14E-14 | 1.05E-14 | | 4.34E-13 | 6.73E-14 | 2.24E-14 |
| *N*-linoleoyl leucine | 1.38E-13 | 3.14E-14 | 1.05E-14 | | 1.58E-13 | 4.89E-14 | 1.63E-14 |
| *N*-docosahexaenoyl leucine | 6.66E-13 | 6.35E-14 | 2.12E-14 | | 1.02E-12 | 1.35E-13 | 4.48E-14 |
| ***N*-acyl methionine** |  |  |  | |  |  |  |
| *N*-palmitoyl methionine | 1.56E-12 | 4.06E-13 | 1.35E-13 | | 1.41E-12 | 4.7E-13 | 1.57E-13 |
| *N*-stearoyl methionine | 1.74E-12 | 2.9E-13 | 9.68E-14 | | 1.68E-12 | 3.95E-13 | 1.32E-13 |
| *N*-oleoyl methionine | 6.23E-13 | 1.94E-13 | 6.47E-14 | | 4.2E-13 | 1.16E-13 | 3.87E-14 |
| *N*-linoleoyl methionine | PISSR |  |  | | PISSR |  |  |
| *N*-arachidonoyl methionine | 4.46E-13 | 1.6E-13 | 5.34E-14 | | 2.69E-13 | 1.32E-13 | 4.42E-14 |
| *N*-docosahexaenoyl methionine | PISSR |  |  | | PISSR |  |  |
| ***N*-acyl phenylalanine** |  |  |  | |  |  |  |
| *N*-palmitoyl phenylalanine | 3.37E-12 | 5.04E-13 | 1.68E-13 | | 3.73E-12 | 8.69E-13 | 2.9E-13 |
| *N*-stearoyl phenylalanine | 3.52E-12 | 2.64E-13 | 8.79E-14 | | 3.37E-12 | 3.04E-13 | 1.01E-13 |
| *N*-oleoyl phenylalanine | 2.2E-12 | 1.97E-13 | 6.55E-14 | | 2.01E-12 | 2.76E-13 | 9.19E-14 |
| *N*-linoleoyl phenylalanine | 1.68E-13 | 4.95E-14 | 1.65E-14 | | 1.48E-13 | 5.26E-14 | 1.75E-14 |
| *N*-arachidonoyl phenylalanine | 9.59E-13 | 1.46E-13 | 4.85E-14 | | 7.25E-13 | 1.35E-13 | 4.51E-14 |
| *N*-docosahexaenoyl phenylalanine | 6.71E-13 | 1.59E-13 | 5.29E-14 | | 5.48E-13 | 1.93E-13 | 6.42E-14 |

**Supplemental Table 9: Continued**

|  | Cortex | | | | | |
| --- | --- | --- | --- | --- | --- | --- |
|  | Vehicle | | | 3mg/kg CP 55,940 | | |
| ***N*-acyl proline** | Mean | Std Dev | Std Error | Mean | Std Dev | Std Error |
| *N*-palmitoyl proline | 8.6E-14 | 2.19E-14 | 7.29E-15 | 7.7E-14 | 3.12E-14 | 1.04E-14 |
| *N*-stearoyl proline | 2.99E-14 | 7.06E-15 | 2.35E-15 | 3.31E-14 | 9.17E-15 | 3.06E-15 |
| *N-*oleoyl proline | PISSR |  |  | PISSR |  |  |
| *N*-linoleoyl proline | BDL |  |  | BDL |  |  |
| *N*-arachidonoyl proline | BDL |  |  | BDL |  |  |
| *N*-docosahexaenoyl proline | BDL |  |  | BDL |  |  |
| ***N*-acyl serine** |  |  |  |  |  |  |
| *N*-palmitoyl serine | 1.4E-11 | 3.69E-12 | 1.23E-12 | 1.08E-11 | 3.23E-12 | 1.08E-12 |
| *N*-stearoyl serine | 1.61E-11 | 2.29E-12 | 7.63E-13 | 1.35E-11 | 2.5E-12 | 8.35E-13 |
| *N*-oleoyl serine | 6.75E-11 | 1.21E-11 | 4.04E-12 | 5.9E-11 | 2.28E-11 | 7.61E-12 |
| *N*-linoleoyl serine | 1.32E-11 | 1.28E-12 | 4.27E-13 | 1.38E-11 | 5.86E-12 | 1.95E-12 |
| *N*-arachidonoyl serine | 3.21E-12 | 1.04E-12 | 3.45E-13 | 2.41E-12 | 7.89E-13 | 2.63E-13 |
| *N*-docosahexaenoyl serine | 9.15E-12 | 2.34E-12 | 7.79E-13 | 5.64E-12 | 2.58E-12 | 8.59E-13 |
| ***N*-acyl taurine** |  |  |  |  |  |  |
| *N*-arachidonoyl taurine | 1.56E-10 | 1.2E-11 | 4.01E-12 | 1.3E-10 | 1.72E-11 | 5.74E-12 |
| ***N*-acyl tryptophan** |  |  |  |  |  |  |
| *N*-palmitoyl tryptophan | 9.15E-13 | 2.85E-13 | 9.48E-14 | 9.75E-13 | 3.55E-13 | 1.18E-13 |
| *N*-stearoyl tryptophan | 3.67E-12 | 5.55E-13 | 1.85E-13 | 3.26E-12 | 6.11E-13 | 2.04E-13 |
| *N*-oleoyl tryptophan | PISSR |  |  | PISSR |  |  |
| *N*-linoleoyl tryptophan | BDL |  |  | BDL |  |  |
| *N*-arachidonoyl tryptophan | BDL |  |  | BDL |  |  |
| *N*-docosahexaenoyl tryptophan | BDL |  |  | BDL |  |  |
| ***N*-acyl tyrosine** |  |  |  |  |  |  |
| *N*-palmitoyl tyrosine | 1.59E-12 | 9.71E-14 | 3.24E-14 | 1.99E-12 | 3.59E-13 | 1.2E-13 |
| *N*-stearoyl tyrosine | 2.08E-13 | 4.61E-14 | 1.54E-14 | 3.04E-13 | 5.5E-14 | 1.83E-14 |
| *N-*oleoyl tyrosine | 6.55E-13 | 1.6E-13 | 5.34E-14 | 6.76E-13 | 1.31E-13 | 4.37E-14 |
| *N*-linoleoyl tyrosine | PISSR |  |  | PISSR |  |  |
| *N*-arachidonoyl tyrosine | 1.17E-12 | 1.37E-13 | 4.55E-14 | 8.47E-13 | 1.25E-13 | 4.16E-14 |
| *N*-docosahexaenoyl tyrosine | 6.58E-13 | 1.24E-13 | 4.14E-14 | 5.7E-13 | 1.58E-13 | 5.28E-14 |
| ***N*-acyl valine** |  |  |  |  |  |  |
| *N*-palmitoyl valine | 9.7E-13 | 1.49E-13 | 4.95E-14 | 1.52E-12 | 2.84E-13 | 9.47E-14 |
| *N*- stearoyl valine | 1.11E-12 | 2.08E-13 | 6.93E-14 | 1.46E-12 | 2.13E-13 | 7.09E-14 |
| *N*-oleoyl valine | 3.5E-13 | 7.92E-14 | 2.64E-14 | 4.65E-13 | 7.79E-14 | 2.6E-14 |
| *N*-nervonoyl valine | BDL |  |  | BDL |  |  |
| *N*-linoleoyl valine | PISSR |  |  | PISSR |  |  |
| *N*-docosahexaenoyl valine | 1.87E-13 | 3.31E-14 | 1.1E-14 | 2.57E-13 | 1.15E-13 | 3.82E-14 |
| **2-acyl glycerols** |  |  |  |  |  |  |
| 2-palmitoyl glycerol | 3.48E-09 | 1.88E-09 | 6.26E-10 | 5.49E-09 | 2.59E-09 | 8.62E-10 |
| 2-oleoyl glycerol | 4.43E-09 | 1.54E-09 | 5.12E-10 | 5.02E-09 | 1.39E-09 | 4.64E-10 |
| 2-linoleoyl glycerol | 1.62E-10 | 7.29E-11 | 2.43E-11 | 1.12E-10 | 5.03E-11 | 1.68E-11 |
| 2-arachidonoyl glycerol | 1.11E-09 | 3.03E-10 | 1.01E-10 | 1.2E-09 | 7.41E-10 | 2.47E-10 |
| **Free Fatty Acids** |  |  |  |  |  |  |
| Oleic acid | 7.72E-09 | 1.79E-09 | 5.95E-10 | 6.83E-09 | 2.24E-09 | 7.46E-10 |
| Linoleic acid | 6.5E-10 | 1.21E-10 | 4.05E-11 | 6.06E-10 | 1.48E-10 | 4.94E-11 |
| Arachidonic acid | 1.85E-09 | 2.2E-10 | 7.33E-11 | 1.78E-09 | 2.75E-10 | 9.16E-11 |
| **PhosphoLEA** |  |  |  |  |  |  |
| PhosphoLEA | 5.94E-12 | 1.09E-12 | 3.63E-13 | 8.98E-12 | 1.62E-12 | 5.4E-13 |
| **Prostaglandins** |  |  |  |  |  |  |
| PGE_2_ | 4.51E-10 | 6.68E-11 | 2.23E-11 | 4.31E-10 | 6.24E-11 | 2.08E-11 |
| PGF_2α_ | 2.62E-10 | 2.42E-11 | 8.08E-12 | 3.11E-10 | 2.26E-11 | 7.54E-12 |
| 6-ketoPGF_1α_ | 8.28E-12 | 9.59E-13 | 3.2E-13 | 5.64E-12 | 4.98E-13 | 1.66E-13 |
| **THC/CP/CBD** |  |  |  |  |  |  |
| Cannabidiol | BDL |  |  | BDL |  |  |
| CP 55,940 | BDL |  |  | 1.11E-10 | 1.03E-11 | 3.43E-12 |
| THC | BDL |  |  | BDL |  |  |
| **THC Metabolites** |  |  |  |  |  |  |
| 11-nor-9-carboxyTHC | BDL |  |  | BDL |  |  |
| 11-OH-THC | BDL |  |  | BDL |  |  |

**Supplemental Table 10.** List of lipids in the cortex significantly affected by 3 mg/kg CP 55,940: PND 35

| PND 35 Cortex Significant Differences in One-Way ANOVA | | | | |
| --- | --- | --- | --- | --- |
| Lipid | F | p | Direction (rel to Veh) | Magnitude (x Veh level) |
| *N-*oleoyl alanine | 8.87 | .009 | ↓ | 0.75 |
| *N*-arachidonoyl alanine | 5.21 | .036 | ↓ | 0.77 |
| *N*-oleoyl GABA | 12.99 | .002 | ↓ | 0.81 |
| *N*-linoleoyl GABA | 3.33 | .087 | ↓ | 0.88 |
| *N*-arachidonoyl GABA | 10.51 | .005 | ↓ | 0.83 |
| *N*-docosahexaenoyl GABA | 3.23 | .087 | ↓ | 0.88 |
| *N*-oleoyl glycine | 4.46 | .051 | ↓ | 0.89 |
| *N*-linoleoyl glycine | 7.28 | .016 | ↓ | 0.69 |
| *N*-arachidonoyl glycine | 6.88 | .018 | ↓ | 0.75 |
| *N*-docosahexaenoyl glycine | 7.90 | .013 | ↓ | 0.78 |
| *N*-palmitoyl leucine | 46.82 | .000 | ↑ | 1.52 |
| *N*-oleoyl leucine | 9.65 | .007 | ↑ | 1.22 |
| *N*-docosahexaenoyl leucine | 50.02 | .000 | ↑ | 1.52 |
| *N*-oleoyl methionine | 7.22 | .016 | ↓ | 0.67 |
| *N*-arachidonoyl methionine | 6.48 | .022 | ↓ | 0.60 |
| *N*-arachidonoyl phenylalanine | 12.53 | .003 | ↓ | 0.76 |
| *N*-palmitoyl serine | 3.92 | .065 | ↓ | 0.77 |
| *N*-stearoyl serine | 5.22 | .036 | ↓ | 0.84 |
| *N*-arachidonoyl serine | 3.41 | .083 | ↓ | 0.75 |
| *N*-docosahexaenoyl serine | 9.16 | .008 | ↓ | 0.62 |
| *N*-arachidonoyl taurine | 14.43 | .002 | ↓ | 0.83 |
| *N*-palmitoyl tyrosine | 10.63 | .005 | ↑ | 1.25 |
| *N*-stearoyl tyrosine | 15.96 | .001 | ↑ | 1.46 |
| *N*-arachidonoyl tyrosine | 27.10 | .000 | ↓ | 0.73 |
| *N*-palmitoyl valine | 26.97 | .000 | ↑ | 1.57 |
| *N*-stearoyl valine | 12.58 | .003 | ↑ | 1.32 |
| *N*-oleoyl valine | 9.66 | .007 | ↑ | 1.33 |
| *N*-docosahexaenoyl valine | 3.13 | .096 | ↑ | 1.37 |
| 2-palmitoyl glycerol | 3.56 | .078 | ↑ | 1.57 |
| phosphoLEA | 21.78 | .000 | ↑ | 1.51 |
| PGF_2α_ | 19.80 | .000 | ↑ | 1.19 |
| 6-ketoPGF_1α_ | 53.92 | .000 | ↓ | 0.68 |
| CP 55,940 | 1048.35 | .000 | ↑ | infinite |

**Supplemental Table 11.** Lipid levels in the hypothalamus of adolescent (PND 35) female mice treated with Vehicle or 3 mg/kg CP 55,940

|  | Hypothalamus | | | | | | |
| --- | --- | --- | --- | --- | --- | --- | --- |
|  | Vehicle | | | 3mg/kg CP 55,940 | | | |
| ***N*-acyl alanine** | Mean | Std Dev | Std Error | | Mean | Std Dev | Std Error |
| *N*-palmitoyl alanine | 3.28E-11 | 5.68E-12 | 1.89E-12 | | 3.03E-11 | 4.05E-12 | 1.35E-12 |
| *N*-stearoyl alanine | 2.1E-11 | 3.12E-12 | 1.04E-12 | | 1.89E-11 | 3.14E-12 | 1.05E-12 |
| *N*-oleoyl alanine | 1.35E-11 | 1.7E-12 | 5.67E-13 | | 1.14E-11 | 6.19E-13 | 2.06E-13 |
| *N*-linoleoyl alanine | 5.53E-12 | 6.98E-13 | 2.33E-13 | | 4.26E-12 | 6.54E-13 | 2.18E-13 |
| *N*-arachidonoyl alanine | 9.41E-12 | 7.27E-13 | 2.42E-13 | | 5.15E-12 | 7.41E-13 | 2.47E-13 |
| *N*-docosahexaenoyl alanine | PISSR |  |  | | PISSR |  |  |
| ***N*-acyl dopamine** |  |  |  | |  |  |  |
| *N*-oleoyl dopamine | BDL |  |  | | BDL |  |  |
| *N*-arachidonoyl dopamine | BDL |  |  | | BDL |  |  |
| ***N*-acyl ethanolamine** |  |  |  | |  |  |  |
| *N*-palmitoyl ethanolamine | 6.78E-11 | 1.13E-11 | 3.77E-12 | | 4.49E-11 | 1.16E-11 | 3.87E-12 |
| *N*-stearoyl ethanolamine | 4.98E-11 | 6.84E-12 | 2.28E-12 | | 3.64E-11 | 1.04E-11 | 3.46E-12 |
| *N*-oleoyl ethanolamine | 2.75E-10 | 7E-11 | 2.33E-11 | | 1.79E-10 | 6.32E-11 | 2.11E-11 |
| *N*-linoleoyl ethanolamine | 2.08E-11 | 5.42E-12 | 1.81E-12 | | 1.7E-11 | 3.84E-12 | 1.28E-12 |
| *N*-arachidonoyl ethanolamine | 7.41E-12 | 1.37E-12 | 4.56E-13 | | 5.6E-12 | 9.6E-13 | 3.2E-13 |
| *N*-docosahexaenoyl ethanolamine | 2.85E-11 | 4.55E-12 | 1.52E-12 | | 1.68E-11 | 2.82E-12 | 9.4E-13 |
| ***N*-acyl GABA** |  |  |  | |  |  |  |
| *N*-palmitoyl GABA | 3.31E-11 | 6.12E-12 | 2.04E-12 | | 3.44E-11 | 4.33E-12 | 1.44E-12 |
| *N*-stearoyl GABA | 3.88E-11 | 5.51E-12 | 1.84E-12 | | 3.84E-11 | 5.19E-12 | 1.73E-12 |
| *N*-oleoyl GABA | 2.33E-11 | 2.55E-12 | 8.49E-13 | | 2.02E-11 | 3.13E-12 | 1.04E-12 |
| *N*-linoleoyl GABA | PISSR |  |  | | PISSR |  |  |
| *N*-arachidonoyl GABA | 5.33E-11 | 4.7E-12 | 1.57E-12 | | 3.93E-11 | 4.25E-12 | 1.42E-12 |
| *N*-docosahexaenoyl GABA | 7.75E-12 | 6.57E-13 | 2.19E-13 | | 5.25E-12 | 5.82E-13 | 1.94E-13 |
| ***N*-acyl glycine** |  |  |  | |  |  |  |
| *N*-palmitoyl glycine | 1.27E-11 | 2.42E-12 | 8.07E-13 | | 1.16E-11 | 1.61E-12 | 5.38E-13 |
| *N*-stearoyl glycine | 9.06E-12 | 2.27E-12 | 7.55E-13 | | 8.37E-12 | 2.03E-12 | 6.78E-13 |
| *N*-oleoyl glycine | 3.93E-12 | 1.19E-12 | 3.98E-13 | | 3.5E-12 | 8.27E-13 | 2.76E-13 |
| *N*-linoleoyl glycine | 3.56E-13 | 1.97E-13 | 6.58E-14 | | 3.45E-13 | 1E-13 | 3.35E-14 |
| *N*-arachidonoyl glycine | 7.62E-12 | 1.81E-12 | 6.03E-13 | | 5.39E-12 | 1.42E-12 | 4.72E-13 |
| *N*-docosahexaenoyl glycine | 5.68E-13 | 1.6E-13 | 5.34E-14 | | 4.59E-13 | 1.72E-13 | 5.75E-14 |
| ***N*-acyl leucine** |  |  |  | |  |  |  |
| *N*-palmitoyl leucine | 1.63E-12 | 2.24E-13 | 7.46E-14 | | 2.12E-12 | 2.1E-13 | 6.99E-14 |
| *N*-stearoyl leucine | 1.48E-12 | 3.58E-13 | 1.19E-13 | | 1.34E-12 | 2.33E-13 | 7.75E-14 |
| *N*-oleoyl leucine | 3.49E-13 | 9.63E-14 | 3.21E-14 | | 3.49E-13 | 1.02E-13 | 3.39E-14 |
| *N*-linoleoyl leucine | BDL |  |  | | BDL |  |  |
| *N*-docosahexaenoyl leucine | PISSR |  |  | | PISSR |  |  |
| ***N*-acyl methionine** |  |  |  | |  |  |  |
| *N*-palmitoyl methionine | 6.31E-12 | 2.53E-12 | 8.44E-13 | | 5.5E-12 | 1.31E-12 | 4.35E-13 |
| *N*-stearoyl methionine | PISSR |  |  | | PISSR |  |  |
| *N*-oleoyl methionine | PISSR |  |  | | PISSR |  |  |
| *N*-linoleoyl methionine | BDL |  |  | | BDL |  |  |
| *N*-arachidonoyl methionine | PISSR |  |  | | PISSR |  |  |
| *N*-docosahexaenoyl methionine | BDL |  |  | | BDL |  |  |
| ***N*-acyl phenylalanine** |  |  |  | |  |  |  |
| *N*-palmitoyl phenylalanine | 3.72E-12 | 8.06E-13 | 2.69E-13 | | 4.4E-12 | 9.5E-13 | 3.17E-13 |
| *N*-stearoyl phenylalanine | 1.68E-12 | 5.44E-13 | 1.81E-13 | | 1.92E-12 | 2.55E-13 | 8.51E-14 |
| *N*-oleoyl phenylalanine | 1.85E-12 | 3.08E-13 | 1.03E-13 | | 1.8E-12 | 3.94E-13 | 1.31E-13 |
| *N*-linoleoyl phenylalanine | PISSR |  |  | | PISSR |  |  |
| *N*-arachidonoyl phenylalanine | 8.53E-13 | 1.2E-13 | 3.99E-14 | | 7.34E-13 | 1.47E-13 | 4.88E-14 |
| *N*-docosahexaenoyl phenylalanine | PISSR |  |  | | PISSR |  |  |

**Supplemental Table 11: Continued**

|  | Hypothalamus | | | | | |
| --- | --- | --- | --- | --- | --- | --- |
|  | Vehicle | | | CP 55,940 | | |
| ***N*-acyl proline** | Mean | Std Dev | Std Error | Mean | Std Dev | Std Error |
| *N*-palmitoyl proline | PISSR |  |  | PISSR |  |  |
| *N*-stearoyl proline | PISSR |  |  | PISSR |  |  |
| *N-*oleoyl proline | PISSR |  |  | PISSR |  |  |
| *N*-linoleoyl proline | PISSR |  |  | PISSR |  |  |
| *N*-arachidonoyl proline | PISSR |  |  | PISSR |  |  |
| *N*-docosahexaenoyl proline | BDL |  |  | BDL |  |  |
| ***N*-acyl serine** |  |  |  |  |  |  |
| *N*-palmitoyl serine | 1.04E-11 | 2.67E-12 | 8.9E-13 | 1.07E-11 | 2.06E-12 | 6.86E-13 |
| *N*-stearoyl serine | 2.11E-11 | 3.86E-12 | 1.29E-12 | 2.17E-11 | 2.77E-12 | 9.24E-13 |
| *N*-oleoyl serine | 2.77E-10 | 5.52E-11 | 1.84E-11 | 2.72E-10 | 3.63E-11 | 1.21E-11 |
| *N*-linoleoyl serine | 6.11E-11 | 1.2E-11 | 4.01E-12 | 6.26E-11 | 9.53E-12 | 3.18E-12 |
| *N*-arachidonoyl serine | PISSR |  |  | PISSR |  |  |
| *N*-docosahexaenoyl serine | PISSR |  |  | PISSR |  |  |
| ***N*-acyl taurine** |  |  |  |  |  |  |
| *N*-arachidonoyl taurine | 2.24E-11 | 3.01E-12 | 1E-12 | 1.56E-11 | 2.23E-12 | 7.44E-13 |
| ***N*-acyl tryptophan** |  |  |  |  |  |  |
| *N*-palmitoyl tryptophan | PISSR |  |  | PISSR |  |  |
| *N*-stearoyl tryptophan | 8.12E-12 | 2.32E-12 | 7.73E-13 | 7.7E-12 | 2.21E-12 | 7.36E-13 |
| *N*-oleoyl tryptophan | BDL |  |  | BDL |  |  |
| *N*-linoleoyl tryptophan | BDL |  |  | BDL |  |  |
| *N*-arachidonoyl tryptophan | BDL |  |  | BDL |  |  |
| *N*-docosahexaenoyl tryptophan | BDL |  |  | BDL |  |  |
| ***N*-acyl tyrosine** |  |  |  |  |  |  |
| *N*-palmitoyl tyrosine | 1.55E-12 | 9.25E-13 | 3.08E-13 | 1.52E-12 | 4.7E-13 | 1.57E-13 |
| *N*-stearoyl tyrosine | PISSR |  |  | PISSR |  |  |
| *N-*oleoyl tyrosine | PISSR |  |  | PISSR |  |  |
| *N*-linoleoyl tyrosine | PISSR |  |  | PISSR |  |  |
| *N*-arachidonoyl tyrosine | PISSR |  |  | PISSR |  |  |
| *N*-docosahexaenoyl tyrosine | BDL |  |  | BDL |  |  |
| ***N*-acyl valine** |  |  |  |  |  |  |
| *N*-palmitoyl valine | 1.23E-12 | 3.57E-13 | 1.19E-13 | 1.65E-12 | 6.31E-13 | 2.1E-13 |
| *N*- stearoyl valine | 1.36E-12 | 4.7E-13 | 1.57E-13 | 1.41E-12 | 3.98E-13 | 1.33E-13 |
| *N*-oleoyl valine | 3.85E-13 | 1.2E-13 | 4E-14 | 3.31E-13 | 1.35E-13 | 4.51E-14 |
| *N*-nervonoyl valine | BDL |  |  | BDL |  |  |
| *N*-linoleoyl valine | PISSR |  |  | PISSR |  |  |
| *N*-docosahexaenoyl valine | PISSR |  |  | PISSR |  |  |
| **2-acyl glycerols** |  |  |  |  |  |  |
| 2-palmitoyl glycerol | 6.16E-10 | 1.03E-10 | 3.42E-11 | 7.93E-10 | 1.24E-10 | 4.13E-11 |
| 2-oleoyl glycerol | 1.37E-09 | 5.14E-10 | 1.71E-10 | 7.45E-10 | 2.11E-10 | 7.04E-11 |
| 2-linoleoyl glycerol | 8.57E-11 | 1.3E-11 | 4.33E-12 | 5.43E-11 | 1.42E-11 | 4.72E-12 |
| 2-arachidonoyl glycerol | 1.65E-09 | 6.96E-10 | 2.32E-10 | 6.64E-10 | 1.59E-10 | 5.31E-11 |
| **Free Fatty Acids** |  |  |  |  |  |  |
| Oleic acid | 1.04E-09 | 7.94E-11 | 2.65E-11 | 7.46E-10 | 8.65E-11 | 2.88E-11 |
| Linoleic acid | 3.35E-10 | 3.89E-11 | 1.3E-11 | 2.5E-10 | 3.4E-11 | 1.13E-11 |
| Arachidonic acid | 3.95E-09 | 4.35E-10 | 1.45E-10 | 3.33E-09 | 3.62E-10 | 1.21E-10 |
| **PhosphoLEA** |  |  |  |  |  |  |
| PhosphoLEA | 4.41E-11 | 6.37E-12 | 2.12E-12 | 6.47E-11 | 8.9E-12 | 2.97E-12 |
| **Prostaglandins** |  |  |  |  |  |  |
| PGE_2_ | 3.09E-10 | 4.85E-11 | 1.62E-11 | 2.42E-10 | 3.75E-11 | 1.25E-11 |
| PGF_2α_ | 2.74E-10 | 3.21E-11 | 1.07E-11 | 3.47E-10 | 4.63E-11 | 1.54E-11 |
| 6-ketoPGF_1α_ | 1.8E-11 | 5.15E-12 | 1.72E-12 | 1.71E-11 | 4.51E-12 | 1.5E-12 |
| **THC/CP/CBD** |  |  |  |  |  |  |
| Cannabidiol | BDL |  |  | BDL |  |  |
| CP 55,940 | BDL |  |  | 5.31E-11 | 4.16E-12 | 1.39E-12 |
| THC | BDL |  |  | BDL |  |  |
| **THC Metabolites** |  |  |  |  |  |  |
| 11-nor-9-carboxyTHC | BDL |  |  | BDL |  |  |
| 11-OH-THC | BDL |  |  | BDL |  |  |

**Supplemental Table 12.** List of lipids in the hypothalamus significantly affected by 3mg/kg CP 55,940: PND 35

| PND 35 Hypothalamus Significant Differences in One-Way ANOVA | | | | |
| --- | --- | --- | --- | --- |
| Lipid | F | p | Direction (relative to Veh) | Magnitude (x Veh level) |
| *N-*oleoyl alanine | 12.67 | .003 | ↓ | 0.84 |
| *N-*linoleoyl alanine | 15.86 | .001 | ↓ | 0.77 |
| *N*-arachidonoyl alanine | 151.40 | .000 | ↓ | 0.55 |
| *N*-palmitoyl ethanolamine | 18.00 | .001 | ↓ | 0.66 |
| *N*-stearoyl ethanolamine | 10.50 | .005 | ↓ | 0.73 |
| *N*-oleoyl ethanolamine | 9.15 | .008 | ↓ | 0.65 |
| *N*-arachidonoyl ethanolamine | 10.47 | .005 | ↓ | 0.76 |
| *N*-docosahexaenoyl ethanolamine | 42.78 | .000 | ↓ | 0.59 |
| *N*-oleoyl GABA | 5.26 | .036 | ↓ | 0.87 |
| *N*-arachidonoyl GABA | 43.98 | .000 | ↓ | 0.74 |
| *N*-docosahexaenoyl GABA | 72.79 | .000 | ↓ | 0.68 |
| *N*-arachidonoyl glycine | 8.53 | .010 | ↓ | 0.70 |
| *N*-palmitoyl leucine | 23.30 | .000 | ↑ | 1.30 |
| *N*-arachidonoyl phenylalanine | 3.58 | .078 | ↓ | 0.86 |
| *N*-arachidonoyl taurine | 29.45 | .000 | ↓ | 0.70 |
| 2-palmitoyl glycerol | 10.90 | .005 | ↑ | 1.29 |
| 2-oleoyl glycerol | 11.46 | .004 | ↓ | 0.55 |
| 2-linoleoyl glycerol | 24.13 | .000 | ↓ | 0.63 |
| 2-arachidonoyl glycerol | 17.26 | .001 | ↓ | 0.39 |
| Oleic acid | 55.09 | .000 | ↓ | 0.72 |
| Linoleic acid | 24.54 | .000 | ↓ | 0.75 |
| Arachidonic acid | 10.77 | .005 | ↓ | 0.85 |
| phoshoLEA | 31.83 | .000 | ↑ | 1.47 |
| PGE_2_ | 10.68 | .005 | ↓ | 0.78 |
| PGF_2α_ | 15.21 | .001 | ↑ | 1.27 |
| CP 55,940 | 1464.77 | .000 | ↑ | infinite |

**Supplemental Table 13.** Lipid levels in the midbrain of PND 35 WT adolescent female mice treated with Vehicle or 3 mg/kg CP 55,940

|  | Midbrain | | | | | | |
| --- | --- | --- | --- | --- | --- | --- | --- |
|  | Vehicle | | | 3mg/kg CP 55,940 | | | |
| ***N*-acyl alanine** | Mean | Std Dev | Std Error | | Mean | Std Dev | Std Error |
| *N*-palmitoyl alanine | 1.18E-11 | 3.45E-12 | 1.15E-12 | | 1.05E-11 | 3.77E-12 | 1.26E-12 |
| *N*-stearoyl alanine | 1.16E-11 | 1.82E-12 | 6.06E-13 | | 9.99E-12 | 2.29E-12 | 7.64E-13 |
| *N*-oleoyl alanine | 7.47E-12 | 1.01E-12 | 3.38E-13 | | 7.02E-12 | 1.55E-12 | 5.17E-13 |
| *N*-linoleoyl alanine | 1.69E-12 | 4.02E-13 | 1.34E-13 | | 1.52E-12 | 5.09E-13 | 1.7E-13 |
| *N*-arachidonoyl alanine | 1.03E-11 | 1.32E-12 | 4.41E-13 | | 8.2E-12 | 1.01E-12 | 3.36E-13 |
| *N*-docosahexaenoyl alanine | 4.6E-12 | 8.52E-13 | 2.84E-13 | | 3.78E-12 | 1.02E-12 | 3.4E-13 |
| ***N*-acyl dopamine** |  |  |  | |  |  |  |
| *N*-oleoyl dopamine | BDL |  |  | | BDL |  |  |
| *N*-arachidonoyl dopamine | BDL |  |  | | BDL |  |  |
| ***N*-acyl ethanolamine** |  |  |  | |  |  |  |
| *N*-palmitoyl ethanolamine | 1.01E-10 | 1.82E-11 | 6.43E-12 | | 9.08E-11 | 8.11E-12 | 2.7E-12 |
| *N*-stearoyl ethanolamine | 2.13E-11 | 5.18E-12 | 1.83E-12 | | 1.75E-11 | 4.28E-12 | 1.43E-12 |
| *N*-oleoyl ethanolamine | 2.28E-10 | 3.85E-11 | 1.36E-11 | | 1.94E-10 | 1.69E-11 | 5.62E-12 |
| *N*-linoleoyl ethanolamine | 3.05E-11 | 7.52E-12 | 2.51E-12 | | 3.78E-11 | 8.2E-12 | 2.73E-12 |
| *N*-arachidonoyl ethanolamine | 1.89E-11 | 3.5E-12 | 1.17E-12 | | 2.18E-11 | 4.07E-12 | 1.36E-12 |
| *N*-docosahexaenoyl ethanolamine | 3.72E-11 | 7.05E-12 | 2.35E-12 | | 3.99E-11 | 9.76E-12 | 3.25E-12 |
| ***N*-acyl GABA** |  |  |  | |  |  |  |
| *N*-palmitoyl GABA | 9.11E-12 | 1.77E-12 | 5.89E-13 | | 1.05E-11 | 2.77E-12 | 9.25E-13 |
| *N*-stearoyl GABA | 7.71E-12 | 1.63E-12 | 5.43E-13 | | 7.82E-12 | 1.41E-12 | 4.7E-13 |
| *N*-oleoyl GABA | 4.92E-12 | 1.03E-12 | 3.42E-13 | | 4E-12 | 9.2E-13 | 3.07E-13 |
| *N*-linoleoyl GABA | 6.92E-13 | 2.1E-13 | 7.01E-14 | | 7.43E-13 | 2.17E-13 | 7.24E-14 |
| *N*-arachidonoyl GABA | 2.21E-11 | 3.48E-12 | 1.16E-12 | | 1.53E-11 | 2.33E-12 | 7.77E-13 |
| *N*-docosahexaenoyl GABA | 2.89E-12 | 4.53E-13 | 1.51E-13 | | 2.08E-12 | 3.7E-13 | 1.23E-13 |
| ***N*-acyl glycine** |  |  |  | |  |  |  |
| *N*-palmitoyl glycine | 4.51E-11 | 2.64E-12 | 8.8E-13 | | 4.59E-11 | 4.46E-12 | 1.49E-12 |
| *N*-stearoyl glycine | 2.23E-11 | 2.75E-12 | 9.17E-13 | | 2.16E-11 | 3.5E-12 | 1.17E-12 |
| *N*-oleoyl glycine | 2.35E-11 | 2.39E-12 | 7.98E-13 | | 2.31E-11 | 2.29E-12 | 7.62E-13 |
| *N*-linoleoyl glycine | 4.83E-12 | 6.14E-13 | 2.05E-13 | | 4.58E-12 | 8.19E-13 | 2.73E-13 |
| *N*-arachidonoyl glycine | 4.99E-11 | 4.03E-12 | 1.34E-12 | | 4.42E-11 | 4.53E-12 | 1.51E-12 |
| *N*-docosahexaenoyl glycine | 2.12E-11 | 1.81E-12 | 6.02E-13 | | 1.87E-11 | 2.54E-12 | 8.46E-13 |
| ***N*-acyl leucine** |  |  |  | |  |  |  |
| *N*-palmitoyl leucine | 2.3E-12 | 2.37E-13 | 7.91E-14 | | 3.23E-12 | 3.34E-13 | 1.11E-13 |
| *N*-stearoyl leucine | 1.11E-12 | 1.48E-13 | 4.94E-14 | | 1.07E-12 | 9.68E-14 | 3.23E-14 |
| *N*-oleoyl leucine | 1.46E-12 | 2.07E-13 | 6.92E-14 | | 1.53E-12 | 1.98E-13 | 6.59E-14 |
| *N*-linoleoyl leucine | 2.97E-13 | 6.7E-14 | 2.23E-14 | | 3.56E-13 | 9.66E-14 | 3.22E-14 |
| *N*-docosahexaenoyl leucine | 5.31E-13 | 4.42E-14 | 1.47E-14 | | 7.41E-13 | 1.3E-13 | 4.33E-14 |
| ***N*-acyl methionine** |  |  |  | |  |  |  |
| *N*-palmitoyl methionine | 2.41E-12 | 2.8E-13 | 9.35E-14 | | 2.56E-12 | 3.35E-13 | 1.12E-13 |
| *N*-stearoyl methionine | 2.36E-12 | 1.98E-13 | 6.59E-14 | | 2.71E-12 | 3.1E-13 | 1.03E-13 |
| *N*-oleoyl methionine | 1.28E-12 | 2.73E-13 | 9.11E-14 | | 1.46E-12 | 2.93E-13 | 9.78E-14 |
| *N*-linoleoyl methionine | PISSR |  |  | | PISSR |  |  |
| *N*-arachidonoyl methionine | 1.68E-12 | 2.64E-13 | 8.81E-14 | | 1.48E-12 | 5.08E-13 | 1.69E-13 |
| *N*-docosahexaenoyl methionine | 6.13E-13 | 1.41E-13 | 4.71E-14 | | 4.7E-13 | 1.85E-13 | 6.15E-14 |
| ***N*-acyl phenylalanine** |  |  |  | |  |  |  |
| *N*-palmitoyl phenylalanine | 2.31E-12 | 2.02E-13 | 6.74E-14 | | 3.04E-12 | 3.36E-13 | 1.12E-13 |
| *N*-stearoyl phenylalanine | 2.21E-12 | 1.95E-13 | 6.51E-14 | | 2.34E-12 | 1.5E-13 | 4.99E-14 |
| *N*-oleoyl phenylalanine | 1.44E-12 | 1.33E-13 | 4.44E-14 | | 1.6E-12 | 1.13E-13 | 3.76E-14 |
| *N*-linoleoyl phenylalanine | 2E-13 | 5.65E-14 | 1.88E-14 | | 2.21E-13 | 5.64E-14 | 1.88E-14 |
| *N*-arachidonoyl phenylalanine | 1.48E-12 | 1.79E-13 | 5.97E-14 | | 1.53E-12 | 2.2E-13 | 7.32E-14 |
| *N*-docosahexaenoyl phenylalanine | 1.14E-12 | 1.81E-13 | 6.02E-14 | | 1.08E-12 | 1.98E-13 | 6.59E-14 |

**Supplemental Table 13: Continued**

|  | Midbrain | | | | | |
| --- | --- | --- | --- | --- | --- | --- |
|  | Vehicle | | | 3mg/kg CP 55,940 | | |
| ***N*-acyl proline** | Mean | Std Dev | Std Error | Mean | Std Dev | Std Error |
| *N*-palmitoyl proline | 1.01E-12 | 4.16E-13 | 1.39E-13 | 9.03E-13 | 1.74E-13 | 5.8E-14 |
| *N*-stearoyl proline | 4.51E-13 | 1.64E-13 | 5.48E-14 | 3.86E-13 | 9.84E-14 | 3.28E-14 |
| *N-*oleoyl proline | PISSR |  |  | PISSR |  |  |
| *N*-linoleoyl proline | PISSR |  |  | PISSR |  |  |
| *N*-arachidonoyl proline | PISSR |  |  | PISSR |  |  |
| *N*-docosahexaenoyl proline | BDL |  |  | BDL |  |  |
| ***N*-acyl serine** |  |  |  |  |  |  |
| *N*-palmitoyl serine | 1.02E-10 | 6.25E-12 | 2.08E-12 | 9.45E-11 | 1.24E-11 | 4.14E-12 |
| *N*-stearoyl serine | 3.63E-11 | 3.32E-12 | 1.11E-12 | 3.54E-11 | 2.75E-12 | 9.15E-13 |
| *N*-oleoyl serine | 3E-10 | 4.53E-11 | 1.51E-11 | 2.91E-10 | 3.25E-11 | 1.08E-11 |
| *N*-linoleoyl serine | 5.37E-11 | 7.15E-12 | 2.38E-12 | 5.04E-11 | 6.31E-12 | 2.1E-12 |
| *N*-arachidonoyl serine | 8.27E-12 | 7.2E-13 | 2.4E-13 | 6.81E-12 | 1.02E-12 | 3.39E-13 |
| *N*-docosahexaenoyl serine | 3.1E-11 | 3.07E-12 | 1.02E-12 | 2.39E-11 | 4.08E-12 | 1.36E-12 |
| ***N*-acyl taurine** |  |  |  |  |  |  |
| *N*-arachidonoyl taurine | 4.42E-11 | 5.92E-12 | 1.97E-12 | 3.84E-11 | 3.4E-12 | 1.13E-12 |
| ***N*-acyl tryptophan** |  |  |  |  |  |  |
| *N*-palmitoyl tryptophan | 4.2E-13 | 7.43E-14 | 2.48E-14 | 4.42E-13 | 4.09E-14 | 1.36E-14 |
| *N*-stearoyl tryptophan | 7.16E-13 | 1.5E-13 | 4.98E-14 | 7E-13 | 1.13E-13 | 3.77E-14 |
| *N*-oleoyl tryptophan | 1.63E-13 | 2.98E-14 | 9.92E-15 | 1.39E-13 | 4.21E-14 | 1.4E-14 |
| *N*-linoleoyl tryptophan | BDL |  |  | BDL |  |  |
| *N*-arachidonoyl tryptophan | PISSR |  |  | PISSR |  |  |
| *N*-docosahexaenoyl tryptophan | PISSR |  |  | PISSR |  |  |
| ***N*-acyl tyrosine** |  |  |  |  |  |  |
| *N*-palmitoyl tyrosine | 2.04E-12 | 2.18E-13 | 7.27E-14 | 2.19E-12 | 3.29E-13 | 1.1E-13 |
| *N*-stearoyl tyrosine | 2.81E-13 | 4.41E-14 | 1.47E-14 | 2.3E-13 | 6.63E-14 | 2.21E-14 |
| *N-*oleoyl tyrosine | 1.13E-12 | 2.17E-13 | 7.24E-14 | 1.05E-12 | 2.44E-13 | 8.15E-14 |
| *N*-linoleoyl tyrosine | PISSR |  |  | PISSR |  |  |
| *N*-arachidonoyl tyrosine | 1.85E-12 | 2.68E-13 | 8.92E-14 | 1.5E-12 | 3.46E-13 | 1.15E-13 |
| *N*-docosahexaenoyl tyrosine | 2.03E-12 | 2.87E-13 | 9.56E-14 | 1.6E-12 | 2.88E-13 | 9.61E-14 |
| ***N*-acyl valine** |  |  |  |  |  |  |
| *N*-palmitoyl valine | 1.56E-12 | 2E-13 | 6.67E-14 | 2.86E-12 | 5.08E-13 | 1.69E-13 |
| *N*- stearoyl valine | 9.37E-13 | 1.06E-13 | 3.52E-14 | 1.32E-12 | 3E-13 | 9.99E-14 |
| *N*-oleoyl valine | 9.34E-13 | 1.15E-13 | 3.82E-14 | 1.38E-12 | 2.35E-13 | 7.84E-14 |
| *N*-nervonoyl valine | BDL |  |  | BDL |  |  |
| *N*-linoleoyl valine | PISSR |  |  | PISSR |  |  |
| *N*-docosahexaenoyl valine | 1.95E-13 | 4.75E-14 | 1.58E-14 | 3.26E-13 | 1.04E-13 | 3.48E-14 |
| **2-acyl glycerols** |  |  |  |  |  |  |
| 2-palmitoyl glycerol | 4.37E-10 | 1.45E-10 | 4.83E-11 | 4.69E-10 | 1.41E-10 | 4.7E-11 |
| 2-oleoyl glycerol | 1.9E-09 | 4.96E-10 | 1.65E-10 | 1.88E-09 | 4.95E-10 | 1.65E-10 |
| 2-linoleoyl glycerol | 2.08E-10 | 7.15E-11 | 2.38E-11 | 1.59E-10 | 2.86E-11 | 9.53E-12 |
| 2-arachidonoyl glycerol | 4.27E-10 | 9.63E-11 | 3.21E-11 | 3.74E-10 | 6.34E-11 | 2.11E-11 |
| **Free Fatty Acids** |  |  |  |  |  |  |
| Oleic acid | 1.53E-09 | 1.97E-10 | 6.58E-11 | 1.55E-09 | 2.28E-10 | 7.61E-11 |
| Linoleic acid | 5.16E-10 | 5.55E-11 | 1.85E-11 | 5.14E-10 | 5.3E-11 | 1.77E-11 |
| Arachidonic acid | 1.86E-09 | 1.22E-10 | 4.06E-11 | 1.63E-09 | 1.31E-10 | 4.36E-11 |
| **PhosphoLEA** |  |  |  |  |  |  |
| PhosphoLEA | 1.11E-11 | 2.23E-12 | 7.42E-13 | 1.57E-11 | 3.88E-12 | 1.29E-12 |
| **Prostaglandins** |  |  |  |  |  |  |
| PGE_2_ | 5.22E-10 | 2.7E-11 | 8.98E-12 | 5.98E-10 | 6.92E-11 | 2.31E-11 |
| PGF_2α_ | 4.57E-10 | 4.88E-11 | 1.63E-11 | 5.59E-10 | 4.72E-11 | 1.57E-11 |
| 6-ketoPGF_1α_ | 2.43E-11 | 2.75E-12 | 9.18E-13 | 2.09E-11 | 3.36E-12 | 1.12E-12 |
| **THC/CP/CBD** |  |  |  |  |  |  |
| Cannabidiol | BDL |  |  | BDL |  |  |
| CP 55,940 | BDL |  |  | 7.91E-11 | 5.22E-12 | 1.74E-12 |
| THC | BDL |  |  | BDL |  |  |
| **THC Metabolites** |  |  |  |  |  |  |
| 11-nor-9-carboxyTHC | BDL |  |  | BDL |  |  |
| 11-OH-THC | BDL |  |  | BDL |  |  |

**Supplemental Table 14:** List of lipids in the PND 35 midbrain significantly affected by 3mg/kg CP 55,940

| Midbrain Significant Differences in One-Way ANOVA | | | | |
| --- | --- | --- | --- | --- |
| Lipid | F | p | Direction (rel to Veh) | Magnitude (x Veh level) |
| *N*-arachidonoyl alanine | 15.04 | .001 | ↓ | 0.79 |
| *N*-docosahexaenoyl alanine | 3.36 | .086 | ↓ | 0.82 |
| *N*-oleoyl ethanolamine | 6.05 | .027 | ↓ | 0.85 |
| *N*-linoleoyl ethanolamine | 3.83 | .068 | ↑ | 1.24 |
| *N*-oleoyl GABA | 3.98 | .064 | ↓ | 0.81 |
| *N*-arachidonoyl GABA | 23.37 | .000 | ↓ | 0.69 |
| *N*-docosahexaenoyl GABA | 17.51 | .001 | ↓ | 0.72 |
| *N*-arachidonoyl glycine | 7.82 | .013 | ↓ | 0.89 |
| *N*-docosahexaenoyl glycine | 5.66 | .030 | ↓ | 0.88 |
| *N*-palmitoyl leucine | 46.19 | .000 | ↑ | 1.40 |
| *N*-docosahexaenoyl leucine | 21.01 | .000 | ↑ | 1.40 |
| *N*-stearoyl methionine | 8.17 | .001 | ↑ | 1.15 |
| *N*-docosahexaenoyl methionine | 3.44 | .082 | ↓ | 0.77 |
| *N*-palmitoyl phenylalanine | 30.96 | .000 | ↑ | 1.31 |
| *N*-oleoyl phenylalanine | 7.79 | .013 | ↑ | 1.11 |
| *N*-arachidonoyl serine | 12.38 | .003 | ↓ | 0.82 |
| *N*-docosahexaenoyl serine | 17.57 | .001 | ↓ | 0.77 |
| *N*-arachidonoyl taurine | 6.37 | .023 | ↓ | 0.87 |
| *N*-stearoyl tyrosine | 3.61 | .075 | ↓ | 0.82 |
| *N*-arachidonoyl tyrosine | 5.82 | .028 | ↓ | 0.81 |
| *N*-docosahexaenoyl tyrosine | 10.03 | .006 | ↓ | 0.79 |
| *N*-palmitoyl valine | 51.32 | .000 | ↑ | 1.83 |
| *N*-stearoyl valine | 12.84 | .002 | ↑ | 1.40 |
| *N*-oleoyl valine | 26.63 | .000 | ↑ | 1.48 |
| *N*-docosahexaenoyl valine | 11.77 | .003 | ↑ | 1.67 |
| 2-linoleoyl glycerol | 3.71 | .072 | ↓ | 0.76 |
| Arachidonic acid | 15.34 | .001 | ↓ | 0.88 |
| phoshoLEA | 9.53 | .007 | ↑ | 1.41 |
| PGE_2_ | 9.37 | .007 | ↑ | 1.15 |
| PGF_2α_ | 20.24 | .000 | ↑ | 1.22 |
| 6-ketoPGF_1α_ | 5.53 | .023 | ↓ | 0.86 |
| CP 55,940 | 2065.75 | .000 | ↑ | infinite |

**Supplemental Table 15.** Lipid levels in the brainstem of PND35 WT female mice treated with Vehicle or 3 mg/kg CP 55,940

|  | Brainstem | | | | | | |
| --- | --- | --- | --- | --- | --- | --- | --- |
|  | Vehicle | | | 3mg/kg CP 55,940 | | | |
| ***N*-acyl alanine** | Mean | Std Dev | Std Error | | Mean | Std Dev | Std Error |
| *N*-palmitoyl alanine | 9.68E-12 | 3.4E-12 | 1.13E-12 | | 8.91E-12 | 3.22E-12 | 1.07E-12 |
| *N*-stearoyl alanine | 1.32E-11 | 2.31E-12 | 7.71E-13 | | 1.31E-11 | 3.45E-12 | 1.15E-12 |
| *N*-oleoyl alanine | 7.74E-12 | 1.42E-12 | 4.72E-13 | | 7.64E-12 | 1.68E-12 | 5.6E-13 |
| *N*-linoleoyl alanine | 1.38E-12 | 3.28E-13 | 1.09E-13 | | 1.32E-12 | 2.83E-13 | 9.42E-14 |
| *N*-arachidonoyl alanine | 7.12E-12 | 1.12E-12 | 3.73E-13 | | 6.76E-12 | 1.52E-12 | 5.08E-13 |
| *N*-docosahexaenoyl alanine | 4.89E-12 | 8.57E-13 | 2.86E-13 | | 4.95E-12 | 1.32E-12 | 4.41E-13 |
| ***N*-acyl dopamine** |  |  |  | |  |  |  |
| *N*-oleoyl dopamine | BDL |  |  | | BDL |  |  |
| *N*-arachidonoyl dopamine | BDL |  |  | | BDL |  |  |
| ***N*-acyl ethanolamine** |  |  |  | |  |  |  |
| *N*-palmitoyl ethanolamine | 1.36E-10 | 1.98E-11 | 6.6E-12 | | 1.31E-10 | 1.88E-11 | 6.27E-12 |
| *N*-stearoyl ethanolamine | 3.08E-11 | 9.35E-12 | 3.31E-12 | | 3.25E-11 | 7.42E-12 | 2.62E-12 |
| *N*-oleoyl ethanolamine | 2.13E-10 | 4.15E-11 | 1.38E-11 | | 2.04E-10 | 3.86E-11 | 1.29E-11 |
| *N*-linoleoyl ethanolamine | 3.19E-11 | 9.71E-12 | 3.24E-12 | | 3.37E-11 | 5.9E-12 | 1.97E-12 |
| *N*-arachidonoyl ethanolamine | 2.13E-11 | 6.6E-12 | 2.2E-12 | | 2.09E-11 | 3.27E-12 | 1.09E-12 |
| *N*-docosahexaenoyl ethanolamine | 4.46E-11 | 1.11E-11 | 3.69E-12 | | 4.42E-11 | 6.95E-12 | 2.32E-12 |
| ***N*-acyl GABA** |  |  |  | |  |  |  |
| *N*-palmitoyl GABA | 6.98E-12 | 1.58E-12 | 5.28E-13 | | 7.63E-12 | 2.59E-12 | 8.65E-13 |
| *N*-stearoyl GABA | 6.89E-12 | 1.56E-12 | 5.2E-13 | | 6.98E-12 | 1.89E-12 | 6.31E-13 |
| *N*-oleoyl GABA | 5.33E-12 | 1.62E-12 | 5.39E-13 | | 4.65E-12 | 1.45E-12 | 4.83E-13 |
| *N*-linoleoyl GABA | 8.87E-13 | 2.88E-13 | 9.59E-14 | | 8E-13 | 2.54E-13 | 8.47E-14 |
| *N*-arachidonoyl GABA | 1.14E-11 | 2.23E-12 | 7.43E-13 | | 8.01E-12 | 1.71E-12 | 5.7E-13 |
| *N*-docosahexaenoyl GABA | 2.63E-12 | 6.45E-13 | 2.15E-13 | | 2.13E-12 | 6.34E-13 | 2.11E-13 |
| ***N*-acyl glycine** |  |  |  | |  |  |  |
| *N*-palmitoyl glycine | 3.99E-11 | 3.96E-12 | 1.32E-12 | | 4.17E-11 | 7E-12 | 2.33E-12 |
| *N*-stearoyl glycine | 2.22E-11 | 3.99E-12 | 1.33E-12 | | 2.18E-11 | 3.67E-12 | 1.22E-12 |
| *N*-oleoyl glycine | 2.73E-11 | 3.33E-12 | 1.11E-12 | | 2.66E-11 | 4.58E-12 | 1.53E-12 |
| *N*-linoleoyl glycine | 5.99E-12 | 9.4E-13 | 3.13E-13 | | 5.99E-12 | 1.17E-12 | 3.91E-13 |
| *N*-arachidonoyl glycine | 4.82E-11 | 4.26E-12 | 1.42E-12 | | 4.63E-11 | 6.27E-12 | 2.09E-12 |
| *N*-docosahexaenoyl glycine | 2.32E-11 | 1.47E-12 | 4.9E-13 | | 2.18E-11 | 2.77E-12 | 9.23E-13 |
| ***N*-acyl leucine** |  |  |  | |  |  |  |
| *N*-palmitoyl leucine | 1.9E-12 | 1.47E-13 | 4.89E-14 | | 2.63E-12 | 2.96E-13 | 9.88E-14 |
| *N*-stearoyl leucine | 8.39E-13 | 6.1E-14 | 2.03E-14 | | 8.28E-13 | 9.31E-14 | 3.1E-14 |
| *N*-oleoyl leucine | 1.17E-12 | 1.17E-13 | 3.9E-14 | | 1.35E-12 | 1.5E-13 | 5.01E-14 |
| *N*-linoleoyl leucine | 3.08E-13 | 3.58E-14 | 1.19E-14 | | 3.47E-13 | 7.02E-14 | 2.34E-14 |
| *N*-docosahexaenoyl leucine | 4.26E-13 | 6.49E-14 | 2.16E-14 | | 5.85E-13 | 3.85E-14 | 1.28E-14 |
| ***N*-acyl methionine** |  |  |  | |  |  |  |
| *N*-palmitoyl methionine | 1.36E-12 | 3.1E-13 | 1.03E-13 | | 1.43E-12 | 5.22E-13 | 1.74E-13 |
| *N*-stearoyl methionine | 1.7E-12 | 2.43E-13 | 8.1E-14 | | 1.7E-12 | 1.73E-13 | 5.77E-14 |
| *N*-oleoyl methionine | 1.13E-12 | 2E-13 | 6.67E-14 | | 1.16E-12 | 1.26E-13 | 4.21E-14 |
| *N*-linoleoyl methionine | BDL |  |  | | BDL |  |  |
| *N*-arachidonoyl methionine | 9.17E-13 | 2.88E-13 | 9.59E-14 | | 8.72E-13 | 2.15E-13 | 7.18E-14 |
| *N*-docosahexaenoyl methionine | PISSR |  |  | | PISSR |  |  |
| ***N*-acyl phenylalanine** |  |  |  | |  |  |  |
| *N*-palmitoyl phenylalanine | 1.66E-12 | 1.43E-13 | 4.76E-14 | | 2.02E-12 | 2.2E-13 | 7.33E-14 |
| *N*-stearoyl phenylalanine | 1.48E-12 | 1.42E-13 | 4.72E-14 | | 1.48E-12 | 1.51E-13 | 5.02E-14 |
| *N*-oleoyl phenylalanine | 1.01E-12 | 1.2E-13 | 3.99E-14 | | 1.05E-12 | 9.88E-14 | 3.29E-14 |
| *N*-linoleoyl phenylalanine | PISSR |  |  | | PISSR |  |  |
| *N*-arachidonoyl phenylalanine | 9.81E-13 | 1.8E-13 | 6.01E-14 | | 9.69E-13 | 1.34E-13 | 4.47E-14 |
| *N*-docosahexaenoyl phenylalanine | 8.59E-13 | 9.95E-14 | 3.32E-14 | | 8.51E-13 | 1.32E-13 | 4.39E-14 |

**Supplemental Table 15: Continued**

|  | Brainstem | | | | | |
| --- | --- | --- | --- | --- | --- | --- |
|  | Vehicle | | | 3mg/kg CP 55,940 | | |
| ***N*-acyl proline** | Mean | Std Dev | Std Error | Mean | Std Dev | Std Error |
| *N*-palmitoyl proline | 7.52E-13 | 1.58E-13 | 5.26E-14 | 9.02E-13 | 1.88E-13 | 6.27E-14 |
| *N*-stearoyl proline | 4.35E-13 | 6.82E-14 | 2.27E-14 | 4.63E-13 | 1.05E-13 | 3.49E-14 |
| *N-*oleoyl proline | 5.91E-13 | 6.35E-14 | 2.12E-14 | 6.88E-13 | 1.25E-13 | 4.16E-14 |
| *N*-linoleoyl proline | PISSR |  |  | PISSR |  |  |
| *N*-arachidonoyl proline | PISSR |  |  | PISSR |  |  |
| *N*-docosahexaenoyl proline | PISSR |  |  | PISSR |  |  |
| ***N*-acyl serine** |  |  |  |  |  |  |
| *N*-palmitoyl serine | 4.22E-11 | 6.87E-12 | 2.29E-12 | 3.95E-11 | 9.11E-12 | 3.04E-12 |
| *N*-stearoyl serine | 2.13E-11 | 2.26E-12 | 7.53E-13 | 2.01E-11 | 4.11E-12 | 1.37E-12 |
| *N*-oleoyl serine | 1.47E-10 | 1.21E-11 | 4.03E-12 | 1.44E-10 | 2.37E-11 | 7.88E-12 |
| *N*-linoleoyl serine | 2.57E-11 | 3.24E-12 | 1.08E-12 | 2.61E-11 | 4.6E-12 | 1.53E-12 |
| *N*-arachidonoyl serine | 3.47E-12 | 1.04E-12 | 3.48E-13 | 3.04E-12 | 8.69E-13 | 2.9E-13 |
| *N*-docosahexaenoyl serine | 1.35E-11 | 2.33E-12 | 7.77E-13 | 1.42E-11 | 2.64E-12 | 8.81E-13 |
| ***N*-acyl taurine** |  |  |  |  |  |  |
| *N*-arachidonoyl taurine | 2.12E-11 | 2.16E-12 | 7.22E-13 | 1.88E-11 | 2.09E-12 | 6.97E-13 |
| ***N*-acyl tryptophan** |  |  |  |  |  |  |
| *N*-palmitoyl tryptophan | 2.47E-13 | 5E-14 | 1.67E-14 | 2.02E-13 | 4.07E-14 | 1.36E-14 |
| *N*-stearoyl tryptophan | 3.23E-13 | 4.96E-14 | 1.65E-14 | 2.84E-13 | 5.5E-14 | 1.83E-14 |
| *N*-oleoyl tryptophan | PISSR |  |  | PISSR |  |  |
| *N*-linoleoyl tryptophan | BDL |  |  | BDL |  |  |
| *N*-arachidonoyl tryptophan | BDL |  |  | BDL |  |  |
| *N*-docosahexaenoyl tryptophan | PISSR |  |  | PISSR |  |  |
| ***N*-acyl tyrosine** |  |  |  |  |  |  |
| *N*-palmitoyl tyrosine | 7.56E-13 | 1.42E-13 | 4.75E-14 | 8.2E-13 | 6.9E-14 | 2.3E-14 |
| *N*-stearoyl tyrosine | 8.53E-14 | 2.32E-14 | 7.72E-15 | 8.52E-14 | 1.99E-14 | 6.65E-15 |
| *N-*oleoyl tyrosine | 5.23E-13 | 8.07E-14 | 2.69E-14 | 5.15E-13 | 1E-13 | 3.35E-14 |
| *N*-linoleoyl tyrosine | BDL |  |  | BDL |  |  |
| *N*-arachidonoyl tyrosine | 5.56E-13 | 1.61E-13 | 5.37E-14 | 6.37E-13 | 1.48E-13 | 4.93E-14 |
| *N*-docosahexaenoyl tyrosine | 1.08E-12 | 3.39E-13 | 1.13E-13 | 8.89E-13 | 2.19E-13 | 7.31E-14 |
| ***N*-acyl valine** |  |  |  |  |  |  |
| *N*-palmitoyl valine | 8.34E-13 | 1.46E-13 | 4.88E-14 | 1.16E-12 | 1.62E-13 | 5.39E-14 |
| *N*- stearoyl valine | 3.69E-13 | 9.6E-14 | 3.2E-14 | 5.2E-13 | 9.93E-14 | 3.31E-14 |
| *N*-oleoyl valine | 4.29E-13 | 9.31E-14 | 3.1E-14 | 5.41E-13 | 1.33E-13 | 4.43E-14 |
| *N*-nervonoyl valine | BDL |  |  | BDL |  |  |
| *N*-linoleoyl valine | PISSR |  |  | PISSR |  |  |
| *N*-docosahexaenoyl valine | 1.17E-13 | 4.58E-14 | 1.53E-14 | 1.82E-13 | 5.35E-14 | 1.78E-14 |
| **2-acyl glycerols** |  |  |  |  |  |  |
| 2-palmitoyl glycerol | 3.17E-10 | 6.66E-11 | 2.22E-11 | 2.82E-10 | 7.91E-11 | 2.64E-11 |
| 2-oleoyl glycerol | 1.8E-09 | 3.19E-10 | 1.06E-10 | 1.53E-09 | 4.44E-10 | 1.48E-10 |
| 2-linoleoyl glycerol | 1.92E-10 | 3.66E-11 | 1.22E-11 | 1.58E-10 | 3.58E-11 | 1.19E-11 |
| 2-arachidonoyl glycerol | 4.91E-10 | 9.58E-11 | 3.19E-11 | 3.96E-10 | 1.01E-10 | 3.37E-11 |
| **Free Fatty Acids** |  |  |  |  |  |  |
| Oleic acid | 1.6E-09 | 2.74E-10 | 9.12E-11 | 1.63E-09 | 2.92E-10 | 9.74E-11 |
| Linoleic acid | 6.2E-10 | 9.59E-11 | 3.2E-11 | 5.88E-10 | 9.92E-11 | 3.31E-11 |
| Arachidonic acid | 1.69E-09 | 3.23E-10 | 1.08E-10 | 1.65E-09 | 2.35E-10 | 7.84E-11 |
| **PhosphoLEA** |  |  |  |  |  |  |
| PhosphoLEA | 2.27E-11 | 5.3E-12 | 1.77E-12 | 2.06E-11 | 4.27E-12 | 1.42E-12 |
| **Prostaglandins** |  |  |  |  |  |  |
| PGE_2_ | 2.55E-10 | 3.13E-11 | 1.04E-11 | 2.93E-10 | 3.91E-11 | 1.3E-11 |
| PGF_2α_ | 2.18E-10 | 3.49E-11 | 1.16E-11 | 2.4E-10 | 3.27E-11 | 1.09E-11 |
| 6-ketoPGF_1α_ | 1.2E-11 | 2.62E-12 | 8.73E-13 | 1.2E-11 | 1.89E-12 | 6.3E-13 |
| **THC/CP/CBD** |  |  |  |  |  |  |
| Cannabidiol | BDL |  |  | BDL |  |  |
| CP 55,940 | BDL |  |  | 7.04E-11 | 8.68E-12 | 2.89E-12 |
| THC | BDL |  |  | BDL |  |  |
| **THC Metabolites** |  |  |  |  |  |  |
| 11-nor-9-carboxyTHC | BDL |  |  | BDL |  |  |
| 11-OH-THC | BDL |  |  | BDL |  |  |

**Supplemental Table 16.** List of lipids in the brainstem significantly affected by 3 mg/kg CP 55,940: PND35

| Brainstem Significant Differences in One-Way ANOVA | | | | |
| --- | --- | --- | --- | --- |
| Lipid | F | p | Direction (relative to Veh) | Magnitude (x Veh level) |
| *N*-arachidonoyl GABA | 12.82 | .003 | ↓ | 0.70 |
| *N*-palmitoyl leucine | 43.92 | .000 | ↑ | 1.38 |
| *N*-oleoyl leucine | 8.44 | .010 | ↑ | 1.15 |
| *N*-docosahexaenoyl leucine | 39.86 | .000 | ↑ | 1.37 |
| *N*-palmitoyl phenylalanine | 17.28 | .001 | ↑ | 1.22 |
| *N*-palmitoyl proline | 3.34 | .086 | ↑ | 1.20 |
| *N*-oleoyl proline | 4.35 | .053 | ↑ | 1.16 |
| *N*-arachidonoyl taurine | 6.04 | .026 | ↓ | 0.89 |
| *N*-palmitoyl tryptophan | 4.38 | .053 | ↓ | 0.82 |
| *N*-palmitoyl valine | 20.74 | .000 | ↑ | 1.40 |
| *N*-stearoyl valine | 10.74 | .005 | ↑ | 1.41 |
| *N*-oleoyl valine | 4.28 | .055 | ↑ | 1.26 |
| *N*-docosahexaenoyl valine | 7.75 | .013 | ↑ | 1.56 |
| 2-linoleoyl glycerol | 3.98 | .063 | ↓ | 0.82 |
| 2-arachidonoyl glycerol | 4.19 | .058 | ↓ | 0.81 |
| PGE_2_ | 5.28 | .035 | ↑ | 1.15 |
| CP 55,940 | 592.70 | .000 | ↑ | infinite |

**Supplemental Table 17.** Lipid levels in the striatum of PND 50 WT adolescent female mice treated with Vehicle or 3 mg/kg CP 55,940

|  | Striatum | | | | | | |
| --- | --- | --- | --- | --- | --- | --- | --- |
|  | Vehicle | | | 3mg/kg CP 55,940 | | | |
| ***N*-acyl alanine** | Mean | Std Dev | Std Error | | Mean | Std Dev | Std Error |
| *N*-palmitoyl alanine | 4.92E-12 | 2E-12 | 7.07E-13 | | 4.63E-12 | 1.64E-12 | 5.47E-13 |
| *N*-stearoyl alanine | 8.12E-13 | 4.33E-13 | 1.53E-13 | | 6.45E-13 | 3.29E-13 | 1.1E-13 |
| *N*-oleoyl alanine | 1.27E-12 | 4.21E-13 | 1.49E-13 | | 1.2E-12 | 4.05E-13 | 1.35E-13 |
| *N*-linoleoyl alanine | 1.52E-13 | 7.82E-14 | 2.76E-14 | | 1.36E-13 | 8.29E-14 | 2.76E-14 |
| *N*-arachidonoyl alanine | PISSR |  |  | | PISSR |  |  |
| *N*-docosahexaenoyl alanine | PISSR |  |  | | PISSR |  |  |
| ***N*-acyl dopamine** |  |  |  | |  |  |  |
| *N*-oleoyl dopamine | BDL |  |  | | BDL |  |  |
| *N*-arachidonoyl dopamine | BDL |  |  | | BDL |  |  |
| ***N*-acyl ethanolamine** |  |  |  | |  |  |  |
| *N*-palmitoyl ethanolamine | 6.27E-11 | 9.61E-12 | 3.4E-12 | | 6.26E-11 | 9.86E-12 | 3.29E-12 |
| *N*-stearoyl ethanolamine | 2.66E-11 | 5.15E-12 | 1.82E-12 | | 2.79E-11 | 3.86E-12 | 1.29E-12 |
| *N*-oleoyl ethanolamine | 7.49E-11 | 1E-11 | 3.55E-12 | | 6.94E-11 | 1E-11 | 3.35E-12 |
| *N*-linoleoyl ethanolamine | 5.87E-11 | 8.72E-12 | 3.08E-12 | | 5.34E-11 | 7.86E-12 | 2.62E-12 |
| *N*-arachidonoyl ethanolamine | 3.17E-11 | 4.66E-12 | 1.65E-12 | | 2.93E-11 | 4.69E-12 | 1.56E-12 |
| *N*-docosahexaenoyl ethanolamine | 3.33E-11 | 5.24E-12 | 1.85E-12 | | 3.09E-11 | 5.37E-12 | 1.79E-12 |
| ***N*-acyl GABA** |  |  |  | |  |  |  |
| *N*-palmitoyl GABA | 2.68E-12 | 9.52E-13 | 3.37E-13 | | 3.11E-12 | 8.87E-13 | 2.96E-13 |
| *N*-stearoyl GABA | 3.84E-12 | 9.4E-13 | 3.32E-13 | | 3.86E-12 | 1.18E-12 | 3.95E-13 |
| *N*-oleoyl GABA | 1.62E-12 | 3.46E-13 | 1.22E-13 | | 1.25E-12 | 5.94E-13 | 1.98E-13 |
| *N*-linoleoyl GABA | PISSR |  |  | | PISSR |  |  |
| *N*-arachidonoyl GABA | 6E-12 | 6.19E-13 | 2.19E-13 | | 4.29E-12 | 5.53E-13 | 1.84E-13 |
| *N*-docosahexaenoyl GABA | 6.16E-13 | 7.88E-14 | 2.79E-14 | | 4.96E-13 | 4.42E-14 | 1.47E-14 |
| ***N*-acyl glycine** |  |  |  | |  |  |  |
| *N*-palmitoyl glycine | 5.34E-12 | 1.81E-12 | 6.39E-13 | | 5.26E-12 | 2.31E-12 | 7.7E-13 |
| *N*-stearoyl glycine | 4.37E-13 | 1.31E-13 | 4.62E-14 | | 4.91E-13 | 2.04E-13 | 6.81E-14 |
| *N*-oleoyl glycine | 9.15E-13 | 4.56E-13 | 1.61E-13 | | 1.27E-12 | 1.63E-12 | 5.45E-13 |
| *N*-linoleoyl glycine | 5.97E-14 | 3.43E-14 | 1.21E-14 | | 6.89E-14 | 4.98E-14 | 1.66E-14 |
| *N*-arachidonoyl glycine | 8.41E-13 | 6.12E-14 | 2.16E-14 | | 5.51E-13 | 5.8E-14 | 1.93E-14 |
| *N*-docosahexaenoyl glycine | 2.27E-13 | 8.05E-14 | 2.85E-14 | | 1.15E-13 | 3.93E-14 | 1.31E-14 |
| ***N*-acyl leucine** |  |  |  | |  |  |  |
| *N*-palmitoyl leucine | 7.97E-13 | 6.61E-14 | 2.34E-14 | | 1.06E-12 | 1.53E-13 | 5.11E-14 |
| *N*-stearoyl leucine | 1.23E-12 | 2.83E-13 | 1E-13 | | 1.1E-12 | 1.82E-13 | 6.08E-14 |
| *N*-oleoyl leucine | 4.53E-13 | 8.95E-14 | 3.16E-14 | | 4.16E-13 | 1.1E-13 | 3.66E-14 |
| *N*-linoleoyl leucine | 4.78E-14 | 2.54E-14 | 8.97E-15 | | 4.43E-14 | 1.72E-14 | 5.75E-15 |
| *N*-docosahexaenoyl leucine | 9.09E-14 | 3.38E-14 | 1.2E-14 | | 1.19E-13 | 5.73E-14 | 1.91E-14 |
| ***N*-acyl methionine** |  |  |  | |  |  |  |
| *N*-palmitoyl methionine | 1.05E-12 | 3.93E-13 | 1.39E-13 | | 1.12E-12 | 3.59E-13 | 1.2E-13 |
| *N*-stearoyl methionine | 7.96E-13 | 4.54E-13 | 1.6E-13 | | 8.01E-13 | 2.18E-13 | 7.28E-14 |
| *N*-oleoyl methionine | 4.14E-13 | 2.22E-13 | 7.85E-14 | | 3.74E-13 | 1.02E-13 | 3.39E-14 |
| *N*-linoleoyl methionine | PISSR |  |  | | PISSR |  |  |
| *N*-arachidonoyl methionine | PISSR |  |  | | PISSR |  |  |
| *N*-docosahexaenoyl methionine | BDL |  |  | | BDL |  |  |
| ***N*-acyl phenylalanine** |  |  |  | |  |  |  |
| *N*-palmitoyl phenylalanine | 8.52E-13 | 1.43E-13 | 5.07E-14 | | 9.78E-13 | 3.06E-13 | 1.02E-13 |
| *N*-stearoyl phenylalanine | 1.05E-12 | 4.18E-13 | 1.48E-13 | | 1.27E-12 | 2.35E-13 | 7.84E-14 |
| *N*-oleoyl phenylalanine | 5.56E-13 | 1.16E-13 | 4.11E-14 | | 5.89E-13 | 2.36E-13 | 7.88E-14 |
| *N*-linoleoyl phenylalanine | PISSR |  |  | | PISSR |  |  |
| *N*-arachidonoyl phenylalanine | 4.92E-13 | 7.61E-14 | 2.69E-14 | | 3.83E-13 | 5.73E-14 | 1.91E-14 |
| *N*-docosahexaenoyl phenylalanine | 5.01E-13 | 1.4E-13 | 4.95E-14 | | 4.38E-13 | 2.01E-13 | 6.68E-14 |

**Supplemental Table 17: Continued**

|  | Striatum | | | | | |
| --- | --- | --- | --- | --- | --- | --- |
|  | Vehicle | | | 3mg/kg CP 55,940 | | |
| ***N*-acyl proline** | Mean | Std Dev | Std Error | Mean | Std Dev | Std Error |
| *N*-palmitoyl proline | BDL |  |  | BDL |  |  |
| *N*-stearoyl proline | BDL |  |  | BDL |  |  |
| *N-*oleoyl proline | BDL |  |  | BDL |  |  |
| *N*-linoleoyl proline | BDL |  |  | BDL |  |  |
| *N*-arachidonoyl proline | BDL |  |  | BDL |  |  |
| *N*-docosahexaenoyl proline | BDL |  |  | BDL |  |  |
| ***N*-acyl serine** |  |  |  |  |  |  |
| *N*-palmitoyl serine | 2.3E-11 | 4.32E-12 | 1.53E-12 | 2.18E-11 | 2.23E-12 | 7.44E-13 |
| *N*-stearoyl serine | 4.85E-11 | 5.65E-12 | 2E-12 | 5.13E-11 | 8.89E-12 | 2.96E-12 |
| *N*-oleoyl serine | 1.97E-10 | 1.93E-11 | 6.81E-12 | 2.17E-10 | 2.7E-11 | 9E-12 |
| *N*-linoleoyl serine | 1.12E-10 | 1.64E-11 | 5.81E-12 | 1.09E-10 | 1.64E-11 | 5.45E-12 |
| *N*-arachidonoyl serine | PISSR |  |  | PISSR |  |  |
| *N*-docosahexaenoyl serine | PISSR |  |  | PISSR |  |  |
| ***N*-acyl taurine** |  |  |  |  |  |  |
| *N*-arachidonoyl taurine | 2.8E-11 | 2.87E-12 | 1.01E-12 | 2.53E-11 | 2.86E-12 | 9.54E-13 |
| ***N*-acyl tryptophan** |  |  |  |  |  |  |
| *N*-palmitoyl tryptophan | BDL |  |  | BDL |  |  |
| *N*-stearoyl tryptophan | BDL |  |  | BDL |  |  |
| *N*-oleoyl tryptophan | BDL |  |  | BDL |  |  |
| *N*-linoleoyl tryptophan | BDL |  |  | BDL |  |  |
| *N*-arachidonoyl tryptophan | BDL |  |  | BDL |  |  |
| *N*-docosahexaenoyl tryptophan | BDL |  |  | BDL |  |  |
| ***N*-acyl tyrosine** |  |  |  |  |  |  |
| *N*-palmitoyl tyrosine | 3.53E-13 | 3.5E-14 | 1.24E-14 | 4.79E-13 | 7.21E-14 | 2.4E-14 |
| *N*-stearoyl tyrosine | 9.46E-14 | 2.93E-14 | 1.04E-14 | 9.9E-14 | 6.14E-14 | 2.05E-14 |
| *N-*oleoyl tyrosine | 9.65E-14 | 2.88E-14 | 1.02E-14 | 1.31E-13 | 8.63E-14 | 2.88E-14 |
| *N*-linoleoyl tyrosine | BDL |  |  | BDL |  |  |
| *N*-arachidonoyl tyrosine | PISSR |  |  | PISSR |  |  |
| *N*-docosahexaenoyl tyrosine | PISSR |  |  | PISSR |  |  |
| ***N*-acyl valine** |  |  |  |  |  |  |
| *N*-palmitoyl valine | 1.04E-13 | 6.48E-14 | 2.29E-14 | 1.79E-13 | 8.94E-14 | 2.98E-14 |
| *N*- stearoyl valine | PISSR |  |  | PISSR |  |  |
| *N*-oleoyl valine | PISSR |  |  | PISSR |  |  |
| *N*-nervonoyl valine | BDL |  |  | BDL |  |  |
| *N*-linoleoyl valine | PISSR |  |  | PISSR |  |  |
| *N*-docosahexaenoyl valine | BDL |  |  | BDL |  |  |
| **2-acyl glycerols** |  |  |  |  |  |  |
| 2-palmitoyl-*sn*-glycerol | 1.13E-09 | 3.47E-10 | 1.23E-10 | 1.09E-09 | 4.37E-10 | 1.46E-10 |
| 2-oleoyl-*sn*-glycerol | 1.07E-09 | 2.99E-10 | 1.06E-10 | 9.26E-10 | 2.44E-10 | 8.14E-11 |
| 2-linoleoyl-*sn*-glycerol | 1.07E-10 | 2.92E-11 | 1.03E-11 | 8.22E-11 | 2.27E-11 | 7.57E-12 |
| 2-arachidonoyl-*sn*-glycerol | 1.57E-10 | 1.88E-11 | 6.63E-12 | 1.35E-10 | 1.48E-11 | 4.94E-12 |
| **Free Fatty Acids** |  |  |  |  |  |  |
| Oleic acid | 2.74E-10 | 3.21E-11 | 1.13E-11 | 2.17E-10 | 1.75E-11 | 5.83E-12 |
| Linoleic acid | 2.04E-10 | 9.5E-12 | 3.36E-12 | 1.59E-10 | 9.5E-12 | 3.17E-12 |
| Arachidonic acid | 2.88E-10 | 2.08E-11 | 7.35E-12 | 2.35E-10 | 1.35E-11 | 4.51E-12 |
| **PhosphoLEA** |  |  |  |  |  |  |
| PhosphoLEA | PISSR |  |  | PISSR |  |  |
| **Prostaglandins** |  |  |  |  |  |  |
| PGE_2_ | 7.84E-11 | 1.58E-11 | 5.6E-12 | 7.65E-11 | 1.46E-11 | 4.87E-12 |
| PGF_2α_ | 8.03E-11 | 9.72E-12 | 3.44E-12 | 9.33E-11 | 1.11E-11 | 3.72E-12 |
| 6-ketoPGF_1α_ | PISSR |  |  | PISSR |  |  |
| **CBD/CP/THC** |  |  |  |  |  |  |
| Cannabidiol | BDL |  |  | BDL |  |  |
| CP 55,940 | BDL |  |  | 5.87E-11 | 1.08E-11 | 3.59E-12 |
| THC | BDL |  |  | BDL |  |  |
| **THC Metabolites** |  |  |  |  |  |  |
| 11-nor-9-carboxyTHC | BDL |  |  | BDL |  |  |
| 11-OH-THC | BDL |  |  | BDL |  |  |

**Supplemental Table 18.** List of lipids in the striatum significantly affected by 3mg/kg CP 55,940: PND 50

| PND 50 Striatum Significant Differences in One-Way ANOVA | | | | |
| --- | --- | --- | --- | --- |
| Lipid | F | p | Direction (relative to Veh) | Magnitude (x Veh level) |
| *N*-arachidonoyl GABA | 36.45 | .000 | ↓ | 0.71 |
| *N*-docosahexaenoyl GABA | 15.44 | .001 | ↓ | 0.81 |
| *N*-arachidonoyl glycine | 100.25 | .000 | ↓ | 0.65 |
| *N*-docosahexaenoyl glycine | 13.78 | .002 | ↓ | 0.51 |
| *N*-palmitoyl leucine | 20.27 | .000 | ↑ | 1.33 |
| *N*-arachidonoyl phenylalanine | 11.37 | .004 | ↓ | 0.78 |
| *N*-arachidonoyl taurine | 3.54 | .079 | ↓ | 0.91 |
| *N*-palmitoyl tyrosine | 19.94 | .000 | ↑ | 1.35 |
| *N*-palmitoyl valine | 3.80 | .070 | ↑ | 1.72 |
| 2-linoleoyl glycerol | 3.73 | .073 | ↓ | 0.77 |
| 2-arachidonoyl glycerol | 6.89 | .019 | ↓ | 0.86 |
| Oleic acid | 21.36 | .000 | ↓ | 0.79 |
| Linoleic acid | 96.92 | .000 | ↓ | 0.78 |
| Arachidonic acid | 40.27 | .000 | ↓ | 0.81 |
| PGF_2α_ | 6.48 | .022 | ↑ | 1.16 |
| CP 55,940 | 235.50 | .000 | ↑ | infinite |

**Supplemental Table 19.** Lipid levels in the hippocampus of PND 50 WT adolescent female mice treated with Vehicle or 3 mg/kg CP 55,940

|  | Hippocampus | | | | | | |
| --- | --- | --- | --- | --- | --- | --- | --- |
|  | Vehicle | | | 3mg/kg CP 55,940 | | | |
| ***N*-acyl alanine** | Mean | Std Dev | Std Error | | Mean | Std Dev | Std Error |
| *N*-palmitoyl alanine | 2.29E-12 | 9.08E-13 | 3.21E-13 | | 2.35E-12 | 9.59E-13 | 3.2E-13 |
| *N*-stearoyl alanine | 1.98E-12 | 7.04E-13 | 2.49E-13 | | 2.21E-12 | 9.98E-13 | 3.33E-13 |
| *N*-oleoyl alanine | 1.97E-12 | 6.55E-13 | 2.32E-13 | | 2.17E-12 | 8.93E-13 | 2.98E-13 |
| *N*-linoleoyl alanine | PISSR |  |  | | PISSR |  |  |
| *N*-arachidonoyl alanine | 2.73E-12 | 5.15E-13 | 1.82E-13 | | 1.94E-12 | 6.04E-13 | 2.01E-13 |
| *N*-docosahexaenoyl alanine | PISSR |  |  | | PISSR |  |  |
| ***N*-acyl dopamine** |  |  |  | |  |  |  |
| *N*-oleoyl dopamine | BDL |  |  | | BDL |  |  |
| *N*-arachidonoyl dopamine | BDL |  |  | | BDL |  |  |
| ***N*-acyl ethanolamine** |  |  |  | |  |  |  |
| *N*-palmitoyl ethanolamine | 3.68E-11 | 1.06E-11 | 3.76E-12 | | 2.9E-11 | 1.02E-11 | 3.41E-12 |
| *N*-stearoyl ethanolamine | 1.23E-11 | 1.05E-11 | 3.7E-12 | | 1.21E-11 | 1.2E-11 | 3.99E-12 |
| *N*-oleoyl ethanolamine | 1.21E-10 | 4.59E-11 | 1.62E-11 | | 8.7E-11 | 5.37E-11 | 1.79E-11 |
| *N*-linoleoyl ethanolamine | 3.55E-11 | 1.01E-11 | 3.58E-12 | | 2.31E-11 | 1.14E-11 | 3.79E-12 |
| *N*-arachidonoyl ethanolamine | 4.06E-11 | 9.15E-12 | 3.23E-12 | | 2.58E-11 | 8.76E-12 | 2.92E-12 |
| *N*-docosahexaenoyl ethanolamine | 2.61E-11 | 6.56E-12 | 2.32E-12 | | 1.92E-11 | 7.31E-12 | 2.44E-12 |
| ***N*-acyl GABA** |  |  |  | |  |  |  |
| *N*-palmitoyl GABA | 1.64E-11 | 3.3E-12 | 1.17E-12 | | 1.72E-11 | 3.65E-12 | 1.22E-12 |
| *N*-stearoyl GABA | 1.3E-11 | 4.11E-12 | 1.45E-12 | | 1.36E-11 | 3.07E-12 | 1.02E-12 |
| *N*-oleoyl GABA | 1.52E-11 | 2.69E-12 | 9.51E-13 | | 1.53E-11 | 3.21E-12 | 1.07E-12 |
| *N*-linoleoyl GABA | PISSR |  |  | | PISSR |  |  |
| *N*-arachidonoyl GABA | 2.31E-11 | 2.74E-12 | 9.69E-13 | | 2.39E-11 | 3.87E-12 | 1.29E-12 |
| *N*-docosahexaenoyl GABA | PISSR |  |  | | PISSR |  |  |
| ***N*-acyl glycine** |  |  |  | |  |  |  |
| *N*-palmitoyl glycine | 7.6E-12 | 6.31E-12 | 2.23E-12 | | 5.47E-12 | 2.64E-12 | 8.8E-13 |
| *N*-stearoyl glycine | 2.26E-12 | 1.01E-12 | 3.58E-13 | | 2.09E-12 | 1.38E-12 | 4.59E-13 |
| *N*-oleoyl glycine | 1.54E-13 | 1.32E-13 | 4.66E-14 | | 1.25E-13 | 8.46E-14 | 2.82E-14 |
| *N*-linoleoyl glycine | 1.22E-13 | 7.41E-14 | 2.62E-14 | | 8.35E-14 | 5.58E-14 | 1.86E-14 |
| *N*-arachidonoyl glycine | 9.12E-13 | 2.52E-13 | 8.91E-14 | | 4.78E-13 | 1.71E-13 | 5.69E-14 |
| *N*-docosahexaenoyl glycine | 5.93E-14 | 2.86E-14 | 1.01E-14 | | 5.66E-14 | 2.96E-14 | 9.87E-15 |
| ***N*-acyl leucine** |  |  |  | |  |  |  |
| *N*-palmitoyl leucine | 1.09E-12 | 3.59E-13 | 1.27E-13 | | 1.17E-12 | 3.07E-13 | 1.02E-13 |
| *N*-stearoyl leucine | 1.22E-12 | 6.83E-13 | 2.41E-13 | | 1.45E-12 | 6.15E-13 | 2.05E-13 |
| *N*-oleoyl leucine | 1.01E-12 | 2.34E-13 | 8.28E-14 | | 7.72E-13 | 2.57E-13 | 8.57E-14 |
| *N*-linoleoyl leucine | PISSR |  |  | | PISSR |  |  |
| *N*-docosahexaenoyl leucine | PISSR |  |  | | PISSR |  |  |
| ***N*-acyl methionine** |  |  |  | |  |  |  |
| *N*-palmitoyl methionine | 9.88E-12 | 5.06E-12 | 1.79E-12 | | 9.9E-12 | 2.45E-12 | 8.18E-13 |
| *N*-stearoyl methionine | PISSR |  |  | | PISSR |  |  |
| *N*-oleoyl methionine | PISSR |  |  | | PISSR |  |  |
| *N*-linoleoyl methionine | BDL |  |  | | BDL |  |  |
| *N*-arachidonoyl methionine | PISSR |  |  | | PISSR |  |  |
| *N*-docosahexaenoyl methionine | BDL |  |  | | BDL |  |  |
| ***N*-acyl phenylalanine** |  |  |  | |  |  |  |
| *N*-palmitoyl phenylalanine | 6.64E-14 | 3.25E-14 | 1.15E-14 | | 9.31E-14 | 4.13E-14 | 1.38E-14 |
| *N*-stearoyl phenylalanine | 6.72E-14 | 3.67E-14 | 1.3E-14 | | 9.22E-14 | 5.62E-14 | 1.87E-14 |
| *N*-oleoyl phenylalanine | 1.55E-13 | 6.91E-14 | 2.44E-14 | | 1.52E-13 | 6.86E-14 | 2.29E-14 |
| *N*-linoleoyl phenylalanine | PISSR |  |  | | PISSR |  |  |
| *N*-arachidonoyl phenylalanine | PISSR |  |  | | PISSR |  |  |
| *N*-docosahexaenoyl phenylalanine | PISSR |  |  | | PISSR |  |  |

**Supplemental Table 19: Continued**

|  | Hippocampus | | | | | |
| --- | --- | --- | --- | --- | --- | --- |
|  | Vehicle | | | 3mg/kg CP 55,940 | | |
| ***N*-acyl proline** | Mean | Std Dev | Std Error | Mean | Std Dev | Std Error |
| *N*-palmitoyl proline | BDL |  |  | BDL |  |  |
| *N*-stearoyl proline | BDL |  |  | BDL |  |  |
| *N-*oleoyl proline | BDL |  |  | BDL |  |  |
| *N*-linoleoyl proline | BDL |  |  | BDL |  |  |
| *N*-arachidonoyl proline | BDL |  |  | BDL |  |  |
| *N*-docosahexaenoyl proline | BDL |  |  | BDL |  |  |
| ***N*-acyl serine** |  |  |  |  |  |  |
| *N*-palmitoyl serine | 1.38E-11 | 4.61E-12 | 1.63E-12 | 1.51E-11 | 3.06E-12 | 1.02E-12 |
| *N*-stearoyl serine | 3.71E-11 | 9.67E-12 | 3.42E-12 | 3.94E-11 | 5.33E-12 | 1.78E-12 |
| *N*-oleoyl serine | 3.31E-10 | 3.08E-11 | 1.09E-11 | 4.19E-10 | 3.28E-11 | 1.09E-11 |
| *N*-linoleoyl serine | 6.03E-11 | 6.56E-12 | 2.32E-12 | 7.96E-11 | 9.72E-12 | 3.24E-12 |
| *N*-arachidonoyl serine | 1.05E-12 | 2.32E-13 | 8.22E-14 | 5.36E-13 | 6.28E-14 | 2.09E-14 |
| *N*-docosahexaenoyl serine | PISSR |  |  | PISSR |  |  |
| ***N*-acyl taurine** |  |  |  |  |  |  |
| *N*-arachidonoyl taurine | 1.25E-10 | 2.04E-11 | 7.2E-12 | 8.7E-11 | 9.7E-12 | 3.23E-12 |
| ***N*-acyl tryptophan** |  |  |  |  |  |  |
| *N*-palmitoyl tryptophan | BDL |  |  | BDL |  |  |
| *N*-stearoyl tryptophan | BDL |  |  | BDL |  |  |
| *N*-oleoyl tryptophan | BDL |  |  | BDL |  |  |
| *N*-linoleoyl tryptophan | BDL |  |  | BDL |  |  |
| *N*-arachidonoyl tryptophan | BDL |  |  | BDL |  |  |
| *N*-docosahexaenoyl tryptophan | BDL |  |  | BDL |  |  |
| ***N*-acyl tyrosine** |  |  |  |  |  |  |
| *N*-palmitoyl tyrosine | 7.44E-13 | 3.93E-13 | 1.39E-13 | 9.92E-13 | 3.81E-13 | 1.27E-13 |
| *N*-stearoyl tyrosine | PISSR |  |  | PISSR |  |  |
| *N-*oleoyl tyrosine | PISSR |  |  | PISSR |  |  |
| *N*-linoleoyl tyrosine | PISSR |  |  | PISSR |  |  |
| *N*-arachidonoyl tyrosine | 1.66E-12 | 2.38E-13 | 8.42E-14 | 2.3E-12 | 4.31E-13 | 1.44E-13 |
| *N*-docosahexaenoyl tyrosine | PISSR |  |  | PISSR |  |  |
| ***N*-acyl valine** |  |  |  |  |  |  |
| *N*-palmitoyl valine | PISSR |  |  | PISSR |  |  |
| *N*- stearoyl valine | PISSR |  |  | PISSR |  |  |
| *N*-oleoyl valine | PISSR |  |  | PISSR |  |  |
| *N*-nervonoyl valine | BDL |  |  | BDL |  |  |
| *N*-linoleoyl valine | BDL |  |  | BDL |  |  |
| *N*-docosahexaenoyl valine | BDL |  |  | BDL |  |  |
| **2-acyl glycerols** |  |  |  |  |  |  |
| 2-palmitoyl glycerol | 2.14E-10 | 7.24E-11 | 2.56E-11 | 2.49E-10 | 1.07E-10 | 3.56E-11 |
| 2-oleoyl glycerol | 1.75E-09 | 6.79E-10 | 2.4E-10 | 1.71E-09 | 6.7E-10 | 2.23E-10 |
| 2-linoleoyl glycerol | 2.48E-10 | 5.96E-11 | 2.11E-11 | 1.81E-10 | 3.99E-11 | 1.33E-11 |
| 2-arachidonoyl glycerol | 2.34E-09 | 6.57E-10 | 2.32E-10 | 2.77E-09 | 5.86E-10 | 1.95E-10 |
| **Free Fatty Acids** |  |  |  |  |  |  |
| Oleic acid | 5.62E-10 | 1.95E-10 | 6.91E-11 | 5.52E-10 | 2.05E-10 | 6.83E-11 |
| Linoleic acid | 7.16E-10 | 7.37E-11 | 2.6E-11 | 5.21E-10 | 9.52E-11 | 3.17E-11 |
| Arachidonic acid | 2.05E-09 | 5.08E-10 | 1.8E-10 | 1.73E-09 | 5.64E-10 | 1.88E-10 |
| **PhosphoLEA** |  |  |  |  |  |  |
| PhosphoLEA | 1.18E-11 | 4.96E-12 | 1.76E-12 | 9.47E-12 | 3.81E-12 | 1.27E-12 |
| **Prostaglandins** |  |  |  |  |  |  |
| PGE_2_ | 4.57E-10 | 1.92E-11 | 6.78E-12 | 5.28E-10 | 3.86E-11 | 1.29E-11 |
| PGF_2α_ | 3.26E-10 | 4.87E-11 | 1.72E-11 | 3.99E-10 | 3.67E-11 | 1.22E-11 |
| 6-ketoPGF_1α_ | 4.21E-11 | 5.52E-12 | 1.95E-12 | 6.15E-11 | 9.3E-12 | 3.1E-12 |
| **THC/CP/CBD** |  |  |  |  |  |  |
| Cannabidiol | BDL |  |  | BDL |  |  |
| CP 55,940 | BDL |  |  | 1.23E-10 | 1.69E-11 | 5.64E-12 |
| THC | BDL |  |  | BDL |  |  |
| **THC Metabolites** |  |  |  |  |  |  |
| 11-nor-9-carboxyTHC | BDL |  |  | BDL |  |  |
| 11-OH-THC | BDL |  |  | BDL |  |  |

**Supplemental Table 20.** List of lipids in the hippocampus significantly affected by 3 mg/kg CP 55,940: PND50

| PND 50 Hippocampus Significant Differences in One-Way ANOVA | | | | |
| --- | --- | --- | --- | --- |
| Lipid | F | p | Direction (relative to Veh) | Magnitude (x Veh level) |
| *N*-arachidonoyl alanine | 8.24 | .012 | ↓ | 0.71 |
| *N*-linoleoyl ethanolamine | 5.61 | .032 | ↓ | 0.65 |
| *N*-arachidonoyl ethanolamine | 11.57 | .004 | ↓ | 0.64 |
| *N*-docosahexaenoyl ethanolamine | 4.24 | .057 | ↓ | 0.73 |
| *N*-arachidonoyl glycine | 17.69 | .001 | ↓ | 0.52 |
| *N*-oleoyl leucine | 3.87 | .068 | ↓ | 0.77 |
| *N*-oleoyl serine | 32.26 | .000 | ↑ | 1.27 |
| *N*-linoleoyl serine | 22.42 | .000 | ↑ | 1.32 |
| *N*-arachidonoyl serine | 41.18 | .000 | ↓ | 0.51 |
| *N*-arachidonoyl taurine | 25.41 | .000 | ↓ | 0.69 |
| *N*-arachidonoyl tyrosine | 13.73 | .002 | ↑ | 1.38 |
| 2-linoleoyl glycerol | 7.43 | .016 | ↓ | 0.73 |
| Linoleic acid | 21.86 | .000 | ↓ | 0.73 |
| PGE_2_ | 21.83 | .000 | ↑ | 1.15 |
| PGF_2α_ | 12.31 | .000 | ↑ | 1.22 |
| 6-ketoPGF_1α_ | 26.42 | .000 | ↑ | 1.46 |
| CP 55,940 | 419.77 | .000 | ↑ | infinite |

**Supplemental Table 21.** Lipid levels in the cerebellum of PND 50 WT adolescent female mice treated with Vehicle or 3 mg/kg CP 55,940

|  | Cerebellum | | | | | | |
| --- | --- | --- | --- | --- | --- | --- | --- |
|  | Vehicle | | | 3mg/kg CP 55,940 | | | |
| ***N*-acyl alanine** | Mean | Std Dev | Std Error | | Mean | Std Dev | Std Error |
| *N*-palmitoyl alanine | 2.93E-12 | 1.28E-12 | 4.52E-13 | | 2.64E-12 | 1.29E-12 | 4.28E-13 |
| *N*-stearoyl alanine | 1.56E-12 | 9.25E-13 | 3.27E-13 | | 1.16E-12 | 5.06E-13 | 1.69E-13 |
| *N*-oleoyl alanine | 1.16E-12 | 3.74E-13 | 1.32E-13 | | 9.8E-13 | 5.72E-13 | 1.91E-13 |
| *N*-linoleoyl alanine | 6.68E-14 | 6.64E-14 | 2.35E-14 | | 7.79E-14 | 5.84E-14 | 1.95E-14 |
| *N*-arachidonoyl alanine | PISSR |  |  | | PISSR |  |  |
| *N*-docosahexaenoyl alanine | PISSR |  |  | | PISSR |  |  |
| ***N*-acyl dopamine** |  |  |  | |  |  |  |
| *N*-oleoyl dopamine | BDL |  |  | | BDL |  |  |
| *N*-arachidonoyl dopamine | BDL |  |  | | BDL |  |  |
| ***N*-acyl ethanolamine** |  |  |  | |  |  |  |
| *N*-palmitoyl ethanolamine | 5.41E-11 | 8.19E-12 | 2.89E-12 | | 5.07E-11 | 7.98E-12 | 2.66E-12 |
| *N*-stearoyl ethanolamine | 2.52E-11 | 7.63E-12 | 2.7E-12 | | 2.6E-11 | 6.64E-12 | 2.21E-12 |
| *N*-oleoyl ethanolamine | 7.11E-11 | 9.14E-12 | 3.23E-12 | | 6.07E-11 | 9.1E-12 | 3.03E-12 |
| *N*-linoleoyl ethanolamine | 4.83E-11 | 7.33E-12 | 2.59E-12 | | 3.74E-11 | 7.47E-12 | 2.49E-12 |
| *N*-arachidonoyl ethanolamine | 2.78E-11 | 4.54E-12 | 1.61E-12 | | 2.25E-11 | 5.67E-12 | 1.89E-12 |
| *N*-docosahexaenoyl ethanolamine | 3.68E-11 | 6.05E-12 | 2.14E-12 | | 3.09E-11 | 8.09E-12 | 2.7E-12 |
| ***N*-acyl GABA** |  |  |  | |  |  |  |
| *N*-palmitoyl GABA | 5.14E-12 | 1.5E-12 | 5.31E-13 | | 5.13E-12 | 1.77E-12 | 5.92E-13 |
| *N*-stearoyl GABA | 6.49E-12 | 1.84E-12 | 6.51E-13 | | 5.74E-12 | 1.93E-12 | 6.43E-13 |
| *N*-oleoyl GABA | 3.18E-12 | 7.29E-13 | 2.58E-13 | | 2.42E-12 | 5.39E-13 | 1.8E-13 |
| *N*-linoleoyl GABA | 7.21E-13 | 2.88E-13 | 1.02E-13 | | 6.58E-13 | 3.13E-13 | 1.04E-13 |
| *N*-arachidonoyl GABA | 5.23E-12 | 9.2E-13 | 3.25E-13 | | 3.92E-12 | 7.07E-13 | 2.36E-13 |
| *N*-docosahexaenoyl GABA | 1.03E-12 | 2.84E-13 | 1.01E-13 | | 9.21E-13 | 2.86E-13 | 9.54E-14 |
| ***N*-acyl glycine** |  |  |  | |  |  |  |
| *N*-palmitoyl glycine | 3.52E-12 | 1.85E-12 | 6.53E-13 | | 3.72E-12 | 1.99E-12 | 6.63E-13 |
| *N*-stearoyl glycine | 6.13E-13 | 3.31E-13 | 1.17E-13 | | 5.05E-13 | 2.52E-13 | 8.39E-14 |
| *N*-oleoyl glycine | 1.18E-12 | 4.9E-13 | 1.73E-13 | | 1.13E-12 | 6.25E-13 | 2.08E-13 |
| *N*-linoleoyl glycine | 1.19E-13 | 6.06E-14 | 2.14E-14 | | 1.39E-13 | 6.62E-14 | 2.21E-14 |
| *N*-arachidonoyl glycine | 1.61E-12 | 4.98E-13 | 1.76E-13 | | 1.1E-12 | 3.45E-13 | 1.15E-13 |
| *N*-docosahexaenoyl glycine | 5.37E-13 | 3.11E-13 | 1.1E-13 | | 4.01E-13 | 2.15E-13 | 7.16E-14 |
| ***N*-acyl leucine** |  |  |  | |  |  |  |
| *N*-palmitoyl leucine | 6.91E-13 | 4.52E-14 | 1.6E-14 | | 1.13E-12 | 1.42E-13 | 4.74E-14 |
| *N*-stearoyl leucine | 8.79E-13 | 9.37E-14 | 3.31E-14 | | 8.15E-13 | 1.75E-13 | 5.84E-14 |
| *N*-oleoyl leucine | 4.73E-13 | 9.29E-14 | 3.29E-14 | | 5.63E-13 | 1.27E-13 | 4.24E-14 |
| *N*-linoleoyl leucine | 4.07E-14 | 9.09E-15 | 3.21E-15 | | 5.12E-14 | 2.97E-14 | 9.9E-15 |
| *N*-docosahexaenoyl leucine | 1.66E-13 | 4.62E-14 | 1.63E-14 | | 2.06E-13 | 4.81E-14 | 1.6E-14 |
| ***N*-acyl methionine** |  |  |  | |  |  |  |
| *N*-palmitoyl methionine | 7.91E-13 | 3.11E-13 | 1.1E-13 | | 9.79E-13 | 3.9E-13 | 1.3E-13 |
| *N*-stearoyl methionine | 4.03E-13 | 1.38E-13 | 4.87E-14 | | 4.91E-13 | 1.26E-13 | 4.21E-14 |
| *N*-oleoyl methionine | 2E-13 | 6.07E-14 | 2.15E-14 | | 2.55E-13 | 9.33E-14 | 3.11E-14 |
| *N*-linoleoyl methionine | PISSR |  |  | | PISSR |  |  |
| *N*-arachidonoyl methionine | PISSR |  |  | | PISSR |  |  |
| *N*-docosahexaenoyl methionine | PISSR |  |  | | PISSR |  |  |
| ***N*-acyl phenylalanine** |  |  |  | |  |  |  |
| *N*-palmitoyl phenylalanine | 1.08E-12 | 1.78E-13 | 6.31E-14 | | 1.27E-12 | 3.52E-13 | 1.17E-13 |
| *N*-stearoyl phenylalanine | 1.11E-12 | 1.81E-13 | 6.41E-14 | | 1.17E-12 | 2.3E-13 | 7.68E-14 |
| *N*-oleoyl phenylalanine | 7.98E-13 | 1.11E-13 | 3.94E-14 | | 1.01E-12 | 1.73E-13 | 5.78E-14 |
| *N*-linoleoyl phenylalanine | PISSR |  |  | | PISSR |  |  |
| *N*-arachidonoyl phenylalanine | 4.24E-13 | 4.08E-14 | 1.44E-14 | | 2.55E-13 | 4.36E-14 | 1.45E-14 |
| *N*-docosahexaenoyl phenylalanine | 3.47E-13 | 6.38E-14 | 2.26E-14 | | 2.68E-13 | 6.47E-14 | 2.16E-14 |

**Supplemental Table 21: Continued**

|  | Cerebellum | | | | | |
| --- | --- | --- | --- | --- | --- | --- |
|  | Vehicle | | | 3mg/kg CP 55,940 | | |
| ***N*-acyl proline** | Mean | Std Dev | Std Error | Mean | Std Dev | Std Error |
| *N*-palmitoyl proline | BDL |  |  | BDL |  |  |
| *N*-stearoyl proline | BDL |  |  | BDL |  |  |
| *N-*oleoyl proline | BDL |  |  | BDL |  |  |
| *N*-linoleoyl proline | BDL |  |  | BDL |  |  |
| *N*-arachidonoyl proline | BDL |  |  | BDL |  |  |
| *N*-docosahexaenoyl proline | BDL |  |  | BDL |  |  |
| ***N*-acyl serine** |  |  |  |  |  |  |
| *N*-palmitoyl serine | 1.42E-11 | 1.59E-12 | 5.63E-13 | 1.43E-11 | 4.93E-12 | 1.64E-12 |
| *N*-stearoyl serine | 3.22E-11 | 4.77E-12 | 1.69E-12 | 3.32E-11 | 6.84E-12 | 2.28E-12 |
| *N*-oleoyl serine | 1.03E-10 | 1.25E-11 | 4.43E-12 | 1.05E-10 | 2.36E-11 | 7.88E-12 |
| *N*-linoleoyl serine | 5.6E-11 | 1.26E-11 | 4.44E-12 | 5.53E-11 | 1.44E-11 | 4.8E-12 |
| *N*-arachidonoyl serine | 5.46E-12 | 1.16E-12 | 4.09E-13 | 4.25E-12 | 8.71E-13 | 2.9E-13 |
| *N*-docosahexaenoyl serine | 1.13E-11 | 5.41E-12 | 1.91E-12 | 8.95E-12 | 3.22E-12 | 1.07E-12 |
| ***N*-acyl taurine** |  |  |  |  |  |  |
| *N*-arachidonoyl taurine | 2.39E-11 | 3.12E-12 | 1.1E-12 | 1.97E-11 | 2.27E-12 | 7.56E-13 |
| ***N*-acyl tryptophan** |  |  |  |  |  |  |
| *N*-palmitoyl tryptophan | BDL |  |  | BDL |  |  |
| *N*-stearoyl tryptophan | BDL |  |  | BDL |  |  |
| *N*-oleoyl tryptophan | BDL |  |  | BDL |  |  |
| *N*-linoleoyl tryptophan | BDL |  |  | BDL |  |  |
| *N*-arachidonoyl tryptophan | BDL |  |  | BDL |  |  |
| *N*-docosahexaenoyl tryptophan | BDL |  |  | BDL |  |  |
| ***N*-acyl tyrosine** |  |  |  |  |  |  |
| *N*-palmitoyl tyrosine | 5.72E-13 | 1.79E-13 | 6.32E-14 | 6.09E-13 | 1E-13 | 3.33E-14 |
| *N*-stearoyl tyrosine | 1.06E-13 | 2.69E-14 | 9.51E-15 | 1.08E-13 | 3.1E-14 | 1.03E-14 |
| *N-*oleoyl tyrosine | 3.43E-13 | 8.68E-14 | 3.07E-14 | 3.12E-13 | 7.47E-14 | 2.49E-14 |
| *N*-linoleoyl tyrosine | BDL |  |  | BDL |  |  |
| *N*-arachidonoyl tyrosine | 3.65E-13 | 6.28E-14 | 2.22E-14 | 2.66E-13 | 3.22E-14 | 1.07E-14 |
| *N*-docosahexaenoyl tyrosine | 7.7E-13 | 1.52E-13 | 5.38E-14 | 6.76E-13 | 1.89E-13 | 6.29E-14 |
| ***N*-acyl valine** |  |  |  |  |  |  |
| *N*-palmitoyl valine | 1.19E-13 | 4.35E-14 | 1.54E-14 | 1.77E-13 | 6.45E-14 | 2.15E-14 |
| *N*- stearoyl valine | 8.15E-14 | 4.78E-14 | 1.69E-14 | 9.25E-14 | 4.33E-14 | 1.44E-14 |
| *N*-oleoyl valine | 3.24E-14 | 2.01E-14 | 7.11E-15 | 4.78E-14 | 2.56E-14 | 8.53E-15 |
| *N*-nervonoyl valine | BDL |  |  | BDL |  |  |
| *N*-linoleoyl valine | PISSR |  |  | PISSR |  |  |
| *N*-docosahexaenoyl valine | PISSR |  |  | PISSR |  |  |
| **2-acyl glycerols** |  |  |  |  |  |  |
| 2-palmitoyl glycerol | 2.27E-09 | 8.55E-10 | 3.02E-10 | 1.57E-09 | 5.36E-10 | 1.79E-10 |
| 2-oleoyl glycerol | 1.93E-09 | 4.68E-10 | 1.66E-10 | 1.49E-09 | 2.32E-10 | 7.72E-11 |
| 2-linoleoyl glycerol | 2.22E-10 | 5.07E-11 | 1.79E-11 | 1.45E-10 | 2.87E-11 | 9.57E-12 |
| 2-arachidonoyl glycerol | 2.19E-10 | 3.16E-11 | 1.12E-11 | 1.99E-10 | 2.41E-11 | 8.04E-12 |
| **Free Fatty Acids** |  |  |  |  |  |  |
| Oleic acid | 2.84E-10 | 6.86E-11 | 2.42E-11 | 2.36E-10 | 4.28E-11 | 1.43E-11 |
| Linoleic acid | 2.57E-10 | 5.7E-11 | 2.02E-11 | 1.87E-10 | 3.92E-11 | 1.31E-11 |
| Arachidonic acid | 2.44E-10 | 3.78E-11 | 1.34E-11 | 1.8E-10 | 3.81E-11 | 1.27E-11 |
| **PhosphoLEA** |  |  |  |  |  |  |
| PhosphoLEA | PISSR |  |  | PISSR |  |  |
| **Prostaglandins** |  |  |  |  |  |  |
| PGE_2_ | 7.5E-11 | 7.4E-12 | 2.62E-12 | 5.14E-11 | 7.74E-12 | 2.58E-12 |
| PGF_2α_ | 8.44E-11 | 5.42E-12 | 1.92E-12 | 6.11E-11 | 8.06E-12 | 2.69E-12 |
| 6-ketoPGF_1α_ | 4.91E-12 | 5.51E-13 | 1.95E-13 | 6.52E-12 | 9.91E-13 | 3.3E-13 |
| **THC/CP/CBD** |  |  |  |  |  |  |
| Cannabidiol | BDL |  |  | BDL |  |  |
| CP 55,940 | BDL |  |  | 7.57E-11 | 1.36E-11 | 4.55E-12 |
| THC | BDL |  |  | BDL |  |  |
| **THC Metabolites** |  |  |  |  |  |  |
| 11-nor-9-carboxyTHC | BDL |  |  | BDL |  |  |
| 11-OH-THC | BDL |  |  | BDL |  |  |

**Supplemental Table 22.** List of lipids in the cerebellum significantly affected by 3 mg/kg CP 55,940: PND 50

| PND 50 Cerebellum Significant Differences in One-Way ANOVA | | | | |
| --- | --- | --- | --- | --- |
| Lipid | F | p | Direction (relative to Veh) | Magnitude (x Veh level) |
| *N*-oleoyl ethanolamine | 5.51 | .033 | ↓ | 0.85 |
| *N*-linoleoyl ethanolamine | 9.19 | .008 | ↓ | 0.77 |
| *N*-arachidonoyl ethanolamine | 4.50 | .051 | ↓ | 0.81 |
| *N*-oleoyl GABA | 6.01 | .027 | ↓ | 0.76 |
| *N*-arachidonoyl GABA | 10.96 | .005 | ↓ | 0.75 |
| *N*-arachidonoyl glycine | 6.09 | .026 | ↓ | 0.68 |
| *N-*palmitoyl leucine | 68.57 | .000 | ↑ | 1.63 |
| *N*-oleoyl phenylalanine | 8.63 | .010 | ↑ | 1.26 |
| *N*-arachidonoyl phenylalanine | 68.07 | .000 | ↓ | 0.60 |
| *N*-docosahexaenoyl phenylalanine | 6.27 | .024 | ↓ | 0.77 |
| *N*-arachidonoyl serine | 6.01 | .027 | ↓ | 0.78 |
| *N*-arachidonoyl taurine | 10.06 | .006 | ↓ | 0.83 |
| *N*-arachidonoyl tyrosine | 17.21 | .001 | ↓ | 0.73 |
| *N*-palmitoyl valine | 4.63 | .048 | ↑ | 1.49 |
| 2-palmitoyl glycerol | 4.22 | .058 | ↓ | 0.69 |
| 2-oleoyl glycerol | 6.20 | .025 | ↓ | 0.77 |
| 2-linoleoyl glycerol | 15.21 | .001 | ↓ | 0.65 |
| Oleic acid | 3.10 | .098 | ↓ | 0.83 |
| Linoleic acid | 8.85 | .009 | ↓ | 0.73 |
| Arachidonic acid | 12.08 | .003 | ↓ | 0.74 |
| PGE_2_ | 41.18 | .000 | ↓ | 0.68 |
| PGF_2α_ | 47.81 | .000 | ↓ | 0.72 |
| 6-ketoPGF_1α_ | 16.53 | .001 | ↑ | 1.33 |
| CP 55,940 | 244.42 | .000 | ↑ | infinite |

**Supplemental Table 23.** Lipid levels in the thalamus of PND 50 WT adolescent female mice treated with Vehicle or 3 mg/kg CP 55,940

|  | Thalamus | | | | | | |
| --- | --- | --- | --- | --- | --- | --- | --- |
|  | Vehicle | | | 3mg/kg CP 55,940 | | | |
| ***N*-acyl alanine** | Mean | Std Dev | Std Error | | Mean | Std Dev | Std Error |
| *N*-palmitoyl alanine | 3.05E-12 | 1.21E-12 | 4.27E-13 | | 2.52E-12 | 9.7E-13 | 3.23E-13 |
| *N*-stearoyl alanine | 7.11E-13 | 3.67E-13 | 1.3E-13 | | 5.6E-13 | 3.09E-13 | 1.03E-13 |
| *N*-oleoyl alanine | 9.57E-13 | 4.95E-13 | 1.75E-13 | | 7.11E-13 | 2.56E-13 | 8.53E-14 |
| *N*-linoleoyl alanine | PISSR |  |  | | PISSR |  |  |
| *N*-arachidonoyl alanine | PISSR |  |  | | PISSR |  |  |
| *N*-docosahexaenoyl alanine | PISSR |  |  | | PISSR |  |  |
| ***N*-acyl dopamine** |  |  |  | |  |  |  |
| *N*-oleoyl dopamine | BDL |  |  | | BDL |  |  |
| *N*-arachidonoyl dopamine | BDL |  |  | | BDL |  |  |
| ***N*-acyl ethanolamine** |  |  |  | |  |  |  |
| *N*-palmitoyl ethanolamine | 8.45E-11 | 1.65E-11 | 5.84E-12 | | 7.34E-11 | 1.5E-11 | 5.01E-12 |
| *N*-stearoyl ethanolamine | 3.42E-11 | 1.44E-11 | 5.08E-12 | | 3.31E-11 | 1.44E-11 | 4.79E-12 |
| *N*-oleoyl ethanolamine | 1.07E-10 | 2.57E-11 | 9.07E-12 | | 9.26E-11 | 2.64E-11 | 8.81E-12 |
| *N*-linoleoyl ethanolamine | 6.69E-11 | 1.3E-11 | 4.6E-12 | | 5.72E-11 | 1.67E-11 | 5.58E-12 |
| *N*-arachidonoyl ethanolamine | 4.31E-11 | 5.72E-12 | 2.02E-12 | | 3.67E-11 | 6.91E-12 | 2.3E-12 |
| *N*-docosahexaenoyl ethanolamine | 5.8E-11 | 1.03E-11 | 3.65E-12 | | 4.85E-11 | 1.28E-11 | 4.28E-12 |
| ***N*-acyl GABA** |  |  |  | |  |  |  |
| *N*-palmitoyl GABA | 9.83E-12 | 2.15E-12 | 7.59E-13 | | 1.06E-11 | 3.5E-12 | 1.17E-12 |
| *N*-stearoyl GABA | 1.17E-11 | 3.3E-12 | 1.16E-12 | | 1.06E-11 | 4.11E-12 | 1.37E-12 |
| *N*-oleoyl GABA | 5.07E-12 | 1.34E-12 | 4.73E-13 | | 4.02E-12 | 1.39E-12 | 4.63E-13 |
| *N*-linoleoyl GABA | 1.1E-12 | 5.03E-13 | 1.78E-13 | | 6.3E-13 | 1.82E-13 | 6.08E-14 |
| *N*-arachidonoyl GABA | 1.55E-11 | 4.32E-12 | 1.53E-12 | | 1.06E-11 | 3.76E-12 | 1.25E-12 |
| *N*-docosahexaenoyl GABA | 1.67E-12 | 6.65E-13 | 2.35E-13 | | 1.17E-12 | 4.71E-13 | 1.57E-13 |
| ***N*-acyl glycine** |  |  |  | |  |  |  |
| *N*-palmitoyl glycine | 9.85E-12 | 3.75E-12 | 1.32E-12 | | 9.61E-12 | 4.08E-12 | 1.36E-12 |
| *N*-stearoyl glycine | 1.15E-12 | 6.6E-13 | 2.33E-13 | | 1.16E-12 | 1E-12 | 3.35E-13 |
| *N*-oleoyl glycine | 1.89E-12 | 7.36E-13 | 2.6E-13 | | 1.74E-12 | 9.29E-13 | 3.1E-13 |
| *N*-linoleoyl glycine | 1.4E-13 | 1.11E-13 | 3.91E-14 | | 1.48E-13 | 9.3E-14 | 3.1E-14 |
| *N*-arachidonoyl glycine | 3.54E-12 | 7.69E-13 | 2.72E-13 | | 2.22E-12 | 5.27E-13 | 1.76E-13 |
| *N*-docosahexaenoyl glycine | 3.44E-13 | 1.87E-13 | 6.63E-14 | | 2.76E-13 | 1.41E-13 | 4.69E-14 |
| ***N*-acyl leucine** |  |  |  | |  |  |  |
| *N*-palmitoyl leucine | 1.2E-12 | 1E-13 | 3.54E-14 | | 1.53E-12 | 2.54E-13 | 8.48E-14 |
| *N*-stearoyl leucine | 1.13E-12 | 3.14E-13 | 1.11E-13 | | 1.04E-12 | 2.77E-13 | 9.22E-14 |
| *N*-oleoyl leucine | 5.47E-13 | 9.92E-14 | 3.51E-14 | | 5.99E-13 | 1.57E-13 | 5.25E-14 |
| *N*-linoleoyl leucine | 5.5E-14 | 2.68E-14 | 9.48E-15 | | 4.17E-14 | 2.13E-14 | 7.11E-15 |
| *N*-docosahexaenoyl leucine | 1.51E-13 | 6.94E-14 | 2.45E-14 | | 1.83E-13 | 5.7E-14 | 1.9E-14 |
| ***N*-acyl methionine** |  |  |  | |  |  |  |
| *N*-palmitoyl methionine | 1.44E-12 | 4.08E-13 | 1.44E-13 | | 1.27E-12 | 3.42E-13 | 1.14E-13 |
| *N*-stearoyl methionine | 9.29E-13 | 2.7E-13 | 9.55E-14 | | 8.73E-13 | 1.94E-13 | 6.46E-14 |
| *N*-oleoyl methionine | 2.91E-13 | 1.84E-13 | 6.52E-14 | | 2.91E-13 | 1.6E-13 | 5.33E-14 |
| *N*-linoleoyl methionine | PISSR |  |  | | PISSR |  |  |
| *N*-arachidonoyl methionine | PISSR |  |  | | PISSR |  |  |
| *N*-docosahexaenoyl methionine | PISSR |  |  | | PISSR |  |  |
| ***N*-acyl phenylalanine** |  |  |  | |  |  |  |
| *N*-palmitoyl phenylalanine | 1.13E-12 | 1.84E-13 | 6.5E-14 | | 1.34E-12 | 3.81E-13 | 1.27E-13 |
| *N*-stearoyl phenylalanine | 1.28E-12 | 2.42E-13 | 8.57E-14 | | 1.21E-12 | 1.49E-13 | 4.97E-14 |
| *N*-oleoyl phenylalanine | 9.04E-13 | 2.04E-13 | 7.22E-14 | | 9.22E-13 | 1.9E-13 | 6.32E-14 |
| *N*-linoleoyl phenylalanine | PISSR |  |  | | PISSR |  |  |
| *N*-arachidonoyl phenylalanine | 4.59E-13 | 5.63E-14 | 1.99E-14 | | 2.35E-13 | 8.63E-14 | 2.88E-14 |
| *N*-docosahexaenoyl phenylalanine | PISSR |  |  | | PISSR |  |  |

**Supplemental Table 23: Continued**

|  | Thalamus | | | | | |
| --- | --- | --- | --- | --- | --- | --- |
|  | Vehicle | | | 3mg/kg CP 55,940 | | |
| ***N*-acyl proline** | Mean | Std Dev | Std Error | Mean | Std Dev | Std Error |
| *N*-palmitoyl proline | BDL |  |  | BDL |  |  |
| *N*-stearoyl proline | BDL |  |  | BDL |  |  |
| *N-*oleoyl proline | BDL |  |  | BDL |  |  |
| *N*-linoleoyl proline | BDL |  |  | BDL |  |  |
| *N*-arachidonoyl proline | BDL |  |  | BDL |  |  |
| *N*-docosahexaenoyl proline | BDL |  |  | BDL |  |  |
| ***N*-acyl serine** |  |  |  |  |  |  |
| *N*-palmitoyl serine | 3.23E-11 | 6E-12 | 2.12E-12 | 2.63E-11 | 2.92E-12 | 9.72E-13 |
| *N*-stearoyl serine | 4.65E-11 | 7.38E-12 | 2.61E-12 | 4.06E-11 | 5.12E-12 | 1.71E-12 |
| *N*-oleoyl serine | 1.37E-10 | 1.22E-11 | 4.33E-12 | 1.27E-10 | 1.32E-11 | 4.4E-12 |
| *N*-linoleoyl serine | 7.1E-11 | 6.96E-12 | 2.46E-12 | 6.59E-11 | 6.09E-12 | 2.03E-12 |
| *N*-arachidonoyl serine | 9.42E-12 | 4.51E-12 | 1.59E-12 | 9.47E-12 | 3.2E-12 | 1.07E-12 |
| *N*-docosahexaenoyl serine | 2.12E-11 | 4.14E-12 | 1.46E-12 | 1.8E-11 | 4.05E-12 | 1.35E-12 |
| ***N*-acyl taurine** |  |  |  |  |  |  |
| *N*-arachidonoyl taurine | 2.79E-11 | 3.84E-12 | 1.36E-12 | 2.48E-11 | 2.94E-12 | 9.79E-13 |
| ***N*-acyl tryptophan** |  |  |  |  |  |  |
| *N*-palmitoyl tryptophan | BDL |  |  | BDL |  |  |
| *N*-stearoyl tryptophan | BDL |  |  | BDL |  |  |
| *N*-oleoyl tryptophan | BDL |  |  | BDL |  |  |
| *N*-linoleoyl tryptophan | BDL |  |  | BDL |  |  |
| *N*-arachidonoyl tryptophan | BDL |  |  | BDL |  |  |
| *N*-docosahexaenoyl tryptophan | BDL |  |  | BDL |  |  |
| ***N*-acyl tyrosine** |  |  |  |  |  |  |
| *N*-palmitoyl tyrosine | 8.72E-13 | 2.04E-13 | 7.2E-14 | 8.81E-13 | 2.26E-13 | 7.54E-14 |
| *N*-stearoyl tyrosine | 1.35E-13 | 4E-14 | 1.41E-14 | 1.18E-13 | 4.25E-14 | 1.42E-14 |
| *N-*oleoyl tyrosine | 3.67E-13 | 1.51E-13 | 5.33E-14 | 4.03E-13 | 3.7E-14 | 1.23E-14 |
| *N*-linoleoyl tyrosine | PISSR |  |  | PISSR |  |  |
| *N*-arachidonoyl tyrosine | 4.45E-13 | 7.03E-14 | 2.49E-14 | 3.46E-13 | 6.42E-14 | 2.14E-14 |
| *N*-docosahexaenoyl tyrosine | 6.96E-13 | 1.04E-13 | 3.68E-14 | 5.89E-13 | 2.75E-13 | 9.16E-14 |
| ***N*-acyl valine** |  |  |  |  |  |  |
| *N*-palmitoyl valine | 2.01E-13 | 7.53E-14 | 2.66E-14 | 1.92E-13 | 8.15E-14 | 2.72E-14 |
| *N*- stearoyl valine | 1.06E-13 | 5.83E-14 | 2.06E-14 | 1.2E-13 | 7.91E-14 | 2.64E-14 |
| *N*-oleoyl valine | PISSR |  |  | PISSR |  |  |
| *N*-nervonoyl valine | BDL |  |  | BDL |  |  |
| *N*-linoleoyl valine | BDL |  |  | BDL |  |  |
| *N*-docosahexaenoyl valine | BDL |  |  | BDL |  |  |
| **2-acyl glycerols** |  |  |  |  |  |  |
| 2-palmitoyl glycerol | 1.33E-09 | 3.59E-10 | 1.27E-10 | 1.02E-09 | 5.8E-10 | 1.93E-10 |
| 2-oleoyl glycerol | 3.58E-09 | 8.05E-10 | 2.84E-10 | 2.79E-09 | 5.12E-10 | 1.71E-10 |
| 2-linoleoyl glycerol | 3.05E-10 | 9.9E-11 | 3.5E-11 | 2.06E-10 | 5.63E-11 | 1.88E-11 |
| 2-arachidonoyl glycerol | 2.6E-10 | 3.83E-11 | 1.35E-11 | 2.19E-10 | 4.1E-11 | 1.37E-11 |
| **Free Fatty Acids** |  |  |  |  |  |  |
| Oleic acid | 3.84E-10 | 5.49E-11 | 1.94E-11 | 3.18E-10 | 6.34E-11 | 2.11E-11 |
| Linoleic acid | 3.15E-10 | 6.56E-11 | 2.32E-11 | 2.28E-10 | 5.45E-11 | 1.82E-11 |
| Arachidonic acid | 4.29E-10 | 7.27E-11 | 2.57E-11 | 3.32E-10 | 8.39E-11 | 2.8E-11 |
| **PhosphoLEA** |  |  |  |  |  |  |
| PhosphoLEA | PISSR |  |  | PISSR |  |  |
| **Prostaglandins** |  |  |  |  |  |  |
| PGE_2_ | 1.19E-10 | 9.58E-12 | 3.39E-12 | 8.74E-11 | 8.76E-12 | 2.92E-12 |
| PGF_2α_ | 1.56E-10 | 1.79E-11 | 6.33E-12 | 1.3E-10 | 1.86E-11 | 6.2E-12 |
| 6-ketoPGF_1α_ | 7.94E-12 | 9.92E-13 | 3.51E-13 | 1.21E-11 | 1.65E-12 | 5.5E-13 |
| **THC/CP/CBD** |  |  |  |  |  |  |
| Cannabidiol | BDL |  |  | BDL |  |  |
| CP 55,940 | BDL |  |  | 9.93E-11 | 1.63E-11 | 5.44E-12 |
| THC | BDL |  |  | BDL |  |  |
| **THC Metabolites** |  |  |  |  |  |  |
| 11-nor-9-carboxyTHC | BDL |  |  | BDL |  |  |
| 11-OH-THC | BDL |  |  | BDL |  |  |

**Supplemental Table 24.** List of lipids in the thalamus significantly affected by 3 mg/kg CP 55,940: PND 50

| PND 50 Thalamus Significant Differences in One-Way ANOVA | | | | |
| --- | --- | --- | --- | --- |
| Lipid | F | p | Direction (relative to Veh) | Magnitude (x Veh level) |
| *N*-arachidonoyl ethanolamine | 4.25 | .057 | ↓ | 0.85 |
| *N*-linoleoyl GABA | 6.98 | .019 | ↓ | 0.57 |
| *N*-arachidonoyl GABA | 6.31 | .024 | ↓ | 0.68 |
| *N*-docosahexaenoyl GABA | 3.31 | .089 | ↓ | 0.70 |
| *N*-arachidonoyl glycine | 17.37 | .001 | ↓ | 0.63 |
| *N*-palmitoyl leucine | 11.51 | .004 | ↑ | 1.27 |
| *N*-arachidonoyl phenylalanine | 38.91 | .000 | ↓ | 0.51 |
| *N*-palmitoyl serine | 6.95 | .019 | ↓ | 0.82 |
| *N*-stearoyl serine | 3.73 | .072 | ↓ | 0.87 |
| *N*-arachidonoyl taurine | 3.65 | .076 | ↓ | 0.88 |
| *N*-arachidonoyl tyrosine | 9.22 | .008 | ↓ | 0.78 |
| 2-oleoyl glycerol | 5.84 | .029 | ↓ | 0.78 |
| 2-linoleoyl glycerol | 6.61 | .021 | ↓ | 0.68 |
| 2-arachidonoyl glycerol | 4.48 | .051 | ↓ | 0.84 |
| Oleic acid | 5.08 | .040 | ↓ | 0.83 |
| Linoleic acid | 8.84 | .009 | ↓ | 0.73 |
| Arachidonic acid | 6.39 | .023 | ↓ | 0.77 |
| PGE_2_ | 49.41 | .000 | ↓ | 0.74 |
| PGF_2α_ | 8.36 | .011 | ↓ | 0.84 |
| 6-ketoPGF_1α_ | 37.79 | .000 | ↑ | 1.52 |
| CP 55,940 | 293.59 | .000 | ↑ | infinite |

**Supplemental Table 25.** Lipid levels in the cortex of WT PND 50 adolescent female mice treated with Vehicle or 3 mg/kg CP 55,940

|  | Cortex | | | | | | |
| --- | --- | --- | --- | --- | --- | --- | --- |
|  | Vehicle | | | 3mg/kg CP 55,940 | | | |
| ***N*-acyl alanine** | Mean | Std Dev | Std Error | | Mean | Std Dev | Std Error |
| *N*-palmitoyl alanine | 1.54E-12 | 5.41E-13 | 1.91E-13 | | 1.42E-12 | 3.8E-13 | 1.27E-13 |
| *N*-stearoyl alanine | 4.57E-12 | 1.99E-12 | 7.03E-13 | | 3.67E-12 | 1.52E-12 | 5.06E-13 |
| *N*-oleoyl alanine | 1.38E-12 | 2.65E-13 | 9.37E-14 | | 1.52E-12 | 6.81E-13 | 2.27E-13 |
| *N*-linoleoyl alanine | PISSR |  |  | | PISSR |  |  |
| *N*-arachidonoyl alanine | 1.21E-12 | 2.97E-13 | 1.05E-13 | | 9.08E-13 | 1.02E-13 | 3.39E-14 |
| *N*-docosahexaenoyl alanine | PISSR |  |  | | PISSR |  |  |
| ***N*-acyl dopamine** |  |  |  | |  |  |  |
| *N*-oleoyl dopamine | BDL |  |  | | BDL |  |  |
| *N*-arachidonoyl dopamine | BDL |  |  | | BDL |  |  |
| ***N*-acyl ethanolamine** |  |  |  | |  |  |  |
| *N*-palmitoyl ethanolamine | 1.82E-11 | 5.38E-12 | 1.9E-12 | | 1.43E-11 | 3.92E-12 | 1.31E-12 |
| *N*-stearoyl ethanolamine | 3.2E-12 | 1.77E-12 | 6.27E-13 | | 2.8E-12 | 8.03E-13 | 2.68E-13 |
| *N*-oleoyl ethanolamine | 5.6E-11 | 1.04E-11 | 3.67E-12 | | 3.66E-11 | 1.1E-11 | 3.66E-12 |
| *N*-linoleoyl ethanolamine | 2.32E-11 | 7.36E-12 | 2.6E-12 | | 1.61E-11 | 7.36E-12 | 2.45E-12 |
| *N*-arachidonoyl ethanolamine | 1.77E-11 | 4.11E-12 | 1.45E-12 | | 1.23E-11 | 2.92E-12 | 9.72E-13 |
| *N*-docosahexaenoyl ethanolamine | 1.74E-11 | 3.73E-12 | 1.32E-12 | | 1.26E-11 | 3.41E-12 | 1.14E-12 |
| ***N*-acyl GABA** |  |  |  | |  |  |  |
| *N*-palmitoyl GABA | 1.66E-11 | 1.71E-12 | 6.05E-13 | | 1.74E-11 | 2.85E-12 | 9.49E-13 |
| *N*-stearoyl GABA | 1.56E-11 | 1.53E-12 | 5.41E-13 | | 1.55E-11 | 2.18E-12 | 7.26E-13 |
| *N*-oleoyl GABA | 1.44E-11 | 1.53E-12 | 5.43E-13 | | 1.38E-11 | 2.25E-12 | 7.51E-13 |
| *N*-linoleoyl GABA | 7.03E-12 | 1.17E-12 | 4.13E-13 | | 6.54E-12 | 1.15E-12 | 3.82E-13 |
| *N*-arachidonoyl GABA | 2.23E-11 | 1.81E-12 | 6.4E-13 | | 1.93E-11 | 1.89E-12 | 6.29E-13 |
| *N*-docosahexaenoyl GABA | 9.75E-12 | 8.45E-13 | 2.99E-13 | | 9.29E-12 | 1.35E-12 | 4.49E-13 |
| ***N*-acyl glycine** |  |  |  | |  |  |  |
| *N*-palmitoyl glycine | 5.91E-12 | 1.27E-12 | 4.5E-13 | | 6.08E-12 | 1.49E-12 | 4.97E-13 |
| *N*-stearoyl glycine | 1.66E-12 | 9.55E-13 | 3.38E-13 | | 1.45E-12 | 7.59E-13 | 2.53E-13 |
| *N*-oleoyl glycine | 1.53E-12 | 4.81E-13 | 1.7E-13 | | 1.38E-12 | 5.76E-13 | 1.92E-13 |
| *N*-linoleoyl glycine | 9.41E-14 | 2.97E-14 | 1.05E-14 | | 1.08E-13 | 3.94E-14 | 1.31E-14 |
| *N*-arachidonoyl glycine | 3.74E-12 | 1.07E-12 | 3.78E-13 | | 3.26E-12 | 1.14E-12 | 3.79E-13 |
| *N*-docosahexaenoyl glycine | 3.38E-13 | 8.42E-14 | 2.98E-14 | | 3.17E-13 | 1.39E-13 | 4.62E-14 |
| ***N*-acyl leucine** |  |  |  | |  |  |  |
| *N*-palmitoyl leucine | 1.4E-12 | 1.46E-13 | 5.14E-14 | | 1.98E-12 | 2.35E-13 | 7.85E-14 |
| *N*-stearoyl leucine | 2.42E-12 | 3.28E-13 | 1.16E-13 | | 2.21E-12 | 2.97E-13 | 9.92E-14 |
| *N*-oleoyl leucine | 8.09E-13 | 1.6E-13 | 5.64E-14 | | 7.87E-13 | 1.25E-13 | 4.17E-14 |
| *N*-linoleoyl leucine | 7.64E-14 | 3.01E-14 | 1.06E-14 | | 7.72E-14 | 1.97E-14 | 6.57E-15 |
| *N*-docosahexaenoyl leucine | 2.83E-13 | 3.94E-14 | 1.39E-14 | | 3.29E-13 | 9.24E-14 | 3.08E-14 |
| ***N*-acyl methionine** |  |  |  | |  |  |  |
| *N*-palmitoyl methionine | 7.09E-13 | 4.58E-14 | 1.62E-14 | | 1.01E-12 | 1.08E-13 | 3.61E-14 |
| *N*-stearoyl methionine | 1.16E-12 | 4.45E-13 | 1.57E-13 | | 1.13E-12 | 3.31E-13 | 1.1E-13 |
| *N*-oleoyl methionine | 2.41E-13 | 6.38E-14 | 2.25E-14 | | 2.34E-13 | 9.37E-14 | 3.12E-14 |
| *N*-linoleoyl methionine | BDL |  |  | | BDL |  |  |
| *N*-arachidonoyl methionine | PISSR |  |  | | PISSR |  |  |
| *N*-docosahexaenoyl methionine | BDL |  |  | | BDL |  |  |
| ***N*-acyl phenylalanine** |  |  |  | |  |  |  |
| *N*-palmitoyl phenylalanine | 1.42E-12 | 2.34E-13 | 8.28E-14 | | 1.98E-12 | 4.37E-13 | 1.46E-13 |
| *N*-stearoyl phenylalanine | 2.66E-12 | 1.9E-13 | 6.7E-14 | | 2.99E-12 | 3.72E-13 | 1.24E-13 |
| *N*-oleoyl phenylalanine | 1.02E-12 | 1.69E-13 | 5.97E-14 | | 1.02E-12 | 2.24E-13 | 7.47E-14 |
| *N*-linoleoyl phenylalanine | 1.12E-13 | 2.61E-14 | 9.21E-15 | | 1E-13 | 3.11E-14 | 1.04E-14 |
| *N*-arachidonoyl phenylalanine | 5.51E-13 | 1.14E-13 | 4.02E-14 | | 4.3E-13 | 1.14E-13 | 3.78E-14 |
| *N*-docosahexaenoyl phenylalanine | 4.85E-13 | 1.09E-13 | 3.86E-14 | | 4.81E-13 | 1.4E-13 | 4.65E-14 |

**Supplemental Table 25: Continued**

|  | Cortex | | | | | |
| --- | --- | --- | --- | --- | --- | --- |
|  | Vehicle | | | 3mg/kg CP 55,940 | | |
| ***N*-acyl proline** | Mean | Std Dev | Std Error | Mean | Std Dev | Std Error |
| *N*-palmitoyl proline | BDL |  |  | BDL |  |  |
| *N*-stearoyl proline | BDL |  |  | BDL |  |  |
| *N-*oleoyl proline | BDL |  |  | BDL |  |  |
| *N*-linoleoyl proline | BDL |  |  | BDL |  |  |
| *N*-arachidonoyl proline | BDL |  |  | BDL |  |  |
| *N*-docosahexaenoyl proline | BDL |  |  | BDL |  |  |
| ***N*-acyl serine** |  |  |  |  |  |  |
| *N*-palmitoyl serine | 4.06E-11 | 3.31E-12 | 1.17E-12 | 4.17E-11 | 5.34E-12 | 1.78E-12 |
| *N*-stearoyl serine | 3.66E-11 | 4.14E-12 | 1.46E-12 | 3.75E-11 | 4.67E-12 | 1.56E-12 |
| *N*-oleoyl serine | 1.12E-10 | 5.47E-12 | 1.93E-12 | 1.22E-10 | 1.22E-11 | 4.06E-12 |
| *N*-linoleoyl serine | 6.16E-11 | 4.97E-12 | 1.76E-12 | 6.54E-11 | 7.2E-12 | 2.4E-12 |
| *N*-arachidonoyl serine | 1.14E-11 | 1.67E-12 | 5.9E-13 | 1.27E-11 | 1.96E-12 | 6.54E-13 |
| *N*-docosahexaenoyl serine | 1.91E-11 | 2.15E-12 | 7.6E-13 | 1.83E-11 | 1.65E-12 | 5.51E-13 |
| ***N*-acyl taurine** |  |  |  |  |  |  |
| *N*-arachidonoyl taurine | 7.1E-11 | 8.86E-12 | 3.13E-12 | 5.99E-11 | 4.32E-12 | 1.44E-12 |
| ***N*-acyl tryptophan** |  |  |  |  |  |  |
| *N*-palmitoyl tryptophan | BDL |  |  | BDL |  |  |
| *N*-stearoyl tryptophan | BDL |  |  | BDL |  |  |
| *N*-oleoyl tryptophan | BDL |  |  | BDL |  |  |
| *N*-linoleoyl tryptophan | BDL |  |  | BDL |  |  |
| *N*-arachidonoyl tryptophan | BDL |  |  | BDL |  |  |
| *N*-docosahexaenoyl tryptophan | BDL |  |  | BDL |  |  |
| ***N*-acyl tyrosine** |  |  |  |  |  |  |
| *N*-palmitoyl tyrosine | 5.34E-13 | 1.06E-13 | 3.74E-14 | 6.65E-13 | 1.46E-13 | 4.85E-14 |
| *N*-stearoyl tyrosine | 2.44E-13 | 3.84E-14 | 1.36E-14 | 2.52E-13 | 3.87E-14 | 1.29E-14 |
| *N-*oleoyl tyrosine | 3.5E-13 | 7.6E-14 | 2.69E-14 | 3.4E-13 | 1.23E-13 | 4.1E-14 |
| *N*-linoleoyl tyrosine | BDL |  |  | BDL |  |  |
| *N*-arachidonoyl tyrosine | 1.98E-13 | 8.84E-14 | 3.12E-14 | 1.87E-13 | 5.03E-14 | 1.68E-14 |
| *N*-docosahexaenoyl tyrosine | 4.59E-13 | 2.3E-13 | 8.12E-14 | 3.22E-13 | 1.3E-13 | 4.34E-14 |
| ***N*-acyl valine** |  |  |  |  |  |  |
| *N*-palmitoyl valine | 2.28E-13 | 5.98E-14 | 2.11E-14 | 3.13E-13 | 7.15E-14 | 2.38E-14 |
| *N*- stearoyl valine | 1.8E-13 | 3.45E-14 | 1.22E-14 | 2.32E-13 | 6.66E-14 | 2.22E-14 |
| *N*-oleoyl valine | 8.62E-14 | 5.37E-14 | 1.9E-14 | 6.43E-14 | 3.44E-14 | 1.15E-14 |
| *N*-nervonoyl valine | BDL |  |  | BDL |  |  |
| *N*-linoleoyl valine | PISSR |  |  | PISSR |  |  |
| *N*-docosahexaenoyl valine | PISSR |  |  | PISSR |  |  |
| **2-acyl glycerols** |  |  |  |  |  |  |
| 2-palmitoyl glycerol | 5.49E-09 | 1.39E-09 | 4.93E-10 | 5.35E-09 | 2.46E-09 | 8.18E-10 |
| 2-oleoyl glycerol | 1.74E-09 | 1.44E-10 | 5.08E-11 | 1.53E-09 | 1.7E-10 | 5.68E-11 |
| 2-linoleoyl glycerol | 1.42E-10 | 3.98E-11 | 1.41E-11 | 9.55E-11 | 1.38E-11 | 4.61E-12 |
| 2-arachidonoyl glycerol | 2.62E-09 | 9.42E-10 | 3.33E-10 | 2.72E-09 | 6.59E-10 | 2.2E-10 |
| **Free Fatty Acids** |  |  |  |  |  |  |
| Oleic acid | 1.95E-09 | 4.24E-10 | 1.5E-10 | 1.6E-09 | 4.74E-10 | 1.58E-10 |
| Linoleic acid | 1.68E-09 | 3.57E-10 | 1.26E-10 | 9.48E-10 | 1.02E-10 | 3.41E-11 |
| Arachidonic acid | 1.86E-09 | 4.36E-10 | 1.54E-10 | 1.42E-09 | 3.72E-10 | 1.24E-10 |
| **PhosphoLEA** |  |  |  |  |  |  |
| PhosphoLEA | 3.05E-12 | 1.22E-12 | 4.31E-13 | 3.22E-12 | 1.06E-12 | 3.54E-13 |
| **Prostaglandins** |  |  |  |  |  |  |
| PGE_2_ | 1.99E-10 | 2.79E-11 | 9.85E-12 | 1.58E-10 | 1.8E-11 | 5.98E-12 |
| PGF_2α_ | 1.76E-10 | 2.66E-11 | 9.4E-12 | 1.71E-10 | 2.06E-11 | 6.87E-12 |
| 6-ketoPGF_1α_ | 5.58E-12 | 1.7E-12 | 6.02E-13 | 5.34E-12 | 2.35E-12 | 7.85E-13 |
| **THC/CP/CBD** |  |  |  |  |  |  |
| Cannabidiol | BDL |  |  | BDL |  |  |
| CP 55,940 | BDL |  |  | 8.61E-11 | 1.36E-11 | 4.54E-12 |
| THC | BDL |  |  | BDL |  |  |
| **THC Metabolites** |  |  |  |  |  |  |
| 11-nor-9-carboxyTHC | BDL |  |  | BDL |  |  |
| 11-OH-THC | BDL |  |  | BDL |  |  |

**Supplemental Table 26.** List of lipids in the cortex significantly affected by 3 mg/kg CP 55,940: PND 50

| PND 50 Cortex Significant Differences in One-Way ANOVA | | | | |
| --- | --- | --- | --- | --- |
| Lipid | F | p | Direction (relative to Veh) | Magnitude (x Veh level) |
| *N*-arachidonoyl alanine | 8.17 | .012 | ↓ | 0.75 |
| *N*-oleoyl ethanolamine | 13.85 | .002 | ↓ | 0.65 |
| *N*-linoleoyl ethanolamine | 3.92 | .066 | ↓ | 0.69 |
| *N*-arachidonoyl ethanolamine | 9.73 | .007 | ↓ | 0.70 |
| *N*-docosahexaenoyl ethanolamine | 7.64 | .014 | ↓ | 0.72 |
| *N*-arachidonoyl GABA | 11.62 | .004 | ↓ | 0.86 |
| *N*-palmitoyl leucine | 35.91 | .000 | ↑ | 1.41 |
| *N*-palmitoyl methionine | 54.55 | .000 | ↑ | 1.43 |
| *N*-palmitoyl phenylalanine | 10.41 | .006 | ↑ | 1.39 |
| *N*-stearoyl phenylalanine | 5.10 | .039 | ↑ | 1.12 |
| *N*-arachidonoyl phenylalanine | 4.84 | .044 | ↓ | 0.78 |
| *N*-oleoyl serine | 5.05 | .040 | ↑ | 1.09 |
| *N*-arachidonoyl taurine | 11.25 | .004 | ↓ | 0.84 |
| *N*-palmitoyl tyrosine | 4.40 | .053 | ↑ | 1.25 |
| *N*-palmitoyl valine | 6.86 | .019 | ↑ | 1.37 |
| *N*-stearoyl valine | 3.89 | .067 | ↑ | 1.29 |
| 2-oleoyl glycerol | 7.07 | .018 | ↓ | 0.88 |
| 2-linoleoyl glycerol | 10.97 | .005 | ↓ | 0.67 |
| Linoleic acid | 34.60 | .000 | ↓ | 0.57 |
| Arachidonic acid | 5.07 | .040 | ↓ | 0.76 |
| PGE_2_ | 13.61 | .002 | ↓ | 0.79 |
| CP 55,940 | 317.38 | .000 | ↑ | infinite |

**Supplemental Table 27.** Lipid levels in the hypothalamus of PND 50 female mice treated with Vehicle or 3 mg/kg CP 55,940

|  | Hypothalamus | | | | | | |
| --- | --- | --- | --- | --- | --- | --- | --- |
|  | Vehicle | | | 3mg/kg CP 55,940 | | | |
| ***N*-acyl alanine** | Mean | Std Dev | Std Error | | Mean | Std Dev | Std Error |
| *N*-palmitoyl alanine | 7.44E-12 | 2.18E-12 | 7.7E-13 | | 6.9E-12 | 1.91E-12 | 6.36E-13 |
| *N*-stearoyl alanine | 1.14E-12 | 3.17E-13 | 1.12E-13 | | 9.94E-13 | 4E-13 | 1.33E-13 |
| *N*-oleoyl alanine | 3.18E-12 | 1.46E-12 | 5.16E-13 | | 2.61E-12 | 8.58E-13 | 2.86E-13 |
| *N*-linoleoyl alanine | 3.78E-13 | 1.93E-13 | 6.82E-14 | | 3.81E-13 | 1.91E-13 | 6.37E-14 |
| *N*-arachidonoyl alanine | PISSR |  |  | | PISSR |  |  |
| *N*-docosahexaenoyl alanine | PISSR |  |  | | PISSR |  |  |
| ***N*-acyl dopamine** |  |  |  | |  |  |  |
| *N*-oleoyl dopamine | BDL |  |  | | BDL |  |  |
| *N*-arachidonoyl dopamine | BDL |  |  | | BDL |  |  |
| ***N*-acyl ethanolamine** |  |  |  | |  |  |  |
| *N*-palmitoyl ethanolamine | 6.82E-11 | 2.1E-11 | 7.44E-12 | | 6.24E-11 | 1.35E-11 | 4.51E-12 |
| *N*-stearoyl ethanolamine | 4.44E-11 | 1.79E-11 | 6.32E-12 | | 3.67E-11 | 9.62E-12 | 3.21E-12 |
| *N*-oleoyl ethanolamine | 9.21E-11 | 3.53E-11 | 1.25E-11 | | 8.36E-11 | 2.05E-11 | 6.84E-12 |
| *N*-linoleoyl ethanolamine | 3.73E-11 | 1.32E-11 | 4.68E-12 | | 3.47E-11 | 9.68E-12 | 3.23E-12 |
| *N*-arachidonoyl ethanolamine | 2.71E-11 | 1.09E-11 | 3.84E-12 | | 2.54E-11 | 7.42E-12 | 2.47E-12 |
| *N*-docosahexaenoyl ethanolamine | 3.7E-11 | 9.38E-12 | 3.31E-12 | | 3.64E-11 | 7.41E-12 | 2.47E-12 |
| ***N*-acyl GABA** |  |  |  | |  |  |  |
| *N*-palmitoyl GABA | 5.44E-12 | 1.31E-12 | 4.62E-13 | | 6.6E-12 | 1.55E-12 | 5.16E-13 |
| *N*-stearoyl GABA | 6.74E-12 | 1.97E-12 | 6.97E-13 | | 7.69E-12 | 1.18E-12 | 3.93E-13 |
| *N*-oleoyl GABA | 1.76E-12 | 7.28E-13 | 2.58E-13 | | 1.97E-12 | 6.55E-13 | 2.18E-13 |
| *N*-linoleoyl GABA | PISSR |  |  | | PISSR |  |  |
| *N*-arachidonoyl GABA | 7.31E-12 | 2.03E-12 | 7.18E-13 | | 6.6E-12 | 2.34E-12 | 7.8E-13 |
| *N*-docosahexaenoyl GABA | PISSR |  |  | | PISSR |  |  |
| ***N*-acyl glycine** |  |  |  | |  |  |  |
| *N*-palmitoyl glycine | 3.69E-12 | 1.2E-12 | 4.24E-13 | | 3.58E-12 | 8.82E-13 | 2.94E-13 |
| *N*-stearoyl glycine | 4.95E-13 | 2.43E-13 | 8.6E-14 | | 5.58E-13 | 2.71E-13 | 9.02E-14 |
| *N*-oleoyl glycine | 6.17E-13 | 2.98E-13 | 1.05E-13 | | 5.38E-13 | 1.75E-13 | 5.85E-14 |
| *N*-linoleoyl glycine | 1.09E-13 | 4.27E-14 | 1.51E-14 | | 1.12E-13 | 5.7E-14 | 1.9E-14 |
| *N*-arachidonoyl glycine | 4.14E-13 | 2.99E-13 | 1.06E-13 | | 4.44E-13 | 2.73E-13 | 9.1E-14 |
| *N*-docosahexaenoyl glycine | 1.05E-13 | 7.69E-14 | 2.72E-14 | | 1.54E-13 | 5.32E-14 | 1.77E-14 |
| ***N*-acyl leucine** |  |  |  | |  |  |  |
| *N*-palmitoyl leucine | 9.39E-13 | 2.23E-13 | 7.9E-14 | | 1.26E-12 | 2.79E-13 | 9.31E-14 |
| *N*-stearoyl leucine | 8.79E-13 | 2.87E-13 | 1.01E-13 | | 9.96E-13 | 1.52E-13 | 5.08E-14 |
| *N*-oleoyl leucine | 3.6E-13 | 1.31E-13 | 4.63E-14 | | 3.54E-13 | 1.13E-13 | 3.77E-14 |
| *N*-linoleoyl leucine | PISSR |  |  | | PISSR |  |  |
| *N*-docosahexaenoyl leucine | PISSR |  |  | | PISSR |  |  |
| ***N*-acyl methionine** |  |  |  | |  |  |  |
| *N*-palmitoyl methionine | 8.43E-13 | 2.87E-13 | 1.02E-13 | | 9.4E-13 | 3.16E-13 | 1.05E-13 |
| *N*-stearoyl methionine | 4.29E-13 | 1.55E-13 | 5.5E-14 | | 5.57E-13 | 2.88E-13 | 9.6E-14 |
| *N*-oleoyl methionine | PISSR |  |  | | PISSR |  |  |
| *N*-linoleoyl methionine | BDL |  |  | | BDL |  |  |
| *N*-arachidonoyl methionine | BDL |  |  | | BDL |  |  |
| *N*-docosahexaenoyl methionine | BDL |  |  | | BDL |  |  |
| ***N*-acyl phenylalanine** |  |  |  | |  |  |  |
| *N*-palmitoyl phenylalanine | 1.1E-12 | 5.05E-13 | 1.78E-13 | | 1.3E-12 | 2.59E-13 | 8.64E-14 |
| *N*-stearoyl phenylalanine | 1.22E-12 | 2.82E-13 | 9.99E-14 | | 1.28E-12 | 3.09E-13 | 1.03E-13 |
| *N*-oleoyl phenylalanine | 7.62E-13 | 3.59E-13 | 1.27E-13 | | 6.88E-13 | 1.41E-13 | 4.7E-14 |
| *N*-linoleoyl phenylalanine | PISSR |  |  | | PISSR |  |  |
| *N*-arachidonoyl phenylalanine | 4.82E-13 | 1.23E-13 | 4.34E-14 | | 3.4E-13 | 8.5E-14 | 2.83E-14 |
| *N*-docosahexaenoyl phenylalanine | PISSR |  |  | | PISSR |  |  |

**Supplemental Table 27: Continued**

|  | Hypothalamus | | | | | |
| --- | --- | --- | --- | --- | --- | --- |
|  | Vehicle | | | CP 55,940 | | |
| ***N*-acyl proline** | Mean | Std Dev | Std Error | Mean | Std Dev | Std Error |
| *N*-palmitoyl proline | BDL |  |  | BDL |  |  |
| *N*-stearoyl proline | BDL |  |  | BDL |  |  |
| *N-*oleoyl proline | BDL |  |  | BDL |  |  |
| *N*-linoleoyl proline | BDL |  |  | BDL |  |  |
| *N*-arachidonoyl proline | BDL |  |  | BDL |  |  |
| *N*-docosahexaenoyl proline | BDL |  |  | BDL |  |  |
| ***N*-acyl serine** |  |  |  |  |  |  |
| *N*-palmitoyl serine | 3.32E-11 | 7.6E-12 | 2.69E-12 | 3.09E-11 | 3.6E-12 | 1.2E-12 |
| *N*-stearoyl serine | 5.23E-11 | 1.03E-11 | 3.64E-12 | 5.5E-11 | 7.05E-12 | 2.35E-12 |
| *N*-oleoyl serine | 2.93E-10 | 4.66E-11 | 1.65E-11 | 3.07E-10 | 4.36E-11 | 1.45E-11 |
| *N*-linoleoyl serine | 1.61E-10 | 2.08E-11 | 7.34E-12 | 1.7E-10 | 2.41E-11 | 8.03E-12 |
| *N*-arachidonoyl serine | PISSR |  |  | PISSR |  |  |
| *N*-docosahexaenoyl serine | PISSR |  |  | PISSR |  |  |
| ***N*-acyl taurine** |  |  |  |  |  |  |
| *N*-arachidonoyl taurine | 5.8E-12 | 1.81E-12 | 6.4E-13 | 6.54E-12 | 2.6E-12 | 8.67E-13 |
| ***N*-acyl tryptophan** |  |  |  |  |  |  |
| *N*-palmitoyl tryptophan | BDL |  |  | BDL |  |  |
| *N*-stearoyl tryptophan | BDL |  |  | BDL |  |  |
| *N*-oleoyl tryptophan | BDL |  |  | BDL |  |  |
| *N*-linoleoyl tryptophan | BDL |  |  | BDL |  |  |
| *N*-arachidonoyl tryptophan | BDL |  |  | BDL |  |  |
| *N*-docosahexaenoyl tryptophan | BDL |  |  | BDL |  |  |
| ***N*-acyl tyrosine** |  |  |  |  |  |  |
| *N*-palmitoyl tyrosine | 3.42E-13 | 1.96E-13 | 6.93E-14 | 4.12E-13 | 1.01E-13 | 3.37E-14 |
| *N*-stearoyl tyrosine | 8.96E-14 | 4.79E-14 | 1.69E-14 | 1.01E-13 | 4.67E-14 | 1.56E-14 |
| *N-*oleoyl tyrosine | 1.89E-13 | 1.19E-13 | 4.22E-14 | 1.89E-13 | 9.41E-14 | 3.14E-14 |
| *N*-linoleoyl tyrosine | BDL |  |  | BDL |  |  |
| *N*-arachidonoyl tyrosine | BDL |  |  | BDL |  |  |
| *N*-docosahexaenoyl tyrosine | BDL |  |  | BDL |  |  |
| ***N*-acyl valine** |  |  |  |  |  |  |
| *N*-palmitoyl valine | 2.37E-13 | 8.52E-14 | 3.01E-14 | 2.32E-13 | 1.01E-13 | 3.36E-14 |
| *N*- stearoyl valine | PISSR |  |  | PISSR |  |  |
| *N*-oleoyl valine | PISSR |  |  | PISSR |  |  |
| *N*-nervonoyl valine | BDL |  |  | BDL |  |  |
| *N*-linoleoyl valine | BDL |  |  | BDL |  |  |
| *N*-docosahexaenoyl valine | BDL |  |  | BDL |  |  |
| **2-acyl glycerols** |  |  |  |  |  |  |
| 2-palmitoyl glycerol | 3.54E-09 | 3.02E-09 | 1.14E-09 | 2.63E-09 | 2.41E-09 | 8.03E-10 |
| 2-oleoyl glycerol | 1.42E-09 | 1.23E-09 | 4.37E-10 | 1.24E-09 | 1.05E-09 | 3.51E-10 |
| 2-linoleoyl glycerol | 1.34E-10 | 7.07E-11 | 2.5E-11 | 1E-10 | 3.58E-11 | 1.19E-11 |
| 2-arachidonoyl glycerol | 3.22E-10 | 6.97E-11 | 2.46E-11 | 2.57E-10 | 4.24E-11 | 1.41E-11 |
| **Free Fatty Acids** |  |  |  |  |  |  |
| Oleic acid | 2.36E-10 | 5.24E-11 | 1.85E-11 | 2.82E-10 | 1.55E-10 | 5.15E-11 |
| Linoleic acid | 1.79E-10 | 4.7E-11 | 1.66E-11 | 1.86E-10 | 1.07E-10 | 3.57E-11 |
| Arachidonic acid | 1.96E-10 | 3.97E-11 | 1.4E-11 | 1.92E-10 | 6.33E-11 | 2.11E-11 |
| **PhosphoLEA** |  |  |  |  |  |  |
| PhosphoLEA | 1.24E-11 | 5.55E-12 | 2.1E-12 | 1.12E-11 | 2.88E-12 | 9.61E-13 |
| **Prostaglandins** |  |  |  |  |  |  |
| PGE_2_ | 7.81E-11 | 1.37E-11 | 4.84E-12 | 7.14E-11 | 1.21E-11 | 4.03E-12 |
| PGF_2α_ | 9.03E-11 | 6.18E-12 | 2.19E-12 | 1.35E-10 | 3.73E-11 | 1.24E-11 |
| 6-ketoPGF_1α_ | 1.4E-11 | 6.5E-12 | 2.3E-12 | 1.26E-11 | 6.34E-12 | 2.11E-12 |
| **THC/CP/CBD** |  |  |  |  |  |  |
| Cannabidiol | BDL |  |  | BDL |  |  |
| CP 55,940 | BDL |  |  | 4.38E-11 | 5.48E-12 | 1.83E-12 |
| THC | BDL |  |  | BDL |  |  |
| **THC Metabolites** |  |  |  |  |  |  |
| 11-nor-9-carboxyTHC | BDL |  |  | BDL |  |  |
| 11-OH-THC | BDL |  |  | BDL |  |  |

**Supplemental Table 28.** List of lipids in the hypothalamus significantly affected by 3mg/kg CP 55,940: PND 50

| Hypothalamus Significant Differences in One-Way ANOVA | | | | |
| --- | --- | --- | --- | --- |
| Lipid | F | p | Direction (relative to Veh) | Magnitude (x Veh level) |
| *N*-palmitoyl leucine | 6.52 | .022 | ↑ | 1.34 |
| *N*-arachidonoyl phenylalanine | 7.92 | .013 | ↓ | 0.70 |
| 2-arachidonoyl glycerol | 5.53 | .033 | ↓ | 0.80 |
| PGF_2α_ | 11.04 | .005 | ↑ | 1.49 |
| CP 55,940 | 505.68 | .000 | ↑ | infinite |

**Supplemental Table 29.** Lipid levels in the midbrain of PND 50 WT adolescent female mice treated with Vehicle or 3 mg/kg CP 55,940

|  | Midbrain | | | | | | |
| --- | --- | --- | --- | --- | --- | --- | --- |
|  | Vehicle | | | 3mg/kg CP 55,940 | | | |
| ***N*-acyl alanine** | Mean | Std Dev | Std Error | | Mean | Std Dev | Std Error |
| *N*-palmitoyl alanine | 1.53E-12 | 4.11E-13 | 1.45E-13 | | 1.18E-12 | 3.53E-13 | 1.18E-13 |
| *N*-stearoyl alanine | 3.96E-13 | 1.23E-13 | 4.37E-14 | | 3.21E-13 | 8.77E-14 | 2.92E-14 |
| *N*-oleoyl alanine | 8.01E-13 | 2.35E-13 | 8.3E-14 | | 6.9E-13 | 3.72E-13 | 1.24E-13 |
| *N*-linoleoyl alanine | PISSR |  |  | | PISSR |  |  |
| *N*-arachidonoyl alanine | 1.45E-13 | 7.01E-14 | 2.48E-14 | | 9.08E-14 | 3.56E-14 | 1.19E-14 |
| *N*-docosahexaenoyl alanine | PISSR |  |  | | PISSR |  |  |
| ***N*-acyl dopamine** |  |  |  | |  |  |  |
| *N*-oleoyl dopamine | BDL |  |  | | BDL |  |  |
| *N*-arachidonoyl dopamine | BDL |  |  | | BDL |  |  |
| ***N*-acyl ethanolamine** |  |  |  | |  |  |  |
| *N*-palmitoyl ethanolamine | 8.93E-11 | 8.18E-12 | 2.89E-12 | | 7.69E-11 | 9.33E-12 | 3.11E-12 |
| *N*-stearoyl ethanolamine | 3.62E-11 | 9.79E-12 | 3.46E-12 | | 3.26E-11 | 8.2E-12 | 2.73E-12 |
| *N*-oleoyl ethanolamine | 1.22E-10 | 2.11E-11 | 7.46E-12 | | 1.01E-10 | 1.59E-11 | 5.31E-12 |
| *N*-linoleoyl ethanolamine | 6.4E-11 | 6.52E-12 | 2.31E-12 | | 5.21E-11 | 7.31E-12 | 2.44E-12 |
| *N*-arachidonoyl ethanolamine | 4.6E-11 | 6.81E-12 | 2.41E-12 | | 3.81E-11 | 6.9E-12 | 2.3E-12 |
| *N*-docosahexaenoyl ethanolamine | 6.48E-11 | 9.19E-12 | 3.25E-12 | | 5.23E-11 | 1.08E-11 | 3.61E-12 |
| ***N*-acyl GABA** |  |  |  | |  |  |  |
| *N*-palmitoyl GABA | 1.22E-11 | 2.96E-12 | 1.05E-12 | | 1.3E-11 | 3.66E-12 | 1.22E-12 |
| *N*-stearoyl GABA | 1.27E-11 | 2.29E-12 | 8.11E-13 | | 1.21E-11 | 3.65E-12 | 1.22E-12 |
| *N*-oleoyl GABA | 6.47E-12 | 1.27E-12 | 4.47E-13 | | 5.62E-12 | 1.97E-12 | 6.58E-13 |
| *N*-linoleoyl GABA | 1.24E-12 | 3.47E-13 | 1.23E-13 | | 1.04E-12 | 3.14E-13 | 1.05E-13 |
| *N*-arachidonoyl GABA | 2.12E-11 | 3.93E-12 | 1.39E-12 | | 1.55E-11 | 5.07E-12 | 1.69E-12 |
| *N*-docosahexaenoyl GABA | 2.05E-12 | 3.55E-13 | 1.25E-13 | | 1.45E-12 | 3.48E-13 | 1.16E-13 |
| ***N*-acyl glycine** |  |  |  | |  |  |  |
| *N*-palmitoyl glycine | 1.32E-11 | 4.19E-12 | 1.48E-12 | | 1.39E-11 | 4.11E-12 | 1.37E-12 |
| *N*-stearoyl glycine | 1.5E-12 | 9E-13 | 3.18E-13 | | 1.13E-12 | 9.37E-13 | 3.12E-13 |
| *N*-oleoyl glycine | 3.32E-12 | 1.6E-12 | 5.67E-13 | | 3.06E-12 | 1.49E-12 | 4.97E-13 |
| *N*-linoleoyl glycine | 2.02E-13 | 5E-14 | 1.77E-14 | | 2.23E-13 | 1.03E-13 | 3.43E-14 |
| *N*-arachidonoyl glycine | 6.74E-12 | 8.75E-13 | 3.09E-13 | | 4.39E-12 | 6.95E-13 | 2.32E-13 |
| *N*-docosahexaenoyl glycine | 7.55E-13 | 2.11E-13 | 7.47E-14 | | 5.53E-13 | 2.75E-13 | 9.15E-14 |
| ***N*-acyl leucine** |  |  |  | |  |  |  |
| *N*-palmitoyl leucine | 1.04E-12 | 1.07E-13 | 3.79E-14 | | 1.38E-12 | 2.36E-13 | 7.88E-14 |
| *N*-stearoyl leucine | 8.83E-13 | 2.35E-13 | 8.31E-14 | | 7.66E-13 | 1.34E-13 | 4.47E-14 |
| *N*-oleoyl leucine | 4.94E-13 | 1.17E-13 | 4.12E-14 | | 5.64E-13 | 1.51E-13 | 5.02E-14 |
| *N*-linoleoyl leucine | 4.02E-14 | 1.86E-14 | 6.57E-15 | | 3.86E-14 | 1.54E-14 | 5.13E-15 |
| *N*-docosahexaenoyl leucine | 8.71E-14 | 3.68E-14 | 1.3E-14 | | 1.28E-13 | 4.62E-14 | 1.54E-14 |
| ***N*-acyl methionine** |  |  |  | |  |  |  |
| *N*-palmitoyl methionine | 1.17E-12 | 4.43E-13 | 1.57E-13 | | 1.13E-12 | 5.21E-13 | 1.74E-13 |
| *N*-stearoyl methionine | 1.42E-12 | 3.24E-13 | 1.15E-13 | | 1.11E-12 | 2.53E-13 | 8.42E-14 |
| *N*-oleoyl methionine | 5.87E-13 | 2.87E-13 | 1.01E-13 | | 3.18E-13 | 1.49E-13 | 4.97E-14 |
| *N*-linoleoyl methionine | BDL |  |  | | BDL |  |  |
| *N*-arachidonoyl methionine | 2.8E-13 | 5.01E-14 | 1.77E-14 | | 1.8E-13 | 2.07E-14 | 6.89E-15 |
| *N*-docosahexaenoyl methionine | BDL |  |  | | BDL |  |  |
| ***N*-acyl phenylalanine** |  |  |  | |  |  |  |
| *N*-palmitoyl phenylalanine | 1.75E-12 | 2.51E-13 | 8.88E-14 | | 1.94E-12 | 5.35E-13 | 1.78E-13 |
| *N*-stearoyl phenylalanine | 1.91E-12 | 2.38E-13 | 8.41E-14 | | 1.62E-12 | 3.58E-13 | 1.19E-13 |
| *N*-oleoyl phenylalanine | 1.28E-12 | 1.64E-13 | 5.8E-14 | | 1.15E-12 | 2.33E-13 | 7.76E-14 |
| *N*-linoleoyl phenylalanine | 1.31E-13 | 6.53E-14 | 2.31E-14 | | 1.31E-13 | 8.75E-14 | 2.92E-14 |
| *N*-arachidonoyl phenylalanine | 7.91E-13 | 6.95E-14 | 2.46E-14 | | 5.45E-13 | 4.24E-14 | 1.41E-14 |
| *N*-docosahexaenoyl phenylalanine | 6.4E-13 | 1.72E-13 | 6.09E-14 | | 6.24E-13 | 2.81E-13 | 9.35E-14 |

**Supplemental Table 29: Continued**

|  | Midbrain | | | | | |
| --- | --- | --- | --- | --- | --- | --- |
|  | Vehicle | | | 3mg/kg CP 55,940 | | |
| ***N*-acyl proline** | Mean | Std Dev | Std Error | Mean | Std Dev | Std Error |
| *N*-palmitoyl proline | BDL |  |  | BDL |  |  |
| *N*-stearoyl proline | BDL |  |  | BDL |  |  |
| *N-*oleoyl proline | BDL |  |  | BDL |  |  |
| *N*-linoleoyl proline | BDL |  |  | BDL |  |  |
| *N*-arachidonoyl proline | BDL |  |  | BDL |  |  |
| *N*-docosahexaenoyl proline | BDL |  |  | BDL |  |  |
| ***N*-acyl serine** |  |  |  |  |  |  |
| *N*-palmitoyl serine | 2.06E-11 | 3.28E-12 | 1.16E-12 | 1.74E-11 | 2.84E-12 | 9.45E-13 |
| *N*-stearoyl serine | 4.18E-11 | 5.13E-12 | 1.81E-12 | 3.85E-11 | 6.64E-12 | 2.21E-12 |
| *N*-oleoyl serine | 1.36E-10 | 1.91E-11 | 6.75E-12 | 1.24E-10 | 1.8E-11 | 6.01E-12 |
| *N*-linoleoyl serine | 7.2E-11 | 1E-11 | 3.55E-12 | 6.73E-11 | 1.09E-11 | 3.64E-12 |
| *N*-arachidonoyl serine | 9.66E-12 | 2.82E-12 | 9.98E-13 | 7.6E-12 | 2.08E-12 | 6.95E-13 |
| *N*-docosahexaenoyl serine | 2.16E-11 | 4.34E-12 | 1.53E-12 | 1.85E-11 | 3.47E-12 | 1.16E-12 |
| ***N*-acyl taurine** |  |  |  |  |  |  |
| *N*-arachidonoyl taurine | 1.47E-11 | 2.31E-12 | 8.16E-13 | 1.38E-11 | 2.25E-12 | 7.5E-13 |
| ***N*-acyl tryptophan** |  |  |  |  |  |  |
| *N*-palmitoyl tryptophan | BDL |  |  | BDL |  |  |
| *N*-stearoyl tryptophan | BDL |  |  | BDL |  |  |
| *N*-oleoyl tryptophan | BDL |  |  | BDL |  |  |
| *N*-linoleoyl tryptophan | BDL |  |  | BDL |  |  |
| *N*-arachidonoyl tryptophan | BDL |  |  | BDL |  |  |
| *N*-docosahexaenoyl tryptophan | BDL |  |  | BDL |  |  |
| ***N*-acyl tyrosine** |  |  |  |  |  |  |
| *N*-palmitoyl tyrosine | 7.55E-13 | 1.63E-13 | 5.77E-14 | 8.39E-13 | 1.87E-13 | 6.23E-14 |
| *N*-stearoyl tyrosine | 1.25E-13 | 3.63E-14 | 1.28E-14 | 1.16E-13 | 5.14E-14 | 1.71E-14 |
| *N-*oleoyl tyrosine | 4.23E-13 | 9.9E-14 | 3.5E-14 | 3.77E-13 | 1.33E-13 | 4.44E-14 |
| *N*-linoleoyl tyrosine | BDL |  |  | BDL |  |  |
| *N*-arachidonoyl tyrosine | 5.74E-13 | 9.04E-14 | 3.2E-14 | 3.91E-13 | 8.28E-14 | 2.76E-14 |
| *N*-docosahexaenoyl tyrosine | 8.3E-13 | 3.26E-13 | 1.15E-13 | 5.71E-13 | 3.99E-13 | 1.33E-13 |
| ***N*-acyl valine** |  |  |  |  |  |  |
| *N*-palmitoyl valine | 3.03E-13 | 1.03E-13 | 3.63E-14 | 3.48E-13 | 1.31E-13 | 4.38E-14 |
| *N*- stearoyl valine | 8.24E-14 | 4.18E-14 | 1.48E-14 | 9.43E-14 | 5.43E-14 | 1.81E-14 |
| *N*-oleoyl valine | 5.1E-14 | 3.18E-14 | 1.12E-14 | 6.01E-14 | 4.59E-14 | 1.53E-14 |
| *N*-nervonoyl valine | BDL |  |  | BDL |  |  |
| *N*-linoleoyl valine | BDL |  |  | BDL |  |  |
| *N*-docosahexaenoyl valine | PISSR |  |  | PISSR |  |  |
| **2-acyl glycerols** |  |  |  |  |  |  |
| 2-palmitoyl glycerol | 3.4E-09 | 7.01E-10 | 2.48E-10 | 3.34E-09 | 1.04E-09 | 3.47E-10 |
| 2-oleoyl glycerol | 5.57E-09 | 6.23E-10 | 2.2E-10 | 4.95E-09 | 6.96E-10 | 2.32E-10 |
| 2-linoleoyl glycerol | 4.53E-10 | 6.95E-11 | 2.46E-11 | 3.73E-10 | 5.27E-11 | 1.76E-11 |
| 2-arachidonoyl glycerol | 3.61E-10 | 6.51E-11 | 2.3E-11 | 3.27E-10 | 4.5E-11 | 1.5E-11 |
| **Free Fatty Acids** |  |  |  |  |  |  |
| Oleic acid | 3.46E-10 | 5.91E-11 | 2.09E-11 | 3.11E-10 | 6.86E-11 | 2.29E-11 |
| Linoleic acid | 3.56E-10 | 5.39E-11 | 1.91E-11 | 2.67E-10 | 5.25E-11 | 1.75E-11 |
| Arachidonic acid | 4.16E-10 | 4.62E-11 | 1.63E-11 | 3.1E-10 | 5.12E-11 | 1.71E-11 |
| **PhosphoLEA** |  |  |  |  |  |  |
| PhosphoLEA | PISSR |  |  | PISSR |  |  |
| **Prostaglandins** |  |  |  |  |  |  |
| PGE_2_ | 1.45E-10 | 1.1E-11 | 3.88E-12 | 1.09E-10 | 2.35E-11 | 7.84E-12 |
| PGF_2α_ | 1.92E-10 | 2.13E-11 | 7.52E-12 | 1.57E-10 | 2.5E-11 | 8.33E-12 |
| 6-ketoPGF_1α_ | 1.62E-11 | 5.38E-12 | 1.9E-12 | 1.22E-11 | 3.78E-12 | 1.26E-12 |
| **THC/CP/CBD** |  |  |  |  |  |  |
| Cannabidiol | BDL |  |  | BDL |  |  |
| CP 55,940 | BDL |  |  | 1.21E-10 | 1.57E-11 | 5.24E-12 |
| THC | BDL |  |  | BDL |  |  |
| **THC Metabolites** |  |  |  |  |  |  |
| 11-nor-9-carboxyTHC | BDL |  |  | BDL |  |  |
| 11-OH-THC | BDL |  |  | BDL |  |  |

**Supplemental Table 30.** List of lipids in the midbrain significantly affected by 3mg/kg CP 55,940: PND 50

| PND 50 Midbrain Significant Differences in One-Way ANOVA | | | | |
| --- | --- | --- | --- | --- |
| Lipid | F | p | Direction (relative to Veh) | Magnitude (x Veh level) |
| *N*-palmitoyl alanine | 3.51 | .081 | ↓ | 0.77 |
| *N*-arachidonoyl alanine | 4.25 | .057 | ↓ | 0.62 |
| *N*-palmitoyl ethanolamine | 8.44 | .011 | ↓ | 0.86 |
| *N*-oleoyl ethanolamine | 5.24 | .037 | ↓ | 0.83 |
| *N*-linoleoyl ethanolamine | 12.53 | .003 | ↓ | 0.81 |
| *N*-arachidonoyl ethanolamine | 5.67 | .031 | ↓ | 0.83 |
| *N*-docosahexaenoyl ethanolamine | 6.47 | .023 | ↓ | 0.81 |
| *N*-arachidonoyl GABA | 6.59 | .021 | ↓ | 0.73 |
| *N*-docosahexaenoyl GABA | 12.35 | .003 | ↓ | 0.71 |
| *N*-arachidonoyl glycine | 38.07 | .000 | ↓ | 0.65 |
| *N*-palmitoyl leucine | 14.04 | .002 | ↑ | 1.33 |
| *N*-docosahexaenoyl leucine | 4.03 | .063 | ↑ | 1.47 |
| *N*-stearoyl methionine | 4.94 | .042 | ↓ | 0.78 |
| *N*-oleoyl methionine | 6.10 | .026 | ↓ | 0.54 |
| *N*-arachidonoyl methionine | 30.38 | .000 | ↓ | 0.64 |
| *N*-stearoyl phenylalanine | 3.60 | .077 | ↓ | 0.85 |
| *N*-arachidonoyl phenylalanine | 80.20 | .000 | ↓ | 0.69 |
| *N*-palmitoyl serine | 4.64 | .048 | ↓ | 0.84 |
| *N*-arachidonoyl tyrosine | 19.07 | .001 | ↓ | 0.68 |
| 2-oleoyl glycerol | 3.72 | .073 | ↓ | 0.89 |
| 2-linoleoyl glycerol | 7.22 | .017 | ↓ | 0.82 |
| Linoleic acid | 11.81 | .004 | ↓ | 0.75 |
| Arachidonic acid | 19.99 | .000 | ↓ | 0.74 |
| PGE_2_ | 15.21 | .001 | ↓ | 0.75 |
| PGF_2α_ | 9.83 | .007 | ↓ | 0.82 |
| 6-ketoPGF_1α_ | 3.30 | .089 | ↓ | 0.75 |
| CP 55,940 | 469.08 | .000 | ↑ | infinite |

**Supplemental Table 31.** Lipid levels in the brainstem of PND50 WT female mice treated with Vehicle or 3 mg/kg CP 55,940

|  | Brainstem | | | | | | |
| --- | --- | --- | --- | --- | --- | --- | --- |
|  | Vehicle | | | 3mg/kg CP 55,940 | | | |
| ***N*-acyl alanine** | Mean | Std Dev | Std Error | | Mean | Std Dev | Std Error |
| *N*-palmitoyl alanine | 2.96E-12 | 8.45E-13 | 2.99E-13 | | 3.07E-12 | 1.31E-12 | 4.36E-13 |
| *N*-stearoyl alanine | 1.71E-12 | 6.89E-13 | 2.44E-13 | | 1.5E-12 | 8.17E-13 | 2.72E-13 |
| *N*-oleoyl alanine | 1.45E-12 | 2.59E-13 | 9.16E-14 | | 1.3E-12 | 4.91E-13 | 1.64E-13 |
| *N*-linoleoyl alanine | 9.37E-14 | 4.3E-14 | 1.52E-14 | | 1.01E-13 | 3.26E-14 | 1.09E-14 |
| *N*-arachidonoyl alanine | 1.53E-13 | 5.52E-14 | 1.95E-14 | | 1.22E-13 | 3.36E-14 | 1.12E-14 |
| *N*-docosahexaenoyl alanine | PISSR |  |  | | PISSR |  |  |
| ***N*-acyl dopamine** |  |  |  | |  |  |  |
| *N*-oleoyl dopamine | BDL |  |  | | BDL |  |  |
| *N*-arachidonoyl dopamine | BDL |  |  | | BDL |  |  |
| ***N*-acyl ethanolamine** |  |  |  | |  |  |  |
| *N*-palmitoyl ethanolamine | 6.51E-11 | 1.15E-11 | 4.07E-12 | | 6.28E-11 | 1.01E-11 | 3.37E-12 |
| *N*-stearoyl ethanolamine | 2.65E-11 | 6.39E-12 | 2.26E-12 | | 2.63E-11 | 3.71E-12 | 1.24E-12 |
| *N*-oleoyl ethanolamine | 7.9E-11 | 1.22E-11 | 4.31E-12 | | 7.29E-11 | 1.07E-11 | 3.57E-12 |
| *N*-linoleoyl ethanolamine | 3.68E-11 | 6.88E-12 | 2.43E-12 | | 3.38E-11 | 6.92E-12 | 2.31E-12 |
| *N*-arachidonoyl ethanolamine | 2.67E-11 | 4.1E-12 | 1.45E-12 | | 2.44E-11 | 5.37E-12 | 1.79E-12 |
| *N*-docosahexaenoyl ethanolamine | 4.1E-11 | 8.16E-12 | 2.89E-12 | | 3.73E-11 | 9.2E-12 | 3.07E-12 |
| ***N*-acyl GABA** |  |  |  | |  |  |  |
| *N*-palmitoyl GABA | 5.92E-12 | 1.4E-12 | 4.94E-13 | | 6.41E-12 | 2.63E-12 | 8.77E-13 |
| *N*-stearoyl GABA | 7.6E-12 | 2.07E-12 | 7.32E-13 | | 7.1E-12 | 2.36E-12 | 7.85E-13 |
| *N*-oleoyl GABA | 4.31E-12 | 1.14E-12 | 4.03E-13 | | 3.95E-12 | 1.59E-12 | 5.29E-13 |
| *N*-linoleoyl GABA | 6.98E-13 | 3.24E-13 | 1.14E-13 | | 6.01E-13 | 2.64E-13 | 8.8E-14 |
| *N*-arachidonoyl GABA | 5.39E-12 | 4.62E-13 | 1.63E-13 | | 3.67E-12 | 5.37E-13 | 1.79E-13 |
| *N*-docosahexaenoyl GABA | 1.05E-12 | 3.37E-13 | 1.19E-13 | | 9.05E-13 | 4.41E-13 | 1.47E-13 |
| ***N*-acyl glycine** |  |  |  | |  |  |  |
| *N*-palmitoyl glycine | 1.42E-11 | 4.02E-12 | 1.42E-12 | | 1.47E-11 | 6.18E-12 | 2.06E-12 |
| *N*-stearoyl glycine | 4.61E-12 | 2.79E-12 | 9.85E-13 | | 4.43E-12 | 3.68E-12 | 1.23E-12 |
| *N*-oleoyl glycine | 6.15E-12 | 2.53E-12 | 8.95E-13 | | 5.38E-12 | 3.14E-12 | 1.05E-12 |
| *N*-linoleoyl glycine | 4.29E-13 | 1.48E-13 | 5.24E-14 | | 4.12E-13 | 2.29E-13 | 7.63E-14 |
| *N*-arachidonoyl glycine | 1.02E-11 | 3.09E-12 | 1.09E-12 | | 9.26E-12 | 6.53E-12 | 2.18E-12 |
| *N*-docosahexaenoyl glycine | 1.72E-12 | 6.07E-13 | 2.15E-13 | | 1.5E-12 | 7.61E-13 | 2.54E-13 |
| ***N*-acyl leucine** |  |  |  | |  |  |  |
| *N*-palmitoyl leucine | 7.69E-13 | 8.58E-14 | 3.03E-14 | | 1.11E-12 | 2.05E-13 | 6.82E-14 |
| *N*-stearoyl leucine | 7.36E-13 | 1.48E-13 | 5.24E-14 | | 7.24E-13 | 1.83E-13 | 6.09E-14 |
| *N*-oleoyl leucine | 4.39E-13 | 7.04E-14 | 2.49E-14 | | 4.93E-13 | 1.12E-13 | 3.73E-14 |
| *N*-linoleoyl leucine | 4.21E-14 | 2.14E-14 | 7.57E-15 | | 4.41E-14 | 2.43E-14 | 8.11E-15 |
| *N*-docosahexaenoyl leucine | 1.02E-13 | 3.6E-14 | 1.27E-14 | | 1.24E-13 | 2.92E-14 | 9.72E-15 |
| ***N*-acyl methionine** |  |  |  | |  |  |  |
| *N*-palmitoyl methionine | 8.5E-13 | 2.47E-13 | 8.74E-14 | | 7.37E-13 | 1.65E-13 | 5.49E-14 |
| *N*-stearoyl methionine | 7.37E-13 | 2.23E-13 | 7.9E-14 | | 8.45E-13 | 2.44E-13 | 8.14E-14 |
| *N*-oleoyl methionine | 2.69E-13 | 9.59E-14 | 3.39E-14 | | 1.84E-13 | 6.01E-14 | 2E-14 |
| *N*-linoleoyl methionine | PISSR |  |  | | PISSR |  |  |
| *N*-arachidonoyl methionine | PISSR |  |  | | PISSR |  |  |
| *N*-docosahexaenoyl methionine | PISSR |  |  | | PISSR |  |  |
| ***N*-acyl phenylalanine** |  |  |  | |  |  |  |
| *N*-palmitoyl phenylalanine | 9E-13 | 1.11E-13 | 3.91E-14 | | 1.09E-12 | 3.34E-13 | 1.11E-13 |
| *N*-stearoyl phenylalanine | 1.02E-12 | 1.62E-13 | 5.74E-14 | | 9.56E-13 | 2.2E-13 | 7.32E-14 |
| *N*-oleoyl phenylalanine | 7.56E-13 | 1.19E-13 | 4.21E-14 | | 7.71E-13 | 2.45E-13 | 8.16E-14 |
| *N*-linoleoyl phenylalanine | PISSR |  |  | | PISSR |  |  |
| *N*-arachidonoyl phenylalanine | 2.27E-13 | 7.25E-14 | 2.56E-14 | | 3.18E-13 | 7.61E-14 | 2.54E-14 |
| *N*-docosahexaenoyl phenylalanine | 2.2E-13 | 6.74E-14 | 2.38E-14 | | 1.89E-13 | 1.11E-13 | 3.71E-14 |

**Supplemental Table 31: Continued**

|  | Brainstem | | | | | |
| --- | --- | --- | --- | --- | --- | --- |
|  | Vehicle | | | 3mg/kg CP 55,940 | | |
| ***N*-acyl proline** | Mean | Std Dev | Std Error | Mean | Std Dev | Std Error |
| *N*-palmitoyl proline | BDL |  |  | BDL |  |  |
| *N*-stearoyl proline | BDL |  |  | BDL |  |  |
| *N-*oleoyl proline | BDL |  |  | BDL |  |  |
| *N*-linoleoyl proline | BDL |  |  | BDL |  |  |
| *N*-arachidonoyl proline | BDL |  |  | BDL |  |  |
| *N*-docosahexaenoyl proline | BDL |  |  | BDL |  |  |
| ***N*-acyl serine** |  |  |  |  |  |  |
| *N*-palmitoyl serine | 2.17E-11 | 4.3E-12 | 1.52E-12 | 2E-11 | 3.12E-12 | 1.04E-12 |
| *N*-stearoyl serine | 2.95E-11 | 2.93E-12 | 1.04E-12 | 3.14E-11 | 5.44E-12 | 1.81E-12 |
| *N*-oleoyl serine | 1.03E-10 | 9.33E-12 | 3.3E-12 | 1.1E-10 | 1.49E-11 | 4.98E-12 |
| *N*-linoleoyl serine | 5.93E-11 | 7.27E-12 | 2.57E-12 | 6.34E-11 | 8.27E-12 | 2.76E-12 |
| *N*-arachidonoyl serine | 6.65E-12 | 8.02E-13 | 2.83E-13 | 5.36E-12 | 9.28E-13 | 3.09E-13 |
| *N*-docosahexaenoyl serine | 1.16E-11 | 3.47E-12 | 1.23E-12 | 1.33E-11 | 3.05E-12 | 1.02E-12 |
| ***N*-acyl taurine** |  |  |  |  |  |  |
| *N*-arachidonoyl taurine | 1.38E-11 | 1.28E-12 | 4.53E-13 | 1.14E-11 | 1.44E-12 | 4.79E-13 |
| ***N*-acyl tryptophan** |  |  |  |  |  |  |
| *N*-palmitoyl tryptophan | BDL |  |  | BDL |  |  |
| *N*-stearoyl tryptophan | BDL |  |  | BDL |  |  |
| *N*-oleoyl tryptophan | BDL |  |  | BDL |  |  |
| *N*-linoleoyl tryptophan | BDL |  |  | BDL |  |  |
| *N*-arachidonoyl tryptophan | BDL |  |  | BDL |  |  |
| *N*-docosahexaenoyl tryptophan | BDL |  |  | BDL |  |  |
| ***N*-acyl tyrosine** |  |  |  |  |  |  |
| *N*-palmitoyl tyrosine | 4.7E-13 | 4.45E-14 | 1.57E-14 | 5.77E-13 | 8.52E-14 | 2.84E-14 |
| *N*-stearoyl tyrosine | 8.33E-14 | 4.15E-14 | 1.47E-14 | 7.69E-14 | 2.32E-14 | 7.75E-15 |
| *N-*oleoyl tyrosine | 2.92E-13 | 1.26E-13 | 4.47E-14 | 2.91E-13 | 8.45E-14 | 2.82E-14 |
| *N*-linoleoyl tyrosine | PISSR |  |  | PISSR |  |  |
| *N*-arachidonoyl tyrosine | 3.25E-13 | 5.95E-14 | 2.1E-14 | 2.42E-13 | 4.98E-14 | 1.66E-14 |
| *N*-docosahexaenoyl tyrosine | 5.81E-13 | 1.66E-13 | 5.87E-14 | 4.65E-13 | 1.35E-13 | 4.51E-14 |
| ***N*-acyl valine** |  |  |  |  |  |  |
| *N*-palmitoyl valine | 1.73E-13 | 8.87E-14 | 3.14E-14 | 1.73E-13 | 4.55E-14 | 1.52E-14 |
| *N*- stearoyl valine | 8.56E-14 | 4.31E-14 | 1.52E-14 | 8.37E-14 | 3.7E-14 | 1.23E-14 |
| *N*-oleoyl valine | 3.53E-14 | 1.6E-14 | 5.66E-15 | 6.02E-14 | 4.7E-14 | 1.57E-14 |
| *N*-nervonoyl valine | BDL |  |  | BDL |  |  |
| *N*-linoleoyl valine | BDL |  |  | BDL |  |  |
| *N*-docosahexaenoyl valine | BDL |  |  | BDL |  |  |
| **2-acyl glycerols** |  |  |  |  |  |  |
| 2-palmitoyl glycerol | 1.25E-09 | 4.31E-10 | 1.52E-10 | 8.83E-10 | 3.08E-10 | 1.03E-10 |
| 2-oleoyl glycerol | 4.46E-09 | 4.99E-10 | 1.76E-10 | 3.85E-09 | 6.44E-10 | 2.15E-10 |
| 2-linoleoyl glycerol | 3.24E-10 | 7.5E-11 | 2.65E-11 | 2.39E-10 | 5.28E-11 | 1.76E-11 |
| 2-arachidonoyl glycerol | 2.43E-10 | 4.25E-11 | 1.5E-11 | 2.23E-10 | 3.51E-11 | 1.17E-11 |
| **Free Fatty Acids** |  |  |  |  |  |  |
| Oleic acid | 2.31E-10 | 2.49E-11 | 8.79E-12 | 1.94E-10 | 3.08E-11 | 1.03E-11 |
| Linoleic acid | 1.73E-10 | 3.25E-11 | 1.15E-11 | 1.4E-10 | 2.94E-11 | 9.81E-12 |
| Arachidonic acid | 1.59E-10 | 9.19E-12 | 3.25E-12 | 1.44E-10 | 2.98E-11 | 9.95E-12 |
| **PhosphoLEA** |  |  |  |  |  |  |
| PhosphoLEA | 9.33E-13 | 3.1E-13 | 1.1E-13 | 7.34E-13 | 3.17E-13 | 1.06E-13 |
| **Prostaglandins** |  |  |  |  |  |  |
| PGE_2_ | 1.52E-10 | 2.36E-11 | 8.34E-12 | 1.11E-10 | 3.55E-11 | 1.18E-11 |
| PGF_2α_ | 1.71E-10 | 2.17E-11 | 7.66E-12 | 1.26E-10 | 1.75E-11 | 5.82E-12 |
| 6-ketoPGF_1α_ | 1.94E-11 | 3.32E-12 | 1.18E-12 | 1.35E-11 | 3.26E-12 | 1.09E-12 |
| **THC/CP/CBD** |  |  |  |  |  |  |
| Cannabidiol | BDL |  |  | BDL |  |  |
| CP 55,940 | BDL |  |  | 8.2E-11 | 1.85E-11 | 6.17E-12 |
| THC | BDL |  |  | BDL |  |  |
| **THC Metabolites** |  |  |  |  |  |  |
| 11-nor-9-carboxyTHC | BDL |  |  | BDL |  |  |
| 11-OH-THC | BDL |  |  | BDL |  |  |

**Supplemental Table 32.** List of lipids in the brainstem significantly affected by 3 mg/kg CP 55,940: PND 50

| PND 50 Brainstem Significant Differences in One-Way ANOVA | | | | |
| --- | --- | --- | --- | --- |
| Lipid | F | p | Direction (relative to Veh) | Magnitude (x Veh level) |
| *N*-arachidonoyl GABA | 49.31 | .000 | ↓ | 0.68 |
| *N*-palmitoyl leucine | 18.95 | .001 | ↑ | 1.44 |
| *N*-oleoyl methionine | 5.02 | .041 | ↓ | 0.68 |
| *N*-arachidonoyl phenylalanine | 6.37 | .023 | ↑ | 1.40 |
| *N*-arachidonoyl serine | 9.39 | .008 | ↓ | 0.80 |
| *N*-arachidonoyl taurine | 12.61 | .003 | ↓ | 0.83 |
| *N*-palmitoyl tyrosine | 10.00 | .006 | ↑ | 1.23 |
| *N*-arachidonoyl tyrosine | 9.92 | .007 | ↓ | 0.74 |
| 2-palmitoyl glycerol | 4.05 | .062 | ↓ | 0.71 |
| 2-oleoyl glycerol | 4.73 | .046 | ↓ | 0.86 |
| 2-linoleoyl glycerol | 7.50 | .015 | ↓ | 0.74 |
| Oleic acid | 7.07 | .018 | ↓ | 0.84 |
| Linoleic acid | 4.86 | .043 | ↓ | 0.81 |
| PGE_2_ | 7.80 | .014 | ↓ | 0.73 |
| PGF_2α_ | 22.88 | .000 | ↓ | 0.74 |
| 6-ketoPGF_1α_ | 13.55 | .002 | ↓ | 0.70 |
| CP 55,940 | 371.76 | .000 | ↑ | infinite |

**Supplemental Table 33.** Lipid levels in the striatum of WT adult female mice treated with Vehicle or 3 mg/kg CP 55,940

|  | Striatum | | | | | | |
| --- | --- | --- | --- | --- | --- | --- | --- |
|  | Vehicle | | | 3mg/kg CP 55,940 | | | |
| ***N*-acyl alanine** | Mean | Std Dev | Std Error | | Mean | Std Dev | Std Error |
| *N*-palmitoyl alanine | 3.78E-12 | 1.41E-12 | 4.97E-13 | | 3.83E-12 | 1.19E-12 | 4.22E-13 |
| *N*-stearoyl alanine | 4.83E-12 | 8.23E-13 | 2.91E-13 | | 4.15E-12 | 9.28E-13 | 3.28E-13 |
| *N*-oleoyl alanine | 4.03E-12 | 2.71E-13 | 9.59E-14 | | 3.96E-12 | 9.49E-13 | 3.36E-13 |
| *N*-linoleoyl alanine | PISSR |  |  | | PISSR |  |  |
| *N*-arachidonoyl alanine | 2.11E-12 | 6.17E-13 | 2.18E-13 | | 2.03E-12 | 6.67E-13 | 2.36E-13 |
| *N*-docosahexaenoyl alanine | BDL |  |  | | BDL |  |  |
| ***N*-acyl dopamine** |  |  |  | |  |  |  |
| *N*-oleoyl dopamine | BDL |  |  | | BDL |  |  |
| *N*-arachidonoyl dopamine | BDL |  |  | | BDL |  |  |
| ***N*-acyl ethanolamine** |  |  |  | |  |  |  |
| *N*-palmitoyl ethanolamine | 1.01E-10 | 3.96E-11 | 1.4E-11 | | 7.87E-11 | 2.11E-11 | 7.45E-12 |
| *N*-stearoyl ethanolamine | 5.1E-11 | 5.09E-11 | 1.8E-11 | | 3.49E-11 | 3.65E-11 | 1.29E-11 |
| *N*-oleoyl ethanolamine | 2.43E-10 | 8.38E-11 | 2.96E-11 | | 1.57E-10 | 7.03E-11 | 2.49E-11 |
| *N*-linoleoyl ethanolamine | 5.64E-11 | 1.72E-11 | 6.09E-12 | | 3.83E-11 | 6.77E-12 | 2.39E-12 |
| *N*-arachidonoyl ethanolamine | 3.31E-11 | 1.07E-11 | 3.79E-12 | | 2.19E-11 | 4.7E-12 | 1.66E-12 |
| *N*-docosahexaenoyl ethanolamine | 3.16E-11 | 6.29E-12 | 2.22E-12 | | 2.36E-11 | 3.39E-12 | 1.2E-12 |
| ***N*-acyl GABA** |  |  |  | |  |  |  |
| *N*-palmitoyl GABA | 2.76E-11 | 4.55E-12 | 1.61E-12 | | 2.94E-11 | 4.25E-12 | 1.5E-12 |
| *N*-stearoyl GABA | 2.12E-11 | 2.03E-12 | 7.17E-13 | | 2.26E-11 | 1.69E-12 | 5.97E-13 |
| *N*-oleoyl GABA | 1.92E-11 | 2.37E-12 | 8.38E-13 | | 1.7E-11 | 3.24E-12 | 1.15E-12 |
| *N*-linoleoyl GABA | PISSR |  |  | | PISSR |  |  |
| *N*-arachidonoyl GABA | 2.48E-11 | 3.05E-12 | 1.08E-12 | | 2.43E-11 | 3.83E-12 | 1.36E-12 |
| *N*-docosahexaenoyl GABA | PISSR |  |  | | PISSR |  |  |
| ***N*-acyl glycine** |  |  |  | |  |  |  |
| *N*-palmitoyl glycine | 2.64E-12 | 7.48E-13 | 2.65E-13 | | 2.5E-12 | 4.9E-13 | 1.73E-13 |
| *N*-stearoyl glycine | 3.05E-12 | 1.13E-12 | 4.01E-13 | | 3.37E-12 | 1.27E-12 | 4.49E-13 |
| *N*-oleoyl glycine | 6.09E-13 | 2.21E-13 | 7.81E-14 | | 5.26E-13 | 1.57E-13 | 5.54E-14 |
| *N*-linoleoyl glycine | PISSR |  |  | | PISSR |  |  |
| *N*-arachidonoyl glycine | 6.91E-13 | 2.2E-13 | 7.79E-14 | | 6.38E-13 | 2.54E-13 | 9E-14 |
| *N*-docosahexaenoyl glycine | PISSR |  |  | | PISSR |  |  |
| ***N*-acyl leucine** |  |  |  | |  |  |  |
| *N*-palmitoyl leucine | 2.99E-12 | 3.87E-13 | 1.37E-13 | | 3E-12 | 4.85E-13 | 1.71E-13 |
| *N*-stearoyl leucine | 3.61E-12 | 5.64E-13 | 1.99E-13 | | 3.52E-12 | 8.6E-13 | 3.04E-13 |
| *N*-oleoyl leucine | 1.66E-12 | 3.59E-13 | 1.27E-13 | | 1.99E-12 | 2.22E-13 | 7.84E-14 |
| *N*-linoleoyl leucine | PISSR |  |  | | PISSR |  |  |
| *N*-docosahexaenoyl leucine | PISSR |  |  | | PISSR |  |  |
| ***N*-acyl methionine** |  |  |  | |  |  |  |
| *N*-palmitoyl methionine | PISSR |  |  | | PISSR |  |  |
| *N*-stearoyl methionine | PISSR |  |  | | PISSR |  |  |
| *N*-oleoyl methionine | PISSR |  |  | | PISSR |  |  |
| *N*-linoleoyl methionine | BDL |  |  | | BDL |  |  |
| *N*-arachidonoyl methionine | BDL |  |  | | BDL |  |  |
| *N*-docosahexaenoyl methionine | BDL |  |  | | BDL |  |  |
| ***N*-acyl phenylalanine** |  |  |  | |  |  |  |
| *N*-palmitoyl phenylalanine | 4.54E-13 | 2.5E-13 | 8.84E-14 | | 3.31E-13 | 6.71E-14 | 2.37E-14 |
| *N*-stearoyl phenylalanine | 5.96E-13 | 2.43E-13 | 8.58E-14 | | 4.25E-13 | 1.33E-13 | 4.71E-14 |
| *N*-oleoyl phenylalanine | 5.15E-13 | 1.98E-13 | 7.01E-14 | | 4.84E-13 | 1.89E-13 | 6.7E-14 |
| *N*-linoleoyl phenylalanine | PISSR |  |  | | PISSR |  |  |
| *N*-arachidonoyl phenylalanine | 1.19E-13 | 6.66E-14 | 2.36E-14 | | 9.64E-14 | 4.63E-14 | 1.64E-14 |
| *N*-docosahexaenoyl phenylalanine | PISSR |  |  | | PISSR |  |  |

**Supplemental Table 33: Continued**

|  | Striatum | | | | | |
| --- | --- | --- | --- | --- | --- | --- |
|  | Vehicle | | | 3mg/kg CP 55,940 | | |
| ***N*-acyl proline** | Mean | Std Dev | Std Error | Mean | Std Dev | Std Error |
| *N*-palmitoyl proline | PISSR |  |  | PISSR |  |  |
| *N*-stearoyl proline | BDL |  |  | BDL |  |  |
| *N-*oleoyl proline | BDL |  |  | BDL |  |  |
| *N*-linoleoyl proline | BDL |  |  | BDL |  |  |
| *N*-arachidonoyl proline | BDL |  |  | BDL |  |  |
| *N*-docosahexaenoyl proline | BDL |  |  | BDL |  |  |
| ***N*-acyl serine** |  |  |  |  |  |  |
| *N*-palmitoyl serine | 6.3E-11 | 1.37E-11 | 4.83E-12 | 4.99E-11 | 1.36E-11 | 4.8E-12 |
| *N*-stearoyl serine | PISSR |  |  | PISSR |  |  |
| *N*-oleoyl serine | 5.1E-10 | 7.61E-11 | 2.69E-11 | 5.05E-10 | 8.48E-11 | 3E-11 |
| *N*-linoleoyl serine | 1.06E-10 | 1.85E-11 | 6.55E-12 | 1.09E-10 | 1.98E-11 | 7E-12 |
| *N*-arachidonoyl serine | PISSR |  |  | PISSR |  |  |
| *N*-docosahexaenoyl serine | PISSR |  |  | PISSR |  |  |
| ***N*-acyl taurine** |  |  |  |  |  |  |
| *N*-arachidonoyl taurine | 1.09E-10 | 1.57E-11 | 5.54E-12 | 9.74E-11 | 1.22E-11 | 4.3E-12 |
| ***N*-acyl tryptophan** |  |  |  |  |  |  |
| *N*-palmitoyl tryptophan | BDL |  |  | BDL |  |  |
| *N*-stearoyl tryptophan | BDL |  |  | BDL |  |  |
| *N*-oleoyl tryptophan | BDL |  |  | BDL |  |  |
| *N*-linoleoyl tryptophan | BDL |  |  | BDL |  |  |
| *N*-arachidonoyl tryptophan | BDL |  |  | BDL |  |  |
| *N*-docosahexaenoyl tryptophan | BDL |  |  | BDL |  |  |
| ***N*-acyl tyrosine** |  |  |  |  |  |  |
| *N*-palmitoyl tyrosine | 2.58E-12 | 5.57E-13 | 1.97E-13 | 2.47E-12 | 7.44E-13 | 2.63E-13 |
| *N*-stearoyl tyrosine | PISSR |  |  | PISSR |  |  |
| *N-*oleoyl tyrosine | PISSR |  |  | PISSR |  |  |
| *N*-linoleoyl tyrosine | BDL |  |  | BDL |  |  |
| *N*-arachidonoyl tyrosine | PISSR |  |  | PISSR |  |  |
| *N*-docosahexaenoyl tyrosine | PISSR |  |  | PISSR |  |  |
| ***N*-acyl valine** |  |  |  |  |  |  |
| *N*-palmitoyl valine | PISSR |  |  | PISSR |  |  |
| *N*- stearoyl valine | PISSR |  |  | PISSR |  |  |
| *N*-oleoyl valine | PISSR |  |  | PISSR |  |  |
| *N*-nervonoyl valine | BDL |  |  | BDL |  |  |
| *N*-linoleoyl valine | PISSR |  |  | PISSR |  |  |
| *N*-docosahexaenoyl valine | BDL |  |  | BDL |  |  |
| **2-acyl glycerols** |  |  |  |  |  |  |
| 2-palmitoyl-*sn*-glycerol | 1.78E-10 | 1.11E-10 | 3.91E-11 | 1.51E-10 | 6.35E-11 | 2.25E-11 |
| 2-oleoyl-*sn*-glycerol | 1.73E-09 | 8.47E-10 | 3E-10 | 1.58E-09 | 5.54E-10 | 1.96E-10 |
| 2-linoleoyl-*sn*-glycerol | 2.11E-10 | 6.24E-11 | 2.21E-11 | 1.72E-10 | 4.07E-11 | 1.44E-11 |
| 2-arachidonoyl-*sn*-glycerol | 2.06E-09 | 5.21E-10 | 1.84E-10 | 1.6E-09 | 4.2E-10 | 1.48E-10 |
| **Free Fatty Acids** |  |  |  |  |  |  |
| Oleic acid | 1.45E-09 | 3.63E-10 | 1.28E-10 | 1.22E-09 | 2.83E-10 | 1E-10 |
| Linoleic acid | 9.65E-10 | 3.21E-10 | 1.13E-10 | 7.69E-10 | 1.98E-10 | 7.01E-11 |
| Arachidonic acid | 2.23E-09 | 2.03E-10 | 7.17E-11 | 1.93E-09 | 2.37E-10 | 8.37E-11 |
| **PhosphoLEA** |  |  |  |  |  |  |
| PhosphoLEA | 2.7E-11 | 7.23E-12 | 2.56E-12 | 2.11E-11 | 6.86E-12 | 2.43E-12 |
| **Prostaglandins** |  |  |  |  |  |  |
| PGE_2_ | 2.48E-10 | 4.01E-11 | 1.42E-11 | 2.64E-10 | 4.54E-11 | 1.6E-11 |
| PGF_2α_ | 2.59E-10 | 4.7E-11 | 1.66E-11 | 2.43E-10 | 3.71E-11 | 1.31E-11 |
| 6-ketoPGF_1α_ | PISSR |  |  | PISSR |  |  |
| **CBD/CP/THC** |  |  |  |  |  |  |
| Cannabidiol | BDL |  |  | BDL |  |  |
| CP 55,940 | BDL |  |  | 2.08E-10 | 2.8E-11 | 9.91E-12 |
| THC | BDL |  |  | BDL |  |  |
| **THC Metabolites** |  |  |  |  |  |  |
| 11-nor-9-carboxyTHC | BDL |  |  | BDL |  |  |
| 11-OH-THC | BDL |  |  | BDL |  |  |

**Supplemental Table 34.** List of lipids in the striatum significantly affected by 3mg/kg CP 55,940: Adult

| Adult Striatum Significant Differences in One-Way ANOVA | | | | |
| --- | --- | --- | --- | --- |
| Lipid | F | p | Direction (relative to Veh) | Magnitude (x Veh level) |
| *N*-oleoyl ethanolamine | 4.955 | .043 | ↓ | 0.65 |
| *N*-linoleoyl ethanolamine | 7.65 | .015 | ↓ | 0.68 |
| *N*-arachidonoyl ethanolamine | 7.29 | .017 | ↓ | 0.66 |
| *N*-docosahexaenoyl ethanolamine | 10.20 | .007 | ↓ | 0.75 |
| *N*-oleoyl leucine | 4.95 | .043 | ↑ | 1.20 |
| *N*-palmitoyl serine | 3.70 | .075 | ↓ | 0.79 |
| 2-arachidonoyl glycerol | 3.81 | .071 | ↓ | 0.78 |
| Arachidonic acid | 7.07 | .019 | ↓ | 0.87 |
| CP 55,940 | 441.34 | .000 | ↑ | infinite |

**Supplemental Table 35.** Lipid levels in the hippocampus of WT adult female mice treated with Vehicle or 3 mg/kg CP 55,940

|  | Hippocampus | | | | | | |
| --- | --- | --- | --- | --- | --- | --- | --- |
|  | Vehicle | | | 3mg/kg CP 55,940 | | | |
| ***N*-acyl alanine** | Mean | Std Dev | Std Error | | Mean | Std Dev | Std Error |
| *N*-palmitoyl alanine | 4.22E-12 | 1.62E-12 | 5.73E-13 | | 3.64E-12 | 1.46E-12 | 5.17E-13 |
| *N*-stearoyl alanine | 2.57E-12 | 8.26E-13 | 2.92E-13 | | 2.38E-12 | 4.06E-13 | 1.44E-13 |
| *N*-oleoyl alanine | 3.62E-12 | 1.2E-12 | 4.24E-13 | | 3.73E-12 | 1.56E-12 | 5.52E-13 |
| *N*-linoleoyl alanine | 2.61E-13 | 1.51E-13 | 5.35E-14 | | 3.37E-13 | 2.62E-13 | 9.28E-14 |
| *N*-arachidonoyl alanine | 4.82E-12 | 1.32E-12 | 4.67E-13 | | 3.66E-12 | 9.58E-13 | 3.39E-13 |
| *N*-docosahexaenoyl alanine | PISSR |  |  | | PISSR |  |  |
| ***N*-acyl dopamine** |  |  |  | |  |  |  |
| *N*-oleoyl dopamine | BDL |  |  | | BDL |  |  |
| *N*-arachidonoyl dopamine | BDL |  |  | | BDL |  |  |
| ***N*-acyl ethanolamine** |  |  |  | |  |  |  |
| *N*-palmitoyl ethanolamine | 4.9E-11 | 2.28E-11 | 8.07E-12 | | 4.41E-11 | 1.63E-11 | 5.77E-12 |
| *N*-stearoyl ethanolamine | 2.06E-11 | 3.35E-11 | 1.18E-11 | | 1.7E-11 | 3.16E-11 | 1.12E-11 |
| *N*-oleoyl ethanolamine | 1.18E-10 | 5.19E-11 | 1.84E-11 | | 1.06E-10 | 5.04E-11 | 1.78E-11 |
| *N*-linoleoyl ethanolamine | 3.25E-11 | 1.11E-11 | 3.91E-12 | | 2.58E-11 | 9.3E-12 | 3.29E-12 |
| *N*-arachidonoyl ethanolamine | 3.85E-11 | 9.72E-12 | 3.44E-12 | | 3.15E-11 | 9.15E-12 | 3.23E-12 |
| *N*-docosahexaenoyl ethanolamine | 2.49E-11 | 5.74E-12 | 2.03E-12 | | 2.18E-11 | 5.12E-12 | 1.81E-12 |
| ***N*-acyl GABA** |  |  |  | |  |  |  |
| *N*-palmitoyl GABA | 2.19E-11 | 2.41E-12 | 8.51E-13 | | 2.14E-11 | 3.35E-12 | 1.18E-12 |
| *N*-stearoyl GABA | 1.74E-11 | 2.1E-12 | 7.42E-13 | | 1.57E-11 | 2.57E-12 | 9.1E-13 |
| *N*-oleoyl GABA | 1.77E-11 | 1.97E-12 | 6.98E-13 | | 1.55E-11 | 2.83E-12 | 1E-12 |
| *N*-linoleoyl GABA | PISSR |  |  | | PISSR |  |  |
| *N*-arachidonoyl GABA | 3.42E-11 | 2.19E-12 | 7.73E-13 | | 2.86E-11 | 2.3E-12 | 8.13E-13 |
| *N*-docosahexaenoyl GABA | 8.49E-12 | 9.26E-13 | 3.27E-13 | | 7.15E-12 | 1.36E-12 | 4.82E-13 |
| ***N*-acyl glycine** |  |  |  | |  |  |  |
| *N*-palmitoyl glycine | 1.08E-12 | 3.77E-13 | 1.33E-13 | | 1.22E-12 | 6.22E-13 | 2.2E-13 |
| *N*-stearoyl glycine | 2.76E-12 | 1.21E-12 | 4.27E-13 | | 2.12E-12 | 1.23E-12 | 4.35E-13 |
| *N*-oleoyl glycine | 5.46E-13 | 2.42E-13 | 8.57E-14 | | 5.21E-13 | 3.23E-13 | 1.14E-13 |
| *N*-linoleoyl glycine | 1.04E-13 | 8.21E-14 | 2.9E-14 | | 1.16E-13 | 7.03E-14 | 2.49E-14 |
| *N*-arachidonoyl glycine | 2.83E-12 | 7.92E-13 | 2.8E-13 | | 2.03E-12 | 7.09E-13 | 2.51E-13 |
| *N*-docosahexaenoyl glycine | 1.15E-13 | 3.93E-14 | 1.39E-14 | | 1.21E-13 | 4.9E-14 | 1.73E-14 |
| ***N*-acyl leucine** |  |  |  | |  |  |  |
| *N*-palmitoyl leucine | 2.28E-12 | 1.98E-13 | 6.99E-14 | | 2.6E-12 | 5.83E-13 | 2.06E-13 |
| *N*-stearoyl leucine | 2.15E-12 | 4.48E-13 | 1.58E-13 | | 2.18E-12 | 4.3E-13 | 1.52E-13 |
| *N*-oleoyl leucine | 1.38E-12 | 2.26E-13 | 8E-14 | | 1.44E-12 | 1.56E-13 | 5.52E-14 |
| *N*-linoleoyl leucine | PISSR |  |  | | PISSR |  |  |
| *N*-docosahexaenoyl leucine | PISSR |  |  | | PISSR |  |  |
| ***N*-acyl methionine** |  |  |  | |  |  |  |
| *N*-palmitoyl methionine | 1.31E-12 | 7.25E-13 | 2.56E-13 | | 1.73E-12 | 5.68E-13 | 2.01E-13 |
| *N*-stearoyl methionine | PISSR |  |  | | PISSR |  |  |
| *N*-oleoyl methionine | PISSR |  |  | | PISSR |  |  |
| *N*-linoleoyl methionine | BDL |  |  | | BDL |  |  |
| *N*-arachidonoyl methionine | PISSR |  |  | | PISSR |  |  |
| *N*-docosahexaenoyl methionine | BDL |  |  | | BDL |  |  |
| ***N*-acyl phenylalanine** |  |  |  | |  |  |  |
| *N*-palmitoyl phenylalanine | 3.65E-13 | 1.26E-13 | 4.44E-14 | | 4.39E-13 | 7.97E-14 | 2.82E-14 |
| *N*-stearoyl phenylalanine | 3.95E-13 | 1.47E-13 | 5.21E-14 | | 3.31E-13 | 1.39E-13 | 4.91E-14 |
| *N*-oleoyl phenylalanine | 5.69E-13 | 2.02E-13 | 7.15E-14 | | 3.98E-13 | 1.08E-13 | 3.82E-14 |
| *N*-linoleoyl phenylalanine | PISSR |  |  | | PISSR |  |  |
| *N*-arachidonoyl phenylalanine | 1.88E-13 | 8.78E-14 | 3.1E-14 | | 1.12E-13 | 4.68E-14 | 1.65E-14 |
| *N*-docosahexaenoyl phenylalanine | PISSR |  |  | | PISSR |  |  |

**Supplemental Table 35: Continued**

|  | Hippocampus | | | | | |
| --- | --- | --- | --- | --- | --- | --- |
|  | Vehicle | | | 3mg/kg CP 55,940 | | |
| ***N*-acyl proline** | Mean | Std Dev | Std Error | Mean | Std Dev | Std Error |
| *N*-palmitoyl proline | BDL |  |  | BDL |  |  |
| *N*-stearoyl proline | BDL |  |  | BDL |  |  |
| *N-*oleoyl proline | BDL |  |  | BDL |  |  |
| *N*-linoleoyl proline | BDL |  |  | BDL |  |  |
| *N*-arachidonoyl proline | BDL |  |  | BDL |  |  |
| *N*-docosahexaenoyl proline | BDL |  |  | BDL |  |  |
| ***N*-acyl serine** |  |  |  |  |  |  |
| *N*-palmitoyl serine | 1.76E-11 | 5.26E-12 | 1.86E-12 | 1.33E-11 | 6.1E-12 | 2.16E-12 |
| *N*-stearoyl serine | 3.28E-11 | 3.26E-12 | 1.15E-12 | 2.78E-11 | 5.61E-12 | 1.98E-12 |
| *N*-oleoyl serine | 3.65E-10 | 3.06E-11 | 1.08E-11 | 3.49E-10 | 2.38E-11 | 8.42E-12 |
| *N*-linoleoyl serine | 7.15E-11 | 6.68E-12 | 2.36E-12 | 7.17E-11 | 8.04E-12 | 2.84E-12 |
| *N*-arachidonoyl serine | PISSR |  |  | PISSR |  |  |
| *N*-docosahexaenoyl serine | PISSR |  |  | PISSR |  |  |
| ***N*-acyl taurine** |  |  |  |  |  |  |
| *N*-arachidonoyl taurine | 2.37E-10 | 3.64E-11 | 1.29E-11 | 2.3E-10 | 2.72E-11 | 9.62E-12 |
| ***N*-acyl tryptophan** |  |  |  |  |  |  |
| *N*-palmitoyl tryptophan | BDL |  |  | BDL |  |  |
| *N*-stearoyl tryptophan | BDL |  |  | BDL |  |  |
| *N*-oleoyl tryptophan | BDL |  |  | BDL |  |  |
| *N*-linoleoyl tryptophan | BDL |  |  | BDL |  |  |
| *N*-arachidonoyl tryptophan | BDL |  |  | BDL |  |  |
| *N*-docosahexaenoyl tryptophan | BDL |  |  | BDL |  |  |
| ***N*-acyl tyrosine** |  |  |  |  |  |  |
| *N*-palmitoyl tyrosine | 1.08E-12 | 3.11E-13 | 1.1E-13 | 9.98E-13 | 3.44E-13 | 1.22E-13 |
| *N*-stearoyl tyrosine | 1.53E-13 | 6.46E-14 | 2.28E-14 | 1.97E-13 | 6.59E-14 | 2.33E-14 |
| *N-*oleoyl tyrosine | 5.09E-13 | 1.12E-13 | 3.95E-14 | 4.1E-13 | 2.46E-13 | 8.71E-14 |
| *N*-linoleoyl tyrosine | BDL |  |  | PISSR |  |  |
| *N*-arachidonoyl tyrosine | PISSR |  |  | PISSR |  |  |
| *N*-docosahexaenoyl tyrosine | PISSR |  |  | PISSR |  |  |
| ***N*-acyl valine** |  |  |  |  |  |  |
| *N*-palmitoyl valine | 1.06E-13 | 4.24E-14 | 1.5E-14 | 1.27E-13 | 7.24E-14 | 2.56E-14 |
| *N*- stearoyl valine | 3.02E-14 | 1.78E-14 | 6.29E-15 | 3.95E-14 | 3.04E-14 | 1.08E-14 |
| *N*-oleoyl valine | PISSR |  |  | PISSR |  |  |
| *N*-nervonoyl valine | BDL |  |  | BDL |  |  |
| *N*-linoleoyl valine | PISSR |  |  | PISSR |  |  |
| *N*-docosahexaenoyl valine | PISSR |  |  | PISSR |  |  |
| **2-acyl glycerols** |  |  |  |  |  |  |
| 2-palmitoyl glycerol | 1.74E-10 | 5.26E-11 | 1.86E-11 | 1.43E-10 | 2.27E-11 | 8.02E-12 |
| 2-oleoyl glycerol | 1.76E-09 | 6.06E-10 | 2.14E-10 | 1.58E-09 | 2.61E-10 | 9.21E-11 |
| 2-linoleoyl glycerol | 2.24E-10 | 5.9E-11 | 2.09E-11 | 1.64E-10 | 3.59E-11 | 1.27E-11 |
| 2-arachidonoyl glycerol | 2.41E-09 | 6.94E-10 | 2.45E-10 | 2.63E-09 | 3.97E-10 | 1.4E-10 |
| **Free Fatty Acids** |  |  |  |  |  |  |
| Oleic acid | 1.6E-09 | 4.11E-10 | 1.45E-10 | 1.43E-09 | 5.63E-10 | 1.99E-10 |
| Linoleic acid | 8.85E-10 | 3.54E-10 | 1.25E-10 | 7.37E-10 | 3.36E-10 | 1.19E-10 |
| Arachidonic acid | 2.1E-09 | 2.53E-10 | 8.95E-11 | 1.89E-09 | 4.66E-10 | 1.65E-10 |
| **PhosphoLEA** |  |  |  |  |  |  |
| PhosphoLEA | 1.24E-11 | 1.08E-12 | 3.83E-13 | 1.8E-11 | 2.84E-12 | 1E-12 |
| **Prostaglandins** |  |  |  |  |  |  |
| PGE_2_ | 2.85E-10 | 1.67E-11 | 5.91E-12 | 3.37E-10 | 2.24E-11 | 7.91E-12 |
| PGF_2α_ | 2.51E-10 | 2.46E-11 | 8.69E-12 | 3.25E-10 | 2.84E-11 | 1E-11 |
| 6-ketoPGF_1α_ | 2.35E-11 | 8.6E-12 | 3.04E-12 | 2.42E-11 | 8.85E-12 | 3.13E-12 |
| **THC/CP/CBD** |  |  |  |  |  |  |
| Cannabidiol | BDL |  |  | BDL |  |  |
| CP 55,940 | BDL |  |  | 2.68E-10 | 3.38E-11 | 1.2E-11 |
| THC | BDL |  |  | BDL |  |  |
| **THC Metabolites** |  |  |  |  |  |  |
| 11-nor-9-carboxyTHC | BDL |  |  | BDL |  |  |
| 11-OH-THC | BDL |  |  | BDL |  |  |

**Supplemental Table 36.** List of lipids in the hippocampus significantly affected by 3 mg/kg CP 55,940: Adult

| Hippocampus Significant Differences in One-Way ANOVA | | | | |
| --- | --- | --- | --- | --- |
| Lipid | F | p | Direction (relative to Veh) | Magnitude (x Veh level) |
| *N*-arachidonoyl alanine | 4.06 | .063 | ↓ | 0.76 |
| *N*-oleoyl GABA | 3.27 | .092 | ↓ | 0.88 |
| *N*-arachidonoyl GABA | 25.58 | .000 | ↓ | 0.84 |
| *N*-docosahexaenoyl GABA | 5.34 | .037 | ↓ | 0.84 |
| *N*-arachidonoyl glycine | 4.60 | .050 | ↓ | 0.72 |
| *N*-oleoyl phenylalanine | 4.48 | .053 | ↓ | 0.70 |
| *N*-arachidonoyl phenylalanine | 4.61 | .050 | ↓ | 0.60 |
| *N*-stearoyl serine | 4.68 | .048 | ↓ | 0.85 |
| 2-linoleoyl glycerol | 5.86 | .030 | ↓ | 0.74 |
| phosphoLEA | 26.89 | .000 | ↑ | 1.45 |
| PGE_2_ | 28.08 | .000 | ↑ | 1.18 |
| PGF_2α_ | 31.49 | .000 | ↑ | 1.30 |
| CP 55,940 | 77.23 | .000 | ↑ | infinite |

**Supplemental Table 37.** Lipid levels in the cerebellum of WT adult female mice treated with Vehicle or 3 mg/kg CP 55,940

|  | Cerebellum | | | | | | |
| --- | --- | --- | --- | --- | --- | --- | --- |
|  | Vehicle | | | 3mg/kg CP 55,940 | | | |
| ***N*-acyl alanine** | Mean | Std Dev | Std Error | | Mean | Std Dev | Std Error |
| *N*-palmitoyl alanine | 2.25E-12 | 9.86E-13 | 3.49E-13 | | 1.61E-12 | 3.57E-13 | 1.26E-13 |
| *N*-stearoyl alanine | 6.55E-12 | 3.24E-12 | 1.15E-12 | | 5.69E-12 | 1.84E-12 | 6.52E-13 |
| *N*-oleoyl alanine | 2.74E-12 | 1.13E-12 | 3.99E-13 | | 2.34E-12 | 5.92E-13 | 2.09E-13 |
| *N*-linoleoyl alanine | 1.83E-13 | 6.5E-14 | 2.3E-14 | | 2.18E-13 | 1.02E-13 | 3.6E-14 |
| *N*-arachidonoyl alanine | PISSR |  |  | | PISSR |  |  |
| *N*-docosahexaenoyl alanine | PISSR |  |  | | PISSR |  |  |
| ***N*-acyl dopamine** |  |  |  | |  |  |  |
| *N*-oleoyl dopamine | BDL |  |  | | BDL |  |  |
| *N*-arachidonoyl dopamine | BDL |  |  | | BDL |  |  |
| ***N*-acyl ethanolamine** |  |  |  | |  |  |  |
| *N*-palmitoyl ethanolamine | 7.58E-11 | 2.08E-11 | 7.34E-12 | | 6.47E-11 | 8.74E-12 | 3.09E-12 |
| *N*-stearoyl ethanolamine | 1.39E-11 | 1E-11 | 3.79E-12 | | 1.6E-11 | 7.88E-12 | 2.78E-12 |
| *N*-oleoyl ethanolamine | 2.43E-10 | 1.2E-10 | 4.53E-11 | | 2.04E-10 | 7.71E-11 | 2.72E-11 |
| *N*-linoleoyl ethanolamine | 2.91E-11 | 1.15E-11 | 4.08E-12 | | 1.87E-11 | 4.48E-12 | 1.58E-12 |
| *N*-arachidonoyl ethanolamine | 2.12E-11 | 9.42E-12 | 3.33E-12 | | 1.33E-11 | 3.32E-12 | 1.17E-12 |
| *N*-docosahexaenoyl ethanolamine | 3.81E-11 | 1.38E-11 | 4.88E-12 | | 2.78E-11 | 5.68E-12 | 2.01E-12 |
| ***N*-acyl GABA** |  |  |  | |  |  |  |
| *N*-palmitoyl GABA | 2.28E-11 | 3.17E-12 | 1.12E-12 | | 2.25E-11 | 2.45E-12 | 8.67E-13 |
| *N*-stearoyl GABA | 1.22E-11 | 3.34E-12 | 1.18E-12 | | 1.12E-11 | 1.79E-12 | 6.32E-13 |
| *N*-oleoyl GABA | 1.78E-11 | 2E-12 | 7.08E-13 | | 1.65E-11 | 1.69E-12 | 5.99E-13 |
| *N*-linoleoyl GABA | 9.31E-12 | 1.87E-12 | 6.62E-13 | | 7.7E-12 | 8.39E-13 | 2.97E-13 |
| *N*-arachidonoyl GABA | 1.86E-11 | 1.85E-12 | 6.55E-13 | | 1.22E-11 | 1.26E-12 | 4.44E-13 |
| *N*-docosahexaenoyl GABA | 1E-11 | 1.85E-12 | 6.53E-13 | | 8.3E-12 | 1.74E-12 | 6.15E-13 |
| ***N*-acyl glycine** |  |  |  | |  |  |  |
| *N*-palmitoyl glycine | 1.42E-12 | 7.35E-13 | 2.6E-13 | | 1.24E-12 | 8.73E-13 | 3.09E-13 |
| *N*-stearoyl glycine | 1.67E-12 | 1.01E-12 | 3.57E-13 | | 1.62E-12 | 1.48E-12 | 5.23E-13 |
| *N*-oleoyl glycine | 3.73E-13 | 3.64E-13 | 1.29E-13 | | 2.49E-13 | 2.28E-13 | 8.05E-14 |
| *N*-linoleoyl glycine | 6.74E-14 | 5.87E-14 | 2.07E-14 | | 6.99E-14 | 8.93E-14 | 3.16E-14 |
| *N*-arachidonoyl glycine | 3.16E-13 | 2.85E-13 | 1.01E-13 | | 1.43E-13 | 1.02E-13 | 3.59E-14 |
| *N*-docosahexaenoyl glycine | 1.13E-13 | 7.51E-14 | 2.66E-14 | | 7.91E-14 | 6.05E-14 | 2.14E-14 |
| ***N*-acyl leucine** |  |  |  | |  |  |  |
| *N*-palmitoyl leucine | 1.18E-12 | 3.74E-13 | 1.32E-13 | | 1.34E-12 | 1.97E-13 | 6.97E-14 |
| *N*-stearoyl leucine | 1.31E-12 | 4.21E-13 | 1.49E-13 | | 1.18E-12 | 2.2E-13 | 7.77E-14 |
| *N*-oleoyl leucine | 8.58E-13 | 3.09E-13 | 1.09E-13 | | 1.06E-12 | 2.58E-13 | 9.12E-14 |
| *N*-linoleoyl leucine | PISSR |  |  | | PISSR |  |  |
| *N*-docosahexaenoyl leucine | 1.25E-12 | 3.03E-13 | 1.07E-13 | | 1.51E-12 | 4.89E-13 | 1.73E-13 |
| ***N*-acyl methionine** |  |  |  | |  |  |  |
| *N*-palmitoyl methionine | 6.68E-12 | 1.81E-12 | 6.42E-13 | | 6.89E-12 | 1.81E-12 | 6.41E-13 |
| *N*-stearoyl methionine | 3.46E-13 | 1.64E-13 | 5.79E-14 | | 3.32E-13 | 1.47E-13 | 5.2E-14 |
| *N*-oleoyl methionine | PISSR |  |  | | PISSR |  |  |
| *N*-linoleoyl methionine | PISSR |  |  | | PISSR |  |  |
| *N*-arachidonoyl methionine | PISSR |  |  | | PISSR |  |  |
| *N*-docosahexaenoyl methionine | PISSR |  |  | | PISSR |  |  |
| ***N*-acyl phenylalanine** |  |  |  | |  |  |  |
| *N*-palmitoyl phenylalanine | 3.06E-13 | 2.36E-13 | 8.35E-14 | | 1.87E-13 | 7.7E-14 | 2.72E-14 |
| *N*-stearoyl phenylalanine | 3.26E-13 | 1.1E-13 | 3.9E-14 | | 2.74E-13 | 1.08E-13 | 3.82E-14 |
| *N*-oleoyl phenylalanine | 7.03E-13 | 2.48E-13 | 8.78E-14 | | 6.03E-13 | 2.53E-13 | 8.96E-14 |
| *N*-linoleoyl phenylalanine | PISSR |  |  | | PISSR |  |  |
| *N*-arachidonoyl phenylalanine | 1.09E-13 | 7.05E-14 | 2.49E-14 | | 4.52E-14 | 3.98E-14 | 1.41E-14 |
| *N*-docosahexaenoyl phenylalanine | 4.73E-14 | 3.78E-14 | 1.34E-14 | | 3.04E-14 | 2.15E-14 | 7.62E-15 |

**Supplemental Table 37: Continued**

|  | Cerebellum | | | | | |
| --- | --- | --- | --- | --- | --- | --- |
|  | Vehicle | | | 3mg/kg CP 55,940 | | |
| ***N*-acyl proline** | Mean | Std Dev | Std Error | Mean | Std Dev | Std Error |
| *N*-palmitoyl proline | BDL |  |  | BDL |  |  |
| *N*-stearoyl proline | BDL |  |  | BDL |  |  |
| *N-*oleoyl proline | BDL |  |  | BDL |  |  |
| *N*-linoleoyl proline | BDL |  |  | BDL |  |  |
| *N*-arachidonoyl proline | BDL |  |  | BDL |  |  |
| *N*-docosahexaenoyl proline | BDL |  |  | BDL |  |  |
| ***N*-acyl serine** |  |  |  |  |  |  |
| *N*-palmitoyl serine | 7.42E-12 | 3.43E-12 | 1.21E-12 | 5.77E-12 | 2.84E-12 | 1E-12 |
| *N*-stearoyl serine | 1.77E-11 | 5.25E-12 | 1.86E-12 | 1.52E-11 | 2.68E-12 | 9.48E-13 |
| *N*-oleoyl serine | 1.46E-10 | 5.68E-11 | 2.01E-11 | 1.05E-10 | 2.68E-11 | 9.47E-12 |
| *N*-linoleoyl serine | 2.54E-11 | 1.05E-11 | 3.72E-12 | 1.99E-11 | 6.88E-12 | 2.43E-12 |
| *N*-arachidonoyl serine | PISSR |  |  | PISSR |  |  |
| *N*-docosahexaenoyl serine | PISSR |  |  | PISSR |  |  |
| ***N*-acyl taurine** |  |  |  |  |  |  |
| *N*-arachidonoyl taurine | 8.54E-11 | 2E-11 | 7.08E-12 | 6.54E-11 | 1.97E-11 | 6.97E-12 |
| ***N*-acyl tryptophan** |  |  |  |  |  |  |
| *N*-palmitoyl tryptophan | BDL |  |  | BDL |  |  |
| *N*-stearoyl tryptophan | BDL |  |  | BDL |  |  |
| *N*-oleoyl tryptophan | BDL |  |  | BDL |  |  |
| *N*-linoleoyl tryptophan | BDL |  |  | BDL |  |  |
| *N*-arachidonoyl tryptophan | BDL |  |  | BDL |  |  |
| *N*-docosahexaenoyl tryptophan | BDL |  |  | BDL |  |  |
| ***N*-acyl tyrosine** |  |  |  |  |  |  |
| *N*-palmitoyl tyrosine | 1.33E-12 | 4.06E-13 | 1.44E-13 | 9.82E-13 | 2.8E-13 | 9.91E-14 |
| *N*-stearoyl tyrosine | PISSR |  |  | PISSR |  |  |
| *N-*oleoyl tyrosine | 8.81E-13 | 4.19E-13 | 1.48E-13 | 8.96E-13 | 3.26E-13 | 1.15E-13 |
| *N*-linoleoyl tyrosine | PISSR |  |  | PISSR |  |  |
| *N*-arachidonoyl tyrosine | PISSR |  |  | PISSR |  |  |
| *N*-docosahexaenoyl tyrosine | PISSR |  |  | PISSR |  |  |
| ***N*-acyl valine** |  |  |  |  |  |  |
| *N*-palmitoyl valine | 8.7E-14 | 2.73E-14 | 9.64E-15 | 1.08E-13 | 6.74E-14 | 2.38E-14 |
| *N*- stearoyl valine | PISSR |  |  | PISSR |  |  |
| *N*-oleoyl valine | 1.18E-14 | 8.42E-15 | 2.98E-15 | 2.42E-14 | 1.11E-14 | 3.94E-15 |
| *N*-nervonoyl valine | BDL |  |  | BDL |  |  |
| *N*-linoleoyl valine | PISSR |  |  | PISSR |  |  |
| *N*-docosahexaenoyl valine | PISSR |  |  | PISSR |  |  |
| **2-acyl glycerols** |  |  |  |  |  |  |
| 2-palmitoyl glycerol | 1.19E-10 | 3.7E-11 | 1.4E-11 | 1.01E-10 | 3.64E-11 | 1.29E-11 |
| 2-oleoyl glycerol | 2.64E-09 | 9.26E-10 | 3.27E-10 | 1.94E-09 | 4.31E-10 | 1.52E-10 |
| 2-linoleoyl glycerol | 3.7E-10 | 1.07E-10 | 3.77E-11 | 2.44E-10 | 4.49E-11 | 1.59E-11 |
| 2-arachidonoyl glycerol | 5.33E-09 | 9.82E-10 | 3.47E-10 | 4.32E-09 | 5.55E-10 | 1.96E-10 |
| **Free Fatty Acids** |  |  |  |  |  |  |
| Oleic acid | 1.01E-09 | 1.86E-10 | 6.57E-11 | 6.36E-10 | 9.27E-11 | 3.28E-11 |
| Linoleic acid | 1.08E-09 | 1.82E-10 | 6.44E-11 | 6.75E-10 | 5.34E-11 | 1.89E-11 |
| Arachidonic acid | 1.12E-09 | 1.3E-10 | 4.58E-11 | 7.51E-10 | 1.09E-10 | 3.84E-11 |
| **PhosphoLEA** |  |  |  |  |  |  |
| PhosphoLEA | 6.53E-12 | 5.26E-13 | 1.86E-13 | 4.64E-12 | 5.29E-13 | 1.87E-13 |
| **Prostaglandins** |  |  |  |  |  |  |
| PGE_2_ | 3.08E-10 | 4.39E-11 | 1.55E-11 | 2.14E-10 | 2.33E-11 | 8.25E-12 |
| PGF_2α_ | 3.31E-10 | 4.06E-11 | 1.43E-11 | 2.22E-10 | 3.4E-11 | 1.2E-11 |
| 6-ketoPGF_1α_ | 3.29E-11 | 7.8E-12 | 2.76E-12 | 2.25E-11 | 7.82E-12 | 2.77E-12 |
| **THC/CP/CBD** |  |  |  |  |  |  |
| Cannabidiol | BDL |  |  | BDL |  |  |
| CP 55,940 | BDL |  |  | 2.8E-10 | 4.05E-11 | 1.43E-11 |
| THC | BDL |  |  | BDL |  |  |
| **THC Metabolites** |  |  |  |  |  |  |
| 11-nor-9-carboxyTHC | BDL |  |  | BDL |  |  |
| 11-OH-THC | BDL |  |  | BDL |  |  |

**Supplemental Table 38.** List of lipids in the cerebellum significantly affected by 3 mg/kg CP 55,940: Adult

| Cerebellum Significant Differences in One-Way ANOVA | | | | |
| --- | --- | --- | --- | --- |
| Lipid | F | p | Direction (relative to Veh) | Magnitude (x Veh level) |
| *N*-linoleoyl ethanolamine | 5.69 | .032 | ↓ | 0.64 |
| *N*-arachidonoyl ethanolamine | 5.06 | .041 | ↓ | 0.63 |
| *N*-docosahexaenoyl ethanolamine | 3.83 | .070 | ↓ | 0.73 |
| *N*-linoleoyl GABA | 4.90 | .044 | ↓ | 0.83 |
| *N*-arachidonoyl GABA | 66.96 | .000 | ↓ | 0.65 |
| *N*-docosahexaenoyl GABA | 3.75 | .073 | ↓ | 0.83 |
| *N*-arachidonoyl phenylalanine | 5.04 | .041 | ↓ | 0.41 |
| *N*-oleoyl serine | 3.40 | .086 | ↓ | 0.72 |
| *N*-arachidonoyl taurine | 4.04 | .064 | ↓ | 0.77 |
| *N-*palmitoyl tyrosine | 4.01 | .065 | ↓ | 0.74 |
| *N*-oleoyl valine | 6.29 | .025 | ↑ | 2.05 |
| 2-oleoyl glycerol | 3.68 | .076 | ↓ | 0.74 |
| 2-linoleoyl glycerol | 9.51 | .008 | ↓ | 0.66 |
| 2-arachidonoyl glycerol | 6.46 | .023 | ↓ | 0.81 |
| Oleic acid | 26.55 | .000 | ↓ | 0.63 |
| Linoleic acid | 36.23 | .000 | ↓ | 0.63 |
| Arachidonic acid | 38.69 | .000 | ↓ | 0.67 |
| phosphoLEA | 51.27 | .000 | ↓ | 0.71 |
| PGE_2_ | 28.54 | .000 | ↓ | 0.70 |
| PGF_2α_ | 34.08 | .000 | ↓ | 0.67 |
| 6-ketoPGF_1α_ | 7.06 | .019 | ↓ | 0.68 |
| CP 55,940 | 381.82 | .000 | ↑ | infinite |
| Sample Mass | 4.97 | .043 | ↑ | 1.10 |

**Supplemental Table 39.** Lipid levels in the thalamus of WT adult female mice treated with Vehicle or 3 mg/kg CP 55,940

|  | Thalamus | | | | | | |
| --- | --- | --- | --- | --- | --- | --- | --- |
|  | Vehicle | | | 3mg/kg CP 55,940 | | | |
| ***N*-acyl alanine** | Mean | Std Dev | Std Error | | Mean | Std Dev | Std Error |
| *N*-palmitoyl alanine | 2.39E-12 | 1.14E-12 | 4.03E-13 | | 2.22E-12 | 9.74E-13 | 3.44E-13 |
| *N*-stearoyl alanine | 2.95E-12 | 9.44E-13 | 3.34E-13 | | 2.97E-12 | 8.05E-13 | 2.85E-13 |
| *N*-oleoyl alanine | 2.46E-12 | 8.18E-13 | 2.89E-13 | | 2.34E-12 | 8.78E-13 | 3.11E-13 |
| *N*-linoleoyl alanine | 5.7E-13 | 2.05E-13 | 7.24E-14 | | 5.44E-13 | 1.5E-13 | 5.3E-14 |
| *N*-arachidonoyl alanine | 1.5E-12 | 7.29E-13 | 2.58E-13 | | 9.44E-13 | 4.48E-13 | 1.58E-13 |
| *N*-docosahexaenoyl alanine | PISSR |  |  | | PISSR |  |  |
| ***N*-acyl dopamine** |  |  |  | |  |  |  |
| *N*-oleoyl dopamine | BDL |  |  | | BDL |  |  |
| *N*-arachidonoyl dopamine | BDL |  |  | | BDL |  |  |
| ***N*-acyl ethanolamine** |  |  |  | |  |  |  |
| *N*-palmitoyl ethanolamine | 7.52E-11 | 9.65E-12 | 3.41E-12 | | 6.49E-11 | 7.6E-12 | 2.69E-12 |
| *N*-stearoyl ethanolamine | 1.18E-11 | 3.08E-12 | 1.17E-12 | | 1.06E-11 | 3.78E-12 | 1.34E-12 |
| *N*-oleoyl ethanolamine | 2.02E-10 | 3.99E-11 | 1.41E-11 | | 1.6E-10 | 4.48E-11 | 1.58E-11 |
| *N*-linoleoyl ethanolamine | 2.05E-11 | 5.69E-12 | 2.01E-12 | | 1.72E-11 | 4.25E-12 | 1.5E-12 |
| *N*-arachidonoyl ethanolamine | 2.1E-11 | 5.02E-12 | 1.77E-12 | | 1.69E-11 | 2.93E-12 | 1.04E-12 |
| *N*-docosahexaenoyl ethanolamine | 3.44E-11 | 9.4E-12 | 3.32E-12 | | 2.99E-11 | 6.33E-12 | 2.24E-12 |
| ***N*-acyl GABA** |  |  |  | |  |  |  |
| *N*-palmitoyl GABA | 2.42E-11 | 2.77E-12 | 9.79E-13 | | 2.74E-11 | 3.44E-12 | 1.22E-12 |
| *N*-stearoyl GABA | 1.7E-11 | 3.39E-12 | 1.2E-12 | | 1.7E-11 | 3.14E-12 | 1.11E-12 |
| *N*-oleoyl GABA | 1.72E-11 | 2.4E-12 | 8.49E-13 | | 1.73E-11 | 2.38E-12 | 8.43E-13 |
| *N*-linoleoyl GABA | 8.91E-12 | 2.4E-12 | 8.5E-13 | | 7.91E-12 | 1.83E-12 | 6.47E-13 |
| *N*-arachidonoyl GABA | 2.54E-11 | 2.73E-12 | 9.65E-13 | | 2.04E-11 | 2.44E-12 | 8.61E-13 |
| *N*-docosahexaenoyl GABA | 9.11E-12 | 1.96E-12 | 6.92E-13 | | 9.36E-12 | 1.49E-12 | 5.27E-13 |
| ***N*-acyl glycine** |  |  |  | |  |  |  |
| *N*-palmitoyl glycine | 2.18E-12 | 1.05E-12 | 3.72E-13 | | 2.5E-12 | 1.15E-12 | 4.06E-13 |
| *N*-stearoyl glycine | 1.56E-12 | 1E-12 | 3.54E-13 | | 1.83E-12 | 1.79E-12 | 6.32E-13 |
| *N*-oleoyl glycine | 5.45E-13 | 4.81E-13 | 1.7E-13 | | 4.19E-13 | 3.3E-13 | 1.17E-13 |
| *N*-linoleoyl glycine | 1.11E-13 | 7.9E-14 | 2.79E-14 | | 9.08E-14 | 6.48E-14 | 2.29E-14 |
| *N*-arachidonoyl glycine | 8.5E-13 | 3.01E-13 | 1.07E-13 | | 5.38E-13 | 1.32E-13 | 4.67E-14 |
| *N*-docosahexaenoyl glycine | 1.78E-13 | 1.4E-13 | 4.96E-14 | | 1.3E-13 | 5.47E-14 | 1.93E-14 |
| ***N*-acyl leucine** |  |  |  | |  |  |  |
| *N*-palmitoyl leucine | 1.8E-12 | 1.52E-13 | 5.38E-14 | | 2.46E-12 | 2.56E-13 | 9.05E-14 |
| *N*-stearoyl leucine | 1.06E-12 | 2.05E-13 | 7.24E-14 | | 1.05E-12 | 2.86E-13 | 1.01E-13 |
| *N*-oleoyl leucine | 7.89E-13 | 2.2E-13 | 7.78E-14 | | 7.81E-13 | 2.05E-13 | 7.24E-14 |
| *N*-linoleoyl leucine | PISSR |  |  | | PISSR |  |  |
| *N*-docosahexaenoyl leucine | PISSR |  |  | | PISSR |  |  |
| ***N*-acyl methionine** |  |  |  | |  |  |  |
| *N*-palmitoyl methionine | 3.06E-12 | 8.26E-13 | 2.92E-13 | | 3.82E-12 | 8.35E-13 | 2.95E-13 |
| *N*-stearoyl methionine | 6.15E-13 | 2.45E-13 | 8.65E-14 | | 4.98E-13 | 1.69E-13 | 5.96E-14 |
| *N*-oleoyl methionine | 5.5E-13 | 2.43E-13 | 8.58E-14 | | 5.74E-13 | 2.42E-13 | 8.56E-14 |
| *N*-linoleoyl methionine | BDL |  |  | | BDL |  |  |
| *N*-arachidonoyl methionine | PISSR |  |  | | PISSR |  |  |
| *N*-docosahexaenoyl methionine | BDL |  |  | | BDL |  |  |
| ***N*-acyl phenylalanine** |  |  |  | |  |  |  |
| *N*-palmitoyl phenylalanine | 3.43E-13 | 2.38E-13 | 8.43E-14 | | 4.13E-13 | 7.99E-14 | 2.82E-14 |
| *N*-stearoyl phenylalanine | 3.12E-13 | 1.72E-13 | 6.1E-14 | | 2.37E-13 | 8.66E-14 | 3.06E-14 |
| *N*-oleoyl phenylalanine | 5.22E-13 | 1.43E-13 | 5.04E-14 | | 4.73E-13 | 1.64E-13 | 5.78E-14 |
| *N*-linoleoyl phenylalanine | PISSR |  |  | | PISSR |  |  |
| *N*-arachidonoyl phenylalanine | 1.6E-13 | 5.71E-14 | 2.02E-14 | | 1.15E-13 | 5.35E-14 | 1.89E-14 |
| *N*-docosahexaenoyl phenylalanine | 5.85E-14 | 4.63E-14 | 1.64E-14 | | 4.66E-14 | 1.76E-14 | 6.23E-15 |

**Supplemental Table 39: Continued**

|  | Thalamus | | | | | |
| --- | --- | --- | --- | --- | --- | --- |
|  | Vehicle | | | 3mg/kg CP 55,940 | | |
| ***N*-acyl proline** | Mean | Std Dev | Std Error | Mean | Std Dev | Std Error |
| *N*-palmitoyl proline | BDL |  |  | BDL |  |  |
| *N*-stearoyl proline | BDL |  |  | BDL |  |  |
| *N-*oleoyl proline | BDL |  |  | BDL |  |  |
| *N*-linoleoyl proline | BDL |  |  | BDL |  |  |
| *N*-arachidonoyl proline | BDL |  |  | BDL |  |  |
| *N*-docosahexaenoyl proline | BDL |  |  | BDL |  |  |
| ***N*-acyl serine** |  |  |  |  |  |  |
| *N*-palmitoyl serine | 1.03E-11 | 2.94E-12 | 1.04E-12 | 1.13E-11 | 3.2E-12 | 1.13E-12 |
| *N*-stearoyl serine | 2.14E-11 | 4.22E-12 | 1.49E-12 | 2.33E-11 | 4.58E-12 | 1.62E-12 |
| *N*-oleoyl serine | 1.92E-10 | 4.03E-11 | 1.42E-11 | 2E-10 | 4.66E-11 | 1.65E-11 |
| *N*-linoleoyl serine | 4.18E-11 | 1.16E-11 | 4.12E-12 | 4.53E-11 | 9.97E-12 | 3.52E-12 |
| *N*-arachidonoyl serine | PISSR |  |  | PISSR |  |  |
| *N*-docosahexaenoyl serine | PISSR |  |  | PISSR |  |  |
| ***N*-acyl taurine** |  |  |  |  |  |  |
| *N*-arachidonoyl taurine | 5.41E-11 | 4.25E-12 | 1.5E-12 | 5.3E-11 | 2.43E-12 | 8.59E-13 |
| ***N*-acyl tryptophan** |  |  |  |  |  |  |
| *N*-palmitoyl tryptophan | BDL |  |  | BDL |  |  |
| *N*-stearoyl tryptophan | BDL |  |  | BDL |  |  |
| *N*-oleoyl tryptophan | BDL |  |  | BDL |  |  |
| *N*-linoleoyl tryptophan | BDL |  |  | BDL |  |  |
| *N*-arachidonoyl tryptophan | BDL |  |  | BDL |  |  |
| *N*-docosahexaenoyl tryptophan | BDL |  |  | BDL |  |  |
| ***N*-acyl tyrosine** |  |  |  |  |  |  |
| *N*-palmitoyl tyrosine | 4.98E-13 | 4.64E-13 | 1.64E-13 | 3.42E-13 | 1.86E-13 | 6.57E-14 |
| *N*-stearoyl tyrosine | 9.1E-14 | 5.4E-14 | 1.91E-14 | 8.82E-14 | 3.74E-14 | 1.32E-14 |
| *N-*oleoyl tyrosine | PISSR |  |  | PISSR |  |  |
| *N*-linoleoyl tyrosine | PISSR |  |  | PISSR |  |  |
| *N*-arachidonoyl tyrosine | 6.57E-13 | 2.8E-13 | 9.89E-14 | 6.37E-13 | 2.79E-13 | 9.87E-14 |
| *N*-docosahexaenoyl tyrosine | BDL |  |  | PISSR |  |  |
| ***N*-acyl valine** |  |  |  |  |  |  |
| *N*-palmitoyl valine | 9.73E-14 | 5.86E-14 | 2.07E-14 | 1.9E-13 | 5.86E-14 | 2.07E-14 |
| *N*- stearoyl valine | PISSR |  |  | PISSR |  |  |
| *N*-oleoyl valine | PISSR |  |  | PISSR |  |  |
| *N*-nervonoyl valine | BDL |  |  | BDL |  |  |
| *N*-linoleoyl valine | BDL |  |  | BDL |  |  |
| *N*-docosahexaenoyl valine | BDL |  |  | BDL |  |  |
| **2-acyl glycerols** |  |  |  |  |  |  |
| 2-palmitoyl glycerol | 1.04E-10 | 3.21E-11 | 1.14E-11 | 8.84E-11 | 1.03E-11 | 3.64E-12 |
| 2-oleoyl glycerol | 3.83E-09 | 5.99E-10 | 2.12E-10 | 3.61E-09 | 5.47E-10 | 1.93E-10 |
| 2-linoleoyl glycerol | 3.53E-10 | 4.7E-11 | 1.66E-11 | 2.71E-10 | 2.06E-11 | 7.29E-12 |
| 2-arachidonoyl glycerol | 3.71E-09 | 2.77E-10 | 1.05E-10 | 3E-09 | 2.93E-10 | 1.04E-10 |
| **Free Fatty Acids** |  |  |  |  |  |  |
| Oleic acid | 5.82E-10 | 5.58E-11 | 1.97E-11 | 4.55E-10 | 4.52E-11 | 1.6E-11 |
| Linoleic acid | 6.48E-10 | 2.34E-10 | 8.28E-11 | 4.92E-10 | 1.49E-10 | 5.28E-11 |
| Arachidonic acid | 1.12E-09 | 2.37E-10 | 8.39E-11 | 9.32E-10 | 1.73E-10 | 6.12E-11 |
| **PhosphoLEA** |  |  |  |  |  |  |
| PhosphoLEA | 4.81E-12 | 1.8E-12 | 6.38E-13 | 3.87E-12 | 1.32E-12 | 4.68E-13 |
| **Prostaglandins** |  |  |  |  |  |  |
| PGE_2_ | 1.95E-10 | 3.07E-11 | 1.09E-11 | 1.93E-10 | 2.89E-11 | 1.02E-11 |
| PGF_2α_ | 2.21E-10 | 3.18E-11 | 1.12E-11 | 2.22E-10 | 3.13E-11 | 1.11E-11 |
| 6-ketoPGF_1α_ | 2.71E-11 | 2.68E-12 | 9.47E-13 | 3.33E-11 | 4.9E-12 | 1.73E-12 |
| **THC/CP/CBD** |  |  |  |  |  |  |
| Cannabidiol | BDL |  |  | BDL |  |  |
| CP 55,940 | BDL |  |  | 2E-10 | 2.21E-11 | 7.83E-12 |
| THC | BDL |  |  | BDL |  |  |
| **THC Metabolites** |  |  |  |  |  |  |
| 11-nor-9-carboxyTHC | BDL |  |  | BDL |  |  |
| 11-OH-THC | BDL |  |  | BDL |  |  |

**Supplemental Table 40.** List of lipids in the thalamus significantly affected by 3 mg/kg CP 55,940: Adult

| Adult Thalamus Significant Differences in One-Way ANOVA | | | | |
| --- | --- | --- | --- | --- |
| Lipid | F | p | Direction (relative to Veh) | Magnitude (x Veh level) |
| *N*-arachidonoyl alanine | 3.32 | .090 | ↓ | 0.63 |
| *N*-palmitoyl ethanolamine | 5.62 | .033 | ↓ | 0.86 |
| *N*-oleoyl ethanolamine | 4.03 | .065 | ↓ | 0.79 |
| *N*-arachidonoyl ethanolamine | 3.92 | .068 | ↓ | 0.81 |
| *N*-palmitoyl GABA | 4.00 | .065 | ↑ | 1.13 |
| *N*-arachidonoyl GABA | 15.29 | .002 | ↓ | 0.80 |
| *N*-arachidonoyl glycine | 7.21 | .018 | ↓ | 0.63 |
| *N*-palmitoyl leucine | 38.35 | .000 | ↑ | 1.36 |
| *N*-palmitoyl methionine | 3.35 | .089 | ↑ | 1.25 |
| *N*-palmitoyl valine | 10.01 | .007 | ↑ | 1.95 |
| 2-linoleoyl glycerol | 20.44 | .000 | ↓ | 0.77 |
| 2-arachidonoyl glycerol | 22.75 | .000 | ↓ | 0.81 |
| Oleic acid | 24.67 | .000 | ↓ | 0.78 |
| Arachidonic acid | 3.31 | .090 | ↓ | 0.83 |
| 6-ketoPGF_1α_ | 10.00 | .007 | ↑ | 1.23 |
| CP 55,940 | 653.09 | .000 | ↑ | infinite |

**Supplemental Table 41.** Lipid levels in the cortex of WT adult female mice treated with Vehicle or 3 mg/kg CP 55,940

|  | Cortex | | | | | | |
| --- | --- | --- | --- | --- | --- | --- | --- |
|  | Vehicle | | | 3mg/kg CP 55,940 | | | |
| ***N*-acyl alanine** | Mean | Std Dev | Std Error | | Mean | Std Dev | Std Error |
| *N*-palmitoyl alanine | 2.75E-12 | 1.46E-12 | 5.18E-13 | | 2.07E-12 | 1.02E-12 | 3.6E-13 |
| *N*-stearoyl alanine | 3.57E-12 | 1.12E-12 | 3.95E-13 | | 3.38E-12 | 1.45E-12 | 5.12E-13 |
| *N*-oleoyl alanine | 2.25E-12 | 6.32E-13 | 2.24E-13 | | 2.22E-12 | 1.33E-12 | 4.69E-13 |
| *N*-linoleoyl alanine | PISSR |  |  | | PISSR |  |  |
| *N*-arachidonoyl alanine | 1.31E-12 | 5.74E-13 | 2.03E-13 | | 1.09E-12 | 4.94E-13 | 1.74E-13 |
| *N*-docosahexaenoyl alanine | PISSR |  |  | | PISSR |  |  |
| ***N*-acyl dopamine** |  |  |  | |  |  |  |
| *N*-oleoyl dopamine | BDL |  |  | | BDL |  |  |
| *N*-arachidonoyl dopamine | BDL |  |  | | BDL |  |  |
| ***N*-acyl ethanolamine** |  |  |  | |  |  |  |
| *N*-palmitoyl ethanolamine | 4.12E-11 | 1.19E-11 | 4.2E-12 | | 3.62E-11 | 7.76E-12 | 2.74E-12 |
| *N*-stearoyl ethanolamine | 3.18E-11 | 2.07E-11 | 7.8E-12 | | 3.18E-11 | 2.4E-11 | 8.5E-12 |
| *N*-oleoyl ethanolamine | 1.68E-10 | 5.29E-11 | 1.87E-11 | | 1.38E-10 | 3.81E-11 | 1.35E-11 |
| *N*-linoleoyl ethanolamine | 3.58E-11 | 9.23E-12 | 3.26E-12 | | 2.93E-11 | 7.1E-12 | 2.51E-12 |
| *N*-arachidonoyl ethanolamine | 3.14E-11 | 7.52E-12 | 2.66E-12 | | 2.77E-11 | 7.34E-12 | 2.6E-12 |
| *N*-docosahexaenoyl ethanolamine | 2.93E-11 | 7.27E-12 | 2.57E-12 | | 2.54E-11 | 6.89E-12 | 2.44E-12 |
| ***N*-acyl GABA** |  |  |  | |  |  |  |
| *N*-palmitoyl GABA | 1.33E-11 | 1.38E-12 | 4.88E-13 | | 1.4E-11 | 2.52E-12 | 8.89E-13 |
| *N*-stearoyl GABA | 1.03E-11 | 1.94E-12 | 6.87E-13 | | 1.05E-11 | 2.11E-12 | 7.45E-13 |
| *N*-oleoyl GABA | 1.09E-11 | 1.13E-12 | 3.99E-13 | | 9.86E-12 | 1.79E-12 | 6.31E-13 |
| *N*-linoleoyl GABA | 4.77E-12 | 6.44E-13 | 2.28E-13 | | 4.08E-12 | 6.77E-13 | 2.39E-13 |
| *N*-arachidonoyl GABA | 1.36E-11 | 1.72E-12 | 6.09E-13 | | 1.28E-11 | 2.21E-12 | 7.8E-13 |
| *N*-docosahexaenoyl GABA | 6.65E-12 | 9.87E-13 | 3.49E-13 | | 6.57E-12 | 1.46E-12 | 5.15E-13 |
| ***N*-acyl glycine** |  |  |  | |  |  |  |
| *N*-palmitoyl glycine | 1.07E-12 | 3.33E-13 | 1.18E-13 | | 9.43E-13 | 2.82E-13 | 9.97E-14 |
| *N*-stearoyl glycine | 1.83E-12 | 9.75E-13 | 3.45E-13 | | 1.95E-12 | 1.26E-12 | 4.45E-13 |
| *N*-oleoyl glycine | 2.77E-13 | 1.49E-13 | 5.27E-14 | | 2.9E-13 | 1.85E-13 | 6.55E-14 |
| *N*-linoleoyl glycine | 4.51E-14 | 4.61E-14 | 1.63E-14 | | 5.03E-14 | 2.9E-14 | 1.02E-14 |
| *N*-arachidonoyl glycine | 9.9E-13 | 7.32E-13 | 2.59E-13 | | 1.07E-12 | 8.26E-13 | 2.92E-13 |
| *N*-docosahexaenoyl glycine | 8.7E-14 | 3.47E-14 | 1.23E-14 | | 8.32E-14 | 6.01E-14 | 2.12E-14 |
| ***N*-acyl leucine** |  |  |  | |  |  |  |
| *N*-palmitoyl leucine | 9.34E-13 | 3.58E-13 | 1.26E-13 | | 1.16E-12 | 1.92E-13 | 6.79E-14 |
| *N*-stearoyl leucine | 1.44E-12 | 2.7E-13 | 9.55E-14 | | 1.32E-12 | 1.33E-13 | 4.72E-14 |
| *N*-oleoyl leucine | 7.93E-13 | 2.61E-13 | 9.21E-14 | | 7.57E-13 | 1.05E-13 | 3.7E-14 |
| *N*-linoleoyl leucine | 1.01E-13 | 2.75E-14 | 9.74E-15 | | 9.31E-14 | 3.64E-14 | 1.29E-14 |
| *N*-docosahexaenoyl leucine | 7.45E-13 | 1.05E-13 | 3.71E-14 | | 8.03E-13 | 2.22E-13 | 7.83E-14 |
| ***N*-acyl methionine** |  |  |  | |  |  |  |
| *N*-palmitoyl methionine | 2.96E-12 | 5.39E-13 | 1.91E-13 | | 2.58E-12 | 4.98E-13 | 1.76E-13 |
| *N*-stearoyl methionine | 4.59E-13 | 1.82E-13 | 6.43E-14 | | 3.92E-13 | 1.2E-13 | 4.24E-14 |
| *N*-oleoyl methionine | PISSR |  |  | | PISSR |  |  |
| *N*-linoleoyl methionine | BDL |  |  | | BDL |  |  |
| *N*-arachidonoyl methionine | PISSR |  |  | | PISSR |  |  |
| *N*-docosahexaenoyl methionine | BDL |  |  | | BDL |  |  |
| ***N*-acyl phenylalanine** |  |  |  | |  |  |  |
| *N*-palmitoyl phenylalanine | 2.42E-13 | 1.32E-13 | 4.66E-14 | | 2.56E-13 | 5.79E-14 | 2.05E-14 |
| *N*-stearoyl phenylalanine | 2.59E-13 | 1.04E-13 | 3.67E-14 | | 1.71E-13 | 5.79E-14 | 2.05E-14 |
| *N*-oleoyl phenylalanine | 3.88E-13 | 1.82E-13 | 6.42E-14 | | 3.2E-13 | 4.21E-14 | 1.49E-14 |
| *N*-linoleoyl phenylalanine | PISSR |  |  | | PISSR |  |  |
| *N*-arachidonoyl phenylalanine | 9.4E-14 | 5.17E-14 | 1.83E-14 | | 5.77E-14 | 4.73E-14 | 1.67E-14 |
| *N*-docosahexaenoyl phenylalanine | 3.12E-14 | 1.24E-14 | 4.39E-15 | | 3E-14 | 1.56E-14 | 5.53E-15 |

**Supplemental Table 41: Continued**

|  | Cortex | | | | | |
| --- | --- | --- | --- | --- | --- | --- |
|  | Vehicle | | | 3mg/kg CP 55,940 | | |
| ***N*-acyl proline** | Mean | Std Dev | Std Error | Mean | Std Dev | Std Error |
| *N*-palmitoyl proline | BDL |  |  | BDL |  |  |
| *N*-stearoyl proline | BDL |  |  | BDL |  |  |
| *N-*oleoyl proline | BDL |  |  | BDL |  |  |
| *N*-linoleoyl proline | BDL |  |  | BDL |  |  |
| *N*-arachidonoyl proline | BDL |  |  | BDL |  |  |
| *N*-docosahexaenoyl proline | BDL |  |  | BDL |  |  |
| ***N*-acyl serine** |  |  |  |  |  |  |
| *N*-palmitoyl serine | 7.22E-12 | 2.6E-12 | 9.2E-13 | 6.45E-12 | 2.16E-12 | 7.64E-13 |
| *N*-stearoyl serine | 9.81E-12 | 1.8E-12 | 6.35E-13 | 9.84E-12 | 1.17E-12 | 4.14E-13 |
| *N*-oleoyl serine | 8.99E-11 | 1.56E-11 | 5.52E-12 | 8.25E-11 | 1.95E-11 | 6.91E-12 |
| *N*-linoleoyl serine | 1.67E-11 | 3.09E-12 | 1.09E-12 | 1.53E-11 | 4.34E-12 | 1.53E-12 |
| *N*-arachidonoyl serine | PISSR |  |  | PISSR |  |  |
| *N*-docosahexaenoyl serine | PISSR |  |  | PISSR |  |  |
| ***N*-acyl taurine** |  |  |  |  |  |  |
| *N*-arachidonoyl taurine | 9.66E-11 | 8.77E-12 | 3.1E-12 | 8.95E-11 | 6.3E-12 | 2.23E-12 |
| ***N*-acyl tryptophan** |  |  |  |  |  |  |
| *N*-palmitoyl tryptophan | BDL |  |  | BDL |  |  |
| *N*-stearoyl tryptophan | BDL |  |  | BDL |  |  |
| *N*-oleoyl tryptophan | BDL |  |  | BDL |  |  |
| *N*-linoleoyl tryptophan | BDL |  |  | BDL |  |  |
| *N*-arachidonoyl tryptophan | BDL |  |  | BDL |  |  |
| *N*-docosahexaenoyl tryptophan | BDL |  |  | BDL |  |  |
| ***N*-acyl tyrosine** |  |  |  |  |  |  |
| *N*-palmitoyl tyrosine | 9.06E-13 | 1.96E-13 | 6.93E-14 | 7.95E-13 | 1.64E-13 | 5.81E-14 |
| *N*-stearoyl tyrosine | 1.53E-13 | 6.5E-14 | 2.3E-14 | 1.21E-13 | 4.93E-14 | 1.74E-14 |
| *N-*oleoyl tyrosine | 4E-13 | 1.78E-13 | 6.29E-14 | 3.46E-13 | 1.33E-13 | 4.71E-14 |
| *N*-linoleoyl tyrosine | BDL |  |  | BDL |  |  |
| *N*-arachidonoyl tyrosine | 7.87E-13 | 2.46E-13 | 8.68E-14 | 6.06E-13 | 2.42E-13 | 8.57E-14 |
| *N*-docosahexaenoyl tyrosine | 4.4E-13 | 1.02E-13 | 3.61E-14 | 3.81E-13 | 1.14E-13 | 4.04E-14 |
| ***N*-acyl valine** |  |  |  |  |  |  |
| *N*-palmitoyl valine | 7.58E-14 | 2.04E-14 | 7.22E-15 | 1.13E-13 | 2.69E-14 | 9.52E-15 |
| *N*- stearoyl valine | 2.69E-14 | 1.23E-14 | 4.35E-15 | 2.51E-14 | 1.56E-14 | 5.52E-15 |
| *N*-oleoyl valine | 9.09E-15 | 3.64E-15 | 1.29E-15 | 1.3E-14 | 7.59E-15 | 2.68E-15 |
| *N*-nervonoyl valine | BDL |  |  | BDL |  |  |
| *N*-linoleoyl valine | PISSR |  |  | PISSR |  |  |
| *N*-docosahexaenoyl valine | PISSR |  |  | PISSR |  |  |
| **2-acyl glycerols** |  |  |  |  |  |  |
| 2-palmitoyl glycerol | 1.02E-10 | 3.25E-11 | 1.15E-11 | 8.13E-11 | 2.33E-11 | 8.23E-12 |
| 2-oleoyl glycerol | 2.05E-09 | 9.67E-10 | 3.42E-10 | 1.56E-09 | 4.71E-10 | 1.67E-10 |
| 2-linoleoyl glycerol | 1.95E-10 | 6.21E-11 | 2.2E-11 | 1.48E-10 | 3.78E-11 | 1.33E-11 |
| 2-arachidonoyl glycerol | 2.99E-09 | 1.27E-09 | 4.48E-10 | 3.28E-09 | 4.59E-10 | 1.62E-10 |
| **Free Fatty Acids** |  |  |  |  |  |  |
| Oleic acid | 4.92E-10 | 9.61E-11 | 3.4E-11 | 4.6E-10 | 1.19E-10 | 4.21E-11 |
| Linoleic acid | 6.26E-10 | 1.79E-10 | 6.33E-11 | 5.34E-10 | 1.16E-10 | 4.09E-11 |
| Arachidonic acid | 1.05E-09 | 1.7E-10 | 6E-11 | 9.03E-10 | 1.35E-10 | 4.76E-11 |
| **PhosphoLEA** |  |  |  |  |  |  |
| PhosphoLEA | 2.06E-12 | 3.56E-13 | 1.26E-13 | 1.8E-12 | 4.87E-13 | 1.72E-13 |
| **Prostaglandins** |  |  |  |  |  |  |
| PGE_2_ | 3.5E-10 | 5.45E-11 | 1.93E-11 | 3.08E-10 | 4.68E-11 | 1.65E-11 |
| PGF_2α_ | 3.11E-10 | 2.46E-11 | 8.68E-12 | 2.77E-10 | 3.12E-11 | 1.1E-11 |
| 6-ketoPGF_1α_ | 2.21E-11 | 4.28E-12 | 1.51E-12 | 1.5E-11 | 4.11E-12 | 1.45E-12 |
| **THC/CP/CBD** |  |  |  |  |  |  |
| Cannabidiol | BDL |  |  | BDL |  |  |
| CP 55,940 | BDL |  |  | 1.85E-10 | 2.51E-11 | 8.86E-12 |
| THC | BDL |  |  | BDL |  |  |
| **THC Metabolites** |  |  |  |  |  |  |
| 11-nor-9-carboxyTHC | BDL |  |  | BDL |  |  |
| 11-OH-THC | BDL |  |  | BDL |  |  |

**Supplemental Table 42.** List of lipids in the cortex significantly affected by 3 mg/kg CP 55,940: Adult

| Adult Cortex Significant Differences in One-Way ANOVA | | | | |
| --- | --- | --- | --- | --- |
| Lipid | F | p | Direction (relative to Veh) | Magnitude (x Veh level) |
| *N*-linoleoyl GABA | 4.37 | .055 | ↓ | 0.86 |
| *N*-stearoyl phenylalanine | 4.32 | .057 | ↓ | 0.66 |
| *N*-arachidonoyl taurine | 3.40 | .086 | ↓ | 0.93 |
| *N*-palmitoyl valine | 9.47 | .008 | ↑ | 1.49 |
| 2-linoleoyl glycerol | 3.32 | .090 | ↓ | 0.76 |
| Arachidonic acid | 3.65 | .077 | ↓ | 0.86 |
| PGF_2α_ | 5.99 | .028 | ↓ | 0.89 |
| 6-ketoPGF_1α_ | 11.50 | .004 | ↓ | 0.68 |
| CP 55,940 | 436.25 | .000 | ↑ | infinite |

**Supplemental Table 43.** Lipid levels in the hypothalamus of WT adult female mice treated with Vehicle or 3 mg/kg CP 55,940

|  | Hypothalamus | | | | | | |
| --- | --- | --- | --- | --- | --- | --- | --- |
|  | Vehicle | | | 3mg/kg CP 55,940 | | | |
| ***N*-acyl alanine** | Mean | Std Dev | Std Error | | Mean | Std Dev | Std Error |
| *N*-palmitoyl alanine | 3.1E-12 | 6.09E-13 | 2.15E-13 | | 2.7E-12 | 1.03E-12 | 3.63E-13 |
| *N*-stearoyl alanine | 3.33E-12 | 1.01E-12 | 3.58E-13 | | 2.77E-12 | 7.7E-13 | 2.72E-13 |
| *N*-oleoyl alanine | 4.88E-12 | 1.09E-12 | 3.86E-13 | | 4.79E-12 | 1.99E-12 | 7.02E-13 |
| *N*-linoleoyl alanine | PISSR |  |  | | PISSR |  |  |
| *N*-arachidonoyl alanine | PISSR |  |  | | PISSR |  |  |
| *N*-docosahexaenoyl alanine | PISSR |  |  | | PISSR |  |  |
| ***N*-acyl dopamine** |  |  |  | |  |  |  |
| *N*-oleoyl dopamine | BDL |  |  | | BDL |  |  |
| *N*-arachidonoyl dopamine | BDL |  |  | | BDL |  |  |
| ***N*-acyl ethanolamine** |  |  |  | |  |  |  |
| *N*-palmitoyl ethanolamine | 2.24E-11 | 1.01E-11 | 3.59E-12 | | 2.56E-11 | 1.06E-11 | 3.73E-12 |
| *N*-stearoyl ethanolamine | 7.76E-12 | 1.28E-11 | 4.53E-12 | | 7.07E-12 | 5.68E-12 | 2.01E-12 |
| *N*-oleoyl ethanolamine | 6.73E-11 | 6.47E-11 | 2.29E-11 | | 7.29E-11 | 4.36E-11 | 1.54E-11 |
| *N*-linoleoyl ethanolamine | 5.77E-12 | 2.36E-12 | 8.35E-13 | | 6.58E-12 | 2.39E-12 | 8.44E-13 |
| *N*-arachidonoyl ethanolamine | 6.23E-12 | 2.1E-12 | 7.44E-13 | | 6.61E-12 | 2.56E-12 | 9.04E-13 |
| *N*-docosahexaenoyl ethanolamine | 1.05E-11 | 3.4E-12 | 1.2E-12 | | 1.18E-11 | 4.66E-12 | 1.65E-12 |
| ***N*-acyl GABA** |  |  |  | |  |  |  |
| *N*-palmitoyl GABA | 2.85E-11 | 1.7E-12 | 6.01E-13 | | 3.12E-11 | 4.77E-12 | 1.69E-12 |
| *N*-stearoyl GABA | PISSR |  |  | | PISSR |  |  |
| *N*-oleoyl GABA | PISSR |  |  | | PISSR |  |  |
| *N*-linoleoyl GABA | BDL |  |  | | BDL |  |  |
| *N*-arachidonoyl GABA | 2.47E-11 | 4.48E-12 | 1.58E-12 | | 2.05E-11 | 4.21E-12 | 1.49E-12 |
| *N*-docosahexaenoyl GABA | PISSR |  |  | | PISSR |  |  |
| ***N*-acyl glycine** |  |  |  | |  |  |  |
| *N*-palmitoyl glycine | 2.37E-12 | 1.04E-12 | 3.67E-13 | | 2.34E-12 | 6.24E-13 | 2.21E-13 |
| *N*-stearoyl glycine | 3.16E-12 | 2.1E-12 | 7.41E-13 | | 2.2E-12 | 9.66E-13 | 3.42E-13 |
| *N*-oleoyl glycine | 2.32E-13 | 1.2E-13 | 4.23E-14 | | 2.66E-13 | 1.88E-13 | 6.63E-14 |
| *N*-linoleoyl glycine | PISSR |  |  | | PISSR |  |  |
| *N*-arachidonoyl glycine | 3.49E-13 | 3.05E-13 | 1.08E-13 | | 2.96E-13 | 1.8E-13 | 6.35E-14 |
| *N*-docosahexaenoyl glycine | PISSR |  |  | | PISSR |  |  |
| ***N*-acyl leucine** |  |  |  | |  |  |  |
| *N*-palmitoyl leucine | 1.83E-12 | 3.35E-13 | 1.18E-13 | | 1.88E-12 | 5.36E-13 | 1.89E-13 |
| *N*-stearoyl leucine | 1.81E-12 | 3.83E-13 | 1.35E-13 | | 1.65E-12 | 5.42E-13 | 1.92E-13 |
| *N*-oleoyl leucine | PISSR |  |  | | PISSR |  |  |
| *N*-linoleoyl leucine | PISSR |  |  | | PISSR |  |  |
| *N*-docosahexaenoyl leucine | PISSR |  |  | | PISSR |  |  |
| ***N*-acyl methionine** |  |  |  | |  |  |  |
| *N*-palmitoyl methionine | BDL |  |  | | BDL |  |  |
| *N*-stearoyl methionine | BDL |  |  | | BDL |  |  |
| *N*-oleoyl methionine | BDL |  |  | | BDL |  |  |
| *N*-linoleoyl methionine | BDL |  |  | | BDL |  |  |
| *N*-arachidonoyl methionine | BDL |  |  | | BDL |  |  |
| *N*-docosahexaenoyl methionine | BDL |  |  | | BDL |  |  |
| ***N*-acyl phenylalanine** |  |  |  | |  |  |  |
| *N*-palmitoyl phenylalanine | 1.84E-13 | 1.04E-13 | 3.67E-14 | | 3.42E-13 | 1.36E-13 | 4.8E-14 |
| *N*-stearoyl phenylalanine | 2.78E-13 | 1.08E-13 | 3.8E-14 | | 2.86E-13 | 1.19E-13 | 4.21E-14 |
| *N*-oleoyl phenylalanine | 4.44E-13 | 2.46E-13 | 8.69E-14 | | 5E-13 | 2.1E-13 | 7.44E-14 |
| *N*-linoleoyl phenylalanine | PISSR |  |  | | PISSR |  |  |
| *N*-arachidonoyl phenylalanine | 1E-13 | 6.57E-14 | 2.32E-14 | | 1.34E-13 | 6.65E-14 | 2.35E-14 |
| *N*-docosahexaenoyl phenylalanine | PISSR |  |  | | PISSR |  |  |

**Supplemental Table 43: Continued**

|  | Hypothalamus | | | | | |
| --- | --- | --- | --- | --- | --- | --- |
|  | Vehicle | | | CP 55,940 | | |
| ***N*-acyl proline** | Mean | Std Dev | Std Error | Mean | Std Dev | Std Error |
| *N*-palmitoyl proline | BDL |  |  | BDL |  |  |
| *N*-stearoyl proline | BDL |  |  | BDL |  |  |
| *N-*oleoyl proline | BDL |  |  | BDL |  |  |
| *N*-linoleoyl proline | BDL |  |  | BDL |  |  |
| *N*-arachidonoyl proline | BDL |  |  | BDL |  |  |
| *N*-docosahexaenoyl proline | BDL |  |  | BDL |  |  |
| ***N*-acyl serine** |  |  |  |  |  |  |
| *N*-palmitoyl serine | PISSR |  |  | PISSR |  |  |
| *N*-stearoyl serine | 5.33E-11 | 1.28E-11 | 4.51E-12 | 4.88E-11 | 1.06E-11 | 3.74E-12 |
| *N*-oleoyl serine | 5.85E-10 | 5.37E-11 | 1.9E-11 | 5.82E-10 | 8.82E-11 | 3.12E-11 |
| *N*-linoleoyl serine | 1.15E-10 | 1.23E-11 | 4.36E-12 | 1.16E-10 | 2.13E-11 | 7.52E-12 |
| *N*-arachidonoyl serine | BDL |  |  | BDL |  |  |
| *N*-docosahexaenoyl serine | PISSR |  |  | PISSR |  |  |
| ***N*-acyl taurine** |  |  |  |  |  |  |
| *N*-arachidonoyl taurine | 1.53E-11 | 2.74E-12 | 9.68E-13 | 1.91E-11 | 4.4E-12 | 1.55E-12 |
| ***N*-acyl tryptophan** |  |  |  |  |  |  |
| *N*-palmitoyl tryptophan | BDL |  |  | BDL |  |  |
| *N*-stearoyl tryptophan | BDL |  |  | BDL |  |  |
| *N*-oleoyl tryptophan | BDL |  |  | BDL |  |  |
| *N*-linoleoyl tryptophan | BDL |  |  | BDL |  |  |
| *N*-arachidonoyl tryptophan | BDL |  |  | BDL |  |  |
| *N*-docosahexaenoyl tryptophan | BDL |  |  | BDL |  |  |
| ***N*-acyl tyrosine** |  |  |  |  |  |  |
| *N*-palmitoyl tyrosine | 1.36E-12 | 2.9E-13 | 1.02E-13 | 1.11E-12 | 3.71E-13 | 1.31E-13 |
| *N*-stearoyl tyrosine | PISSR |  |  | PISSR |  |  |
| *N-*oleoyl tyrosine | PISSR |  |  | PISSR |  |  |
| *N*-linoleoyl tyrosine | BDL |  |  | BDL |  |  |
| *N*-arachidonoyl tyrosine | PISSR |  |  | PISSR |  |  |
| *N*-docosahexaenoyl tyrosine | PISSR |  |  | PISSR |  |  |
| ***N*-acyl valine** |  |  |  |  |  |  |
| *N*-palmitoyl valine | 1.75E-13 | 1.2E-13 | 4.25E-14 | 1.56E-13 | 9.46E-14 | 3.34E-14 |
| *N*- stearoyl valine | PISSR |  |  | PISSR |  |  |
| *N*-oleoyl valine | PISSR |  |  | PISSR |  |  |
| *N*-nervonoyl valine | BDL |  |  | BDL |  |  |
| *N*-linoleoyl valine | BDL |  |  | BDL |  |  |
| *N*-docosahexaenoyl valine | BDL |  |  | BDL |  |  |
| **2-acyl glycerols** |  |  |  |  |  |  |
| 2-palmitoyl glycerol | 2.01E-10 | 1.18E-10 | 4.17E-11 | 1.78E-10 | 4.13E-11 | 1.46E-11 |
| 2-oleoyl glycerol | 1.49E-09 | 1.12E-09 | 3.94E-10 | 1.71E-09 | 6.91E-10 | 2.44E-10 |
| 2-linoleoyl glycerol | 1.71E-10 | 7.98E-11 | 2.82E-11 | 1.7E-10 | 5.99E-11 | 2.12E-11 |
| 2-arachidonoyl glycerol | 2.72E-09 | 9.24E-10 | 3.27E-10 | 2.11E-09 | 6.45E-10 | 2.28E-10 |
| **Free Fatty Acids** |  |  |  |  |  |  |
| Oleic acid | 1.91E-10 | 3.61E-11 | 1.28E-11 | 2.14E-10 | 8.72E-11 | 3.08E-11 |
| Linoleic acid | 1.18E-10 | 2.69E-11 | 9.5E-12 | 1.2E-10 | 4.16E-11 | 1.47E-11 |
| Arachidonic acid | 6.81E-10 | 1.07E-10 | 3.78E-11 | 6.14E-10 | 9.26E-11 | 3.28E-11 |
| **PhosphoLEA** |  |  |  |  |  |  |
| PhosphoLEA | 7.78E-11 | 1.27E-11 | 4.49E-12 | 5.73E-11 | 8.8E-12 | 3.11E-12 |
| **Prostaglandins** |  |  |  |  |  |  |
| PGE_2_ | 1.91E-10 | 2.58E-11 | 9.13E-12 | 2.25E-10 | 3.31E-11 | 1.17E-11 |
| PGF_2α_ | 2.76E-10 | 2.73E-11 | 9.65E-12 | 2.18E-10 | 2.46E-11 | 8.7E-12 |
| 6-ketoPGF_1α_ | 3.5E-11 | 1.08E-11 | 3.8E-12 | 2.15E-11 | 4.43E-12 | 1.57E-12 |
| **THC/CP/CBD** |  |  |  |  |  |  |
| Cannabidiol | BDL |  |  | BDL |  |  |
| CP 55,940 | BDL |  |  | 1.17E-10 | 2.16E-11 | 7.64E-12 |
| THC | BDL |  |  | BDL |  |  |
| **THC Metabolites** |  |  |  |  |  |  |
| 11-nor-9-carboxyTHC | BDL |  |  | BDL |  |  |
| 11-OH-THC | BDL |  |  | BDL |  |  |

**Supplemental Table 44.** List of lipids in the hypothalamus significantly affected by 3mg/kg CP 55,940: Adult

| Adult Hypothalamus Significant Differences in One-Way ANOVA | | | | |
| --- | --- | --- | --- | --- |
| Lipid | F | p | Direction (relative to Veh) | Magnitude (x Veh level) |
| *N*-arachidonoyl GABA | 3.76 | .073 | ↓ | 0.83 |
| *N*-palmitoyl phenylalanine | 6.83 | .020 | ↑ | 1.86 |
| *N*-arachidonoyl taurine | 4.17 | .060 | ↑ | 1.24 |
| phosphoLEA | 14.06 | .002 | ↓ | 0.74 |
| PGE_2_ | 5.38 | .036 | ↑ | 1.18 |
| PGF_2α_ | 20.07 | .001 | ↓ | 0.79 |
| 6-ketoPGF_1α_ | 10.81 | .005 | ↓ | 0.61 |
| CP 55,940 | 235.52 | .000 | ↑ | infinite |
| Sample Mass | 5.80 | .030 | ↑ | 1.10 |

**Supplemental Table 45.** Lipid levels in the midbrain of adult WT adult female mice treated with Vehicle or 3 mg/kg CP 55,940

|  | Midbrain | | | | | | |
| --- | --- | --- | --- | --- | --- | --- | --- |
|  | Vehicle | | | 3mg/kg CP 55,940 | | | |
| ***N*-acyl alanine** | Mean | Std Dev | Std Error | | Mean | Std Dev | Std Error |
| *N*-palmitoyl alanine | 2.07E-12 | 8.72E-13 | 3.08E-13 | | 1.61E-12 | 1.07E-12 | 3.79E-13 |
| *N*-stearoyl alanine | 2.38E-12 | 9.24E-13 | 3.27E-13 | | 2.08E-12 | 7.21E-13 | 2.55E-13 |
| *N*-oleoyl alanine | 2.74E-12 | 8.73E-13 | 3.09E-13 | | 2.56E-12 | 1.1E-12 | 3.89E-13 |
| *N*-linoleoyl alanine | 5.04E-13 | 3.29E-13 | 1.16E-13 | | 4.8E-13 | 3.12E-13 | 1.1E-13 |
| *N*-arachidonoyl alanine | 1.02E-12 | 4.61E-13 | 1.63E-13 | | 8.5E-13 | 5.38E-13 | 1.9E-13 |
| *N*-docosahexaenoyl alanine | PISSR |  |  | | PISSR |  |  |
| ***N*-acyl dopamine** |  |  |  | |  |  |  |
| *N*-oleoyl dopamine | BDL |  |  | | BDL |  |  |
| *N*-arachidonoyl dopamine | BDL |  |  | | BDL |  |  |
| ***N*-acyl ethanolamine** |  |  |  | |  |  |  |
| *N*-palmitoyl ethanolamine | 8.44E-11 | 1.48E-11 | 5.24E-12 | | 6.9E-11 | 8.53E-12 | 3.02E-12 |
| *N*-stearoyl ethanolamine | 9.34E-12 | 1.51E-12 | 5.72E-13 | | 1.35E-11 | 5.42E-12 | 1.91E-12 |
| *N*-oleoyl ethanolamine | 3.73E-10 | 1.37E-10 | 5.16E-11 | | 3.41E-10 | 1.2E-10 | 4.24E-11 |
| *N*-linoleoyl ethanolamine | 1.82E-11 | 7.8E-12 | 2.76E-12 | | 1.5E-11 | 6.24E-12 | 2.2E-12 |
| *N*-arachidonoyl ethanolamine | 1.81E-11 | 4.24E-12 | 1.5E-12 | | 1.42E-11 | 3.32E-12 | 1.17E-12 |
| *N*-docosahexaenoyl ethanolamine | 3.88E-11 | 1.23E-11 | 4.35E-12 | | 3.34E-11 | 9.41E-12 | 3.33E-12 |
| ***N*-acyl GABA** |  |  |  | |  |  |  |
| *N*-palmitoyl GABA | 2.88E-11 | 4.9E-12 | 1.73E-12 | | 2.91E-11 | 6.06E-12 | 2.14E-12 |
| *N*-stearoyl GABA | 1.87E-11 | 3.85E-12 | 1.36E-12 | | 1.9E-11 | 3.47E-12 | 1.23E-12 |
| *N*-oleoyl GABA | 1.75E-11 | 2.57E-12 | 9.1E-13 | | 1.69E-11 | 2.37E-12 | 8.39E-13 |
| *N*-linoleoyl GABA | PISSR |  |  | | PISSR |  |  |
| *N*-arachidonoyl GABA | 2.22E-11 | 3.28E-12 | 1.16E-12 | | 2.05E-11 | 3.96E-12 | 1.4E-12 |
| *N*-docosahexaenoyl GABA | 9.76E-12 | 1.94E-12 | 6.86E-13 | | 8.66E-12 | 2.04E-12 | 7.22E-13 |
| ***N*-acyl glycine** |  |  |  | |  |  |  |
| *N*-palmitoyl glycine | 1.85E-12 | 8.56E-13 | 3.03E-13 | | 1.85E-12 | 1.2E-12 | 4.23E-13 |
| *N*-stearoyl glycine | 2.73E-12 | 1.86E-12 | 6.59E-13 | | 2.96E-12 | 2.37E-12 | 8.38E-13 |
| *N*-oleoyl glycine | 5.9E-13 | 4.15E-13 | 1.47E-13 | | 4.95E-13 | 5.36E-13 | 1.89E-13 |
| *N*-linoleoyl glycine | 8.66E-14 | 4.58E-14 | 1.62E-14 | | 4.47E-14 | 3.65E-14 | 1.29E-14 |
| *N*-arachidonoyl glycine | 1.03E-12 | 5.65E-13 | 2E-13 | | 7.94E-13 | 5.42E-13 | 1.92E-13 |
| *N*-docosahexaenoyl glycine | 1.51E-13 | 9.25E-14 | 3.27E-14 | | 1.02E-13 | 6.56E-14 | 2.32E-14 |
| ***N*-acyl leucine** |  |  |  | |  |  |  |
| *N*-palmitoyl leucine | 1.99E-12 | 1.43E-13 | 5.06E-14 | | 2.19E-12 | 3.08E-13 | 1.09E-13 |
| *N*-stearoyl leucine | 1.14E-12 | 3.03E-13 | 1.07E-13 | | 1.09E-12 | 2.13E-13 | 7.52E-14 |
| *N*-oleoyl leucine | 1.03E-12 | 3.26E-13 | 1.15E-13 | | 1.1E-12 | 3.96E-13 | 1.4E-13 |
| *N*-linoleoyl leucine | PISSR |  |  | | PISSR |  |  |
| *N*-docosahexaenoyl leucine | PISSR |  |  | | PISSR |  |  |
| ***N*-acyl methionine** |  |  |  | |  |  |  |
| *N*-palmitoyl methionine | 3.24E-12 | 6.32E-13 | 2.23E-13 | | 2.91E-12 | 7.78E-13 | 2.75E-13 |
| *N*-stearoyl methionine | 4.41E-13 | 1.56E-13 | 5.51E-14 | | 4.14E-13 | 1.99E-13 | 7.04E-14 |
| *N*-oleoyl methionine | PISSR |  |  | | PISSR |  |  |
| *N*-linoleoyl methionine | BDL |  |  | | BDL |  |  |
| *N*-arachidonoyl methionine | PISSR |  |  | | PISSR |  |  |
| *N*-docosahexaenoyl methionine | BDL |  |  | | BDL |  |  |
| ***N*-acyl phenylalanine** |  |  |  | |  |  |  |
| *N*-palmitoyl phenylalanine | 1.32E-12 | 6.1E-13 | 2.16E-13 | | 1.17E-12 | 6.45E-13 | 2.28E-13 |
| *N*-stearoyl phenylalanine | 9.84E-13 | 5.46E-13 | 1.93E-13 | | 7.89E-13 | 3.14E-13 | 1.11E-13 |
| *N*-oleoyl phenylalanine | 1.49E-12 | 4.06E-13 | 1.43E-13 | | 1.49E-12 | 5.73E-13 | 2.02E-13 |
| *N*-linoleoyl phenylalanine | PISSR |  |  | | PISSR |  |  |
| *N*-arachidonoyl phenylalanine | 3.8E-13 | 3.19E-13 | 1.13E-13 | | 2.39E-13 | 1.58E-13 | 5.57E-14 |
| *N*-docosahexaenoyl phenylalanine | 1.47E-13 | 1.02E-13 | 3.6E-14 | | 1.11E-13 | 6.81E-14 | 2.41E-14 |

**Supplemental Table 45: Continued**

|  | Midbrain | | | | | |
| --- | --- | --- | --- | --- | --- | --- |
|  | Vehicle | | | 3mg/kg CP 55,940 | | |
| ***N*-acyl proline** | Mean | Std Dev | Std Error | Mean | Std Dev | Std Error |
| *N*-palmitoyl proline | BDL |  |  | BDL |  |  |
| *N*-stearoyl proline | BDL |  |  | BDL |  |  |
| *N-*oleoyl proline | BDL |  |  | BDL |  |  |
| *N*-linoleoyl proline | BDL |  |  | BDL |  |  |
| *N*-arachidonoyl proline | BDL |  |  | BDL |  |  |
| *N*-docosahexaenoyl proline | BDL |  |  | BDL |  |  |
| ***N*-acyl serine** |  |  |  |  |  |  |
| *N*-palmitoyl serine | PISSR |  |  | PISSR |  |  |
| *N*-stearoyl serine | 1.72E-11 | 2.9E-12 | 1.03E-12 | 1.92E-11 | 2.68E-12 | 9.47E-13 |
| *N*-oleoyl serine | 1.49E-10 | 1.33E-11 | 4.72E-12 | 1.28E-10 | 3.44E-11 | 1.22E-11 |
| *N*-linoleoyl serine | 2.64E-11 | 4.56E-12 | 1.61E-12 | 2.09E-11 | 7.11E-12 | 2.51E-12 |
| *N*-arachidonoyl serine | 8.17E-13 | 5.34E-13 | 1.89E-13 | 4.34E-13 | 2.98E-13 | 1.05E-13 |
| *N*-docosahexaenoyl serine | PISSR |  |  | PISSR |  |  |
| ***N*-acyl taurine** |  |  |  |  |  |  |
| *N*-arachidonoyl taurine | 5.73E-11 | 6.51E-12 | 2.3E-12 | 5.5E-11 | 4.1E-12 | 1.45E-12 |
| ***N*-acyl tryptophan** |  |  |  |  |  |  |
| *N*-palmitoyl tryptophan | BDL |  |  | BDL |  |  |
| *N*-stearoyl tryptophan | BDL |  |  | BDL |  |  |
| *N*-oleoyl tryptophan | BDL |  |  | BDL |  |  |
| *N*-linoleoyl tryptophan | BDL |  |  | BDL |  |  |
| *N*-arachidonoyl tryptophan | BDL |  |  | BDL |  |  |
| *N*-docosahexaenoyl tryptophan | BDL |  |  | BDL |  |  |
| ***N*-acyl tyrosine** |  |  |  |  |  |  |
| *N*-palmitoyl tyrosine | 1.23E-12 | 4.96E-13 | 1.75E-13 | 1.01E-12 | 1.61E-13 | 5.7E-14 |
| *N*-stearoyl tyrosine | PISSR |  |  | PISSR |  |  |
| *N-*oleoyl tyrosine | 7.4E-13 | 2.84E-13 | 1E-13 | 7.3E-13 | 3.9E-13 | 1.38E-13 |
| *N*-linoleoyl tyrosine | BDL |  |  | BDL |  |  |
| *N*-arachidonoyl tyrosine | 1.31E-12 | 6.46E-13 | 2.28E-13 | 1.09E-12 | 5.31E-13 | 1.88E-13 |
| *N*-docosahexaenoyl tyrosine | PISSR |  |  | PISSR |  |  |
| ***N*-acyl valine** |  |  |  |  |  |  |
| *N*-palmitoyl valine | 1.78E-13 | 7.46E-14 | 2.64E-14 | 1.76E-13 | 8.15E-14 | 2.88E-14 |
| *N*- stearoyl valine | 3.3E-14 | 1.55E-14 | 5.48E-15 | 3.08E-14 | 2.07E-14 | 7.32E-15 |
| *N*-oleoyl valine | 1.61E-14 | 1.36E-14 | 4.82E-15 | 2.38E-14 | 1.09E-14 | 3.85E-15 |
| *N*-nervonoyl valine | BDL |  |  | BDL |  |  |
| *N*-linoleoyl valine | PISSR |  |  | PISSR |  |  |
| *N*-docosahexaenoyl valine | PISSR |  |  | PISSR |  |  |
| **2-acyl glycerols** |  |  |  |  |  |  |
| 2-palmitoyl glycerol | 1.02E-10 | 3.04E-11 | 1.07E-11 | 8.64E-11 | 1.82E-11 | 6.45E-12 |
| 2-oleoyl glycerol | 5.85E-09 | 4.92E-10 | 1.74E-10 | 4.34E-09 | 1.99E-10 | 7.04E-11 |
| 2-linoleoyl glycerol | 4.24E-10 | 5.91E-11 | 2.09E-11 | 3.28E-10 | 4.72E-11 | 1.67E-11 |
| 2-arachidonoyl glycerol | 4.32E-09 | 4.62E-10 | 1.63E-10 | 3.47E-09 | 2.08E-10 | 7.36E-11 |
| **Free Fatty Acids** |  |  |  |  |  |  |
| Oleic acid | 5.39E-10 | 1.4E-10 | 4.94E-11 | 4.73E-10 | 6.95E-11 | 2.46E-11 |
| Linoleic acid | 2.76E-10 | 7.07E-11 | 2.5E-11 | 2.55E-10 | 3.71E-11 | 1.31E-11 |
| Arachidonic acid | 6.91E-10 | 4.28E-11 | 1.51E-11 | 5.39E-10 | 1.93E-11 | 6.82E-12 |
| **PhosphoLEA** |  |  |  |  |  |  |
| PhosphoLEA | 3.38E-11 | 6.57E-12 | 2.32E-12 | 2.38E-11 | 4.25E-12 | 1.5E-12 |
| **Prostaglandins** |  |  |  |  |  |  |
| PGE_2_ | 2.04E-10 | 2.41E-11 | 8.51E-12 | 2.39E-10 | 3.89E-11 | 1.37E-11 |
| PGF_2α_ | 2.85E-10 | 3.29E-11 | 1.16E-11 | 2.91E-10 | 4.06E-11 | 1.43E-11 |
| 6-ketoPGF_1α_ | 2.55E-11 | 4.67E-12 | 1.65E-12 | 3.28E-11 | 8.64E-12 | 3.05E-12 |
| **THC/CP/CBD** |  |  |  |  |  |  |
| Cannabidiol | BDL |  |  | BDL |  |  |
| CP 55,940 | BDL |  |  | 2.2E-10 | 2.28E-11 | 8.05E-12 |
| THC | BDL |  |  | BDL |  |  |
| **THC Metabolites** |  |  |  |  |  |  |
| 11-nor-9-carboxyTHC | BDL |  |  | BDL |  |  |
| 11-OH-THC | BDL |  |  | BDL |  |  |

**Supplemental Table 46.** List of lipids in the midbrain significantly affected by 3mg/kg CP 55,940: Adult

| Adult Midbrain Significant Differences in One-Way ANOVA | | | | |
| --- | --- | --- | --- | --- |
| Lipid | F | p | Direction (relative to Veh) | Magnitude (x Veh level) |
| *N*-palmitoyl ethanolamine | 6.48 | .023 | ↓ | 0.82 |
| *N*-stearoyl ethanolamine | 3.80 | .073 | ↑ | 1.44 |
| *N*-arachidonoyl ethanolamine | 4.19 | .060 | ↓ | 0.78 |
| *N*-linoleoyl glycine | 4.09 | .063 | ↓ | 0.52 |
| *N*-linoleoyl serine | 3.38 | .087 | ↓ | 0.79 |
| *N*-arachidonoyl serine | 3.14 | .098 | ↓ | 0.53 |
| 2-oleoyl glycerol | 64.74 | .000 | ↓ | 0.74 |
| 2-linoleoyl glycerol | 12.84 | .003 | ↓ | 0.77 |
| 2-arachidonoyl glycerol | 22.35 | .000 | ↓ | 0.80 |
| Arachidonic acid | 83.96 | .000 | ↓ | 0.78 |
| phosphoLEA | 13.24 | .003 | ↓ | 0.70 |
| PGE_2_ | 4.63 | .049 | ↑ | 1.17 |
| 6-ketoPGF_1α_ | 4.39 | .055 | ↑ | 1.29 |
| CP 55,940 | 749.67 | .000 | ↑ | infinite |

**Supplemental Table 47.** Lipid levels in the brainstem of adult WT female mice treated with Vehicle or 3 mg/kg CP 55,940

|  | Brainstem | | | | | | |
| --- | --- | --- | --- | --- | --- | --- | --- |
|  | Vehicle | | | 3mg/kg CP 55,940 | | | |
| ***N*-acyl alanine** | Mean | Std Dev | Std Error | | Mean | Std Dev | Std Error |
| *N*-palmitoyl alanine | 3.35E-12 | 1.09E-12 | 3.84E-13 | | 3.16E-12 | 1.49E-12 | 5.28E-13 |
| *N*-stearoyl alanine | 5.12E-12 | 1.4E-12 | 4.93E-13 | | 5.4E-12 | 2.08E-12 | 7.36E-13 |
| *N*-oleoyl alanine | 3.19E-12 | 1.33E-12 | 4.69E-13 | | 3.26E-12 | 1.2E-12 | 4.24E-13 |
| *N*-linoleoyl alanine | 2.36E-13 | 1.44E-13 | 5.08E-14 | | 2E-13 | 4.58E-14 | 1.62E-14 |
| *N*-arachidonoyl alanine | 1.28E-12 | 4.62E-13 | 1.63E-13 | | 9.45E-13 | 4.92E-13 | 1.74E-13 |
| *N*-docosahexaenoyl alanine | 3.86E-13 | 2E-13 | 7.07E-14 | | 3.28E-13 | 1.68E-13 | 5.95E-14 |
| ***N*-acyl dopamine** |  |  |  | |  |  |  |
| *N*-oleoyl dopamine | BDL |  |  | | BDL |  |  |
| *N*-arachidonoyl dopamine | BDL |  |  | | BDL |  |  |
| ***N*-acyl ethanolamine** |  |  |  | |  |  |  |
| *N*-palmitoyl ethanolamine | 9.01E-11 | 9.69E-12 | 3.42E-12 | | 8.74E-11 | 9.41E-12 | 3.33E-12 |
| *N*-stearoyl ethanolamine | 2.81E-11 | 7.28E-12 | 2.75E-12 | | 3.01E-11 | 1.5E-11 | 5.67E-12 |
| *N*-oleoyl ethanolamine | 3.21E-10 | 8.06E-11 | 3.05E-11 | | 2.44E-10 | 7.35E-11 | 2.78E-11 |
| *N*-linoleoyl ethanolamine | 9.53E-12 | 1.53E-12 | 5.41E-13 | | 8.37E-12 | 1.45E-12 | 5.14E-13 |
| *N*-arachidonoyl ethanolamine | 1.34E-11 | 3.87E-12 | 1.46E-12 | | 1.14E-11 | 3.68E-12 | 1.39E-12 |
| *N*-docosahexaenoyl ethanolamine | 3.01E-11 | 7.76E-12 | 2.93E-12 | | 2.85E-11 | 9.06E-12 | 3.42E-12 |
| ***N*-acyl GABA** |  |  |  | |  |  |  |
| *N*-palmitoyl GABA | 1.77E-11 | 1.44E-12 | 5.08E-13 | | 1.95E-11 | 3.9E-12 | 1.38E-12 |
| *N*-stearoyl GABA | 1.31E-11 | 1.37E-12 | 4.84E-13 | | 1.37E-11 | 1.97E-12 | 6.95E-13 |
| *N*-oleoyl GABA | 1.61E-11 | 8.35E-13 | 2.95E-13 | | 1.65E-11 | 2.75E-12 | 9.72E-13 |
| *N*-linoleoyl GABA | 5.57E-12 | 1.03E-12 | 3.66E-13 | | 6.22E-12 | 1.6E-12 | 5.64E-13 |
| *N*-arachidonoyl GABA | 1.1E-11 | 8.58E-13 | 3.03E-13 | | 1.08E-11 | 1.75E-12 | 6.17E-13 |
| *N*-docosahexaenoyl GABA | 7.35E-12 | 1.23E-12 | 4.36E-13 | | 7.61E-12 | 1.91E-12 | 6.74E-13 |
| ***N*-acyl glycine** |  |  |  | |  |  |  |
| *N*-palmitoyl glycine | 2.41E-12 | 7.84E-13 | 2.77E-13 | | 2.79E-12 | 1.25E-12 | 4.43E-13 |
| *N*-stearoyl glycine | 4.92E-12 | 2.8E-12 | 9.89E-13 | | 4.95E-12 | 3.98E-12 | 1.41E-12 |
| *N*-oleoyl glycine | 2.08E-12 | 1.11E-12 | 3.91E-13 | | 2.1E-12 | 1.98E-12 | 6.98E-13 |
| *N*-linoleoyl glycine | 1.85E-13 | 6.9E-14 | 2.44E-14 | | 1.41E-13 | 5.79E-14 | 2.05E-14 |
| *N*-arachidonoyl glycine | 2.1E-12 | 1.04E-12 | 3.69E-13 | | 1.87E-12 | 1.14E-12 | 4.03E-13 |
| *N*-docosahexaenoyl glycine | 5.24E-13 | 2.24E-13 | 7.9E-14 | | 5.52E-13 | 3.93E-13 | 1.39E-13 |
| ***N*-acyl leucine** |  |  |  | |  |  |  |
| *N*-palmitoyl leucine | 2.35E-12 | 5.04E-13 | 1.78E-13 | | 3.05E-12 | 6.88E-13 | 2.43E-13 |
| *N*-stearoyl leucine | 1.74E-12 | 2.83E-13 | 9.99E-14 | | 1.96E-12 | 7.38E-13 | 2.61E-13 |
| *N*-oleoyl leucine | 1.78E-12 | 6.05E-13 | 2.14E-13 | | 2E-12 | 7.85E-13 | 2.77E-13 |
| *N*-linoleoyl leucine | PISSR |  |  | | PISSR |  |  |
| *N*-docosahexaenoyl leucine | PISSR |  |  | | PISSR |  |  |
| ***N*-acyl methionine** |  |  |  | |  |  |  |
| *N*-palmitoyl methionine | 4.69E-12 | 9.07E-13 | 3.21E-13 | | 5.07E-12 | 1.12E-12 | 3.96E-13 |
| *N*-stearoyl methionine | 5.65E-13 | 2.17E-13 | 7.66E-14 | | 4.69E-13 | 1.48E-13 | 5.22E-14 |
| *N*-oleoyl methionine | 4.52E-13 | 1.84E-13 | 6.52E-14 | | 4.11E-13 | 1.15E-13 | 4.06E-14 |
| *N*-linoleoyl methionine | BDL |  |  | | BDL |  |  |
| *N*-arachidonoyl methionine | PISSR |  |  | | PISSR |  |  |
| *N*-docosahexaenoyl methionine | PISSR |  |  | | PISSR |  |  |
| ***N*-acyl phenylalanine** |  |  |  | |  |  |  |
| *N*-palmitoyl phenylalanine | 2.63E-13 | 2.67E-13 | 9.45E-14 | | 2.38E-13 | 9.52E-14 | 3.36E-14 |
| *N*-stearoyl phenylalanine | 2.05E-13 | 1.44E-13 | 5.1E-14 | | 1.53E-13 | 5.12E-14 | 1.81E-14 |
| *N*-oleoyl phenylalanine | 4.89E-13 | 3.27E-13 | 1.16E-13 | | 4.28E-13 | 1.14E-13 | 4.02E-14 |
| *N*-linoleoyl phenylalanine | PISSR |  |  | | PISSR |  |  |
| *N*-arachidonoyl phenylalanine | 5.38E-14 | 1.85E-14 | 6.54E-15 | | 5.84E-14 | 2.02E-14 | 7.15E-15 |
| *N*-docosahexaenoyl phenylalanine | 2.37E-14 | 4.23E-14 | 1.5E-14 | | 3.64E-14 | 2.17E-14 | 7.69E-15 |

**Supplemental Table 47: Continued**

|  | Brainstem | | | | | |
| --- | --- | --- | --- | --- | --- | --- |
|  | Vehicle | | | 3mg/kg CP 55,940 | | |
| ***N*-acyl proline** | Mean | Std Dev | Std Error | Mean | Std Dev | Std Error |
| *N*-palmitoyl proline | BDL |  |  | BDL |  |  |
| *N*-stearoyl proline | BDL |  |  | BDL |  |  |
| *N-*oleoyl proline | BDL |  |  | BDL |  |  |
| *N*-linoleoyl proline | BDL |  |  | BDL |  |  |
| *N*-arachidonoyl proline | BDL |  |  | BDL |  |  |
| *N*-docosahexaenoyl proline | BDL |  |  | BDL |  |  |
| ***N*-acyl serine** |  |  |  |  |  |  |
| *N*-palmitoyl serine | 8E-12 | 2.81E-12 | 9.95E-13 | 1.08E-11 | 4.98E-12 | 1.76E-12 |
| *N*-stearoyl serine | 1.16E-11 | 2.28E-12 | 8.05E-13 | 1.31E-11 | 2.17E-12 | 7.67E-13 |
| *N*-oleoyl serine | 9.8E-11 | 3.31E-11 | 1.17E-11 | 1.03E-10 | 3.27E-11 | 1.16E-11 |
| *N*-linoleoyl serine | 2.11E-11 | 8.82E-12 | 3.12E-12 | 1.98E-11 | 5.43E-12 | 1.92E-12 |
| *N*-arachidonoyl serine | PISSR |  |  | PISSR |  |  |
| *N*-docosahexaenoyl serine | PISSR |  |  | PISSR |  |  |
| ***N*-acyl taurine** |  |  |  |  |  |  |
| *N*-arachidonoyl taurine | 2.8E-11 | 2.18E-12 | 7.71E-13 | 2.62E-11 | 1.51E-12 | 5.33E-13 |
| ***N*-acyl tryptophan** |  |  |  |  |  |  |
| *N*-palmitoyl tryptophan | BDL |  |  | BDL |  |  |
| *N*-stearoyl tryptophan | BDL |  |  | BDL |  |  |
| *N*-oleoyl tryptophan | BDL |  |  | BDL |  |  |
| *N*-linoleoyl tryptophan | BDL |  |  | BDL |  |  |
| *N*-arachidonoyl tryptophan | BDL |  |  | BDL |  |  |
| *N*-docosahexaenoyl tryptophan | BDL |  |  | BDL |  |  |
| ***N*-acyl tyrosine** |  |  |  |  |  |  |
| *N*-palmitoyl tyrosine | 9.48E-13 | 5.91E-13 | 2.09E-13 | 7.4E-13 | 1.44E-13 | 5.09E-14 |
| *N*-stearoyl tyrosine | PISSR |  |  | PISSR |  |  |
| *N-*oleoyl tyrosine | 5.65E-13 | 2.03E-13 | 7.19E-14 | 5.06E-13 | 1.17E-13 | 4.14E-14 |
| *N*-linoleoyl tyrosine | PISSR |  |  | PISSR |  |  |
| *N*-arachidonoyl tyrosine | 9.59E-13 | 3.86E-13 | 1.37E-13 | 6.33E-13 | 2.16E-13 | 7.63E-14 |
| *N*-docosahexaenoyl tyrosine | 6.56E-13 | 3.36E-13 | 1.19E-13 | 5.03E-13 | 1.96E-13 | 6.94E-14 |
| ***N*-acyl valine** |  |  |  |  |  |  |
| *N*-palmitoyl valine | 8.93E-14 | 4.22E-14 | 1.49E-14 | 1.28E-13 | 2.1E-14 | 7.43E-15 |
| *N*- stearoyl valine | 2.43E-14 | 1.3E-14 | 4.6E-15 | 2.21E-14 | 1.3E-14 | 4.6E-15 |
| *N*-oleoyl valine | 1.16E-14 | 3.19E-15 | 1.13E-15 | 1.48E-14 | 1.3E-14 | 4.61E-15 |
| *N*-nervonoyl valine | BDL |  |  | BDL |  |  |
| *N*-linoleoyl valine | PISSR |  |  | PISSR |  |  |
| *N*-docosahexaenoyl valine | BDL |  |  | BDL |  |  |
| **2-acyl glycerols** |  |  |  |  |  |  |
| 2-palmitoyl glycerol | 4.8E-11 | 7.09E-12 | 2.68E-12 | 3.83E-11 | 5.35E-12 | 2.02E-12 |
| 2-oleoyl glycerol | 3.87E-09 | 3.89E-10 | 1.47E-10 | 3.17E-09 | 4.23E-10 | 1.6E-10 |
| 2-linoleoyl glycerol | 3.35E-10 | 5.65E-11 | 2.13E-11 | 2.54E-10 | 3.66E-11 | 1.38E-11 |
| 2-arachidonoyl glycerol | 5.51E-09 | 7.99E-10 | 3.02E-10 | 4.32E-09 | 7.5E-10 | 2.84E-10 |
| **Free Fatty Acids** |  |  |  |  |  |  |
| Oleic acid | 5.33E-10 | 1.1E-10 | 3.9E-11 | 4.28E-10 | 1.03E-10 | 3.64E-11 |
| Linoleic acid | 6.4E-10 | 5.71E-11 | 2.02E-11 | 4.56E-10 | 6.28E-11 | 2.22E-11 |
| Arachidonic acid | 4.77E-10 | 4.85E-11 | 1.72E-11 | 4.08E-10 | 5.95E-11 | 2.1E-11 |
| **PhosphoLEA** |  |  |  |  |  |  |
| PhosphoLEA | 2.05E-12 | 7.55E-13 | 2.67E-13 | 2.15E-12 | 7.61E-13 | 2.69E-13 |
| **Prostaglandins** |  |  |  |  |  |  |
| PGE_2_ | 1.89E-10 | 2.37E-11 | 8.37E-12 | 1.64E-10 | 1.69E-11 | 5.96E-12 |
| PGF_2α_ | 1.61E-10 | 1.54E-11 | 5.45E-12 | 1.69E-10 | 1.97E-11 | 6.98E-12 |
| 6-ketoPGF_1α_ | 2.78E-11 | 1.23E-11 | 4.37E-12 | 2.07E-11 | 9.31E-12 | 3.29E-12 |
| **THC/CP/CBD** |  |  |  |  |  |  |
| Cannabidiol | BDL |  |  | BDL |  |  |
| CP 55,940 | BDL |  |  | 1.52E-10 | 2.23E-11 | 7.88E-12 |
| THC | BDL |  |  | BDL |  |  |
| **THC Metabolites** |  |  |  |  |  |  |
| 11-nor-9-carboxyTHC | BDL |  |  | BDL |  |  |
| 11-OH-THC | BDL |  |  | BDL |  |  |

**Supplemental Table 48.** List of lipids in the brainstem significantly affected by 3 mg/kg CP 55,940: Adult

| Adult Brainstem Significant Differences in One-Way ANOVA | | | | |
| --- | --- | --- | --- | --- |
| Lipid | F | p | Direction (relative to Veh) | Magnitude (x Veh level) |
| *N*-oleoyl ethanolamine | 3.56 | .084 | ↓ | 0.76 |
| *N*-palmitoyl leucine | 5.43 | .035 | ↑ | 1.30 |
| *N*-arachidonoyl taurine | 3.39 | .087 | ↓ | 0.94 |
| *N*-arachidonoyl tyrosine | 4.34 | .056 | ↓ | 0.66 |
| *N*-palmitoyl valine | 5.38 | .036 | ↑ | 1.43 |
| 2-palmitoyl glycerol | 8.44 | .013 | ↓ | 0.80 |
| 2-oleoyl glycerol | 10.44 | .007 | ↓ | 0.82 |
| 2-linoleoyl glycerol | 10.00 | .008 | ↓ | 0.76 |
| 2-arachidonoyl glycerol | 8.22 | .014 | ↓ | 0.78 |
| Oleic acid | 3.88 | .069 | ↓ | 0.80 |
| Linoleic acid | 37.74 | .000 | ↓ | 0.71 |
| Arachidonic acid | 6.49 | .023 | ↓ | 0.86 |
| PGE_2_ | 5.77 | .031 | ↓ | 0.87 |
| CP 55,940 | 371.76 | .000 | ↑ | infinite |
| Sample Mass | 7.54 | .016 | ↓ | 0.93 |

**Supplemental Table 49.** Descriptive statistics for PND 35 CP 55,940 levels measured 2 hours after an acute 3 mg/kg injection

| **Descriptives** | | | | | | |
| --- | --- | --- | --- | --- | --- | --- |
| CP | | | | | | |
|  | N | Mean | Std. Deviation | Std. Error | 95% Confidence Interval for Mean | |
|  |  |  |  |  | Lower Bound | Upper Bound |
| STR | 9 | 5.7413E-011 | 6.12794E-012 | 2.04265E-012 | 5.2702E-011 | 6.2123E-011 |
| HIPP | 9 | 9.6303E-011 | 5.55807E-012 | 1.85269E-012 | 9.2030E-011 | 1.0057E-010 |
| CER | 9 | 7.2155E-011 | 4.77780E-012 | 1.59260E-012 | 6.8483E-011 | 7.5828E-011 |
| THAL | 9 | 8.8715E-011 | 7.48317E-012 | 2.49439E-012 | 8.2962E-011 | 9.4467E-011 |
| CTX | 9 | 1.1107E-010 | 1.02907E-011 | 3.43025E-012 | 1.0316E-010 | 1.1898E-010 |
| HYP | 9 | 5.3113E-011 | 4.16328E-012 | 1.38776E-012 | 4.9913E-011 | 5.6313E-011 |
| MID | 9 | 7.9142E-011 | 5.22387E-012 | 1.74129E-012 | 7.5127E-011 | 8.3158E-011 |
| STEM | 9 | 7.0414E-011 | 8.67690E-012 | 2.89230E-012 | 6.3745E-011 | 7.7084E-011 |
| Total | 72 | 7.8540E-011 | 1.95288E-011 | 2.30149E-012 | 7.3951E-011 | 8.3129E-011 |

**Supplemental Table 50.** Output of one-way ANOVA for PND 35 CP 55,940 levels measured 2 hours after an acute 3 mg/kg injection, showing a main effect of brain area on CP 55,940 levels.

| **ANOVA** | | | | | |
| --- | --- | --- | --- | --- | --- |
| CP | | | | | |
|  | Sum of Squares | df | Mean Square | F | Sig. |
| Between Groups | 2.409E-20 | 7 | 3.442E-21 | 73.804 | 3.382E-28 |
| Within Groups | 2.985E-21 | 64 | 4.663E-23 |  |  |
| Total | 2.708E-20 | 71 |  |  |  |

**Supplemental Table 51.** Descriptive statistics for PND 50 CP 55,940 levels measured 2 hours after an acute 3 mg/kg injection

| **Descriptives** | | | | | | |
| --- | --- | --- | --- | --- | --- | --- |
| CP | | | | | | |
|  | N | Mean | Std. Deviation | Std. Error | 95% Confidence Interval for Mean | |
|  |  |  |  |  | Lower Bound | Upper Bound |
| STR | 9 | 5.8704E-011 | 1.07800E-011 | 3.59332E-012 | 5.0418E-011 | 6.6990E-011 |
| HIPP | 9 | 1.2297E-010 | 1.69141E-011 | 5.63802E-012 | 1.0997E-010 | 1.3597E-010 |
| CER | 9 | 7.5674E-011 | 1.36401E-011 | 4.54671E-012 | 6.5189E-011 | 8.6158E-011 |
| THAL | 9 | 9.9312E-011 | 1.63333E-011 | 5.44443E-012 | 8.6757E-011 | 1.1187E-010 |
| CTX | 9 | 8.6116E-011 | 1.36218E-011 | 4.54060E-012 | 7.5645E-011 | 9.6586E-011 |
| HYP | 9 | 4.3769E-011 | 5.48489E-012 | 1.82830E-012 | 3.9553E-011 | 4.7985E-011 |
| MID | 9 | 1.2091E-010 | 1.57319E-011 | 5.24396E-012 | 1.0882E-010 | 1.3300E-010 |
| STEM | 8 | 8.3747E-011 | 1.89696E-011 | 6.70676E-012 | 6.7888E-011 | 9.9606E-011 |
| Total | 71 | 8.6438E-011 | 2.96829E-011 | 3.52271E-012 | 7.9412E-011 | 9.3464E-011 |

**Supplemental Table 52.** Output of one-way ANOVA for PND 50 CP 55,940 levels measured 2 hours after an acute 3 mg/kg injection, showing a main effect of brain area on CP 55,940 levels.

| **ANOVA** | | | | | |
| --- | --- | --- | --- | --- | --- |
| CP | | | | | |
|  | Sum of Squares | df | Mean Square | F | Sig. |
| Between Groups | 4.861E-20 | 7 | 6.944E-21 | 33.486 | 6.341E-19 |
| Within Groups | 1.306E-20 | 63 | 2.074E-22 |  |  |
| Total | 6.168E-20 | 70 |  |  |  |

**Supplemental Table 53.** Descriptive statistics for adult CP 55,940 levels measured 2 hours after an acute 3 mg/kg injection

| **Descriptives** | | | | | | |
| --- | --- | --- | --- | --- | --- | --- |
| CP | | | | | | |
|  | N | Mean | Std. Deviation | Std. Error | 95% Confidence Interval for Mean | |
|  |  |  |  |  | Lower Bound | Upper Bound |
| STR | 8 | 2.0821E-010 | 2.80317E-011 | 9.91069E-012 | 1.8477E-010 | 2.3164E-010 |
| HIPP | 8 | 2.6840E-010 | 3.38004E-011 | 1.19502E-011 | 2.4014E-010 | 2.9666E-010 |
| CER | 8 | 2.8002E-010 | 4.05329E-011 | 1.43305E-011 | 2.4614E-010 | 3.1391E-010 |
| THAL | 8 | 2.0002E-010 | 2.21374E-011 | 7.82677E-012 | 1.8151E-010 | 2.1853E-010 |
| CTX | 8 | 1.8513E-010 | 2.50694E-011 | 8.86337E-012 | 1.6417E-010 | 2.0608E-010 |
| HYP | 8 | 1.1726E-010 | 2.16123E-011 | 7.64111E-012 | 9.9196E-011 | 1.3533E-010 |
| MID | 8 | 2.2043E-010 | 2.27713E-011 | 8.05086E-012 | 2.0140E-010 | 2.3947E-010 |
| STEM | 8 | 1.5203E-010 | 2.23015E-011 | 7.88477E-012 | 1.3338E-010 | 1.7067E-010 |
| Total | 64 | 2.0394E-010 | 5.76930E-011 | 7.21163E-012 | 1.8953E-010 | 2.1835E-010 |

**Supplemental Table 54.** Output of one-way ANOVA for adult CP 55,940 levels measured 2 hours after an acute 3 mg/kg injection, showing a main effect of brain area on CP 55,940 levels.

| **ANOVA** | | | | | |
| --- | --- | --- | --- | --- | --- |
| CP | | | | | |
|  | Sum of Squares | df | Mean Square | F | Sig. |
| Between Groups | 1.665E-19 | 7 | 2.378E-20 | 30.825 | 5.160E-17 |
| Within Groups | 4.321E-20 | 56 | 7.716E-22 |  |  |
| Total | 2.097E-19 | 63 |  |  |  |
